# Ovarian Cancer‐Associated Ascites Accumulates NK Lymphocytes Displaying a Transcriptomic Profile Consistent With the Immature/Secretory CD56^bright^ Subtype

**DOI:** 10.1111/imcb.70159

**Published:** 2026-08-20

**Authors:** Caroline Natânia de Souza‐Araújo, Glenerson Baptista, Gabriela Rapozo Guimarães, Raissa Carolina Alves da Silva, Adriana Yoshida, Cláudia Rodrigues Tonetti, José Andrés Yunes, Daniela Maira Cardoso, Sophie Derchain, Mariana Boroni, Fernando Guimarães

**Affiliations:** ^1^ School of Medicine Sciences, University of Campinas Campinas SP Brazil; ^2^ Laboratory of Bioinformatics and Computational Biology, Division of Experimental and Translational Research Brazilian National Cancer Institute (INCA) Rio de Janeiro RJ Brazil; ^3^ Women's Hospital José Aristodemo Pinotti‐Centro de Atenção Integral à Saúde da Mulher (CAISM) University of Campinas Campinas SP Brazil; ^4^ Centro Infantil de Investigações Hematológicas Dr Domingos A Boldrini Campinas SP Brazil

**Keywords:** high‐grade serous ovarian cancer, NK‐cell subtypes, RNA sequencing; tumor environment

## Abstract

Natural killer (NK) cells are important lymphocytes of the innate immune response against cancer. Based on their molecular and functional phenotypes, NK cells are categorized into two main subtypes known as CD56^dim^ and CD56^bright^, which display distinct transcriptional profiles. Additionally, transcriptomic alterations may arise in response to different stimuli or microenvironmental conditions, ultimately affecting NK‐cell functions. Here, RNA sequencing (RNAseq) was performed to characterize the transcriptomic profile of NK cells from epithelial ovarian cancer (EOC)‐associated ascites. Two bulk RNAseq datasets were analyzed: one generated from samples collected from patients assisted at the Women's Hospital of the University of Campinas (CAISM‐Unicamp, Brazil) and a second publicly available dataset obtained from the Gene Expression Omnibus database (GSE153713). Our transcriptomic analysis identified 5427 differentially expressed genes (DEGs; adjusted *p* < 0.05), revealing extensive differences between ascites and blood NK cells. Furthermore, these findings indicated a possible shift in the biological program of ascites NK cells, from the conventional cytotoxic CD16^+^CD56^dim^ subtype to an immature/secretory CD56^bright^ profile. Comparison of ascites and blood NK RNAseq based on a previously defined transcriptional framework showed reduced NK1 and increased NK2 subtype scores in ascites, confirming association with distinct biological programs. In conclusion, ascites NK cells retain elements of the conventional NK‐cell program while displaying a transcriptional profile distinct from peripheral blood NK cells, consistent with transcriptional remodeling to biological programs usually associated with the CD56^bright^ NK subtype.

## Graphical abstract

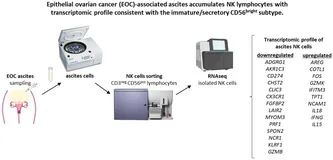

## 1 Introduction

Natural killer (NK) cells are cytotoxic innate lymphoid cells known for the ability to kill malignant cells and restraining metastatic spread of cancer [1, 2, 3, 4]. The cytotoxic action of NK cells against cancer is driven by the loss of self‐recognition signaling molecules added to the expression of stress molecules on malignant cells [5, 6, 7]. In addition, the presence of antibodies directed to tumor epitopes can also drive cytotoxicity through the NK CD16 (FcRIIIa) receptor, allowing NK cells to recognize antibody‐opsonized malignant cells as cellular targets [8, 9, 10]. NK cells comprise up to 15% of the peripheral blood lymphocytes in humans, where they can be differentiated from other lymphocyte populations by their cell surface molecular phenotype CD3^−^CD56^+^. Furthermore, the relative expression of the CD56 molecule allows categorizing NK cells in two subsets known as CD56^bright^ and CD56^dim^, in accordance with cells' relative emission of fluorescence intensity, when labeled with fluorochrome‐conjugated monoclonal antibody (MAb) anti‐CD56 molecule [2, 11].

The CD56^dim^ subset accounts for more than 80% of NK lymphocytes present in peripheral blood, while the CD56^bright^ subset prevails in lymphoid tissues and overcomes the body total number of CD56^dim^ NK cells [2, 11]. Although related in their origin, the CD56^dim^ subset mediates cytotoxic antitumor responses, whereas the CD56^bright^ is linked to the modulation of immune responses, as well as the precursor stages of NK lymphocyte maturation. Thus, CD56^bright^ NK cells are primarily cytokine producers, displaying lower antitumor cytotoxicity when compared to the CD56^dim^ subset [12, 13], although the antitumor response of CD56^bright^ NK cells can be markedly enhanced by interleukins (IL)‐2 and IL‐15 [14, 15, 16]. Interestingly, NK responses to specific stimuli or different tissue environments can modulate their genetic, molecular, and functional phenotypes, giving rise to the diversification of their subsets, as observed in pregnancy, immunological responses against virus infections, or NK maturation [17, 18, 19].

Ovarian cancer is a highly lethal disease, although it is not the most frequent malignancy that can affect a woman's health. Such high lethality results from the advanced stage at which the disease is often diagnosed. Accordingly, 70%–75% of women with ovarian cancer are diagnosed at advanced stages, resulting in the worst 5‐year survival rate (20%–25%) among all gynecological malignancies [20, 21, 22]. Although ovarian cancer comprises multiple subtypes, 90% of occurrences are of epithelial origin, of which the serous histotype is the most prevalent, comprising 70% of all epithelial ovarian cancers (EOC). The high‐grade serous EOC is the most aggressive subtype and accounts for two‐thirds of all ovarian cancer deaths [23, 24, 25]. The initial development of EOC is asymptomatic and lacks specific early‐detection biomarkers. Furthermore, its progression is characterized by the spread of malignant cell aggregates through the abdominal cavity, resulting in intraperitoneal seeding [26]. As a consequence, first symptoms of EOC, such as bloating or pain, are frequently associated with the presence of metastases beyond the ovaries [27].

Peritoneal ascites is the pathological accumulation of fluid in the peritoneal cavity, being a frequent comorbidity in EOC intraperitoneal dissemination which, in addition, can be observed at initial diagnosis of advanced cancer (stages III and IV), as well as in almost all cases of relapse [28, 29, 30]. Although attributed to ascites a facilitating effect on the metastatic spread of EOC, ascites may or may not contain detectable malignant cells. Additionally, ascites also displays different signaling molecules and cell types, such as VEGF, IL‐6, IL‐10, fibroblasts, adipocytes, mesothelial, endothelial, lymphocytes, and inflammatory cells [31, 32, 33, 34]. NK lymphocytes are also present in ascites, where their antitumor action has been shown to be impaired either by immunomodulatory or tumor escape mechanisms. However, knowledge about transcriptomic alterations underlying NK‐cell dysfunctionality in ascites is limited.

Thus, the objective of this study was to evaluate NK cells isolated from ascites of EOC patients at the RNA expression level. To this end, two NK‐cell bulk RNA sequencing (RNAseq) datasets were analyzed together, one generated from sampling of patients assisted at the Women's Hospital (CAISM) of the University of Campinas (Unicamp, SP, Brazil), and the other made available at Gene Expression Omnibus database by the accession number GSE153713 [35]. Our results showed extensive transcriptomic alterations in ascites NK cells, which affected the RNA expression of genes encoding molecules involved in cytotoxic function, as well as molecules with the potential to promote a shift in the functional profile of NK cells.

## 2 Results

To evaluate NK cells from EOC‐associated environment at the transcriptomic level, RNAseq was performed in NK cells isolated from ascites and blood of patients assisted at the Women's Hospital (CAISM) of the University of Campinas (UNICAMP, Campinas, SP, Brazil). Cell sorting resulted in NK enriched cell suspensions presenting purity > 95% of CD3^−^CD56^+^ lymphocytes. The resulting NK‐cell RNAseq of the CAISM series was combined with a preexisting dataset of RNAseq (GSE153713) similarly carried out using isolated NK cells of ascites samples from advanced EOC patients as well as blood samples from healthy donors [35].

Hierarchical clustering analysis of bulk RNAseq organized the NK‐cell samples from blood and EOC‐associated ascites according to their level of similarity between transcriptomes (Figure 1a). As a result, blood samples from the CAISM and GSE153713 series were closely related (Figure 1a, Cluster 2). In addition, the analysis also showed that three of the ascites NK‐cell samples belonging to the CAISM series shared transcriptomic similarities with blood samples. However, most of the ascites NK‐cell samples were assembled apart from blood samples (Figure 1a, Cluster 1), and principal component analysis interpreted ascites and blood as two distinct groups, with the PC1 and PC2 components explaining 61.9% of the total variance between the groups (Figure 1b).

**FIGURE 1:**
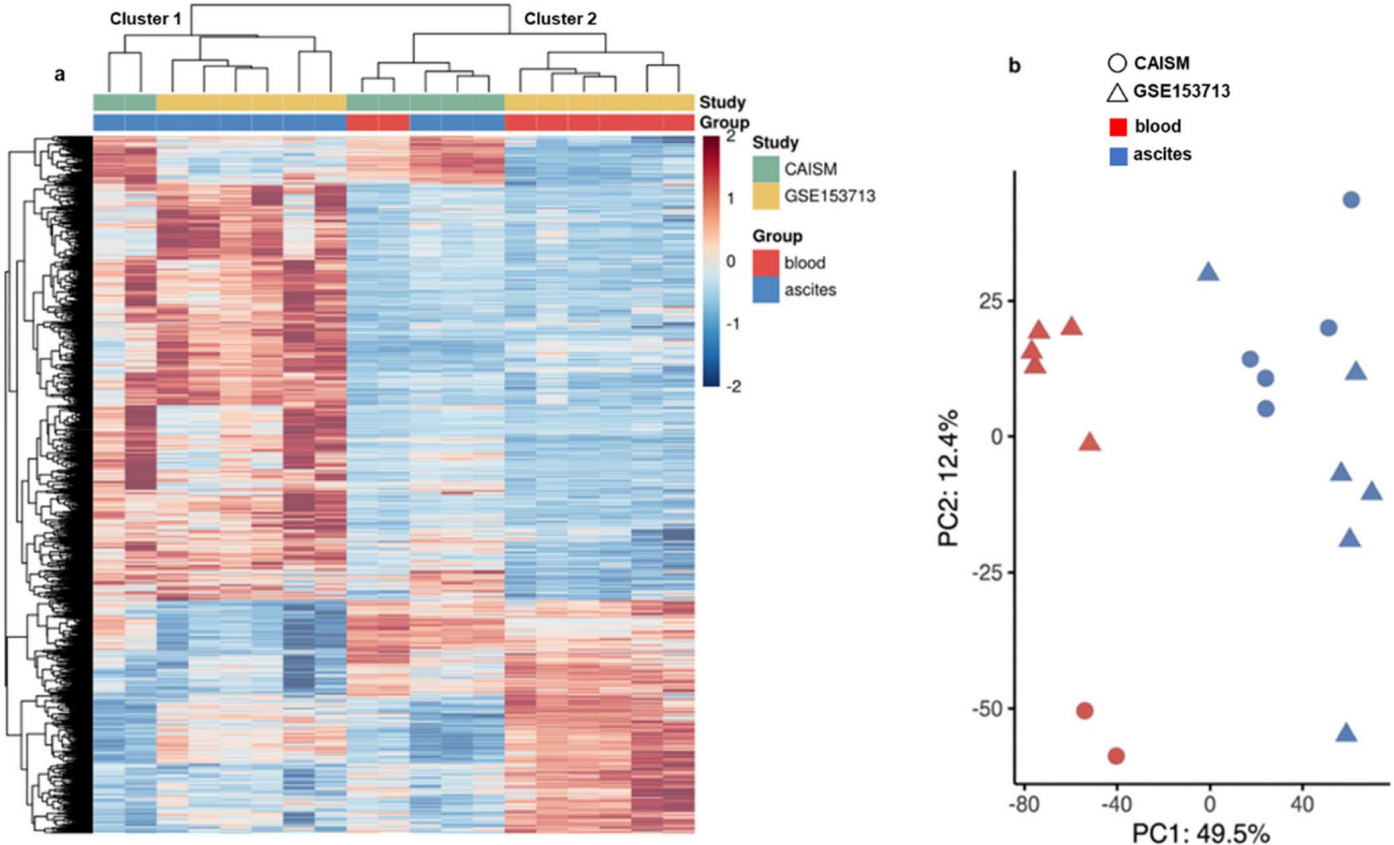
Global transcriptomic profiling of NK cells isolated from epithelial ovarian cancer‐associated ascites and blood. (a) Heatmap showing hierarchical clustering of the top (*p* adjusted < 0.001) differentially expressed genes (DEG) across ascites and blood NK‐cell samples from the CAISM and GSE153713 datasets. Gene expression is displayed as gene‐wise *z*‐scores. (b) Principal component analysis (PCA) of variance‐stabilized expression data after removal of unwanted variation using RUVr. PC1 and PC2 explain 61.9% of the total variance.

Comparison between transcriptomes of NK‐cell samples from ascites and blood identified 5427 genes differentially expressed (DEG; adjusted *p* < 0.05), being 3202 upregulated and 2225 downregulated in the ascites (Table S2). Gene enrichment analysis of DEG (GSEA), combining statistical criteria and biological relevance, allowed to specify not only potentially altered biological processes in ascites NK cells but also gene subsets that account for the enrichment signal along three main axes: the signal transduction and intracellular signaling pathways as well as the modulation of cellular effector function (Figure 2a,b).

**FIGURE 2:**
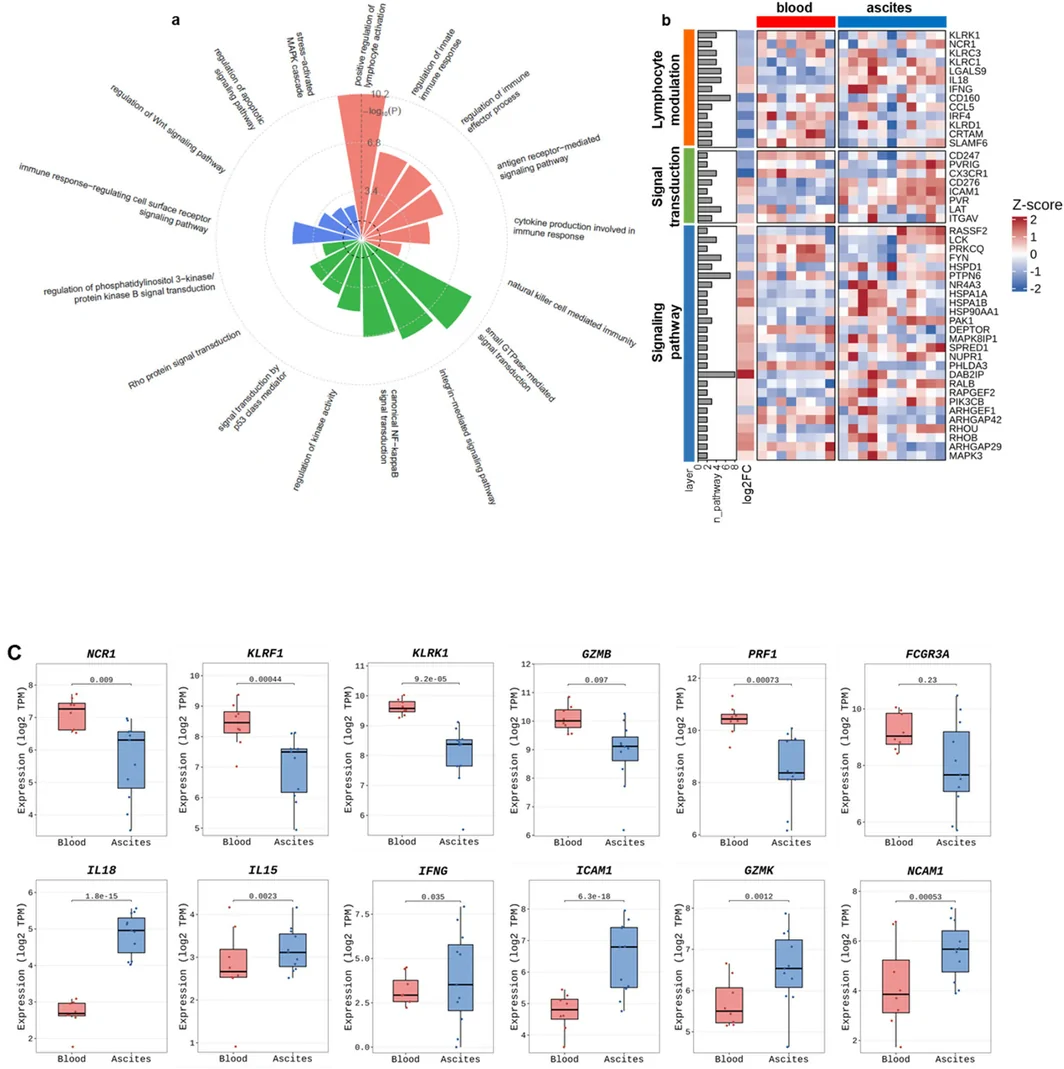
Global enrichment analysis of differentially expressed genes in NK cells from ovarian cancer‐associated ascites and blood. (a) Representative Gene Ontology (GO) biological processes identified by gene set enrichment analysis (GSEA) and organized into three major functional axes: signal transduction, intracellular signaling, and modulation of cellular effector function. Bar length is proportional to enrichment significance. (b) Heatmap showing representative genes related to the enriched biological processes displayed in panel (a), grouped according to their corresponding functional axis. (c) Expression levels (TPM) of selected canonical NK‐cell genes in blood and ascites samples. Adjusted *p* values were obtained from differential expression analysis using DESeq2 (Wald test with Benjamini–Hochberg correction).

The results also showed that genes such as *NCR1*, *KLRF1*, *KLRK1*, *GZMB*, *PRF1*, *FCGR3A*, *CLIC3*, *LAIR2*, *SPON2*, and *CD160* typically expressed in conventional cytotoxic NK cells from peripheral blood, comprising the mature CD16^+^CD56^dim^ NK subtype, were found downregulated in ascites compared with blood. Concomitantly, the cytokine‐encoding genes *IL18*, *IL15*, and *IFNG*, as well as genes such as *NCAM1*, *ICAM1*, and *GZMK*, which are frequently associated with the CD56^bright^ subtype, were upregulated in ascites NK cells (Figure 2c). These results suggest that, instead of being random, alterations in the transcriptomic profile of ascites NK cells reflect a possible shift in the biological program. Thus, to confirm the association between the transcriptomic profiles found and the biological programs of specific NK cell subtypes, ascites and blood NK‐cell samples were additionally compared using a transcriptional framework previously defined by Rebuffet et al. [36] to identify the NK1, NK2, and NK3 subtypes of NK cells, which comprise the CD16^+^CD56^dim^, CD56^bright^, and adaptive NK subtypes, respectively. As a result, the score of the NK1 subtype was found significantly lower (*p* = 0.00016) in ascites, whereas the NK2 subtype was significantly higher (*p* = 0.0075) compared with blood (Figure 3a,b; Table S3).

**FIGURE 3:**
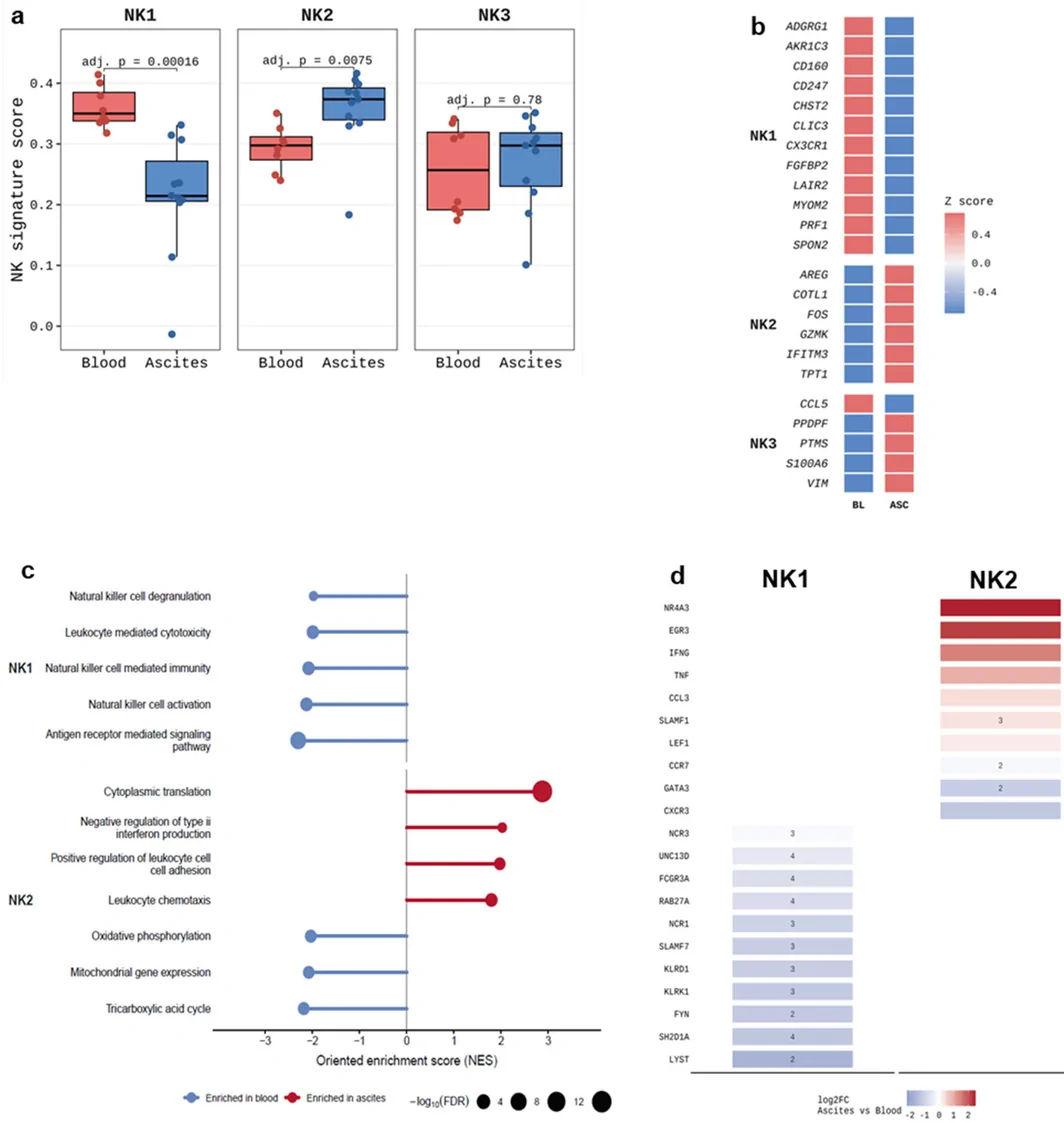
NK‐cell transcriptional signatures associated with ascites and blood samples. (a) Comparison of NK1, NK2, and NK3 signature scores between ascites and blood samples. Scores were calculated using the singscore method. Statistical comparisons were performed using the Wilcoxon rank‐sum test with Benjamini–Hochberg correction for multiple testing. (b) Heatmap showing the expression patterns of genes composing the NK1, NK2, and NK3 signatures across blood (BL) and ascites (ASC) samples. Gene expression is displayed as gene‐wise *z*‐scores. (c) Score‐oriented enrichment analysis based on NK1 and NK2 signature scores. Representative Gene Ontology (GO) biological processes associated with each transcriptional program are shown according to their normalized enrichment scores (NES). Blue symbols indicate enrichment in blood, whereas red symbols indicate enrichment in ascites. The position and length of each segment represent the magnitude and direction of the NES. Point size is proportional to enrichment significance (−log10 adjusted *p*‐value). (d) Representative leading‐edge genes contributing to the enrichment of NK1‐ and NK2‐associated biological processes. Color scale represents log2 fold change (Ascites vs. Blood). Numbers within boxes indicate recurrence across enriched pathways. Additional enriched pathways and corresponding leading‐edge genes are provided in Table S4.

Furthermore, enrichment analysis employing the gene expression scores established for NK1 and NK2 subtypes allowed correlating biological processes potentially affected by transcriptomic alterations between ascites and blood with the NK‐cell subtype. As a result, distinct biological processes were associated with the NK1 or NK2 subtypes based on their normalized enrichment scores (NES), where negative NES values indicate enrichment in the blood, whereas positive values indicate enrichment in the ascites (Figure 3c). Interestingly, canonical programs of cellular effector function, such as activation, degranulation, cytotoxicity, and antigen receptor‐mediated signaling, were linked to the NK1 subtype, as well as to its enrichment in the blood. In contrast, the NK2 subtype was associated with biological programs related to immune regulation, mitochondrial metabolism, and cell adhesion and migration. In this context, biological processes such as leukocyte chemotaxis, positive regulation of cell–cell adhesion, negative regulation of type II interferon production, and cytoplasmic translation showed enrichment in ascites, concomitantly to the enrichment of oxidative phosphorylation, tricarboxylic acid cycle, and mitochondrial gene expression in blood (Figure 3c, Table S4). Evaluation of the core genes accounting for enrichment showed that, although genes such as *FCGR3A*, *NCR1*, *KLRD1*, and *KLRK1* were linked to the NK1 subtype and recurrent across processes of effector cytotoxic function, they were downregulated in ascites. In contrast, leading‐edge genes accounted for enrichment of cellular programs linked to the immunoregulation and cell interactions that were associated with the NK2 subtype and upregulation in ascites (Figure 3d). Taken together, the results showed that NK cells present in EOC‐associated ascites display a transcriptional profile distinct from peripheral blood NK cells, encompassing biological programs usually associated with the CD56^brigth^ NK‐cell functions.

## 3 Discussion

Ascites represents a valuable source for investigating the environment associated with EOC and the mechanisms that sustain tumor progression. In this context, studies based on sampling of ascites have enabled the characterization of lymphocyte subpopulations within the EOC‐associated environment and helped to identify different immunomodulatory mechanisms that impair the antitumor action of cytotoxic lymphocytes [33, 34]. Likewise, the present study was successful in combining two bulk RNAseq datasets to evaluate NK cells isolated from the ascites of patients with EOC at the transcriptomic level, providing further insight into the transcriptional profile of NK cells and alterations associated with the functional impairment of NK cells in the tumor‐associated environment.

In this sense, our results showed extensive differences in RNA expression between NK cells from ascites and blood. Furthermore, despite the occurrence of variability among ascites samples from different patients, a prevailing pattern of alterations was noticed in the RNA expression of ascites NK cells. These findings corroborate the dominant effect of ascites in modulating NK‐cell RNA expression, as demonstrated by Fraser et al. [35], who reported similar transcriptomic changes shared by ascites NK cells and blood NK cells from healthy donors that were incubated in ascites from patients with EOC. Our results also corroborate previous findings showing that the transcriptomic alterations observed in ascites NK cells were consistent with an overall suppression of signal transduction pathways and intracellular signaling [35]. In this context, the shift from the canonical MAPK pathway to p38 has been linked to biological programs driving cytokine production and modulatory functions in NK cells, features typically attributed to the CD56^bright^ subpopulation [37]. Considering that the CD56^bright^ subset is expanded in ovarian cancer‐associated ascites, and that our results revealed an upregulation of genes encoding p38 pathway‐related proteins (such as *DAB2IP*, *SPRY4*, *DUSP1*, *MAPK8IP1*, and *MAPK11*), we hypothesize that this pathway could play a role in the adaptation of NK cells within the tumor environment. Nonetheless, further functional assays evaluating protein expression are required to validate this hypothesis.

The resulting RNA profile was the effect of a dynamic process that combined NK cells at different stages of adaptation to the EOC‐associated environment, which, in turn, varied in its molecular and cellular composition among different patients. Overall, our results showed that NK cells present in ascites samples lacked an RNA profile consistent with that of the conventional cytotoxic NK cells from blood, which is characterized by the expression of genes that encode activating receptors (*NCR1*, *KLRK1*), as well as *PRF1* and *GZMB*, among others. Alternatively, we observed the expression of genes that encode cytokines (*IL18*, *IFNG*) and markers such as *ICAM1* and *GZMK*, which supports the idea that ascites NK cells have a transcriptomic profile consistent with the CD56^bright^ subtype.

Accordingly, previous studies have shown that EOC‐associated ascites not only accumulates NK CD56^bright^ (Figure S1) and T‐regulatory (T‐reg) lymphocytes [16, 38, 39, 40] but also contains ascites NK cells with low expression of activating receptors, such as DNAM‐1, NKp30, CD16, and NKG2D. This has been explained as a downregulation of these receptors due to excessive exposure to their ligands in the tumor environment [16, 39, 40, 41]. Additionally, in contrast to the blood of EOC patients, ascites accumulates NK and T lymphocytes expressing the PD‐1 molecule, which is a hallmark of the development of lymphocyte exhaustion processes [42, 43]. Here, our results extended the phenotypic alterations affecting NK cells in the ascites of EOC patients to the transcriptomic level, indicating that, similar to other tumor environments, impairment of cellular cytotoxicity in ascites may accrue from more comprehensive changes that lead to the functional conversion of NK cells.

The biological implication of the presence of CD56^bright^ NK cells in the ascites of EOC patients has been explained in terms of an immunomodulatory effect similar to that occurring in fetal maternal tolerance, where the CD56^bright^ subset plays a role in placenta homeostasis [17, 38, 44]. Moreover, the CD56^bright^ NK cells have been found infiltrating breast, colon, and lung cancers, where they undergo transcriptomic alterations, eliciting an angiogenic profile that has been implicated in the development of a tumor‐supportive environment [17, 45, 46]. It has also been reported that NK cells infiltrating renal carcinoma begin to express genes associated with angiogenesis and inflammation, which under the influence of hypoxia, converts the transcriptomic phenotype of these infiltrating NK cells into one similar to tissue‐resident NK cells [47, 48].

While our findings have shown that ascites NK cells differ substantially from conventional blood NK cells, they did not clarify whether such alterations show isolated transcriptional changes or broader shifts in NK cell‐associated biological programs. To further investigate these biological programs associated with the transcriptional alterations observed in ascites NK cells, we employed the transcriptional framework recently proposed by Rebuffet et al. [36], which describes three major NK‐cell transcriptional programs, designated as NK1, NK2, and NK3. These programs broadly correspond to cytotoxic/terminally mature, regulatory‐secretory and adaptive HCMV‐driven NK‐cell states, respectively. Although the biological processes associated with the NK1 gene subset score reflected multiple components of the canonical cytotoxic NK‐cell response, including activating receptor signaling, target‐cell recognition, cytotoxicity, and degranulation, the genes contributing to these pathways spanned distinct stages of this response, including target recognition (*NCR1*, *KLRK1*, and *FCGR3A*) and granule trafficking/exocytosis (*UNC13D*, *RAB27A*, and *LYST*). Nonetheless, this suggests that the transcriptional profile of ascites NK cells diverges from the conventional cytotoxic NK‐cell program typically observed in peripheral blood.

In contrast to the predominantly cytotoxic programs associated with NK1, NK2‐associated pathways pointed to biological programs related to intercellular communication and immune interactions consistent with the regulatory‐secretory programs performed by the CD56^bright^ subtype. These observations are consistent with previous studies in ovarian cancer‐associated ascites showing that NK cells display phenotypic characteristics distinct from those observed in peripheral blood [49]. Interestingly, despite these phenotypic alterations, ascites‐derived NK cells have been reported to retain responsiveness to cytokine stimulation, including IL‐2 and IL‐15 [43]. Furthermore, the transcriptional differences observed between compartments were not limited to pathways associated with immune communication and regulation. Metabolic programs related to oxidative phosphorylation, mitochondrial gene expression, and the tricarboxylic acid cycle were preferentially represented in peripheral blood NK cells, suggesting that the transcriptional divergence observed between compartments also extends to biological processes linked to cellular metabolism [35].

In this study, the analysis of bulk RNA expression of isolated NK cells could have been compromised by genes highly expressed in contaminating cells remaining in the final NK‐cell suspension after sorting. This possibility constitutes a limitation inherent to similar studies, as it may introduce DEGs that do not belong to the target cell subset. In addition, our limitations included the modest size of the blood control group from the CAISM series and the integration of two datasets rather than their independent analysis. However, we believe that our analytical approach—based on correcting for unwanted variation and using gene expression scores that reflect specific NK‐cell transcriptional programs to compare ascites and blood NK transcriptomes—enabled us to overcome these limitations. In conclusion, our results showed that NK cells present in EOC‐associated ascites retain elements of the conventional NK‐cell program while displaying a transcriptional profile distinct from peripheral blood NK cells. This profile encompasses biological programs usually associated with the CD56^bright^ NK‐cell functions, which is consistent with transcriptional remodeling rather than complete replacement of the canonical cytotoxic NK‐cell program.

## 4 Materials and Methods

### 4.1 Patients, Ascites, and Blood Sampling

Women assisted at the Women's Hospital of the University of Campinas (Campinas, Brazil) for the treatment of adnexal masses were included in this study. Patients' clinical and pathological characteristics at the time of sample collection are summarized in Table S1. Signed informed consent was obtained from all patients. The study was approved by the Research Ethics Committee of the University of Campinas (#2.029.221/2017) and registered at the Brazilian National Health Council (CAAE: 65367617.9.0000.5404). Ascites samples from patients with ovarian cancer were collected by ultrasonography‐guided paracentesis, quickly transferred to tubes containing sodium heparin (1 μL/mL of ascites; Liquemine 5000 UI/mL, Roche, Brazil) under sterile conditions. The cellular fraction of the ascites was isolated by centrifugation (centrifuged at 600× *g* for 5 min) and red blood cells eliminated by washing the cell pellet with lysis solution buffer (NH_4_Cl 150 mM, KHCO_3_ 10 mM, Na_2_EDTA 0.1 mM, pH 7.2). Blood samples were collected using 10 mL vacuum blood‐sampling tubes containing sodium heparin (Vacuette, Brazil). Peripheral blood mononuclear cells (PBMC) were isolated by gradient centrifugation, using Ficoll‐Paque Plus (Cytiva, Sweden), followed by a washing procedure performed twice (centrifuged at 600× *g* for 5 min) using a balanced salt solution (PBS; Nutricell Nutrientes Celulares Ltda, Brazil). Cell viability and counting were assessed using trypan blue exclusion in a Neubauer chamber.

### 4.2 NK Cell Sorting

NK cells were isolated from ascites‐derived cellular suspensions and PBMC samples by fluorescence‐activated cell sorting (FACS). Briefly, cells were stained with fluorochrome‐conjugated monoclonal antibodies (MAb) against CD3 (FITC) and CD56 (APC) (BD Biosciences, USA). The staining procedures were carried out by the incubation (30 min/8°C, protected from light) of cell suspensions at 1 × 10^6^ cells/mL with MAb mixture at a pretitled dilution (1:50 in PBS containing 2% fetal bovine serum (FBS) and ethylenediaminetetraacetic acid (EDTA), 2 mM—Invitrogen Life Technologies, USA). Then, the cells were washed by centrifugation to remove unbound antibodies, suspended with PBS containing 0.5% FBS up to the density of 1 × 10^6^ cells/mL, and then submitted to acquisition in FACS sorter (BD FACSAria III) for NK‐cell isolation, which was carried out following a pre‐standardized gating strategy (Figure S2). The resulting enriched NK‐cell suspension was finally centrifuged and resuspended in 150 μL of the guanidine‐sarkosyl solution (4 M guanidine isothiocyanate, 0.5% w/v n‐Lauroylsarcosine sodium salt, 25 mM sodium citrate, 0.1 M 2‐mercaptoethanol, pH 7.0) and stored at −80°C until RNA extraction.

### 4.3 RNA Extraction and NGS Sequencing

Total RNA was extracted from NK‐cell samples of blood and ascites using Zymo Quick‐RNA MicroPrep reagent kit (Zymo Research Corporation, USA) according to the manufacturer's instructions. RNA concentration and integrity were assessed using the Qubit RNA HS Assay Kit (Thermo‐Fischer, USA). Samples with RNA integrity number (RIN) ≥ 7 were used in downstream procedures. mRNA libraries were prepared by the Life Sciences Core Facility (LaCTAD) of University of Campinas. Library quality was assessed by Bioanalyzer capillary electrophoresis, Qubit quantification and real‐time PCR using the KAPA Fast Universal kit (Merck, Germany). Sequencing was performed on the Illumina HiSeq2500 platform using a paired‐end 150 bp (PE150) strategy.

### 4.4 RNAseq Preprocessing and Quantification

Bulk RNAseq analyses were performed using two integrated datasets of isolated NK cells. The CAISM series comprised ascites‐derived NK cells from five patients with advanced high‐grade serous epithelial ovarian cancer (EOC), and peripheral blood‐derived NK cells from two patients with benign adnexal masses. The CAISM dataset is available in the University of Campinas Data Repository (REDU, https://doi.org/10.25824/redu/WRQ10C). The CAISM dataset was integrated with a publicly available RNAseq dataset deposited in the Gene Expression Omnibus repository (GSE153713), comprising ascites‐derived NK cells from six patients with advanced serous EOC and peripheral blood NK cells from six healthy donors. Raw sequencing reads were subjected to quality control using FastQC (v0.11.9), followed by adapter and low‐quality base trimming using Trimmomatic (v0.39) with a Phred quality score cutoff of 20. An RSEM reference was generated from the human GRCh38 genome assembly and the corresponding Ensembl gene annotation (release 108). Cleaned reads were aligned to the resulting transcript reference and quantified using RSEM (v1.3.3), with Bowtie2 (v2.4.4) as the alignment algorithm. Gene expression values were reported as expected counts, transcripts per million (TPM), and fragments per kilobase of transcript per million mapped reads (FPKM).

### 4.5 Differential Expression and Unsupervised Analyses

Differential expression analysis was performed using DESeq2, based on a generalized linear model within a negative binomial framework [50]. The design formula included a sample condition as the variable of interest. Lowly expressed genes were filtered, retaining genes with at least 10 counts in a number of samples equal to or greater than the smallest experimental group. Statistical significance was assessed using the Wald test, and *p*‐values were adjusted using the Benjamini–Hochberg procedure. Genes with false discovery rate (FDR) < 0.05 were considered statistically significant. Unwanted sources of variation were estimated using the RUVr approach, based on residuals obtained from an initial model fit [51] and using the 5000 least variable genes as empirical negative controls. The resulting latent factors (W1 and W2) were used to evaluate the impact of unwanted variation on sample structure. Principal component analysis (PCA) was performed using variance‐stabilized transformed data (blind = FALSE). For visualization purposes, unwanted variation associated with latent factors was regressed out prior to PCA plotting. PCA representations before and after correction were used to assess technical variability and sample clustering. The correction procedure was applied exclusively to exploratory visualizations and did not influence differential expression analyses. Heatmaps were generated using variance‐stabilized expression data, with hierarchical clustering applied to both genes and samples using the ComplexHeatmap package (v2.28.0).

### 4.6 Functional Enrichment Analysis

Functional enrichment analysis was performed using the fgsea package (Fast Gene Set Enrichment Analysis; v1.32.4) in R, following the Gene Set Enrichment Analysis framework [52]. Genes were ranked according to the DESeq2 Wald statistic. Gene sets from the Gene Ontology (GO) Biological Process collection were used as reference. Pathways with adjusted *p*‐value < 0.05 were considered significantly enriched. For visualization and biological interpretation, representative nonredundant pathways were manually curated based on normalized enrichment score (NES), adjusted *p*‐value, biological relevance, and redundancy across related GO terms.

### 4.7 NK Gene Subset Scoring and Score‐Oriented Enrichment Analysis

NK1, NK2, and NK3 gene subsets (Table S3) as described by Rebuffet et al. [36] were used to characterize distinct NK‐cell transcriptional states. These gene subsets or signatures were originally derived from single‐cell human NK‐cell transcriptomic data and represent functionally distinct NK‐cell programs corresponding to cytotoxic/terminally mature (NK1), regulatory‐secretory CD56^bright^ (NK2), and adaptive (NK3) states. NK1, NK2, and NK3 gene subset scores were calculated for each sample using the Singscore package (v1.32.0) based on normalized gene expression values. The resulting scores were used as continuous variables to identify biological processes associated with each NK‐cell transcriptional state. Heatmaps representing NK1, NK2, and NK3 signature genes were generated from log2 (TPM + 1) transformed expression values and displayed as gene‐wise *z*‐scores. For each gene subset, Spearman correlation coefficients were calculated between gene expression levels and the corresponding NK signature scores across samples. Genes composing each of the NK subsets were removed prior to downstream analyses to prevent circularity and to enable the identification of biological processes associated with each transcriptional program independently of the genes originally used to define it. Genes were subsequently ranked according to their correlation coefficients and analyzed using the fgsea package (v1.32.4) with Gene Ontology (GO) Biological Process gene sets as the reference collection. Pathways with an adjusted *p*‐value < 0.05 were considered significantly enriched. Subsequent analyses focused on pathways associated with the NK1 and NK2 programs, as the NK3 score did not consistently discriminate ascites and blood samples. Enriched pathways were subsequently curated based on statistical significance, relevance to NK‐cell biology, and redundancy across related GO terms. Representative pathways were selected to summarize broader groups of functionally related biological processes. Leading‐edge genes were extracted from significantly enriched pathways using the fgsea output.

## Author Contributions

**Caroline Natânia de Souza‐Araújo:** data curation, formal analysis, investigation, writing – original draft, methodology, conceptualization. **Glenerson Baptista:** formal analysis, writing – review and editing, investigation, methodology. **Gabriela Rapozo Guimarães:** investigation, methodology, writing – review and editing, formal analysis. **Mariana Boroni:** validation, visualization, writing – review and editing, supervision. **Cláudia Rodrigues Tonetti:** data curation, investigation, methodology. **Daniela Maira Cardoso:** validation, visualization, writing – review and editing. **Adriana Yoshida:** writing – review and editing, investigation, validation, methodology. **Fernando Guimarães:** conceptualization, writing – original draft, writing – review and editing, funding acquisition, resources, supervision, project administration, validation. **Sophie Derchain:** writing – review and editing, validation, visualization. **José Andrés Yunes:** writing – review and editing, validation, visualization. **Raissa Carolina Alves da Silva:** data curation, methodology, investigation.

## Funding

This study was supported by grants from the São Paulo Research Foundation—FAPESP (2018/06959‐1). Caroline Natânia de Souza‐Araújo, Glenerson Batista, Cláudia Rodrigues Tonetti, Gabriela Rapozo Guimarães, and Raissa Carolina Alves da Silva received scholarships from the Brazilian Coordination for the Improvement of Higher Education Personnel—CAPES. Mariana Boroni is a Young Investigator Fellow funded by the Rio de Janeiro Research Foundation—FAPERJ (E‐26/201.322/2022; 272260) and the National Council for Scientific and Technological Development—CNPq (310376/2022‐0). The funding agencies did not participate in the study design, sample collection, analysis, data interpretation, or writing the manuscript.

## Ethics Statement

The study was approved by the Research Ethics Committee of the University of Campinas (April 24, 2017, #2.029.221) and was registered on the Brazilian National Health Council (CAAE: 653676179.0000.5404).

## Consent

Blood and ascites donors provided written informed consent for the use of their samples.

## Conflicts of Interest

The authors declare no conflicts of interest.

## Supporting information


**Figure S1:** NK CD56^bright^ subset accumulates in ovarian cancer ascites.

**Figure S2:** Sorting strategy for CD56+ lymphocyte isolation by flow cytometry.

**Table S1:** Characteristics of patients included in the study at the time of ascites or blood sampling. Staging classification followed FIGO Committee on Gynecologic Oncology guidelines.

**Table S2:** Comparison between transcriptomes of NK‐cell samples from ovarian cancer associated‐ascites and from blood of healthy donors identified 5427 genes differentially expressed (DEG; adjusted *p* < 0.05), being 3202 upregulated and 2225 downregulated in the ascites.

**Table S3:** NK1, NK2, and NK3 gene subsets were used to characterize distinct NK‐cell transcriptional states. These gene subsets or signatures were originally derived from single‐cell human NK‐cell transcriptomic data and represent functionally distinct NK‐cell programs corresponding to cytotoxic/terminally mature (NK1), regulatory‐secretory CD56bright (NK2), and adaptive (NK3) states (FDR, false discovery rate; log_2_FC, base‐2 logarithm of the ratio between two values).

**Table S4:** Additional enriched biological processes and corresponding leading‐edge genes associated with NK1 and NK2 scores.

## Data Availability

The data that supports the findings of this study are available in the Supporting Information of this article and from the corresponding author upon reasonable request. Additionally, the CAISM dataset is available in the University of Campinas Data Repository (REDU, https://doi.org/10.25824/redu/WRQ10C).
