# Supplementary material for: Ovarian Cancer‐Associated Ascites Accumulates NK Lymphocytes Displaying a Transcriptomic Profile Consistent With the Immature/Secretory CD56bright Subtype
Source: Immunol Cell Biol. 2026 Aug 20;104(9):896–905. doi: 10.1111/imcb.70159 (PMC13628415; doi:10.1111/imcb.70159)
Supplement: Supplementary file 1 [file IMCB-104-896-s001.pdf]

Gene subsets of NK cell subtypes			
NK signature	Gene	log2FC (Ascites vs Blood)	FDR
NK1	CD38	0.66	0.057888725
NK1	CTSD	0.53	0.180703184
NK1	FCER1G	0.34	0.561558898
NK1	IGFBP7	-0.14	0.810610778
NK1	CCL4	-0.55	0.371254051
NK1	GZMB	-0.75	0.098062978
NK1	KLRB1	-0.87	0.026744223
NK1	NKG7	-0.92	0.007253329
NK1	ADGRG1	-1.38	5.36525E-05
NK1	PRF1	-1.45	0.000850499
NK1	CD247	-1.59	3.42834E-06
NK1	CHST2	-1.66	1.27338E-05
NK1	FGFBP2	-1.76	0.000323018
NK1	CD160	-1.87	0.001325071
NK1	CLIC3	-1.88	6.0882E-08
NK1	AKR1C3	-2.38	4.05205E-06
NK1	CX3CR1	-2.43	9.25909E-06
NK1	SPON2	-2.50	1.77283E-05
NK1	LAIR2	-2.64	2.44636E-06
NK1	MYOM2	-3.56	0.017632653
NK2	FOS	5.35	2.04127E-12
NK2	AREG	2.75	0.000149479
NK2	COTL1	2.42	2.04759E-08
NK2	IFITM3	1.75	0.000219282
NK2	TPT1	1.35	5.87711E-05
NK2	GZMK	1.34	0.001130396
NK2	GPR183	0.50	0.312403309
NK2	PIK3R1	0.13	0.690487094
NK2	CD44	0.09	0.859513099
NK2	XCL1	0.05	0.927715675
NK2	CMC1	-0.08	0.853193512
NK2	ZFP36L2	-0.09	0.755467752
NK2	XCL2	-0.09	0.867312169
NK2	SELL	-0.26	0.600356624
NK2	LTB	-0.29	0.627990424
NK2	IL7R	-0.34	0.642319474
NK2	DUSP2	-0.62	0.282962787
NK2	TCF7	-0.76	0.028429634
NK2	IL2RB	-0.89	0.006884836
NK2	KLRC1	-0.97	0.012133736
NK3	S100A6	2.45	0.000106094
NK3	PPDPF	2.21	0.000730011
NK3	PTMS	1.59	0.047245527
NK3	VIM	1.36	0.004040481
NK3	LGALS1	0.90	0.126585025
NK3	ITGB1	0.69	0.005969178
NK3	DSTN	0.62	0.141360928
NK3	S100A4	0.40	0.484404955
NK3	CD3E	0.37	0.619021898
NK3	GZMH	-0.21	0.788464359
NK3	PRDM1	-0.22	0.685230872
NK3	CD3D	-0.47	0.614404045
NK3	ZBTB38	-0.49	0.037152004
NK3	IL32	-0.68	0.157953268
NK3	CD2	-0.82	0.087271468
NK3	CD3G	-0.92	0.289933863
NK3	CCL5	-1.04	0.010281236
NK3	KLRC2	-1.13	0.138437272
