# Supplementary material for: Ovarian Cancer‐Associated Ascites Accumulates NK Lymphocytes Displaying a Transcriptomic Profile Consistent With the Immature/Secretory CD56bright Subtype
Source: Immunol Cell Biol. 2026 Aug 20;104(9):896–905. doi: 10.1111/imcb.70159 (PMC13628415; doi:10.1111/imcb.70159)
Supplement: Supplementary file 2 [file IMCB-104-896-s002.pdf]

Supplementary table 1. Characteristics of patients included in the study at the time of ascites or blood sampling. Staging classification followed FIGO Committee on Gynecologic Oncology guidelines.

Sample n	Age (years)	Sample	Histotype	FIGO	Ascites cytology	CA125 U/ml
asc #10	53	ascites	HGS	III	negative	1827,0
asc #11	65	ascites	HGS	IV	positive	2844,0
asc #12	70	ascites	HGS	III	positive	783,7
asc #14	33	ascites	LGS	III	negative	786,4
asc #15	78	ascites	HGS	III	negative	4664,0
bl #21	61	blood	benign			1,1
bl #22	64	blood	benign			16,0

HGS = high-grade serous; LGS = low-grade serous
