# Supplementary material for: Ovarian Cancer‐Associated Ascites Accumulates NK Lymphocytes Displaying a Transcriptomic Profile Consistent With the Immature/Secretory CD56bright Subtype
Source: Immunol Cell Biol. 2026 Aug 20;104(9):896–905. doi: 10.1111/imcb.70159 (PMC13628415; doi:10.1111/imcb.70159)
Supplement: Supplementary file 3 [file IMCB-104-896-s003.pdf]

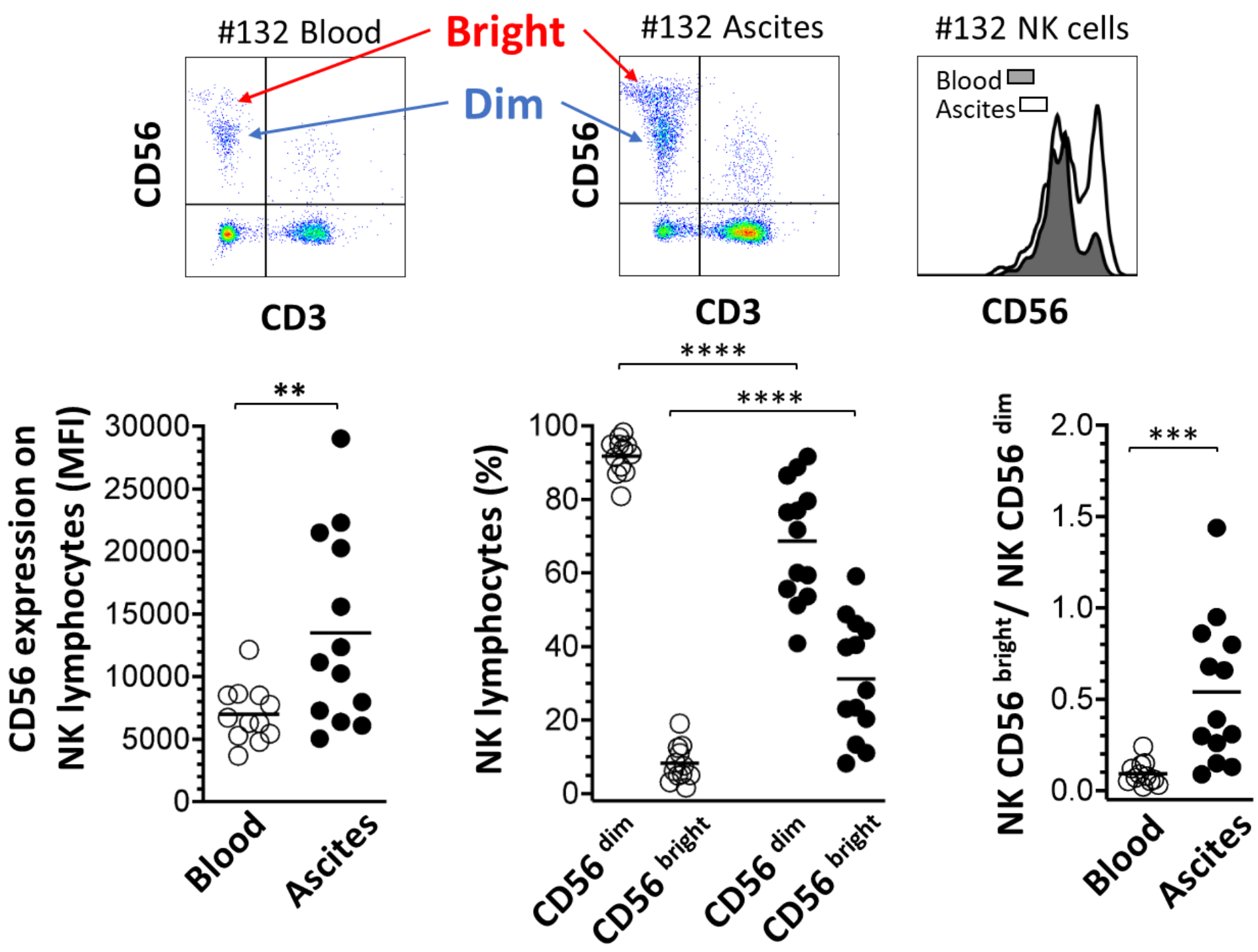

Supplementary figure 1. NK CD56^{bright} subset accumulates in ovarian cancer ascites.

The percentages of the CD56^{bright} and ^{dim} subsets within NK cells were assessed by flow cytometry immunophenotyping of the blood and ascites from 14 patients with serous advanced EOC. Analysis showed accumulation of the CD56^{bright} subset in ascites (black circles), resulting in altered subset rates of NK cells compared to blood of patients (white circles). Unpaired two-tailed Student's t-test, n=14 (*p < 0.05, **p < 0.01, *** p < 0.001 and **** p < 0.0001).

Tonetti, C.R.; de Souza-Araújo, C.N.; Yoshida, A.; da Silva, R.F.; Alves, P.C.M.; Mazzola, T.N.; Derchain, S.; Fernandes, L.G.R.; Guimarães, F. Ovarian Cancer-Associated Ascites Have High Proportions of Cytokine-Responsive CD56bright NK Cells. Cells 2021, 10, 1702. doi: 10.3390/ cells10071702
