# Supplementary material for: Ovarian Cancer‐Associated Ascites Accumulates NK Lymphocytes Displaying a Transcriptomic Profile Consistent With the Immature/Secretory CD56bright Subtype
Source: Immunol Cell Biol. 2026 Aug 20;104(9):896–905. doi: 10.1111/imcb.70159 (PMC13628415; doi:10.1111/imcb.70159)
Supplement: Supplementary file 4 [file IMCB-104-896-s004.pdf]

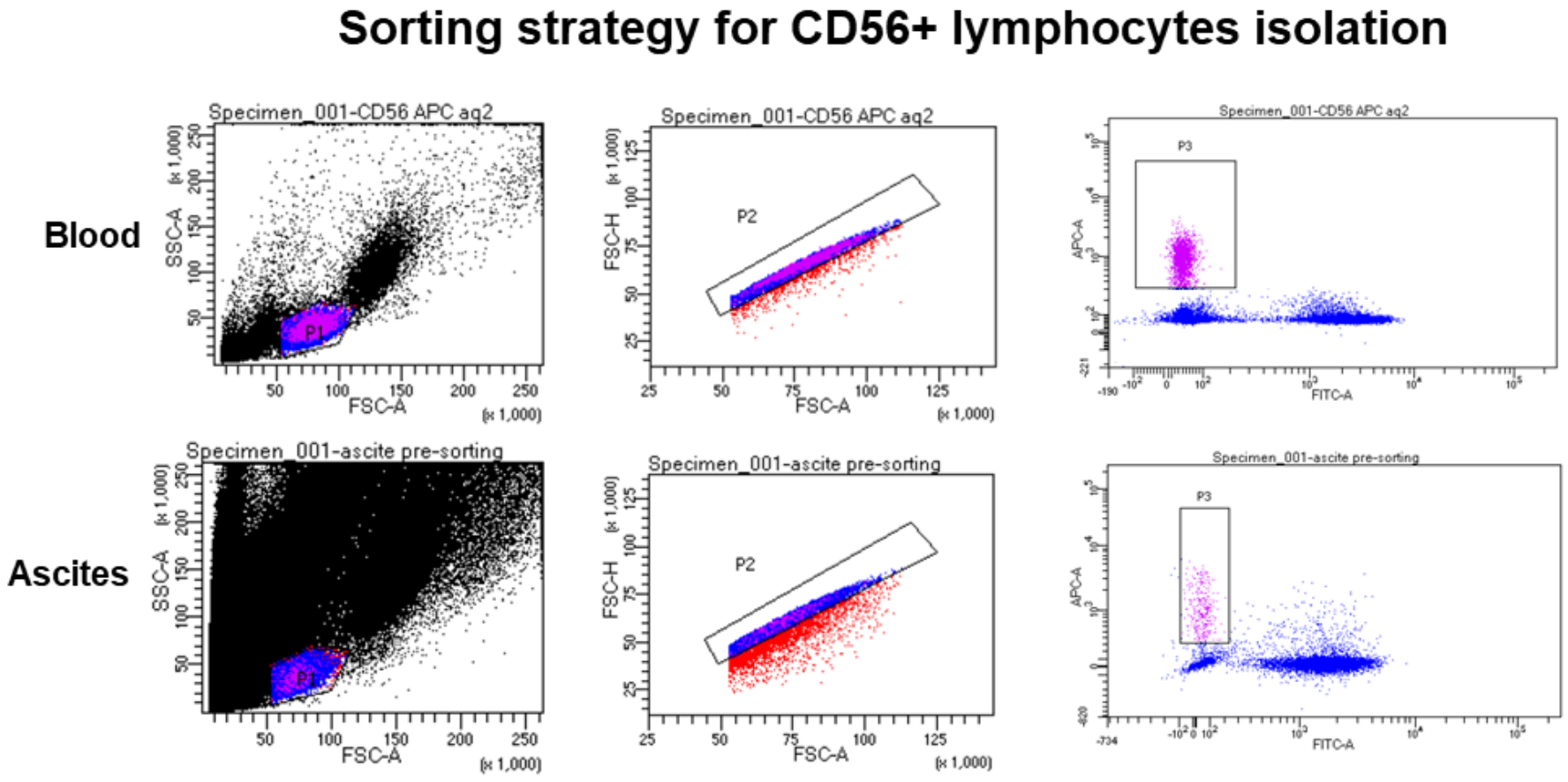

Supplementary figure 2. Sorting strategy for CD56+ lymphocyte isolation by flow cytometry.

NK cells were isolated from blood and ascites associated with ovarian cancer by flow cytometry, using a gate strategy (P1, P2 and P3) based on morphological cell parameters (FSC-A and SSC-A and FSC-H) to identify singlets in lymphocyte population, as well as labeling of specific cell surface molecules (CD3 and CD56) with monoclonal antibodies (MAb) conjugated with fluorophores.
