# Supplementary material for: Ovarian Cancer‐Associated Ascites Accumulates NK Lymphocytes Displaying a Transcriptomic Profile Consistent With the Immature/Secretory CD56bright Subtype
Source: Immunol Cell Biol. 2026 Aug 20;104(9):896–905. doi: 10.1111/imcb.70159 (PMC13628415; doi:10.1111/imcb.70159)
Supplement: Supplementary file 5 [file IMCB-104-896-s005.pdf]

gene name	baseMean	log2FoldChange	lfcSE	stat	pvalue	padj
[illegible]	[illegible]	[illegible]	[illegible]	[illegible]	[illegible]	[illegible]
ABHD17B	255,5147107	-0,4662219758	0,1493302707	-3,1221531543	0,0017953052	0,0070166118
ABHD4	513,6654427	-1,4738475146	0,2217837631	-6,6454256793	0,0000000000	0,0000000015
ABI3	683,152686	-1,4751775087	0,3943266336	-3,7410039874	0,0001832866	0,0010834597
ABI3BP	101,5081808	5,3342542075	0,8105803441	6,5807840595	0,0000000000	0,0000000022
ABL2	166,2278172	0,4578953691	0,1927252444	2,3748582238	0,0175556919	0,0447113743
ABLIM3	37,30801404	4,3840468868	1,0855260780	4,0386378400	0,0000537525	0,0003952175
ABT1	547,2932847	-0,7149171723	0,1611363090	-4,4367230235	0,0000091339	0,0000883838
ABTB1	1002,678564	-1,0247548385	0,2172265586	-4,7172496683	0,0000023905	0,0000283495
ABTB2	26,3402569	2,4862310521	0,5749958842	4,3239110410	0,0000153287	0,0001368768
ACAA1	759,7824649	-0,4057913786	0,0961431777	-4,2206986306	0,0000243546	0,0002012360
ACACA	251,5956674	1,5306715827	0,4229661963	3,6188881434	0,0002958601	0,0016122576
ACAD8	427,3376147	-0,8074113225	0,2052312346	-3,9341541956	0,0000834902	0,0005638624
ACAD9	325,220369	0,4160135765	0,1386471757	3,0005196601	0,0026951935	0,0097808857
ACADS	203,3589896	-0,8194580160	0,1887996517	-4,3403152940	0,0000142278	0,0001286143
ACADVL	4808,165299	-0,6705673901	0,2575865197	-2,6032705085	0,0092339064	0,0266256171
ACAT1	222,5961823	0,8591175691	0,2592116680	3,3143475971	0,0009185724	0,0040511089
ACBD3	375,489742	0,5790400910	0,2054062394	2,8190004978	0,0048172443	0,0156318804
ACBD4	139,5414899	-0,6491112439	0,2124492193	-3,0553712833	0,0022478206	0,0084418711
ACBD6	271,592214	0,4584772803	0,1620449654	2,8293213503	0,0046646835	0,0152421755
ACD	312,1685236	-1,1926973042	0,2490735539	-4,7885344930	0,0000016800	0,0000208417
ACE	35,06681484	3,1918061858	0,6064002880	5,2635301281	0,0000001413	0,0000024464
ACER3	188,4160471	0,4937811058	0,2006346400	2,4610959794	0,0138513306	0,0370432961
ACOT7	135,45366	0,9923774689	0,4065836378	2,4407707948	0,0146559534	0,0387638303
ACOT8	279,3529187	-0,4444932519	0,1789798810	-2,4834816596	0,0130109334	0,0352002765
ACOT9	366,6892159	0,5741822174	0,1631144552	3,5201185377	0,0004313540	0,0021860032
ACOX1	270,3582777	0,4982825823	0,1703903418	2,9243078219	0,0034522317	0,0120007849
ACP2	384,558767	0,9000010025	0,3072109731	2,9295861182	0,0033941375	0,0118263242
ACP3	55,31024338	3,7080554853	0,7044957277	5,2634179876	0,0000001414	0,0000024464
ACP5	163,5218774	0,9754460276	0,4179957215	2,3336368229	0,0196152708	0,0489926787
ACSF2	115,7765771	1,4376809994	0,3907266879	3,6795355055	0,0002336866	0,0013271824
ACSL1	757,4830276	1,5666571108	0,3691380346	4,2440841095	0,0000219488	0,0001856888
ACSL3	423,1396524	0,8465116418	0,2048504726	4,1323392187	0,0000359090	0,0002797078
ACSL4	297,0462727	0,6870695631	0,2532226827	2,7133017022	0,0066616425	0,0203827685
ACSL6	62,30582233	3,2644701585	0,4860269764	6,7166439666	0,0000000000	0,0000000010
ACSS1	1000,666642	-1,0106915788	0,2109937247	-4,7901499449	0,0000016666	0,0000207128
ACSS2	143,8194134	1,6473045804	0,4715071408	3,4937001756	0,0004763757	0,0023721853
ACTG1	15256,12128	0,8459571256	0,2609623165	3,2416821173	0,0011882607	0,0049748021
ACTL10	37,20569867	-1,0793317293	0,3348119075	-3,2236957678	0,0012654777	0,0052375006
ACTN1	560,6847927	1,7043854038	0,3760038719	4,5329892253	0,0000058181	0,0000605661
ACTR1A	821,2249646	-0,5341939804	0,1149713467	-4,6463227220	0,0000033790	0,0000381560
ACTR6	190,610221	-0,4065372517	0,1563404031	-2,6003339110	0,0093133090	0,0269086268
ACTR8	253,4730281	-0,4875251741	0,1853025851	-2,6310219784	0,0085128530	0,0249258219
ACVR1	109,8263417	1,3144637305	0,3407998304	3,8569964334	0,0001147888	0,0007353916
ACVR1B	98,56054587	1,7885494211	0,3591472900	4,9799886308	0,0000006359	0,0000089905
ACVR2A	105,2943383	1,3132687831	0,3669165921	3,5792024987	0,0003446443	0,0018270630
ACY1	171,5103076	0,7282358812	0,2499191915	2,9138857921	0,0035696047	0,0122165546
ADA	743,8565172	-1,2287586727	0,2938960452	-4,1809329957	0,0000290315	0,0002340987
ADAM15	441,0098397	1,0551847717	0,3650362108	2,8906296186	0,0038447095	0,0131178173
ADAM19	52,48029435	2,0457593113	0,5196130843	3,9371012257	0,0000824718	0,0005578245
ADAM8	3617,292893	-1,5124077378	0,2964409648	-5,1018850879	0,0000003363	0,0000051968
ADAM9	1015,038343	3,8703626877	0,5362533461	7,2174145226	0,0000000000	0,0000000000
ADAMTS1	638,4223803	2,1829949694	0,6080584021	3,5901074006	0,0003305417	0,0017641125
ADAMTS10	679,0057541	-1,1086229359	0,2396835829	-4,6253603287	0,0000037395	0,0000415292
ADAMTS14	29,70908574	2,5264399902	0,7325637255	3,4487662538	0,0005631581	0,0027207855
ADAMTS15	29,99483908	7,1263051221	1,1655544327	6,1139458412	0,0000000010	0,0000000319
ADAMTS17	56,41325613	2,9100778242	0,4945642133	5,8841255509	0,0000000040	0,0000001104
ADAMTSL4	32,50020458	2,4063182542	0,5080117915	4,7367370097	0,0000021719	0,0000260547
ADAMTSL5	12,58684569	1,9306694041	0,6927614313	2,7869181464	0,0053211918	0,0169723221
ADAP1	555,1585401	-1,1686845317	0,1750380042	-6,6767092122	0,0000000000	0,0000000013
ADAP2	374,6137007	3,2087810296	0,6996173672	4,5864799533	0,0000045078	0,0000488216
ADARB2	56,95551617	-1,9732011087	0,8109845868	-2,4331165379	0,0149653481	0,0394503692
ADCK5	155,8634888	-0,8705110434	0,2089533083	-4,1660553294	0,0000309916	0,0002463947
ADCY6	59,95757258	2,7484592409	0,7683003897	3,5773228558	0,0003471300	0,0018368714
ADCY7	2873,066791	-0,6898601430	0,2072613762	-3,3284548988	0,0008732914	0,0038832186
ADD1	2318,512349	-0,3277467036	0,1405157707	-2,3307961963	0,0197641088	0,0492728958
ADGRA3	93,0653083	1,2681012340	0,4928299688	2,5731008953	0,0100791820	0,0285722497
ADGRD1	84,16191271	3,4843828814	0,6734750389	5,1737520772	0,0000002294	0,0000037208
ADGRE5	4068,115016	-0,6393724878	0,2545145522	-2,5121254652	0,0120006415	0,0329314239
ADGRF1	124,114469	4,2280253475	1,2459932054	3,3932872741	0,0006905665	0,0032073932
ADGRG1	2582,046485	-1,3816412171	0,3027249859	-4,5640144728	0,0000050185	0,0000536525
ADGRG5	749,7487846	-1,4800568551	0,3275786087	-4,5182781787	0,0000062782	0,0000645569
ADGRG6	41,68932244	6,5690722654	1,2165764712	5,3996377714	0,0000000668	0,0000012713
ADGRL1-AS1	48,3059964	-0,8909687573	0,2392467746	-3,7240575505	0,0001960462	0,0011477955
ADGRL2	122,8582163	4,6443388051	0,7124932437	6,5184320642	0,0000000001	0,0000000032
ADHFE1	306,6433677	-1,0928042132	0,2258576284	-4,8363240520	0,0000013226	0,0000170852
ADI1	403,8652027	0,4925890144	0,1440417753	3,4197649485	0,0006267527	0,0029600318
ADK	157,8560898	0,6423855975	0,1568254306	4,0960214371	0,0000420493	0,0003212545
[illegible]	[illegible]	[illegible]	[illegible]	[illegible]	[illegible]	[illegible]
AIP	572,811483	-0,6660036084	0,1368985831	-4,8649056837	0,0000011451	0,0000151556
AJM1	173,4582111	-0,8991615806	0,3460621373	-2,5982663044	0,0093695798	0,0269015666
AJUBA	62,21284154	2,8681050849	0,7099265063	4,0400022475	0,0000534507	0,0003933550
AK2	1092,903564	0,4101193817	0,1325970248	3,0929757473	0,0019816033	0,0076118662
AK4	179,9469189	4,2560800791	0,6740785436	6,3139230875	0,0000000003	0,0000000103
AK9	103,0588756	-0,7872968519	0,2464791861	-3,1941717443	0,0014023266	0,0057076857
AKAP1	177,3619966	1,4791508539	0,4150778102	3,5635502976	0,0003658725	0,0019102578
AKAP12	145,5890857	5,6782402915	0,8846183869	6,4188730566	0,0000000001	0,0000000056
AKAP13	3665,652645	-0,6561519235	0,1521285334	-4,3131417147	0,0000160951	0,0001427140
AKAP17A	1440,070726	-0,5645639723	0,1686991330	-3,3465730507	0,0008181712	0,0036856038
AKAP6	9,334211662	1,7195471516	0,4579415617	3,6747049042	0,0002381245	0,0013494036
AKAP7	75,61034745	0,4918723012	0,2012245823	2,4443946936	0,0145095432	0,0384521465
AKAP8L	1262,951567	-0,5918457430	0,1471687299	-4,0215454968	0,0000578175	0,0004188504
AKAP9	961,2562342	-0,4711531700	0,1993851403	-2,3630305113	0,0181261772	0,0458692132
AKNA	2090,67422	-0,6269879377	0,2411836485	-2,5996287123	0,0093324673	0,0269329346
AKR1A1	631,8167648	0,5331518889	0,2006689703	2,6568726000	0,0078869242	0,0234707052
AKR1B1	745,6262688	-0,6588057262	0,2390715878	-2,7556939027	0,0058569569	0,0183185196
AKR1C3	344,0344221	-2,3754452471	0,4607940851	-5,1551122810	0,0000002535	0,0000040521
AKT1S1	133,4573861	0,7017214491	0,2641713559	2,6563116457	0,0079000569	0,0235045902
AKT3	276,0134321	-0,8594858912	0,2542572106	-3,3764465883	0,0007342863	0,0033722059
ALCAM	311,6563191	4,6101608260	0,8964304584	5,1427980640	0,0000002707	0,0000042913
ALDH16A1	317,1267915	-0,5313188685	0,2034182964	-2,6119393680	0,0090030220	0,0261108975
ALDH18A1	430,0584865	0,5389121360	0,1675936318	3,2155883871	0,0013017740	0,0053649851
ALDH1A1	53,91426895	3,0847765562	0,7669223684	4,0222800679	0,0000576375	0,0004181584
ALDH1A2	69,16394358	3,0517598096	0,9211411631	3,3130316305	0,0009229054	0,0040618427
ALDH1A3	270,9691497	9,9306931259	1,3859896112	7,1650559615	0,0000000000	0,0000000001
ALDH2	328,911832	2,2293524770	0,4840887063	4,6052466058	0,0000041198	0,0000452301
ALDH3A2	424,1323951	0,3464437715	0,1492012441	2,3219882739	0,0202335670	0,0502201387
ALDH3B1	215,656368	3,2348138599	0,4524622731	7,1493564045	0,0000000000	0,0000000001
ALDH7A1	130,6269188	3,1855018857	0,5259346127	6,0568401635	0,0000000014	0,0000000433
ALDOA	7569,124929	1,0485044631	0,2272624632	4,6136279986	0,0000039570	0,0000437644
ALDOC	410,7666207	0,7662513836	0,2352637008	3,2556056053	0,0011315081	0,0047802575
ALG1	355,6639791	-0,3326825782	0,1262840779	-2,6343984441	0,0084286514	0,0247436535
ALG12	362,354282	-0,4907579092	0,2061149283	-2,3809915824	0,0172661065	0,0441325092
ALG13	349,7104334	0,8626811971	0,2012874622	4,2858168493	0,0000182069	0,0001585154
ALG14	67,73740098	0,6261515163	0,2691647327	2,3262762174	0,0200038199	0,0497508160
ALG5	310,1751067	0,7692593834	0,1273069200	6,0425574942	0,0000000015	0,0000000468
ALG8	343,2298456	0,8536881511	0,1826944002	4,6727658328	0,0000029717	0,0000342752
ALKBH4	100,4544974	-0,7045603259	0,1944715112	-3,6234530036	0,0002906948	0,0015879129
ALKBH6	302,8031758	-0,9251009250	0,1838389056	-5,0321281128	0,0000004851	0,0000071255
ALKBH7	118,5877613	0,4515242340	0,1774410939	2,5446429807	0,0109389517	0,0305220337
ALOX12-AS1	100,0043875	-1,2518417995	0,2692709369	-4,6490045084	0,0000033354	0,0000378219
ALOX5	306,8347513	4,8830029551	0,7616248260	6,4112969910	0,0000000001	0,0000000058
ALPK1	47,10666898	2,6133886781	0,3305605004	7,9054541184	0,0000000000	0,0000000000
ALS2CL	213,0387158	3,8519095107	0,9565545511	4,0268581717	0,0000565271	0,0004117180
AMD1	786,0031265	1,3007953303	0,2877630264	4,5203899252	0,0000061732	0,0000635978
AMIGO1	110,9906659	-0,8706003539	0,3679271074	-2,3662305287	0,0179702509	0,0455588729
AMOTL2	246,4634363	10,5846175997	1,2374737139	8,5534080285	0,0000000000	0,0000000000
AMPD3	172,1256801	1,6630506510	0,3431281425	4,8467334642	0,0000012551	0,0000164091
AMZ1	80,12755841	-2,5958028150	0,6660720894	-3,8971799843	0,0000973193	0,0006408599
ANAPC1	426,383072	0,4712578024	0,1351916350	3,4858503079	0,0004905754	0,0024267348
ANAPC13	296,1382369	0,4110266758	0,1262300646	3,2561702258	0,0011292603	0,0047738201
ANAPC15	177,0120176	0,4173237287	0,1582103628	2,6377774582	0,0083451326	0,0245466532
ANAPC5	1649,267779	-0,3046627829	0,1305032556	-2,3329882327	0,0196487629	0,0490672262
ANAPC7	411,7064979	-0,4062067034	0,1378926834	-2,9458176702	0,0032210221	0,0113091559
ANGPTL4	131,6204376	6,6699959804	1,1118682770	5,9989308855	0,0000000020	0,0000000592
ANGPTL6	31,01132955	-1,6882039965	0,5905086889	-2,8588977599	0,0042511577	0,0141460033
ANK3	71,07069707	1,2840859454	0,3658279712	3,5100813676	0,0004479696	0,0022540825
ANKEF1	49,38964891	0,9803708174	0,3794020031	2,5839895663	0,0097654749	0,0278156479
ANKMY1	314,9359662	-0,5352462914	0,1970352354	-2,7159928539	0,0066077344	0,0202504794
ANKRD12	1003,869454	-0,5570538510	0,2031224002	-2,7424540599	0,0060981980	0,0189521460
ANKRD13D	842,8338446	-0,5913976625	0,1315689017	-4,4949312474	0,0000069592	0,0000705889
ANKRD27	485,3017515	-0,5292772358	0,2197342124	-2,4087156478	0,0160087655	0,0416450374
ANKRD33B	17,36931153	1,9677864776	0,6344225800	3,1489688802	0,0016383923	0,0064956585
ANKRD34A	14,18590415	-1,8402434352	0,4479471609	-4,1081707754	0,0000398805	0,0003070891
ANKRD37	111,7539153	2,2056757378	0,6770401572	3,2578211418	0,0011227114	0,0047476268
ANKRD39	117,729561	-0,4776991896	0,1601017207	-2,9837228957	0,0028476454	0,0102460637
ANKRD49	294,0725844	-0,4520687564	0,1634904228	-2,7651384918	0,0056903867	0,0179086207
ANKRD50	57,22848116	1,5908238075	0,3751938482	4,2400050407	0,0000223515	0,0001886212
ANKRD52	543,7093557	-0,3031622152	0,1116338740	-2,7156830113	0,0066139210	0,0202648252
ANKRD54	243,7835755	-0,4496015669	0,1716272340	-2,6196384721	0,0088023028	0,0256337075
ANKRD6	70,37972595	1,0386432133	0,2890122183	3,5937692161	0,0003259286	0,0017436400
ANKRD9	82,53438592	0,8677519558	0,3495857424	2,4822292528	0,0130563246	0,0352816962
ANKS6	126,8140281	1,5836882641	0,4262656333	3,7152613782	0,0002029838	0,0011827138
ANKZF1	1057,857803	-0,7454830078	0,2019709852	-3,6910401117	0,0002233389	0,0012813903
ANLN	108,7057794	5,0044580808	0,8230048507	6,0807151705	0,0000000012	0,0000000381
ANO9	422,5681006	1,9412155599	0,3308299855	5,8677134635	0,0000000044	0,0000001209
ANP32B	471,6595786	0,7309046584	0,2438715851	2,9970882343	0,0027257181	0,0098756838
ANPEP	890,9167682	4,8158264584	0,9062801840	5,3138384184	0,0000001073	0,0000019267
ANTXR2	196,6519476	1,5724049704	0,4153170379	3,7860353098	0,0001530699	0,0009231165
ANXA2	6617,952754	2,5665312657	0,5726621769	4,4817544608	0,0000074032	0,0000745302
ANXA2R	195,2027742	-0,7965626531	0,3431876273	-2,3210704285	0,0202830431	0,0503243797
ANXA2R-AS1	182,0000448	-0,7821399925	0,3314132948	-2,3600139306	0,0182742488	0,0462004863
ANXA3	183,8824524	6,2658870379	1,0287324535	6,0908810805	0,0000000011	0,0000000360
ANXA5	2183,482938	1,9235158110	0,3323036640	5,7884282334	0,0000000071	0,0000001832
ANXA8	116,8846567	8,7390510219	1,1261899152	7,7598377535	0,0000000000	0,0000000000
AOPEP	101,4061385	1,4436530166	0,4124529594	3,5001880852	0,0004649296	0,0023220605
AP1B1	600,1516148	0,8781995287	0,2815471511	3,1191917105	0,0018134793	0,0070772487
AP1G2	2073,778751	-0,7054922014	0,2029747232	-3,4757638301	0,0005094007	0,0025014713
AP1G2-AS1	213,1677839	-0,6417771550	0,2142940889	-2,9948430134	0,0027458612	0,0099352936
AP1M1	722,8836829	-0,5727057009	0,2148999698	-2,6649873475	0,0076991219	0,0230494176
AP1S1	40,5407676	1,9217893733	0,6495890017	2,9584696910	0,0030917064	0,0109626893
AP1S2	490,3853899	0,9783648684	0,4046318263	2,4179137792	0,0156097751	0,0407975869
AP1S3	66,224516	1,2572076467	0,4402421875	2,8557182440	0,0042939600	0,0142602275
AP2S1	328,7962751	0,5710538281	0,1591972690	3,5870820682	0,0003343978	0,0017811613
AP4B1	426,0661386	-0,4330631377	0,1305711688	-3,3166827064	0,0009109300	0,0040266299
AP4B1-AS1	30,39862281	-0,9239659554	0,2719019146	-3,3981222857	0,0006785008	0,0031588835
AP4M1	110,7378835	-0,6907942706	0,2426147729	-2,8495968387	0,0043774678	0,0144938594
AP5B1	387,1494776	-0,7862751833	0,1791651352	-4,3885524626	0,0000114109	0,0001060941
AP5Z1	626,8545269	-0,5913152605	0,1647932402	-3,5882252190	0,0003329366	0,0017740804
APBA3	335,9998632	-1,2084326859	0,2451869968	-4,9286185418	0,0000008281	0,0000113741
APEX1	618,7063606	0,7858365669	0,1959982089	4,0094068777	0,0000608715	0,0004374625
APEX2	300,4403919	-0,6832360135	0,1621811445	-4,2127586146	0,0000252271	0,0002074259
APH1A	834,9873696	0,6215348994	0,2188184392	2,8404137316	0,0045055057	0,0148093058
APH1B	117,3504339	0,8725884640	0,2251437686	3,8756964933	0,0001063210	0,0006903429
APLP1	60,47193498	5,3355607269	0,8785914307	6,0728576905	0,0000000013	0,0000000395
APLP2	3063,78653	1,4260840977	0,3695213123	3,8592742829	0,0001137242	0,0007303093
APMAP	4601,974174	-0,9454707486	0,2316479963	-4,0814976336	0,0000447654	0,0003377268
APOA1	52,86822844	5,0767970098	0,9714635160	5,2259065802	0,0000001733	0,0000029228
APOBEC3A	65,53691505	1,9649738673	0,6151824816	3,1939691492	0,0014032111	0,0057099661
APOBEC3B	56,99880445	1,7280783048	0,4651681311	3,7149542052	0,0002032405	0,0011827138
APOBEC3C	1911,684726	-1,4444886996	0,3437834455	-4,2017401211	0,0000264871	0,0002158097
APOBEC3D	322,3160616	-1,3313957154	0,3795703298	-3,5076390620	0,0004521020	0,0022714792
APOBEC3F	605,6632128	-1,7216316024	0,4067272611	-4,2328886215	0,0000230708	0,0001934783
APOBEC3G	4284,854672	-1,7189607648	0,4619538489	-3,7210660091	0,0001983836	0,0011604706
APOBR	587,3454851	-0,8752967326	0,2705882250	-3,2344182689	0,0012189080	0,0050778403
APOC1	158,579628	7,5765268289	0,8502762639	8,9106672154	0,0000000000	0,0000000000
APOC2	82,38173849	6,3074072519	0,8495956638	7,4240106445	0,0000000000	0,0000000000
APOE	1647,514803	7,1278521204	0,6681715278	10,6676995708	0,0000000000	0,0000000000
APOL3	613,5133881	-1,1539786871	0,2802471373	-4,1177180195	0,0000382642	0,0002958281
APP	517,526645	5,6355219889	0,6934061003	8,1273037480	0,0000000000	0,0000000000
[illegible]	[illegible]	[illegible]	[illegible]	[illegible]	[illegible]	[illegible]
AQP9	261,4338152	2,8311444218	0,9418161770	3,0060477734	0,0026466741	0,0096496340
ARAF	856,7222777	-0,6035996758	0,1389239415	-4,3448211364	0,0000139389	0,0001265124
ARAP1	1635,885775	-0,6596459692	0,2275336494	-2,8991139152	0,0037421891	0,0128592966
ARAP1-AS2	18,19075216	-1,2098983518	0,3916254468	-3,0894298661	0,0020054105	0,0076888983
ARAP3	97,00429038	-1,2562982696	0,3267622180	-3,8446895262	0,0001207352	0,0007663639
AREG	324,5193966	2,7532044138	0,6401517541	4,3008620946	0,0000170135	0,0001494789
ARF4	1283,710183	1,0895607493	0,2715789237	4,0119488443	0,0000602196	0,0004334520
ARFGAP2	1145,636852	-0,5052484687	0,1790531987	-2,8217840972	0,0047757317	0,0155156525
ARFGEF3	54,07218658	4,7677055898	0,9135178673	5,2190611269	0,0000001798	0,0000030181
ARFRP1	391,3218722	-0,3504030354	0,1346594371	-2,6021424381	0,0092643367	0,0268962045
ARHGAP11A	70,27644368	1,2654165877	0,3599721145	3,5153183721	0,0004392271	0,0022200513
ARHGAP12	323,5256605	1,4939412056	0,3194344588	4,6765186542	0,0000029179	0,0000337226
ARHGAP21	233,2513773	1,2649886045	0,2881890468	4,3894402605	0,0000113643	0,0001060036
ARHGAP22	35,39483933	1,6170710732	0,4903959125	3,2974807332	0,0009755637	0,0042426776
ARHGAP23	166,3252094	7,9696829150	1,2211023191	6,5266299066	0,0000000001	0,0000000030
ARHGAP24	21,62862898	4,0329879012	0,6834444371	5,9009740696	0,0000000036	0,0000001011
ARHGAP25	1778,600757	-1,4029705417	0,3273513549	-4,2858247594	0,0000182062	0,0001585154
ARHGAP27	1120,923114	-0,9416490996	0,1920577396	-4,9029479440	0,0000009441	0,0000127585
ARHGAP28	24,66298396	2,5814090476	0,8103869305	3,1854031088	0,0014455250	0,0058551910
ARHGAP29	162,0168829	4,1663379594	0,7424733672	5,6114308516	0,0000000201	0,0000004496
ARHGAP32	63,95267204	1,8275874543	0,5806581451	3,1474358266	0,0016470820	0,0065224649
ARHGAP42	29,19978921	1,7877516741	0,7063041770	2,5311356385	0,0113693873	0,0314686311
ARHGAP44	15,17915869	1,6609141005	0,5763307050	2,8818768221	0,0039531424	0,0133960968
ARHGAP5	282,361828	0,9204100116	0,3461137886	2,6592699917	0,0078310180	0,0233558721
ARHGAP8	49,03461078	1,7449850623	0,7118853589	2,4512222937	0,0142371999	0,0379020392
ARHGAP9	2750,933548	-0,7753535308	0,2803263037	-2,7658887619	0,0056767995	0,0178819535
ARHGEF1	5991,798148	-0,9546324012	0,2206582014	-4,3716136321	0,0000123332	0,0001133910
ARHGEF10L	140,2758194	3,4975338211	0,8201143858	4,2646902455	0,0000200180	0,0001719476
ARHGEF11	112,1877874	2,3332229517	0,4401716336	5,3007117534	0,0000001154	0,0000020683
ARHGEF17	46,93012619	6,7818617956	1,1134250279	6,0909909742	0,0000000011	0,0000000360
ARHGEF2	470,0064123	-0,6135636790	0,2289463063	-2,6799146910	0,0074503379	0,0223992737
ARHGEF3	2181,179775	-1,1526022465	0,1535598447	-7,5058782347	0,0000000000	0,0000000000
ARHGEF35	33,12920979	5,1063467688	1,0134199579	5,0387272808	0,0000004686	0,0000069470
ARHGEF40	38,43953122	1,5171664014	0,4932256059	3,0760089973	0,0020976151	0,0079700643
ARHGEF5	59,44838608	3,2691539582	0,7404331184	4,4151914291	0,0000100921	0,0000962034
ARHGEF7	535,213238	-0,5719772146	0,1280880080	-4,4655316789	0,0000079881	0,0000793508
ARID1A	1260,131475	-0,5919211429	0,1167180306	-5,0713336126	0,0000003950	0,0000060014
ARID2	500,8198911	-0,4789251287	0,1959508163	-2,4441088729	0,0145210437	0,0384750490
ARID4A	471,2888711	-0,4727156285	0,1952982374	-2,4204795331	0,0155000518	0,0405817346
ARID4B	948,3062822	-0,5960347542	0,1878743110	-3,1757122674	0,0014946912	0,0060145124
ARIH2	1483,752497	-0,5543835939	0,1475167741	-3,7581054587	0,0001712047	0,0010219138
ARL1	418,2026236	0,9557767739	0,2853233188	3,3498825256	0,0008086919	0,0036477859
ARL15	102,0077526	-0,5429852627	0,1377568551	-3,9416213604	0,0000809327	0,0005493463
ARL2	221,1370141	-0,4439085912	0,1252064745	-3,5454124305	0,0003919992	0,0020210199
ARL4A	255,1457695	1,9769269946	0,2888553657	6,8440036173	0,0000000000	0,0000000005
ARL4C	3827,523809	-0,8110095663	0,3154661739	-2,5708289298	0,0101455429	0,0287311188
ARL4D	58,58120837	1,3189983294	0,4993398804	2,6414840496	0,0082543694	0,0243328541
ARL5B	303,5196147	1,0652067052	0,3907235711	2,7262412201	0,0064060163	0,0197537181
ARL6IP5	1982,416806	-0,7957214395	0,1750964825	-4,5444741570	0,0000055073	0,0000580029
ARMC10	271,3888497	0,4560461792	0,1741429641	2,6188023584	0,0088238796	0,0256854281
ARMC5	268,1222918	0,5167723691	0,1597672974	3,2345315808	0,0012184245	0,0050773972
ARMC9	72,53512679	1,2363137377	0,2170768003	5,6952826643	0,0000000123	0,0000002945
ARMCX1	19,18036601	1,9572685250	0,5153455969	3,7979727521	0,0001458844	0,0008862133
ARMCX2	48,23672478	1,9139894089	0,4781634019	4,0027826080	0,0000625989	0,0004469827
ARNT2	26,98637791	2,8652048218	0,5692176837	5,0335826424	0,0000004814	0,0000070892
ARPC1A	432,4059878	0,6917261890	0,2506304764	2,7599444365	0,0057811193	0,0181215337
ARPIN	48,0865072	2,5171027985	0,4869149684	5,1694915163	0,0000002347	0,0000037929
ARRDC1	651,9205088	-0,4879902040	0,1919391059	-2,5424219786	0,0110087176	0,0306721877
ARRDC1-AS1	149,8511094	-1,0878176876	0,1981989723	-5,4885132599	0,0000000405	0,0000008279
ARRDC2	1009,430446	-0,8475268670	0,2505257251	-3,3829933701	0,0007170036	0,0033054950
ARRDC3	1160,581021	0,9344781535	0,1697513133	5,5049823524	0,0000000369	0,0000007657
ARRDC4	230,3626611	2,6920861905	0,5038489974	5,3430416740	0,0000000914	0,0000016876
ASAH1	1557,82259	1,1472283497	0,2604994843	4,4039561493	0,0000106294	0,0001001907
ASAP1	400,4549439	1,0821745177	0,1741715887	6,2132666186	0,0000000005	0,0000000182
ASAP2	93,18058799	6,9520640390	1,3586626495	5,1168434206	0,0000003107	0,0000048398
ASAP3	27,61587199	3,5245569061	0,7094758868	4,9678318485	0,0000006771	0,0000094929
ASB3	248,6189118	-0,4308234821	0,1818387065	-2,3692616959	0,0178236370	0,0452633682
ASB6	422,2734533	-0,5923881009	0,1340895614	-4,4178539686	0,0000099686	0,0000953610
ASB8	537,2284843	-0,6982432108	0,2390689294	-2,9206777928	0,0034927086	0,0121164580
ASCC3	445,4257654	0,5719224969	0,1436237389	3,9820889032	0,0000683122	0,0004793865
ASCL2	64,86663691	-2,4215722917	0,5321724856	-4,5503522964	0,0000053556	0,0000566719
ASF1A	218,7000894	-0,7178304458	0,2205357210	-3,2549395739	0,0011341649	0,0047871243
ASMTL	305,8394317	-0,7800934279	0,1856187128	-4,2026658635	0,0000263790	0,0002153190
ASPH	394,5556742	2,1513588201	0,3468376961	6,2027790346	0,0000000006	0,0000000193
ASPHD2	52,09119091	-0,7250005165	0,2754761565	-2,6318085956	0,0084931696	0,0248818888
ASPM	37,19632921	1,4052727586	0,5678894017	2,4745525935	0,0133402804	0,0358976757
ASRGL1	65,75516091	1,4116943238	0,4341765159	3,2514294811	0,0011482626	0,0048374379
ASS1	286,7340621	8,7810720614	1,1128807203	7,8903882264	0,0000000000	0,0000000000
ASTE1	216,7150114	-0,8278067128	0,3202312404	-2,5850279682	0,0097371099	0,0277555059
ATAD2	242,6199579	0,9746151170	0,2898011878	3,3630473929	0,0007708714	0,0035068122
ATF3	529,8442018	4,4783334158	0,7596893117	5,8949528275	0,0000000037	0,0000001040
ATF4	2298,115816	0,9200474122	0,1466035186	6,2757525946	0,0000000003	0,0000000127
ATF5	84,02103938	2,6306589371	0,5583452930	4,7115265441	0,0000024587	0,0000290552
ATF6	546,3581371	0,4892678557	0,1434358642	3,4110566319	0,0006471165	0,0030338516
ATF7IP	1498,406106	-0,3794476513	0,1389086982	-2,7244344925	0,0064411707	0,0198428362
ATG13	979,1451765	-0,5158066354	0,1926360074	-2,6776231636	0,0074146587	0,0223068525
ATG16L2	848,8346326	-0,7974470582	0,2007449934	-3,9724380944	0,0000711407	0,0004958592
ATG4B	881,6483603	-0,9033485496	0,1616255887	-5,5891431359	0,0000000228	0,0000005011
ATG4D	360,2391177	-0,8437347571	0,1659847403	-5,0832067777	0,0000003711	0,0000056764
ATG9A	696,8516529	-0,6825162171	0,1610685363	-4,2374010175	0,0000226122	0,0001903641
ATIC	404,8545248	0,6699881531	0,1502542350	4,4590966323	0,0000082306	0,0000813528
ATM	2338,248449	-0,5788871122	0,1968177434	-2,9412362034	0,0032676227	0,0113574502
ATP11A	314,8435593	1,1096235258	0,4047636616	2,7414109297	0,0061175945	0,0190036514
ATP13A3	381,8381978	0,5850901846	0,2051119620	2,9012843899	0,0037162457	0,0127832014
ATP1A1	997,8284953	1,2800397508	0,3699318331	3,4602043847	0,0005397655	0,0026256755
ATP1B1	179,4380108	1,5967298539	0,3699036546	4,3201117465	0,0000155950	0,0001387706
ATP23	61,77779364	-0,6382359835	0,2390580027	-2,6700206541	0,0075805479	0,0227450235
ATP2A2	744,8758504	1,3789945561	0,4205562575	3,2789768400	0,0010418419	0,0044730267
ATP2B1	522,7204146	1,2222484843	0,1872106108	6,5287350908	0,0000000001	0,0000000030
ATP2B1-AS1	55,95420801	2,0967559801	0,3575238734	5,8646600573	0,0000000045	0,0000001229

ATP2C1	393,5083874	1,1123702356	0,2922769336	3,8093003182	0,0001393606	0,0008636251
ATP5F1E	1030,336220	0,5988163711	0,1920282789	3,1183743497	0,0018185166	0,0070927945
ATP5MF-PTCD1	37,84630682	-1,5475722206	0,4889388848	-3,1651649494	0,0015499506	0,0062034893
ATP5PD	432,7489136	0,7305766517	0,1854796291	3,9388654040	0,0000818713	0,0005548777
ATP5PF	364,9373154	0,3792652088	0,1549060006	2,4483571147	0,0143509338	0,0381294056
ATP5PO	844,2555766	0,6457770400	0,1149400485	5,6183815785	0,0000000193	0,0000004360
ATP6AP2	1784,526151	0,4365987141	0,1524541701	2,8638030285	0,0041858822	0,0139910539
ATP6V0A1	252,8689063	2,1413410237	0,4035612982	5,3047964549	0,0000001128	0,0000020199
ATP6V0C	716,7217473	0,8254199006	0,2850041979	2,8961675187	0,0037775069	0,0129443083
ATP6V0D1-DT	43,70700704	-1,3905867953	0,3686198403	-3,7724144046	0,0001616754	0,0009741054
ATP6V1C1	511,6155115	0,5198614212	0,1755071411	2,9620528146	0,0030559536	0,0108500072
ATP6V1C2	17,3963528	1,1819583076	0,4389542404	2,6963542237	0,0070103074	0,0212838471
ATP6V1H	329,4330927	0,3589399974	0,1409612761	2,5463730699	0,0108848789	0,0304088809
ATP8B1	157,6531171	5,3730483407	0,8519145655	6,3070272045	0,0000000003	0,0000000106
ATP8B4	253,3043636	0,8999697782	0,3372581168	2,6684816908	0,0076194931	0,0228364234
ATRIP	161,632743	-0,7567386340	0,1886328594	-4,0117010172	0,0000602828	0,0004326754
ATRN	347,1188729	0,9233784634	0,2715314234	3,4006320586	0,0006723025	0,0031355482
ATRX	925,2904323	-0,4630564309	0,1517369840	-3,0517062706	0,0022754465	0,0085265895
AURKA	76,06892633	1,3052256767	0,4648870737	2,8076402861	0,0049905938	0,0160778823
AURKB	31,49323615	2,1784605468	0,5070982510	4,2960346741	0,0000173880	0,0001524710
AVL9	255,675225	0,6479818066	0,2557315670	2,5338358276	0,0112821576	0,0312657691
AVPR2	19,24983058	3,1553417712	1,2614102382	2,5014397979	0,0123689470	0,0337288857
AXIN2	23,98504097	2,3799475384	0,5375868571	4,4270839761	0,0000095511	0,0000916308
AXL	71,02359065	3,2569932711	0,4572670071	7,1227394013	0,0000000000	0,0000000001
AZI2	292,4481420	0,4979987653	0,1401959938	3,5521611687	0,0003820808	0,0019787637
AZIN1	824,797068	1,2685123339	0,2842299121	4,4629797208	0,0000080828	0,0000801725
B3GALNT1	102,5664816	2,0591160422	0,3185948802	6,4632200441	0,0000000001	0,0000000044
B3GALT4	224,0008293	-0,8637175543	0,2695819504	-3,2039146281	0,0013557267	0,0055566631
B3GALT6	257,9417867	-0,4266202065	0,1729377227	-2,4669008004	0,0136288108	0,0365790217
B3GAT3	354,0033063	-0,5536974971	0,1220132600	-4,5380408458	0,0000056787	0,0000593906
B3GNT5	109,2433114	2,2874604043	0,7190544991	3,1812058850	0,0014666336	0,0059296754
B3GNT9	32,37271412	1,5779648727	0,6732258026	2,3438865040	0,0190839777	0,0478789077
B4GALNT1	17,1402926	5,1406411491	0,9458940248	5,4346803716	0,0000000549	0,0000010739
B4GALNT3	70,55651471	3,4380992257	0,9944690937	3,4572207888	0,0005457775	0,0026482210
B4GALT2	49,70264944	1,8609740500	0,5583035316	3,3332657678	0,0008583291	0,0038278262
B4GALT3	591,2127014	-0,7655987058	0,1595562113	-4,7982978519	0,0000016002	0,0000200546
B4GALT5	444,4959123	1,3084607968	0,5352659635	2,4445058830	0,0145050715	0,0384478658
B4GALT6	61,65298545	0,8837570031	0,3562311806	2,4808524589	0,0131068609	0,0353827413
BACE1	54,9848824	2,6178042684	0,5703632628	4,5897140297	0,0000044385	0,0000482964
BACE2	155,9967915	4,1965358887	0,7553195113	5,5559745590	0,0000000276	0,0000005917
BACH2	204,3412252	-1,6757247562	0,3566871389	-4,6980243487	0,0000026269	0,0000307730
BAG3	194,1488089	1,8891680005	0,5416073777	3,4880765627	0,0004865087	0,0024128258
BAIAP2	195,7702043	1,9206431215	0,5369495649	3,5769525612	0,0003476232	0,0018376588
BAIAP2-DT	44,6051476	0,5680009435	0,2321095666	2,4471342257	0,0144001197	0,0382299036
BAIAP2L1	67,90994641	3,9659076613	0,7925880234	5,0036179508	0,0000005626	0,0000080566
BAMBI	21,93505613	3,4124763334	0,7296182502	4,6770703238	0,0000029100	0,0000336792
BANK1	42,22374337	3,6969567450	0,8811130225	4,1957803945	0,0000271934	0,0002206285
BANP	241,5786434	-1,2829437189	0,2217156789	-5,7865723658	0,0000000072	0,0000001849
BASP1	101,48871	2,9239107568	0,5790198243	5,0497593245	0,0000004424	0,0000066012
BAZ1B	667,7319675	-0,3367162367	0,1221314895	-2,7569977092	0,0058334760	0,0182582992
BAZ2B	470,888806	0,5380476301	0,1940315017	2,7729611149	0,0055543637	0,0175953367
BBS5	49,25165906	0,9000384801	0,3147179094	2,8598260638	0,0042387342	0,0141263756
BCAM	154,6318438	5,6624190514	1,0405580536	5,4417241931	0,0000000528	0,0000010414
BCAR1	109,8902855	8,4061350092	1,4380369787	5,8455629294	0,0000000050	0,0000001362
BCAR3	110,3566972	1,5980852928	0,6865292401	2,3274355349	0,0199420963	0,0496156407
BCAS1	149,2799424	2,4608955868	0,9031503158	2,7247907061	0,0064342259	0,0198270790
BCAS3	376,8379502	-0,7197614937	0,2059725344	-3,4944537421	0,0004750329	0,0023672503
BCAT1	392,6478355	5,7049665832	0,9898726872	5,7633336659	0,0000000082	0,0000002056
BCCIP	308,8422665	0,3908478638	0,1592405402	2,4544494979	0,0141100482	0,0375932798
BCDIN3D	64,27821423	-1,3159093140	0,2349667047	-5,6027920805	0,0000000211	0,0000004693
BCKDHB	119,8399388	0,7197756726	0,2930278019	2,4563391872	0,0140360607	0,0374554674
BCKDK	603,8038588	0,5053037812	0,1586224047	3,1855763514	0,0014445598	0,0058634279
BCL11A	37,95654583	3,1790905967	0,6347957424	5,0080528075	0,0000005498	0,0000078984
BCL11B	303,8411322	-1,0585877003	0,2334173851	-4,5351707622	0,0000057557	0,0000599729
BCL2A1	230,4868065	2,1872557701	0,4715138212	4,6387946051	0,0000035045	0,0000392731
BCL2L2	97,8916675	0,8763586971	0,3405437558	2,5734838440	0,0100713344	0,0286016764
BCL7A	22,05637455	2,0121933823	0,6582246183	3,0570011002	0,0022356346	0,0084021391
BCLAF1	2103,122059	0,3554789433	0,1232515334	2,8841746399	0,0039244109	0,0133288353
BCO2	45,06502836	1,5339001422	0,3471725063	4,4183514365	0,0000099457	0,0000950096
BCOR	376,4280736	-0,7885958063	0,2194532585	-3,5934573572	0,0003263191	0,0017450357
BDH2	185,3756477	0,5752143142	0,1534433553	3,7487078726	0,0001777480	0,0010572195
BEST1	64,57375014	2,0713264273	0,4800658336	4,3146716191	0,0000159840	0,0001418227
BEX3	140,0395711	2,8849976280	0,4102589358	7,0319775398	0,0000000000	0,0000000001
BHLHE40-AS1	57,00756035	-0,5478910881	0,2293249605	-2,3891472038	0,0168875345	0,0433707716
BHLHE41	204,6518754	7,7307360752	1,0944132555	7,0638180196	0,0000000000	0,0000000001
BICC1	22,13359837	5,2010807316	0,6759421214	7,6947637254	0,0000000000	0,0000000000
BIK	42,58645315	3,6624064250	0,9163515034	3,9967266370	0,0000642244	0,0004559390
BIN2	2950,61679	-0,7197971195	0,3076903992	-2,3393551485	0,0193170607	0,0483465298
BIRC3	396,2183329	-1,0235359109	0,4007180271	-2,5542674654	0,0106411480	0,0298271247
BIRC5	90,95478348	3,1396337110	0,6088056069	5,1570381011	0,0000002509	0,0000040202
BLNK	33,27581077	2,2444831876	0,5731010549	3,9163829285	0,0000898874	0,0006004391
BLOC1S3	108,264487	-0,9185460871	0,2060419813	-4,4580530682	0,0000082707	0,0000814965
BLOC1S4	297,8962759	-0,9936706313	0,2032989764	-4,8877065738	0,0000010202	0,0000136768
BLOC1S6	190,7798589	-0,7946040572	0,2351778859	-3,3778858674	0,0007304539	0,0033578926
BLTP2	1281,20963	0,2847299650	0,0975763295	2,9180229115	0,0035225847	0,0121855537
BLVRB	453,2017468	1,8339594057	0,3821344999	4,7992510655	0,0000015926	0,0000199911
BMAL1	643,6677753	-1,1478912340	0,2394155336	-4,8146662965	0,0000014745	0,0000187405
BMAL2	42,06273651	2,9064030840	0,6842089158	4,2478298906	0,0000215851	0,0001829804
BMF	72,13211995	-0,8368632663	0,2676027270	-3,1272598592	0,0017644390	0,0069268833
BMP1	153,4708389	1,3891298295	0,4590362955	2,9826180256	0,0028579443	0,0102626887
BMP2K	127,8937295	1,9466707347	0,3787310105	5,1399824176	0,0000002748	0,0000043409
BMP8A	65,27575381	-1,0662293476	0,3144811446	-3,3907576521	0,0006969970	0,0032307989
BNIP3	277,297405	1,5149063799	0,3992661895	3,7942265583	0,0001481044	0,0009073669
BNIP3L	836,427971	0,5575535624	0,2253882636	2,4737459983	0,0133704719	0,0359717273
BOD1L1	776,8530516	-0,5262316590	0,1775604961	-2,9636753136	0,0030398885	0,0108074218
BOLA1	92,84163037	-1,6090678231	0,2692602516	-5,9758832716	0,0000000023	0,0000000673
BOLA3-DT	32,30696455	-0,7669019359	0,3213477573	-2,3865171565	0,0170088137	0,0436322914
BORCS6	235,5264825	-1,5878021914	0,3001293118	-5,2903936040	0,0000001221	0,0000021484
BPNT2	544,1991139	0,7217070076	0,2721164930	2,6521886944	0,0079959457	0,0237565935
BPTF	972,08625	-0,5773748569	0,1564010353	-3,6916306586	0,0002228209	0,0012789633
BRD3	476,6822704	-0,6872238451	0,1893207938	-3,6299966434	0,0002834249	0,0015564664
BRD3OS	163,8472617	-0,4081776842	0,1688340579	-2,4176264516	0,0156221050	0,0408205166
BRI3	126,3898847	1,1420394600	0,4156851239	2,7473666830	0,0060075927	0,0187120784
BRI3BP	146,8996778	0,5416503741	0,2254037310	2,4030231073	0,0162601599	0,0420887986
BSDC1	719,6841989	-0,6620923176	0,1675439466	-3,9517531427	0,0000775807	0,0005314182
BSPRY	73,99090605	1,7705303191	0,6789620884	2,6077012977	0,0091152458	0,0263625296
BST1	93,49511772	2,7063087132	0,5793132878	4,6715805940	0,0000029889	0,0000344146
BST2	1088,669007	0,6808743039	0,1900782978	3,5820528727	0,0003409033	0,0018100824
BTBD1	677,8933222	0,5004420589	0,1479143193	3,3833238502	0,0007161410	0,0033043187
BTBD19	21,18782725	2,6862929449	0,6293150473	4,2685879880	0,0000196705	0,0001680874
BTBD3	84,32433658	3,6390612829	0,6849510415	5,3128779468	0,0000001079	0,0000019444
BTBD6	413,323278	-0,4657965541	0,1821475203	-2,5572489452	0,0105503683	0,0296158411
BTD	201,8782451	-0,5381588729	0,1279091550	-4,2073561742	0,0000258376	0,0002115412
BTF3	1557,053714	0,7891572983	0,2085617929	3,7838056889	0,0001544484	0,0009406578
BTF3L4	302,1222504	0,8657917305	0,2166456144	3,9963501357	0,0000643266	0,0004564236
BTG1	6488,286311	-0,7120618607	0,3042174866	-2,3406342240	0,0192510172	0,0482322070
BTG3	159,0174921	0,9942048692	0,3348216514	2,9693564780	0,0029842418	0,0106348675
BTK	112,5534596	1,9508107859	0,6449045219	3,0249606256	0,0024866550	0,0091574216
BTN2A1	605,3326541	-0,4566258059	0,1964117595	-2,3248394451	0,0200805469	0,0498855440
BTN2A2	234,7989889	-0,7624534580	0,2723194932	-2,7998489898	0,0051126518	0,0163887338
BTN3A2	2306,808644	-1,2894281004	0,3293487604	-3,9150841156	0,0000903727	0,0006030820
BTN3A3	1318,842579	-1,0386189141	0,2902058361	-3,5789042978	0,0003450378	0,0018294286
BUB1	107,7998652	1,0497785159	0,4503760961	2,3308820582	0,0197589994	0,0492693835
BUB1B	41,63069105	1,9869550973	0,3847673591	5,1640427685	0,0000002417	0,0000038957
BUB3	1323,549205	-0,3882840984	0,1358477274	-2,8582303563	0,0042601100	0,0141723890
BYSL	79,9381229	0,9648196759	0,3658182713	2,6374224966	0,0083538713	0,0245669887
BZW2	227,2930053	1,5016342007	0,2645526181	5,6761267822	0,0000000138	0,0000003231
C10orf143	46,54000741	-1,2660626066	0,3006438985	-4,2111701345	0,0000254051	0,0002083809
C10orf95-AS1	156,1063286	-0,9057434059	0,2060569368	-4,3955988738	0,0000110467	0,0001036160
C11orf58	416,1060662	-1,5028196016	0,2382237990	-6,3084360521	0,0000000003	0,0000000105
C11orf98	228,0019637	-0,4244152317	0,1679019081	-2,5277585541	0,0114793265	0,0317142314
C12orf4	124,8312044	0,7253927415	0,2111446689	3,4355247765	0,0005914074	0,0028288356
C12orf43	132,8901452	-0,4579636074	0,1390959839	-3,2924286947	0,0009932608	0,0043081571
C12orf75	526,6443653	-0,6373420117	0,1966645463	-3,2407570330	0,0011921274	0,0049863379
C14orf119	533,1944765	-0,9054467233	0,1800991407	-5,0274905255	0,0000004969	0,0000072784
C15orf40	248,1543773	-0,5785071673	0,1732662400	-3,3388337358	0,0008413090	0,0037746732
C15orf48	85,51957767	2,4112990817	0,7261353807	3,3207293650	0,0008976255	0,0039787701
C15orf62	39,61025067	1,4173575298	0,5622991642	2,5206466950	0,0117139400	0,0322432787
C16orf54	749,8687058	-1,2030460640	0,4995559711	-2,4082307763	0,0160300445	0,0416532588
C16orf86	32,02713873	-1,4415282255	0,4095338843	-3,5199241885	0,0004316702	0,0021867822
C17orf49	505,2180075	-0,8499388639	0,1862773905	-4,5627618128	0,0000050485	0,0000538280
C17orf67	26,2149205	-1,1636112163	0,3024909297	-3,8467639915	0,0001196882	0,0007517034
C19orf12	232,0032874	-0,7910930011	0,1439025328	-5,4974223592	0,0000000385	0,0000007929
C19orf25	234,4756423	-1,0134679520	0,1757269048	-5,7672896088	0,0000000081	0,0000002024
C19orf38	21,99554969	1,9454628308	0,5442359488	3,5746586570	0,0003506717	0,0018473742
C19orf47	77,76261643	-0,4240469967	0,1639212275	-2,5867365426	0,0096889642	0,0276475426
C19orf84	12,27889769	-1,9978673456	0,5648117547	-3,5372269235	0,0004043521	0,0020709966
C1orf115	29,85807197	2,0065228222	0,7767062825	2,5833740081	0,0097826195	0,0279489585
C1orf159	211,040088	-0,5301037527	0,1884866466	-2,8124207316	0,0049170141	0,0158723127
C1orf174	418,1328944	-0,7061039039	0,2183647237	-3,2473372620	0,0011649026	0,0048862823
C1orf198	154,7900196	1,5992713544	0,3952347742	4,0463822104	0,0000520151	0,0003840497
C1orf21	961,1459204	-1,0994071488	0,2405905545	-4,5696189157	0,0000048861	0,0000525297
C1orf216	229,9603377	-0,8332963429	0,2909522056	-2,8640317102	0,0041828614	0,0139882120
C1orf35	165,4926043	-0,5635828673	0,1499196996	-3,7592315664	0,0001704360	0,0010186807
C1orf43	959,62925	0,5825305297	0,1441505490	4,0411258873	0,0000531952	0,0003916887
C1orf54	64,81885143	1,5612892344	0,4839768556	3,2259584652	0,0012555158	0,0052029686
C1orf56	46,52870820	-0,7680895824	0,3175762242	-2,4185981393	0,0155803993	0,0407445452
C1QA	521,7267945	8,7403354733	1,0306840450	8,4801307582	0,0000000000	0,0000000000
C1QB	1064,006803	8,0351845117	1,0145682855	7,9198057286	0,0000000000	0,0000000000
C1QBP	418,743255	0,4753652892	0,1709941856	2,7800084979	0,0054357476	0,0172763076
C1QC	287,365411	9,5419792473	0,9842649294	9,6945232548	0,0000000000	0,0000000000
C1R	779,0978152	2,9819951849	0,6232431500	4,7846417320	0,0000017129	0,0000212107
C1S	863,6109437	5,7991792671	0,6498842311	8,9234035684	0,0000000000	0,0000000000
C2	497,0616034	4,1835660344	0,5488816734	7,6219816355	0,0000000000	0,0000000000
C20orf204	12,53525491	1,6628302186	0,6642659358	2,5031844406	0,0123081384	0,0336039640
C20orf96	21,88855753	1,3201184665	0,4291405713	3,0761912406	0,0020966322	0,0079674430
C21orf91	222,8223801	-0,4567294623	0,1610812629	-2,8353960634	0,0045768905	0,0149963355
C22orf39	392,5261708	-0,9998882770	0,1262817521	-7,9179157761	0,0000000000	0,0000000000
C2CD2	93,8908	0,9138106682	0,2546204906	3,5889125297	0,0003320602	0,0017708124
C2orf27A	27,61874535	1,6858884772	0,6452438520	2,6127827790	0,0089805735	0,0260570208
C2orf42	198,6805065	-1,3395514425	0,2715136018	-4,9336621345	0,0000008070	0,0000110955
C2orf68	374,4643077	-0,8813900490	0,1902614177	-4,6325211898	0,0000036124	0,0000402840
C2orf74	105,9137758	-0,9796190410	0,2288410684	-4,2807833737	0,0000186237	0,0001614130
C3	2587,568367	7,4581029171	0,6165109457	12,0972777192	0,0000000000	0,0000000000
C3orf14	22,26262144	2,7934018047	0,6927749252	4,0321824236	0,0000552589	0,0004046708
C3orf18	112,9668717	-0,5944709376	0,2122157237	-2,8012577357	0,0050903846	0,0163407051
C4A	263,2251631	3,1597245313	0,6611776857	4,7789328413	0,0000017623	0,0000217816
C4B	292,6820389	3,8487178820	0,6236862649	6,1808298962	0,0000000006	0,0000000218
C4orf19	44,01737082	2,3878371235	0,6318411114	3,7791734037	0,0001573498	0,0009544518
C4orf48	32,23846331	1,2282343152	0,4899851331	2,5066768974	0,0121872127	0,0333278916
C5orf15	289,8261783	0,9731353541	0,2840422286	3,4260235140	0,0006124874	0,0029038575
C5orf34	23,39159242	-0,9827404993	0,3743450567	-2,6252263296	0,0086591387	0,0252988945
C6orf132	163,8372856	2,9385446502	0,6942957604	4,2324104767	0,0000231200	0,0001937301
C6orf47	357,9451291	-0,8109732111	0,1653989636	-4,9031341753	0,0000009432	0,0000127585
C7orf50	311,7079593	0,8750290235	0,1822212853	4,8020132341	0,0000015708	0,0000198151
C8orf76	106,124227	-0,4637157603	0,1498860957	-3,0937877062	0,0019761884	0,0075923474
C9orf40	27,93275648	-1,0598885623	0,2174047675	-4,8751762638	0,0000010871	0,0000144733
C9orf72	201,5234683	1,6709433905	0,3211022888	5,2037729063	0,0000001953	0,0000032286
C9orf78	995,8256874	-0,6529085577	0,1932682169	-3,3782335751	0,0007295309	0,0033547951
CA11	49,29120496	-1,1860115631	0,2228454269	-5,3221265499	0,0000001026	0,0000018630
CA12	287,9861599	8,8287699066	0,9637559346	9,1607943358	0,0000000000	0,0000000000
CA2	101,206526	3,7074488378	0,7326742912	5,0601588954	0,0000004189	0,0000063212
CA5B	221,4263383	-0,5205968897	0,2097143414	-2,4824095779	0,0130497183	0,0352708254
CAB39L	89,34480096	-0,5276221773	0,2214770528	-2,3822882358	0,0172054245	0,0440284073
CABLES1	105,8285208	1,5959936416	0,3516940290	4,5371644384	0,0000057016	0,0000599321
CABYR	11,98958455	1,8091281909	0,6025832852	3,0022868788	0,0026798953	0,0097374061
CACNB3	52,38252537	1,2988012778	0,4980270493	2,6078930445	0,0091101415	0,0263565097
CACUL1	398,176602	0,4751584360	0,1318789244	3,6029899266	0,0003145777	0,0016943642
CALCOCO1	2042,049288	-0,9614237831	0,2220385880	-4,3299656242	0,0000149133	0,0001338217
CALCRL	15,89954492	1,7822071708	0,6951676942	2,5637082758	0,0103560546	0,0291982604
CALD1	86,22887498	5,6614768257	0,8308746863	6,8138778578	0,0000000000	0,0000000006
CALHM2	558,8695225	-1,0742258743	0,3883777739	-2,7659879370	0,0056715856	0,0178697430
CALM2	4596,902648	0,7571951154	0,1685910358	4,4913130284	0,0000070785	0,0000715832
CALML4	25,9766065	2,6259593606	0,7400232987	3,5484820075	0,0003874585	0,0020019927
CALR	2681,319479	1,2960180053	0,3537048821	3,6641224574	0,0002481880	0,0013948270
CALU	910,2131668	1,3541794077	0,2340701771	5,7852564439	0,0000000072	0,0000001859
CAMK2D	268,224183	0,7071191748	0,2446032749	2,8908818778	0,0038416248	0,0131131976
CAMK2N1	178,6666623	3,9299126366	1,0338619725	3,8011866213	0,0001439990	0,0008862488
CAMK4	91,29184071	1,2348257321	0,5123133782	2,4102837474	0,0159396810	0,0414906414
CAMSAP2	51,18083948	3,9661703539	0,7701189219	5,1500751913	0,0000002604	0,0000041427
CANX	3668,670724	0,9283347489	0,1741144766	5,3317493587	0,0000000973	0,0000017765
CAPG	908,0531556	2,1097870651	0,2461648388	8,5706272380	0,0000000000	0,0000000000
CAPN10	453,3470448	-0,5264749429	0,1716420427	-3,0672943001	0,0021601330	0,0081649509
CAPRIN1	1206,16411	0,5722534670	0,1660520389	3,4462298932	0,0005684670	0,0027415139
CAPZA2	1153,721366	0,5714414265	0,1873722107	3,0497661551	0,0022901961	0,0085723135
CARD10	39,97032072	3,5793580130	0,8251685121	4,3377345046	0,0000143965	0,0001298777
CARD11	1301,887724	-0,5858588663	0,1588180923	-3,6888672942	0,0002252547	0,0012901820
CARD16	290,8702111	-0,9139913210	0,2667070497	-3,4269484898	0,0006104048	0,0028950138
CARD19	650,2726958	-1,2290102314	0,3447687573	-3,5646517276	0,0003644786	0,0019076370
CARD6	75,22100343	1,1670247080	0,4510896412	2,5871227143	0,0096780827	0,0276282093
CARD8	901,3453119	-0,8617913471	0,2222352058	-3,8778344948	0,0001053904	0,0006849611
CARD8-AS1	75,63516229	-0,7549188010	0,3151888013	-2,3951320536	0,0166143803	0,0427671752
CARD9	58,05416554	3,2468606727	0,7258560121	4,4731470413	0,0000077077	0,0000770674
CARMIL1	25,46499682	4,8670052674	0,8695075497	5,5974273818	0,0000000218	0,0000004832
CARMIL2	89,84128126	-2,2727107974	0,4178291865	-5,4392987473	0,0000000535	0,0000010517
CARNMT1	144,8241881	0,7541504464	0,2497835040	3,0192163791	0,0025342946	0,0093023193
CARNS1	214,7820897	-1,1857796273	0,4520564800	-2,6230784865	0,0087139196	0,0254203203
CASC3	887,8945913	-0,2870079682	0,1172913277	-2,4469666575	0,0144064166	0,0382390785
CASK	364,7729897	0,7034343375	0,2437113854	2,8863417123	0,0038974874	0,0132678297
CASP2	345,0771535	-0,3338948603	0,1398422107	-2,3876543325	0,0169562817	0,0435141185
CASP6	78,4110836	0,7260574808	0,2894279407	2,5085849855	0,0121212375	0,0332081938
CASP7	321,3166148	1,0703668025	0,3073284908	3,4828655462	0,0004964111	0,0024510980
CASP8AP2	346,7231574	-0,7193551634	0,2388000225	-3,0123495437	0,0025923391	0,0094766116
CASTOR2	188,4462293	-0,7197656625	0,2758952194	-2,6088362967	0,0090850693	0,0262978565
CASTOR3P	10,17296978	-2,4261409738	0,9042425620	-2,6830643411	0,0072950954	0,0220112046
CATSPER1	76,10675294	-1,0230870249	0,3122272779	-3,2767381239	0,0010501372	0,0045015435
CATSPERB	14,19591629	1,6356673655	0,6467453082	2,5290748070	0,0114363649	0,0316215020
CAV1	144,8128307	3,5669404288	0,7755963848	4,5989647431	0,0000042460	0,0000464639
CAV2	90,24492717	5,8423205580	0,9041762527	6,4614841854	0,0000000001	0,0000000044
CAVIN1	264,5962116	6,6620172665	0,7514782987	8,8652157731	0,0000000000	0,0000000000
CAVIN3	57,63768813	7,4636347384	1,0965332374	6,8065734835	0,0000000000	0,0000000006
CBLB	1304,071743	-0,7034901789	0,2526731404	-2,7841905859	0,0053661493	0,0170873833
CBLL1	445,8343319	-0,5725306004	0,1644823754	-3,4808021165	0,0004999147	0,0024626764
CBLL1-AS1	14,51691826	-1,1633002394	0,3743116798	-3,1078384727	0,0018846104	0,0073082271
CBR4	145,318456	-0,5832100061	0,2453070489	-2,3774594153	0,0174318885	0,0444802312
CBS	89,69838812	5,6674475591	0,9260370165	6,1201090862	0,0000000009	0,0000000308
CBX3	784,3527429	0,4763910775	0,1765929091	2,6963053757	0,0070113357	0,0212838471
CBX6	364,3141778	-0,4062669610	0,1744351588	-2,3290428598	0,0198567953	0,0494680233
CBX7	456,7110315	-0,7096909673	0,2445966631	-2,9014744450	0,0037141107	0,0127791233
CCAR2	1114,897413	-0,5423838312	0,2081202529	-2,6061078808	0,0091577613	0,0264684276
CCDC102A	157,3863824	-1,3011975940	0,2106352063	-6,1774904536	0,0000000007	0,0000000222
CCDC106	20,27862094	1,3723084574	0,4888627576	2,8071446152	0,0049982797	0,0160948390
CCDC107	248,1518839	-0,5373200360	0,2010776202	-2,6722320857	0,0075355252	0,0226301137
CCDC112	46,41979812	0,8311483736	0,3163288995	2,6273902686	0,0086042588	0,0251604005
CCDC115	559,2443919	-0,4747718112	0,1273688521	-3,7275346636	0,0001933620	0,0011345476
CCDC12	540,1035291	-0,5998276945	0,1704781253	-3,5185024090	0,0004339899	0,0021968799
CCDC134	190,3982321	-0,5481324884	0,1626920148	-3,3691490276	0,0007534595	0,0034423268
CCDC136	53,48235378	-1,1283336542	0,4431604052	-2,5460228116	0,0108969285	0,0304355971
CCDC141	35,1481226	2,1348998201	0,4490412007	4,7543519768	0,0000019908	0,0000242066
CCDC142	265,0190566	-1,1185084665	0,3201874603	-3,4932922130	0,0004771041	0,0023749340
CCDC149	31,87016126	2,9239708191	0,5647306830	5,1776376019	0,0000002247	0,0000036574
CCDC157	70,20920762	-0,8798850958	0,2256756817	-3,8980057091	0,0000969881	0,0006390172
CCDC159	240,4033184	-0,5877422864	0,1216129604	-4,8328926940	0,0000013456	0,0000173157
CCDC28A	221,3409899	-0,8966461534	0,2944494827	-3,0451612455	0,0023255559	0,0086661887
CCDC28B	91,94549733	-1,0970787342	0,2119061040	-5,1771926505	0,0000002252	0,0000036617
CCDC30	61,32905365	-0,5268913345	0,2177686865	-2,4286843449	0,0151537317	0,0398377401
CCDC32	256,8301424	-0,7031425623	0,1956529345	-3,5875032248	0,0003212865	0,0017229149
CCDC43	158,8469208	0,5879351340	0,2461382083	2,3886566672	0,0169100968	0,0434121501
CCDC47	610,3152511	0,8321005159	0,1499745143	5,5482794505	0,0000000288	0,0000006144
CCDC50	284,2280276	1,7626589126	0,2248339181	7,8398265154	0,0000000000	0,0000000000
CCDC59	280,1604065	0,6046660007	0,2202405580	2,7454797888	0,0060422489	0,0187999700
CCDC6	289,4305223	1,9663014077	0,4980389792	3,9480794508	0,0000787807	0,0005377220
CCDC61	86,68843379	-0,5832226295	0,1886482083	-3,0916251104	0,0019906408	0,0076401553
CCDC65	47,81746365	-0,9551726789	0,3605747718	-2,6490280344	0,0080723635	0,0239484269
CCDC69	2175,925175	-0,8031389790	0,2996041100	-2,6851485890	0,0072497568	0,0218842175
CCDC77	63,06245131	-0,5720078639	0,1788541577	-3,1981804120	0,0013829773	0,0056477167
CCDC80	632,5739364	5,5181266791	0,7474761210	7,3823455014	0,0000000000	0,0000000000
CCDC85B	217,3517627	-0,3810832850	0,1341589927	-2,8405771270	0,0045031982	0,0148088538
CCDC85C	217,2053048	1,5825421032	0,6517107560	2,4282906008	0,0151701825	0,0398731999
CCDC86	108,8773188	0,6563549281	0,2329360623	2,8177471603	0,0048361877	0,0156666874
CCDC88A	265,0161539	1,4249087299	0,3523250378	4,0443016448	0,0000524793	0,0003868410
CCDC88B	2357,094931	-0,8061202646	0,2329141062	-3,4610195060	0,0005381338	0,0026192248
CCDC88C	1676,55225	-0,7643963741	0,1892912671	-4,0384132151	0,0000538140	0,0003953801
CCDC92	493,8521349	-0,8856160031	0,2479567593	-3,5716550161	0,0003547325	0,0018649738
CCDC97	380,216338	-0,7214929528	0,1403688915	-5,1399775626	0,0000002748	0,0000043409
CCDC9B	99,88004981	3,0606792308	0,7244347066	4,2249207597	0,0000239025	0,0001982301
CCL20	28,48322138	4,5176896960	0,8702258005	5,1913893989	0,0000002087	0,0000034282
CCL5	12610,84413	-1,0402137476	0,3488487803	-2,9818471676	0,0028651499	0,0102812364
CCL8	49,9338768	-2,6135354115	1,0803948843	-2,4190557078	0,0155608569	0,0407092583
CCM2	962,7692032	-1,0172552878	0,2065498162	-4,9249881037	0,0000008437	0,0000115755
CCN1	297,3392127	11,0168753194	1,2227205159	9,0101338948	0,0000000000	0,0000000000
CCN2	244,2060453	9,4942592438	1,2774035843	7,4324663609	0,0000000000	0,0000000000
CCNA2	56,21626181	2,3973821369	0,5672279134	4,2264900584	0,0000237365	0,0001973287
CCNB1	160,6415329	2,2908127687	0,4023989313	5,6929180906	0,0000000125	0,0000002980
CCNB2	68,03171236	3,8515922237	0,5314755929	7,2469785729	0,0000000000	0,0000000000
CCNC	269,3331951	0,4151148045	0,1543376953	2,6896527389	0,0071526407	0,0216347289
CCND1	372,2765424	6,3824520375	1,0088849751	6,3381800076	0,0000000002	0,0000000089
CCNDBP1	1253,754619	-0,8708189940	0,1901311680	-4,5800959581	0,0000046476	0,0000502549
CCNE1	48,56842638	2,9168904139	0,6937605861	4,2044625655	0,0000261703	0,0002137455
CCNE2	19,27208157	1,4769318552	0,5575341597	2,6490429880	0,0080720064	0,0239484269
CCNJL	280,9893006	-1,4355456145	0,3145246869	-4,5641746872	0,0000050146	0,0000536525
CCNL1	3432,000327	0,7863145678	0,1888841548	4,1629461641	0,0000314167	0,0002493234
CCNYL1	68,46097176	1,2992443382	0,2798126616	4,6432649984	0,0000034295	0,0000386532
CCR1	508,6206755	2,2185001993	0,5924615845	3,7445468530	0,0001807197	0,0010711081
CCR2	88,2525105	3,4641856041	0,7294530447	4,7555372707	0,0000019792	0,0000241085
CCR5	224,7710571	1,8893695838	0,5294606713	3,5684795611	0,0003590589	0,0018847200
CCR6	32,16458046	2,1820653073	0,8903545733	2,4507823879	0,0142546102	0,0379183900
CCR9	19,82433433	1,8338764996	0,7361765462	2,4910821458	0,0127354671	0,0345533940
CCS	361,612259	-0,8892487824	0,2016263095	-4,4103867418	0,0000103189	0,0000978915
CCSAP	101,4985502	0,6632478278	0,1862290538	3,5614627497	0,0003687944	0,0019232751
CCSER2	1274,180568	-0,7558297805	0,2738370492	-2,7601443371	0,0057775829	0,0181146572
CCT2	775,4075982	0,5330250337	0,1347669486	3,9551643400	0,0000764821	0,0005249610
CCT4	743,8167746	0,5129635584	0,1417569880	3,6179090539	0,0002969925	0,0016167537
CCT5	1323,453885	0,5989726251	0,1792314937	3,3418838427	0,0008320888	0,0037392001
CCT6A	1039,603002	0,3987833200	0,1432047777	2,7847068122	0,0053576143	0,0170642423
CCT8	903,4047343	0,4809481585	0,1450475043	3,3157875448	0,0009138200	0,0040354289
CD101	25,67660726	2,6046900922	0,7858570554	3,3144578574	0,0009182102	0,0040506388
CD109	93,56037643	2,6576651423	0,8224924286	3,2312335651	0,0012325718	0,0051315855
CD14	3264,089669	5,5863585663	0,8465230046	6,5991810452	0,0000000000	0,0000000020
CD151	439,0084081	1,6183558004	0,4372681662	3,7010601855	0,0002147005	0,0012376314
CD160	326,1045338	-1,8705982273	0,5082626496	-3,6802344694	0,0002330196	0,0013250713
CD163	3142,999044	4,9638850849	0,8725378953	5,6890194816	0,0000000128	0,0000003039
CD180	148,3388331	2,6911226079	0,7438640715	3,6177612431	0,0002971623	0,0016167537
CD1D	38,35966419	3,3826352704	0,9288187813	3,6418678521	0,0002706670	0,0014998574
CD200	40,04458272	2,7880244454	0,6533209797	4,2563895383	0,0000207755	0,0001771755
CD24	1461,544839	9,4426728778	1,4006638324	6,7415704320	0,0000000000	0,0000000009
CD244	1100,625518	-1,7695467431	0,2962944235	-5,9722580056	0,0000000023	0,0000000686
CD247	4962,496875	-1,5852441813	0,3053402823	-5,1917295982	0,0000002083	0,0000034283
CD276	214,9869396	3,4394356939	0,6562503327	5,2410421949	0,0000001597	0,0000027171
CD28	100,7539638	1,8197287769	0,6912182207	2,6326362485	0,0084725033	0,0248452928
CD2AP	282,3870116	1,1124277911	0,3037982125	3,6617204565	0,0002505271	0,0014038698
CD2BP2	781,0958697	-0,9683582706	0,1070000457	-9,0500827755	0,0000000000	0,0000000000
CD2BP2-DT	22,05524757	1,2457758010	0,5235407036	2,3795189034	0,0173352539	0,0442924292
CD300C	129,3722333	1,9148723614	0,5416779789	3,5350751808	0,0004076592	0,0020822197
CD300E	316,219947	2,7492080043	1,0993622956	2,5007297551	0,0123937712	0,0337897835
CD300LB	81,10716720	2,2587606573	0,6057940159	3,7285851959	0,0001925502	0,0011303956
CD300LF	36,24465721	1,6252893033	0,6506277325	2,4980326263	0,0124884699	0,0340066367
CD302	83,38257241	2,3241123026	0,8167497234	2,8455623984	0,0044333072	0,0146290531
CD33	106,9039118	3,4287463671	0,7714857083	4,4443420408	0,0000088161	0,0000859882
CD36	983,1331881	2,6042108528	0,8678042334	3,0009195075	0,0026916571	0,0097733218
CD4	511,342941	3,4034612368	0,6559581382	5,1885342042	0,0000002120	0,0000034720
CD46	1601,747527	0,4058144144	0,1649965841	2,4655829491	0,0136786980	0,0366762693
CD55	2243,074726	3,3163902485	0,5669640970	5,8493821722	0,0000000049	0,0000001334
CD58	663,638921	-0,9062545040	0,3450089109	-2,6267568036	0,0086202919	0,0252018091
CD59	741,8223543	1,2127368641	0,3838250225	3,1596086053	0,0015798121	0,0062967932
CD6	951,8862998	-1,3693328102	0,4970289612	-2,7550362594	0,0058685631	0,0183443706
CD63	2999,380154	0,9647133687	0,2138422014	4,5113329515	0,0000064422	0,0000660910
CD69	3039,155465	1,0930965090	0,4319634475	2,5305091133	0,0113946157	0,0314113250
CD74	8820,794193	1,2259943216	0,3268696153	3,7525426770	0,0001749528	0,0010388619
CD79A	14,72311924	-1,3302206484	0,5532224707	-2,4062411412	0,0161200792	0,0419738278
CD79B	108,3425198	-1,4261134397	0,3481810114	-4,0958937826	0,0000420543	0,0003212545
CD81	1283,297697	0,5877974234	0,2242932594	2,6206625580	0,0087758822	0,0255789031
CD82	1509,420121	-1,3402498340	0,5528504265	-2,4242539571	0,0153398740	0,0402450048
CD84	794,9294711	1,5401778267	0,5146356287	2,9927528815	0,0027647252	0,0099928036
CD86	240,7167043	3,2606279915	0,6590819038	4,9472273059	0,0000007528	0,0000104458
CD9	1606,70652	4,0906179721	0,6845055882	5,9760182865	0,0000000023	0,0000000673
CD93	102,6706284	2,2853023006	0,7640604203	3,1349645083	0,0017187490	0,0067802937
CD96	2759,605223	-0,8838579730	0,3687852331	-2,3966739816	0,0165446370	0,0426447364
CDA	42,47168892	2,3742237235	0,6303493123	3,7665206851	0,0001655384	0,0009938209
CDAN1	216,6874183	-0,8815032546	0,1998705294	-4,4103563300	0,0000103201	0,0000978915
CDC20	81,23149176	3,5134963174	0,5892970142	5,9621824522	0,0000000025	0,0000000725
CDC25A	10,36292826	1,8718390762	0,7772007642	2,4084368980	0,0160209912	0,0416474518

CDC26	209,6257064	-0,5720834998	0,1600915229	-3,5734777804	0,0003522711	0,0018536238
CDC34	347,3893649	-0,6514715564	0,1925581511	-3,3832458015	0,0007163449	0,0033043187
CDC37L1	96,93357262	-0,6186280372	0,2138680125	-2,8925692533	0,0038210493	0,0130602651
CDC42	1095,404843	0,7326112774	0,2926433171	2,5034273285	0,0122996937	0,0335877210
CDC42BPA	47,28716762	6,4205670510	0,8229801131	7,8016065620	0,0000000000	0,0000000000
CDC42BPB	118,8669426	4,8169066524	0,8468117201	5,6881672019	0,0000000128	0,0000003043
CDC42BPG	100,8196325	1,4320445188	0,6119630981	2,3400821245	0,0192794483	0,0482703450
CDC42EP1	89,88310251	5,2517654085	0,8061632514	6,5145184813	0,0000000001	0,0000000032
CDC42EP3	722,8299427	-0,8470649832	0,1752318025	-4,8312112864	0,0000013570	0,0000174459
CDC42EP4	115,0436658	5,3184905141	0,8073269470	6,5902274922	0,0000000000	0,0000000021
CDC42SE2	2710,995454	-0,7162788396	0,2400445968	-2,9835240997	0,0028494959	0,0102419935
CDC45	31,63699103	1,8568338810	0,7055460318	2,6317663459	0,0084942258	0,0248818888
CDC6	38,11211616	3,0661497944	0,5739995830	5,3417282610	0,0000000921	0,0000016952
CDCA2	25,03293098	2,6929874235	0,7739251839	3,4796611862	0,0005020483	0,0024690678
CDCA5	46,45993812	1,2880052386	0,4788050109	2,6900412678	0,0071443184	0,0216144134
CDCA7	31,96991397	2,5449950649	0,5491517721	4,6344110936	0,0000035796	0,0000399509
CDCA7L	94,40501604	2,8843781491	0,5631460031	5,1219011292	0,0000003025	0,0000047340
CDCA8	47,27692722	0,8865370282	0,3470528367	2,5544727805	0,0106348741	0,0298219596
CDCP1	216,4801617	4,1137450725	0,5234880721	7,8582208166	0,0000000000	0,0000000000
CDH1	381,8970135	6,6551985411	1,0108830663	6,5835493372	0,0000000000	0,0000000022
CDH2	38,25698642	5,1819688595	0,9692620784	5,3463031054	0,0000000898	0,0000016643
CDH23	42,46618174	1,8933858577	0,6704338685	2,8241203402	0,0047410595	0,0154328080
CDH24	61,73345213	-0,8691680612	0,2904209433	-2,9927916033	0,0027643844	0,0099928036
CDH3	113,2543935	3,9896547212	0,6946954319	5,7430415372	0,0000000093	0,0000002284
CDH6	128,3373248	7,3520052946	1,2267482879	5,9935680151	0,0000000021	0,0000000607
CDHR1	413,8168041	1,7203006783	0,3430081861	5,0153201371	0,0000005295	0,0000076711
CDHR3	33,01647084	-1,0780654702	0,3753854237	-2,8718895360	0,0040802557	0,0137278238
CDIP1	288,509706	-0,5701320674	0,1908272248	-2,9876872539	0,0028109704	0,0101246083
CDIPT	647,0984606	-0,4844853437	0,1445174655	-3,3501164056	0,0008077761	0,0036448762
CDK1	104,3211367	3,6689111554	0,5618847305	6,5296509345	0,0000000001	0,0000000030
CDK10	723,1682079	-0,8786758008	0,2692772745	-3,2630893283	0,0011020478	0,0046844996
CDK11A	475,666568	-0,4446871790	0,1639988682	-2,7115259024	0,0066974308	0,0204740901
CDK14	38,46600896	4,5494124005	0,8281617221	5,4933864716	0,0000000394	0,0000008090
CDK17	689,6129080	-0,5758826616	0,1810462496	-3,1799821274	0,0014734616	0,0059504328
CDK18	87,987419	3,2184365752	0,8364102335	3,8479163049	0,0001191267	0,0007584890
CDK2AP1	300,5940495	1,6123344305	0,2810982201	5,7358196507	0,0000000097	0,0000002371
CDK2AP2	825,8228975	-0,3839174146	0,1322144210	-2,9037484085	0,0036872433	0,0126921701
CDK6	296,220466	0,7744711095	0,2619159453	2,9569452466	0,0031070329	0,0110083344
CDKN1A	604,385823	2,1575934846	0,6240642076	3,4572361188	0,0005455642	0,0026481406
CDKN1C	27,63467206	2,6817717253	0,9864911732	2,7184854391	0,0065579566	0,0201300104
CDKN2A	61,54777347	3,0492371297	0,8315630051	3,6668744412	0,0002455333	0,0013828522
CDKN2B	46,83208514	4,2029587207	0,8316468847	5,0537779882	0,0000004332	0,0000064925
CDKN3	37,12089795	1,8568279803	0,4680394535	3,9672467063	0,0000727077	0,0005054689
CDON	31,23045892	3,8282511950	0,6640942822	5,7646200206	0,0000000082	0,0000000048
CDPF1	106,3016826	-0,6314205759	0,2517638261	-2,5079876867	0,0121420898	0,0332382609
CDV3	1107,172328	0,5739026775	0,2055930448	2,7914498665	0,0052472486	0,0167523638
CEACAM1	40,18701538	3,4466777650	0,6281680455	5,4868721689	0,0000000409	0,0000008343
CEACAM21	108,3005209	-1,5357682859	0,4411511489	-3,4812745922	0,0004990326	0,0024622406
CELF1	1226,046672	-0,4706550622	0,1096529893	-4,2922228121	0,0000176893	0,0001545090
CELSR1	216,5979867	4,1361106964	0,7008586369	5,9014906557	0,0000000036	0,0000001011
CEMIP	206,5605998	4,5270796919	0,6273432041	7,2162716371	0,0000000000	0,0000000000
CENPA	14,04641720	3,0540222091	0,8629930075	3,5388724853	0,0004018399	0,0020620039
CENPBD1P	102,2164967	-1,1036932528	0,2793785426	-3,9505297811	0,0000779784	0,0005335990
CENPE	30,24742753	0,9818212268	0,3483420998	2,8185545968	0,0048240406	0,0156403345
CENPF	86,65259820	1,0739653410	0,3826900062	2,8063018102	0,0050113730	0,0161286745
CENPM	28,43850618	1,7992203567	0,4566456185	3,9400802931	0,0000814544	0,0005523304
CENPT	962,2403614	-1,1265682826	0,2453703921	-4,5917042918	0,0000043964	0,0000479155
CENPU	47,27637513	0,9403561779	0,3257957201	2,8863368054	0,0038975482	0,0132678297
CENPW	34,58918271	4,1351385351	0,6312022138	6,5512104439	0,0000000001	0,0000000026
CEP170	191,4782766	1,2284046666	0,2236975274	5,4913645258	0,0000000399	0,0000008159
CEP19	49,76959603	-1,8838187239	0,4339186955	-4,3414094474	0,0000141572	0,0001281475
CEP250	676,5953813	-0,5908344953	0,2085507428	-2,8330481054	0,0046106303	0,0150958607
CEP350	918,7141569	-0,5978942261	0,2112435063	-2,8303555300	0,0046496305	0,0152050597
CEP55	55,112519	4,6036621787	0,7426631661	6,1988562094	0,0000000006	0,0000000197
CEP70	94,73197534	1,7541875259	0,4402078606	3,9849073424	0,0000675065	0,0004754772
CEP78	717,8684201	-0,7670909349	0,2030305326	-3,7782048095	0,0001579629	0,0009577383
CEP83	67,63256799	0,6014512894	0,2519344719	2,3873322483	0,0169711459	0,0435428619
CEP85	164,57511	-0,5944034344	0,1880184983	-3,1614093279	0,0015700769	0,0062709897
CERK	1081,100557	-1,0361087051	0,2725621491	-3,8013688018	0,0001439001	0,0008860454
CERS4	280,3588017	-0,6735104121	0,2827939484	-2,3816295080	0,0172362289	0,0440812541
CERS5	518,1278526	-0,5382525760	0,1964926611	-2,7394405854	0,0061543837	0,0190958979
CERS6	282,4324688	1,0168376513	0,2294797985	4,4310551861	0,0000093773	0,0000903497
CES1	36,04197466	-2,0259968606	0,7394852294	-2,7397390863	0,0061487975	0,0190829639
CETN2	164,0048322	1,0039869681	0,3286531544	3,0549709620	0,0022515737	0,0084535853
CFAP298-TCP10L	153,3562859	-1,0057739983	0,3757298397	-2,6768541970	0,0074316968	0,0223482439
CFB	2761,898103	8,9138748136	0,7368540749	12,1004893849	0,0000000000	0,0000000000
CFD	63,70684312	2,3729704330	0,6644048815	3,5715728454	0,0003549438	0,0018649738
CFH	315,1229870	4,1795115671	0,7083889101	5,9000239942	0,0000000036	0,0000001015
CFI	188,6543554	5,3260140922	0,5669586849	9,3955646237	0,0000000000	0,0000000000
CFL2	160,4728607	1,0992201076	0,3337029096	3,2940081614	0,0009876963	0,0042871307
CFLAR	2880,985218	-0,3696944615	0,1338079099	-2,7628744955	0,0057294792	0,0180015851
CFP	84,01714874	2,3483070402	0,8274831668	2,8378910103	0,0045412586	0,0149086526
CGN	120,8589707	4,7487561203	0,9345556241	5,0812985424	0,0000003749	0,0000057272
CGNL1	16,05645112	3,2010666666	0,8130375361	3,9371695949	0,0000824483	0,0005578245
CHCHD10	59,21608611	2,7435044519	0,5790677404	4,7377953573	0,0000021606	0,0000258421
CHCHD3	333,9526891	0,3591348116	0,1327926791	2,7044774899	0,0068411905	0,0208661735
CHCHD4	97,91393797	-0,7173424180	0,2661109368	-2,6956517713	0,0070251066	0,0213160358
CHCHD6	46,48463554	1,0937395792	0,4515779358	2,4220394589	0,0154336746	0,0404315415
CHDH	19,94530774	1,0076720329	0,3683903539	2,7353377258	0,0062316304	0,0192866738
CHEK1	28,37395383	1,2364887854	0,4467545972	2,7677126245	0,0056451035	0,0178113206
CHFR	612,7184107	-0,4333966637	0,1709531734	-2,5351776460	0,0112390315	0,0311956045
CHI3L2	81,08487663	-1,8196039928	0,7008040322	-2,5964519456	0,0094192080	0,0270037359
CHID1	410,9549604	0,3921093387	0,1272006587	3,0826046253	0,0020519758	0,0078331239
CHKA	193,1074438	1,0081415219	0,2222248592	4,5365830164	0,0000057173	0,0000597012
CHKB	1123,510295	-0,8320940165	0,1724813763	-4,8242542721	0,0000014053	0,0000179801
CHKB-DT	32,19589023	-0,6648587955	0,2699465600	-2,4629274604	0,0137807794	0,0368966056
CHML	117,9141871	1,7050140830	0,4188006789	4,0711827100	0,0000467750	0,0003501624
CHMP2B	310,3570886	0,8249704312	0,2437069261	3,3850922675	0,0007115433	0,0032844215
CHMP3	594,3951764	0,6730722568	0,2041140615	3,2975300755	0,0009753923	0,0042426776
CHMP3-AS1	52,96242239	1,6750744187	0,5822075421	2,8771087584	0,0040133725	0,0135558027
CHMP6	409,3016312	-0,6401800200	0,2531029112	-2,5293269714	0,0114281507	0,0316100748
CHMP7	500,1909215	-0,6619202066	0,2435513004	-2,7177855571	0,0065720417	0,0201550072
CHN1	9,761419312	1,3324330839	0,4587517202	2,9044753953	0,0036786912	0,0126702106
CHRM3-AS2	63,74882399	1,8483961297	0,4817412316	3,8369066391	0,0001245928	0,0007869698
CHRNB1	128,9083418	-1,1955248460	0,2054137802	-5,8200810331	0,0000000059	0,0000001556
CHST12	1010,602336	-1,0427112714	0,2556444876	-4,0787551539	0,0000452775	0,0003406568
CHST15	386,524338	4,1583005457	0,7449004056	5,5823577415	0,0000000237	0,0000005176
CHST2	708,7438815	-1,6581568974	0,3381429942	-4,9037224859	0,0000009404	0,0000127338
CHSY1	184,4804478	0,6150178926	0,2537052050	2,4241427716	0,0153445293	0,0402450048
CHTF18	300,8402289	-0,7685842717	0,2355975164	-3,2622766293	0,0011052124	0,0046942752
CHTF8	412,3318075	-0,6085532840	0,2154427752	-2,8246539411	0,0047331706	0,0154108553
CHUK	267,3488427	0,5380051712	0,2117414561	2,5436825823	0,0109687573	0,0305798412
CIAO1	1191,892976	-0,3957580783	0,1278615035	-3,0952090153	0,0019667425	0,0075625296
CIAO3	320,3339965	-0,4964332500	0,1645476220	-3,0169585188	0,0025532475	0,0093642265
CIAPIN1	255,86038	-0,4895070585	0,1842335021	-2,6569926363	0,0078841165	0,0234675283
CIITA	330,8084018	1,1550195470	0,3675571498	3,1424216546	0,0016755655	0,0066215829
CILP	32,63080923	2,9421618912	0,8669671609	3,3936256457	0,0006897391	0,0032046559
CIP2A	35,54820178	1,2587134402	0,3730186572	3,3743880790	0,0007397730	0,0033926219
CIT	33,48615581	0,8999200891	0,3609539988	2,4931711298	0,0126607809	0,0343763192
CITED2	372,1913729	1,7934892151	0,4336012298	4,1362641341	0,0000353006	0,0002752873
CITED4	50,53032942	1,5824200626	0,5362914794	2,9506716466	0,0031708381	0,0111877829
CKAP2L	28,09132358	1,5585251605	0,5613350115	2,7764617005	0,0054954109	0,0174412240
CKAP4	211,0150621	2,1100499082	0,4025902842	5,2411863782	0,0000001595	0,0000027171
CKB	112,1236114	3,7723236093	0,7789646519	4,8427404249	0,0000012806	0,0000166219
CKLF	285,1863027	-0,5400018929	0,2130283873	-2,5348704219	0,0112488927	0,0312057082
CKLF-CMTM1	10,52389496	-1,6285131821	0,4455127248	-3,6567726117	0,0002574100	0,0014358636
CKS1B	196,6619892	1,2590816865	0,3645014477	3,4523096192	0,0005537530	0,0026840172
CKS2	147,9549391	1,1456494646	0,4406870542	2,5996894322	0,0093308171	0,0268328346
CLBA1	44,82441945	0,8441487752	0,3463284094	2,4374228399	0,0147923708	0,0390709008
CLCC1	217,1298695	0,5901036605	0,1830559745	3,2236241510	0,0012657942	0,0052375006
CLCN5	26,2995684	1,6302836645	0,4413600262	3,6937728106	0,0002209512	0,0012687728
CLDN1	412,1196855	11,2791998969	1,2736531953	8,8557997186	0,0000000000	0,0000000000
CLDN12	91,73431994	4,2403473331	0,7626521375	5,5600018984	0,0000000270	0,0000005810
CLDN15	110,4968833	-0,8188200637	0,2097631707	-3,9036499487	0,0000947528	0,0006280437
CLDN4	1460,230275	10,0308967392	1,3574161161	7,3896891647	0,0000000000	0,0000000000
CLDN7	288,2219019	6,0886231462	1,0274188214	5,9261354953	0,0000000031	0,0000000888
CLDND1	1535,470644	-0,9112814335	0,2749549242	-3,3179140563	0,0009069239	0,0040115659
CLDND2	49,28026898	-0,9319580751	0,3782503726	-2,4632191285	0,0137695732	0,0368832748
CLEC10A	123,1662325	3,4419486187	0,9011639022	3,8194479500	0,0001337507	0,0008336792
CLEC12A	51,68587247	1,7431624532	0,5441458859	3,2034836581	0,0013577575	0,0055632923
CLEC2B	965,614478	-1,0635617858	0,3070404775	-3,4639139254	0,0005323768	0,0025963015
[illegible]	[illegible]	[illegible]	[illegible]	[illegible]	[illegible]	[illegible]
CNPY2	425,1324779	-0,3535930584	0,1244352041	-2,8415677129	0,0044892318	0,0147728531
CNPY4	153,5189955	-0,9082181307	0,2695081399	-3,3702808799	0,0007509160	0,0034343627
CNR2	192,4269009	-1,4044907097	0,3755220210	-3,7401021280	0,0001839455	0,0010864813
CNTLN	17,54502695	2,2020937750	0,4627456055	4,7587567531	0,0000019479	0,0000237703
CNTN4	11,49410411	2,0091671518	0,7002297300	2,8688874192	0,0041191839	0,0138198573
COA4	164,7471045	0,4370017195	0,1560359704	2,8006473020	0,0051000226	0,0163677405
COA5	242,9048089	-0,4591337215	0,1633485439	-2,8107610306	0,0049424480	0,0159494964
COA7	81,14936717	0,6607206729	0,2113548017	3,1261209473	0,0017712869	0,0069448067
COASY	601,6917789	-0,4265182387	0,1083824775	-3,9353062271	0,0000830907	0,0005615619
COBLL1	38,66338153	4,4098045966	0,7221259707	6,1050353757	0,0000000010	0,0000000335
COG4	572,2888021	-0,5057065540	0,2084265826	-2,4263054541	0,0152534260	0,0400607051
COG8	288,6843692	-0,6992547623	0,1909704104	-3,6615869494	0,0002506578	0,0014040173
COL12A1	45,12119128	5,9802155975	0,9652876575	6,1952678571	0,0000000006	0,0000000201
COL13A1	21,43513251	-2,2621983007	0,9445403093	-2,3950256985	0,0166192011	0,0427714046
COL16A1	16,48645446	3,8779872294	0,7525838234	5,1528674036	0,0000002565	0,0000040954
COL18A1	342,1204811	3,4859132870	0,7298386073	4,7762796488	0,0000017857	0,0000220003
COL1A1	1462,200461	5,5879490481	1,0562412972	5,2903143761	0,0000001221	0,0000021484
COL1A2	2016,062162	6,5193518249	1,1554306995	5,6423651613	0,0000000168	0,0000003866
COL27A1	34,58272057	3,2542543867	0,8748875036	3,7196260928	0,0001995179	0,0011650808
COL3A1	2477,857935	7,0832279861	1,4045861582	5,0429287979	0,0000004585	0,0000068262
COL4A2	65,64056294	7,1993980247	0,9287438594	7,7527584123	0,0000000000	0,0000000000
COL4A4	40,20123928	1,4609626072	0,3671355905	3,9790844171	0,0000691812	0,0004843860
COL5A1	63,17249516	7,2017450658	0,9282898782	7,7580777842	0,0000000000	0,0000000000
COL5A2	95,23552889	5,0570168419	0,7913639351	6,3902543664	0,0000000002	0,0000000066
COL6A1	197,1838023	3,5809663016	0,7699140311	4,6511248751	0,0000033013	0,0000376252
COL8A1	103,8857471	4,5327846872	0,7553632948	6,0008608308	0,0000000020	0,0000000586
COL8A2	14,20181389	4,9621744477	0,7878405316	6,2984503188	0,0000000003	0,0000000111
COL9A2	129,2153458	0,9337855589	0,3568981967	2,6163819219	0,0088864492	0,0258284617
COLEC12	286,8247392	3,6084132466	0,6072689427	5,9420251076	0,0000000028	0,0000000811
COLGALT1	808,0379186	0,9675590192	0,3179081896	3,0435171249	0,0023383013	0,0087039645
COLGALT2	274,2669962	-1,7489954182	0,2622302824	-6,6696899709	0,0000000000	0,0000000013
COMMD10	193,2545296	0,7879107023	0,1772400832	4,4454428334	0,0000087711	0,0000856742
COMMD3	420,815755	-0,8593995314	0,1622946340	-5,2953046566	0,0000001188	0,0000020961
COMMD5	307,0176178	-0,8240532455	0,1645388848	-5,0082431424	0,0000005493	0,0000078984
COMT	569,1350767	0,6186212775	0,2479038804	2,4954178688	0,0125808876	0,0342305937
COPA	2282,259781	0,3168247031	0,1108182097	2,8589588660	0,0042503389	0,0141460033
COPG1	1652,388656	0,3923700782	0,1328763631	2,9529858792	0,0031480802	0,0111244841
COPRS	108,409478	0,6917328487	0,2502900511	2,7637389042	0,0057144995	0,0179712998
COPS2	271,6743435	0,8676197124	0,3048735125	2,8458349986	0,0044295140	0,0146231701
COPS5	747,980202	-0,3893926604	0,1318886544	-2,9524348575	0,0031527858	0,0111381883
COPZ2	57,07162636	-1,0624745761	0,4135748246	-2,5690893394	0,0102174775	0,0288740809
COQ10A	163,6450529	-0,8525602523	0,2392239172	-3,5638587576	0,0003654426	0,0019102578
COQ2	96,83141364	1,0339029726	0,2795679502	3,6978594006	0,0002174253	0,0012511945
COQ8A	556,2191802	-0,8033802531	0,2535215458	-3,1687585486	0,0015309152	0,0061419130
COQ8B	345,755353	-0,9078274455	0,1489659509	-6,0941942761	0,0000000011	0,0000000355
COQ9	298,7844127	-0,4555420697	0,1186587161	-3,8390948811	0,0001234887	0,0007807224
CORO1B	377,5074567	1,4999476494	0,3735613824	4,0152642109	0,0000593792	0,0004285493
CORO2A	192,5771609	0,9633884979	0,3163036483	3,0457710597	0,0023208448	0,0086533437
CORO6	25,12058680	-0,9428970624	0,3945132139	-2,3890126597	0,0168937202	0,0433793812
COTL1	2087,557252	2,4245757633	0,3915529226	6,1922044940	0,0000000006	0,0000000205
COX1	58275,22499	0,8407071752	0,3130054288	2,6859188303	0,0072330658	0,0218583443
COX10-DT	128,2297492	-0,7525240174	0,2119267887	-3,5508706660	0,0003839590	0,0019861986
COX16	158,2805674	0,5607853853	0,1615313457	3,4716814827	0,0005172096	0,0025361170
COX19	383,4733032	-0,6502441104	0,1909219737	-3,4058107498	0,0006596789	0,0030830927
COX20	255,8748008	0,5931924457	0,1993805744	2,9751767317	0,0029281979	0,0104629469
COX3	27395,18036	0,8517283071	0,3059394380	2,7839768314	0,0053696870	0,0170929703
COX6B1	1648,044397	0,2626301230	0,1016272368	2,5842493736	0,0097591204	0,0278064682
COX6A	643,418178	0,4736122685	0,1163513887	4,0705335207	0,0000469056	0,0003505548
COX6C	681,2961593	0,7081574920	0,2662497223	2,6597492235	0,0078198852	0,0233279376
COX7A2	720,7567759	0,2762899531	0,1133867528	2,4367028920	0,0148218068	0,0391232914
COX7B	511,9608125	0,4065595117	0,1500803732	2,7088810860	0,0067508697	0,0206239641
COX7C	801,8315293	0,6708580836	0,2060371034	3,2560061872	0,0011299129	0,0047750793
COX8A	444,5117771	0,5422570151	0,1960052395	2,7221021719	0,0064868084	0,0199538681
CP	199,4362438	6,7277641548	0,8766928778	7,6740262488	0,0000000000	0,0000000000
CPAMD8	65,76547284	2,1304934468	0,7308707656	2,9150070689	0,0035568049	0,0122755389
CPE	58,38589002	6,7573449435	0,8842649800	7,6417647385	0,0000000000	0,0000000000
CPEB2	144,9771424	0,9587356876	0,2809581445	3,4123790549	0,0006439850	0,0030286644
CPEB4	211,2112773	0,8002719490	0,3005640571	2,6625617152	0,0077548348	0,0231801190
CPM	188,2586893	2,4350088278	0,4894562688	4,9749254135	0,0000006527	0,0000091967
CPNE1	1656,536813	-0,5346357607	0,1993622183	-2,6817305974	0,0073242416	0,0220744049
CPNE2	119,9361069	2,8142800117	0,3886669691	7,2408520293	0,0000000000	0,0000000000
CPNE8	48,06178383	1,2024560546	0,4348664263	2,7651170151	0,0056902380	0,0179080207
CPPED1	208,2610601	1,0947566275	0,3351344372	3,2666193502	0,0010883992	0,0046360294
CPSF3	402,8168252	0,3467347521	0,1034272442	3,3524508439	0,0008009946	0,0036191299
CPSF4	201,5582496	-0,3714308279	0,1316269394	-2,8218450541	0,0047748241	0,0155156525
CPVL	696,1527279	4,0835094168	1,0131878497	4,0303576658	0,0000556920	0,0004071781
CPXM1	92,8026478	3,0981525731	0,6610227973	4,6869072982	0,0000027726	0,0000323512
CR1	125,6426126	2,9934295670	0,8774307891	3,4115848273	0,0006458540	0,0030332623
CRABP2	84,78597837	5,3666654684	0,8840755057	6,0703700459	0,0000000013	0,0000000400
CRACR2B	134,5549808	2,0306670717	0,3844212845	5,2824600046	0,0000001275	0,0000022346
CRADD	87,21360142	-0,8921419350	0,2128223600	-4,1919570234	0,0000276558	0,0002236757
CRBN	569,2467629	-0,6061071099	0,1405604880	-4,3090075657	0,0000163989	0,0001451209
CREB1	508,9893296	-0,5012025808	0,1189867308	-4,2122565216	0,0000252832	0,0002076340
[illegible]	[illegible]	[illegible]	[illegible]	[illegible]	[illegible]	[illegible]
CST7	3092,312872	-1,1185225564	0,3196103431	-3,4996444285	0,0004658791	0,0023250772
CSTF1	300,6179171	-0,4544303540	0,1626912169	-2,7932076648	0,0052188177	0,0166655482
CSTF3	196,6447721	0,2620200054	0,1118059478	2,3435265337	0,0191024025	0,0479162040
CTBP1	1111,654802	-0,4568905193	0,1834938574	-2,4899499407	0,0127761089	0,0346357353
CTC1	1008,254436	-0,5672870401	0,2007387748	-2,8259963266	0,0047123805	0,0153650039
CTCF	695,272382	-0,4599214193	0,1508906381	-3,0480447627	0,0023033561	0,0086072109
CTDP1	366,0843307	-0,7610789355	0,1971939005	-3,8595507081	0,0001135957	0,0007300106
CTDSPL	20,38284109	2,8863145821	1,0025201057	2,8790590490	0,0039886364	0,0134913536
CTIF	42,53147102	1,2039506571	0,4234651841	2,8430824248	0,0044678112	0,0147177531
CTNNA1	1064,414202	2,1643671985	0,5435610132	3,9818293547	0,0000683869	0,0004796703
CTNNAL1	92,34223646	1,9163870527	0,5527603896	3,4669399051	0,0005264196	0,0025727767
CTNNBIP1	168,3308446	0,7208691371	0,2803843748	2,5710032434	0,0101404377	0,0287227045
CTNND1	398,3442518	3,8420047788	0,5528032307	6,9500403854	0,0000000000	0,0000000002
CTR9	819,1451191	-0,4348094798	0,1601689753	-2,7150499277	0,0066265779	0,0202938302
CTSA	2232,925679	0,6961129281	0,2245714935	3,0982589300	0,0019459559	0,0075028835
CTSB	25290,64106	2,4728917037	0,5647573009	4,3786803634	0,0000119400	0,0001104554
CTSF	571,9879063	-1,2160060588	0,3320743645	-3,6618496361	0,0002504018	0,0014038698
CTSH	718,3192689	1,9045615714	0,4693752540	4,0576522840	0,0000495685	0,0003676522
CTSK	90,16281643	1,0232821933	0,3461296886	2,9563548775	0,0031129869	0,0110207268
CTSL	4790,272002	2,4302295176	0,8630527439	2,8158528374	0,0048647945	0,0157290737
CTSW	4641,360056	-0,7954091629	0,3352835559	-2,3723476711	0,0176754499	0,0449568220
CTSZ	491,9515016	1,6655710120	0,3188040585	5,2244347824	0,0000001747	0,0000029428
CTTN	306,2508838	7,4263730622	1,2900299821	5,7567445450	0,0000000086	0,0000002133
CTTNBP2NL	95,18306674	4,4318158078	0,7961609972	5,5664819344	0,0000000260	0,0000005607
CTU1	37,3850946	-0,9907238965	0,2327669230	-4,2562915886	0,0000207846	0,0001771755
CUEDC1	64,49210273	4,7445831814	0,7808641312	6,0760675148	0,0000000012	0,0000000390
CUL5	264,8416221	0,4620833647	0,1400953425	3,2983492280	0,0009725509	0,0042364193
CUL7	298,4945025	0,8434707476	0,2422386393	3,4805458589	0,0005003932	0,0024644317
CUL9	431,3562986	-0,4273841149	0,1807248803	-2,3648327462	0,0180382151	0,0456806738
CUTC	295,2557959	-0,4648418235	0,1918460396	-2,4229865301	0,0153934974	0,0403577085
CUX1	528,0757606	0,7859645497	0,1757940096	4,4709251977	0,0000077882	0,0000777087
CWF19L2	111,2656046	-0,4501802906	0,1880659749	-2,3937359786	0,0166777492	0,0428974782
CX3CR1	1243,089536	-2,4291065621	0,4884122500	-4,9734759150	0,0000006576	0,0000092581
CXADR	279,098519	8,5179606015	0,9719817544	8,7634984536	0,0000000000	0,0000000000
CXCL16	620,4586874	3,3685321282	0,4985182202	6,7570757385	0,0000000000	0,0000000008
CXCL2	110,126422	3,0329150865	0,8623081451	3,5172056577	0,0004361157	0,0022068112
CXCR2	123,9010358	-1,5673394372	0,4517292817	-3,4696423699	0,0005211518	0,0025517290
CXCR3	1131,555841	-1,2481855668	0,5276142539	-2,3657157119	0,0179952567	0,0455978207
CXCR5	39,47657881	-2,0258339150	0,6514412310	-3,1097723303	0,0018723160	0,0072710251
CXXC1	841,3510368	-0,7290584796	0,1355077559	-5,3801974276	0,0000000744	0,0000013968
CXXC5	888,7787491	-0,9773306802	0,2432107065	-4,0184525353	0,0000585816	0,0004234739
CYB561	319,8298834	1,0300635219	0,2996237854	3,4378563115	0,0005863387	0,0028075843
CYB561D1	283,9268022	-0,8278718564	0,1836089945	-4,5088850832	0,0000065169	0,0000666548
CYB5B	559,5763137	-0,5236951453	0,1991257298	-2,6349503050	0,0084661615	0,0253029356
CYB5R1	298,2839112	0,5492214205	0,2075258908	2,6465199986	0,0081324696	0,0240736840
CYB5R2	41,77377284	4,8707101638	0,8448285701	5,7653236837	0,0000000082	0,0000002043
CYBB	1008,917948	2,0934791774	0,6449693571	3,2457526761	0,0011714056	0,0049180035
CYBRD1	259,1478767	1,3128589046	0,4343680467	3,0224573713	0,0025073139	0,0092258283
CYCS	885,3283678	0,8851603338	0,2625554902	3,3713266972	0,0007480709	0,0034236770
CYFIP1	709,2814514	2,8701047573	0,4423518805	6,4882842906	0,0000000001	0,0000000037
CYFIP2	3363,871832	-0,8364113646	0,2925143307	-2,8593859403	0,0042446203	0,0141417300
CYLD	2111,576539	-0,9141921339	0,3120823291	-2,9293300153	0,0033969355	0,0118326707
CYP1B1-AS1	35,23261462	1,6503346226	0,7044011625	2,3428802588	0,0191350104	0,0479890516
CYP2S1	89,42502888	5,7244742897	0,8450970283	6,7737479817	0,0000000000	0,0000000007
CYR1A	592,8278062	-0,5835548605	0,2276294074	-2,5636180625	0,0103587464	0,0291997333

[illegible]	[illegible]	[illegible]	[illegible]	[illegible]	[illegible]	[illegible]
DNMT1	1047,750508	-0,4719326176	0,1426771843	-3,3076950588	0,0009406718	0,0041215632
DNMT3B	28,84200566	2,0148545215	0,5528299558	3,6446189290	0,0002677883	0,0014875899
DNPH1	305,228387	-1,6995900695	0,4015090389	-4,2330057475	0,0000230589	0,0001934783
DOCK1	55,65292844	6,0132219806	0,8846899421	6,7969824169	0,0000000000	0,0000000006
DOCK4	69,08284817	2,1711006533	0,7217738800	3,0080066812	0,0026296736	0,0095922510
DOCK6	38,07734265	3,9144050812	0,7420177623	5,2753523626	0,0000001325	0,0000023192
DOCK7	134,1263352	2,0721464107	0,2880972536	7,1925340006	0,0000000000	0,0000000001
DOCK9	363,8584467	0,8034063449	0,3137030443	2,5610409571	0,0104359052	0,0293680294
DOK1	250,4231028	-1,1560124392	0,2684232828	-4,3066772275	0,0000165725	0,0001464649
DOK3	94,91582583	1,8089421742	0,5530201978	3,2710255087	0,0010715824	0,0045745321
DOK7	91,9246077	2,4112510140	0,7277242620	3,3134129776	0,0009216478	0,0040580241
DOLK	154,4931198	-0,8735752166	0,2444858603	-3,5731114080	0,0003527646	0,0018854949
DONSON	94,71787546	0,4139652108	0,1714292406	2,4147876372	0,0157443862	0,0410696751
DPCD	55,97379405	2,3725632214	0,4633408051	5,1205574716	0,0000003046	0,0000047623
DPEP2	481,9658874	-1,3445587469	0,3707966811	-3,6261374664	0,0002876922	0,0015743155
DPF2	727,1087547	-1,0264817769	0,1688481683	-6,0793184019	0,0000000012	0,0000000383
DPH6-DT	62,03879603	-0,7874549206	0,3246363382	-2,4256524237	0,0152808974	0,0401171989
DPH7	529,7352258	-0,9152048754	0,2547437262	-3,5926493231	0,0003273330	0,0017483737
DPP4	226,3488756	3,0586212200	0,4343209176	7,0423069586	0,0000000000	0,0000000001
DPY19L3	160,8175452	0,4646702581	0,1783760962	2,6050029570	0,0091873467	0,0265368426
DPY19L4	148,9671596	1,1986011057	0,3237089964	3,7027117533	0,0002133072	0,0012311813
DPYSL3	96,76141466	6,2329480609	0,9262622881	6,7290314430	0,0000000000	0,0000000009
DRAM1	362,8284238	2,2020975727	0,3567313829	6,1729852726	0,0000000007	0,0000000228
DRAM2	368,3250327	0,5386197667	0,1981683751	2,7179768134	0,0065682441	0,0201550072
DRG2	416,3786307	-0,5775134618	0,1958250112	-2,9491251306	0,0031867492	0,0112228268
DROSHA	355,344362	0,6281166573	0,2295149597	2,7367133627	0,0062056338	0,0192106327
DSC2	176,3963072	6,2709936122	0,8198194614	7,6492373108	0,0000000000	0,0000000000
DSC3	43,65670479	5,6939279270	0,7571680849	7,5200216705	0,0000000000	0,0000000000
DSCC1	13,79090059	1,7127137724	0,6717576748	2,5496006024	0,0107846391	0,0301790523
DSE	297,5769327	2,9259416815	0,4731609290	6,1836092547	0,0000000006	0,0000000215
DSG2	448,2581336	6,5795226491	0,7986521912	8,2403464120	0,0000000000	0,0000000000
DSP	317,6474452	7,3917048915	1,2912302538	5,7245443792	0,0000000104	0,0000002524
DST	182,6831094	2,3026491456	0,3836625178	6,0017568538	0,0000000020	0,0000000584
DSTYK	365,7685495	-0,7574125188	0,2260577960	-3,3505259818	0,0008065824	0,0036408683
DTD1	192,7031854	0,6657088020	0,2502560422	2,6601113254	0,0078114828	0,0233183761
DTD2	56,68210279	0,6198287908	0,2411309914	2,5705065420	0,0101549909	0,0287457786
DTHD1	250,5676862	-1,7623946233	0,3931578828	-4,4826632136	0,0000073717	0,0000743629
DTNBP1	286,3529813	-0,5766881240	0,2087974311	-2,7619502838	0,0057457227	0,0180273723
DTX4	61,62627556	3,1281554926	0,7903285476	3,9580444136	0,0000755659	0,0005207973
DUBR	12,76531998	1,5903590012	0,6681072895	2,3803949248	0,0172940924	0,0441956491
DUS1L	1100,444597	-0,5172932813	0,1193772800	-4,3332650177	0,0000146914	0,0001321303
DUSP1	6796,992996	3,1342764851	0,4590465324	6,8277968872	0,0000000000	0,0000000005
DUSP11	337,4106846	-0,8123972396	0,1620098486	-5,0144929235	0,0000005317	0,0000078876
DUSP18	136,816427	-0,6397469095	0,2529389671	-2,5292737591	0,0114298837	0,0316100748
DUSP19	15,96158566	-1,1403211244	0,3709112386	-3,0743773867	0,0021094241	0,0080047501
DUSP22	416,88287	-0,5567240486	0,1690755782	-3,2927525943	0,0009921173	0,0043049321
DUSP28	182,2725532	-0,5099280549	0,2140013423	-2,3828170859	0,0171807287	0,0439978326
DUSP3	372,8050665	1,6501435527	0,3043577866	5,4217228053	0,0000000590	0,0000011379
DUSP5	369,3461596	2,8858465763	0,7098734759	4,0652970903	0,0000479713	0,0003577252
DUSP6	889,292748	3,2143588577	0,4598962292	6,9893120548	0,0000000000	0,0000000002
DUXAP10	45,94910994	2,1228012658	0,7414705293	2,8629610777	0,0041970212	0,0140108891
DUXAP8	32,86051309	2,0298707565	0,7808448048	2,5995828414	0,0093337147	0,0268329346
DVL2	322,8249046	-0,5603511353	0,1919701106	-2,9189499014	0,0035121266	0,0121650089
DYNC1LI2	544,6283464	0,4167155042	0,1595984896	2,6110341101	0,0090271529	0,0261636704
DYNC2LI1	68,77174589	1,2337683110	0,3458424881	3,5674283909	0,0003605019	0,0018907094
DYNLT3	279,8169099	0,7224831283	0,2881600964	2,5072282295	0,0121682115	0,0332826918
DYRK1B	194,4327382	-0,8362449488	0,3208677733	-2,6061976974	0,0091553604	0,0264571716
DYRK3	25,25795146	2,7707174374	0,5526255649	5,0137337345	0,0000005338	0,0000077015
E2F4	690,8223254	-0,4703289117	0,1934077214	-2,4318000761	0,0150239975	0,0395662310
E4F1	414,9031537	-0,5437749273	0,1687930172	-3,2215487121	0,0012749979	0,0052739617
EAPP	502,603793	-0,5499782257	0,1639433376	-3,3546948171	0,0007945546	0,0035984874
EARS2	149,6564061	0,7633842523	0,2795298555	2,7309578485	0,0063150548	0,0195134615
EBAG9	128,5334673	0,5996793558	0,1705048016	3,5150203862	0,0004397202	0,0022217096
EBI3	15,80642872	-1,7888928801	0,7059906783	-2,5338760625	0,0112808623	0,0312657691
EBNA1BP2	258,6031854	0,6640203377	0,1572280317	4,2232948574	0,0000240757	0,0001994176
EBPL	102,4794331	0,9009956429	0,3788147703	2,3784596419	0,0173851399	0,0443861880
ECE1	621,9032719	0,8845768050	0,3244542910	2,7263579385	0,0064037512	0,0197512583
ECHDC3	29,33980718	1,8376327607	0,7175385802	2,5606660314	0,0104471731	0,0293935931
ECHS1	572,1479858	0,4190271739	0,1361862357	3,0768864974	0,0020918897	0,0079516620
ECM1	32,85184495	2,7107596868	0,6784778289	3,9953548538	0,0000645974	0,0004576902
ECPAS	635,6023001	0,6083998293	0,1588173476	3,8308146968	0,0001277197	0,0008018222
ECT2	118,9123391	1,5988787889	0,4085840905	3,9132184143	0,0000910741	0,0006068600
EDARADD	60,04400881	-1,8603680375	0,4938047429	-3,7674163004	0,0001649458	0,0009915903
EDC3	389,6494885	-0,6184258085	0,1797747934	-3,4400028880	0,0005817080	0,0027913688
EDC4	897,5166503	-0,6204302469	0,1988224312	-3,1205243954	0,0018052934	0,0070473462
EDEM1	657,0420383	0,4430444376	0,1432289305	3,0908854428	0,0019957405	0,0076575648
EDEM3	287,2318021	0,7263898526	0,2037916866	3,5643733305	0,0003647265	0,0019079748
EED	216,8554735	-0,4473745451	0,1802640799	-2,4817731039	0,0130730489	0,0353024412
EEF1A1	41818,59215	0,4790362385	0,1550128008	3,0903011619	0,0019995364	0,0076698229
EEF1AKMT2	117,6949361	0,4224271790	0,1457884282	2,8975357237	0,0037610690	0,0129078973
EEF1B2	1378,128838	0,9438934511	0,1467908076	6,4301945492	0,0000000001	0,0000000052
EEF1G	4374,960865	0,5504018629	0,1462675363	3,7629803349	0,0001679004	0,0010064755
EEF2	4848,57082	0,7305494364	0,2444553796	2,9884776416	0,0028037101	0,0101038657
EEFSEC	168,3262646	-0,4606876282	0,1553685079	-2,9651287403	0,0030255629	0,0107650215
EEPD1	44,5411486	2,2670096738	0,6337676733	3,5770356701	0,0003475128	0,0018378588
EFCAB11	43,63939065	0,7002647880	0,2767065801	2,5307124529	0,0113831125	0,0315001426
EFEMP1	388,8269572	9,1864788627	1,1211375379	8,1938910727	0,0000000000	0,0000000000
EFEMP2	151,9653327	1,3020967411	0,2808027245	4,6370516656	0,0000035341	0,0000395096
EFHC2	32,30188275	-1,0787147922	0,3046754140	-3,5405377094	0,0003993126	0,0020522135
EFHD2	4607,85428	-0,7496241244	0,2344351025	-3,1975762858	0,0013858775	0,0056526625
EFNA1	152,4551809	3,1732878874	0,7163481010	4,4298126805	0,0000094315	0,0000907954
EFNB1	111,8343972	1,5175692360	0,4731043780	3,2076837727	0,0013380854	0,0054943958
EFNB2	212,3612574	6,4146869912	1,1262141504	5,6957968332	0,0000000123	0,0000002941
EFR3A	753,189337	0,5054183853	0,1952119027	2,5890758571	0,0096233949	0,0275187794
EGFL8	50,6616067	-0,8942986521	0,1539140137	-5,8103783437	0,0000000062	0,0000001632
EGFR	103,8888921	9,8207240076	1,3928575289	7,0507742564	0,0000000000	0,0000000001
EGLN2	766,587861	-0,7137105881	0,1575706284	-4,5294646293	0,0000059133	0,0000614147
EGLN3	126,7232133	3,7512710784	0,6711476595	5,5893379428	0,0000000228	0,0000005011
EGR1	1113,961328	4,9438629840	0,7152398909	6,9121745681	0,0000000000	0,0000000003
EGR3	54,33309538	2,2402613375	0,6495336445	3,4490304796	0,0005626032	0,0027190804
EHBP1	128,99726	0,6771317802	0,1657068170	4,0863242229	0,0000438261	0,0003314043
EHBP1L1	968,1297506	0,7253938770	0,3042384511	2,3842928799	0,0171119310	0,0438550251
EHD2	112,3453558	5,9968767071	0,9377288140	6,3950326284	0,0000000002	0,0000000064
EHD4	321,0616225	1,2609924915	0,3024687144	4,1689670087	0,0000305956	0,0002435342
EHHADH	41,8988656	1,0156933384	0,4017508321	2,5281673547	0,0114659682	0,0316838293
EHMT1	633,3874798	-0,5010673531	0,1456802971	-3,4394988032	0,0005827902	0,0027955653
EHMT2	637,6258807	-0,3931489307	0,1264462857	-3,1092169174	0,0018758394	0,0072784084
EI24	526,9405257	0,6604091026	0,1612568856	4,0953854478	0,0000421457	0,0003217777
EID2B	37,65134808	-0,8725290096	0,3209101626	-2,7189198457	0,0065495477	0,0201087848
EID3	45,03701963	-0,7322824990	0,2918342324	-2,5092404073	0,0120991109	0,0331543226
EIF1	8877,713823	1,2590765141	0,2295119451	5,4858866419	0,0000000411	0,0000008377
EIF1AX	461,5631258	0,8748128962	0,2836765565	3,0838392395	0,0020434798	0,0078073343
EIF2A	421,8527766	0,8756166318	0,1882308535	4,6492520697	0,0000033298	0,0000377899
EIF2AK2	540,1738633	0,5753362826	0,1701489614	3,3813681721	0,0007212583	0,0033224288
EIF2AK3-DT	26,8082994	1,3717122560	0,5867176469	2,3379427281	0,0193902193	0,0485025743
EIF2B3	129,8807824	0,4460952435	0,1548051601	2,8816561809	0,0039559116	0,0134021066
EIF2B4	505,862003	-0,4009867778	0,1704688002	-2,3522590489	0,0186597747	0,0469810264
EIF2S2	365,8517842	0,8372640667	0,2798469476	2,9918642101	0,0027727957	0,0100165942
EIF2S3	740,408132	0,7649815154	0,2361614111	3,2392316412	0,0011985219	0,0050084151
EIF3C	2191,779503	0,4867128830	0,1838798903	2,6469070769	0,0081231571	0,0240651215
EIF3D	1031,438168	0,4104424319	0,1302477660	3,1512425452	0,0016257586	0,0064551168
EIF3E	1040,994824	1,1630937913	0,2602259400	4,4695526175	0,0000078383	0,0000781454
EIF3H	959,495608	0,8779312444	0,2549059294	3,4441381829	0,0005728828	0,0027598436
EIF3I	1008,793909	0,3582968700	0,1236681540	2,8972210004	0,0037649444	0,0129173606
EIF3J	232,2509604	0,5069505975	0,2098965352	2,4152404282	0,0157248261	0,0410382274
EIF3J-DT	118,3769677	-0,8237782677	0,2549933099	-3,2305877676	0,0012353597	0,0051363622
EIF3K	914,8627227	0,6383784289	0,2148107760	2,9718175260	0,0029604258	0,0105583812
EIF3L	1271,3429	0,4200777186	0,1273824266	3,2977682234	0,0009745654	0,0042424485
EIF3M	826,4060452	0,6292381039	0,1215876906	5,1751793342	0,0000002277	0,0000036969
EIF4A1	3266,580349	1,0484223087	0,2523926790	4,1539321209	0,0000326809	0,0002578945
EIF4A2	3588,196191	0,6869256022	0,2750093809	2,4974624499	0,0125085712	0,0340544839
EIF4A3	946,5728852	0,8430223471	0,2609583152	3,2304866251	0,0012357969	0,0051363622
EIF4EBP3	37,24997182	1,5485471095	0,4924026191	3,1448799202	0,0016615501	0,0065758497
EIF4ENIF1	271,3754328	-0,4048424908	0,1225982874	-3,3021822269	0,0009594325	0,0041901237
EIF4G1	1232,993235	0,7499684066	0,1641158148	4,5697509881	0,0000048830	0,0000525297
EIF4G2	6328,421692	0,6853393175	0,1367746593	5,0107185119	0,0000005423	0,0000078113
EIF5	2238,625872	0,5163758993	0,1169952873	4,4136478597	0,0000101643	0,0000967217
ELF2	481,1112846	-0,3983815597	0,1241927379	-3,2077685572	0,0013376910	0,0054943958
ELF3	740,8259887	7,4684410242	1,1667718566	6,4009437508	0,0000000002	0,0000000062
ELL	392,4487944	-0,9005152562	0,2113440633	-4,2608973350	0,0000203608	0,0001742328
ELL2	180,889148	1,2009296202	0,3025451591	3,9694226927	0,0000720470	0,0005016825
ELMO2	1033,712982	-0,7116060390	0,2010774725	-3,5389645097	0,0004016999	0,0020620039
ELMO3	45,08625319	1,6777816839	0,6847032863	2,4503777292	0,0142706421	0,0379460377
ELMOD3	308,3289397	-0,9827796858	0,1852062255	-5,3064074570	0,0000001118	0,0000020066
ELOC	258,0877925	0,5068829189	0,1754317576	2,8893452701	0,0038604494	0,0131581791
ELOF1	391,1237565	-0,3691144160	0,1246912529	-2,9602270191	0,0030741242	0,0109060917
ELOVL5	758,1696403	0,6642764460	0,2311654772	2,8735671046	0,0040582630	0,0136663048
ELP5	370,4287591	-0,5910874445	0,1830411764	-3,2292584276	0,0012411125	0,0051543889
EMC6	158,2859459	-0,5636180266	0,1869406311	-3,0149573327	0,0025701541	0,0094082877
EMC9	97,43454449	-0,8384362426	0,1979728187	-4,2351078705	0,0000228442	0,0001918628
EMD	311,1578917	0,8732582333	0,3256814158	2,6813265691	0,0073330914	0,0220911841
EMID1	15,04428061	1,3959053175	0,5296884844	2,6353892118	0,0084040856	0,0246930783
EMILIN1	47,68789195	1,7622737604	0,5508104855	3,1994194137	0,0013770468	0,0056320419
EMILIN2	448,131233	1,6193471283	0,4590840810	3,5273432372	0,0004197522	0,0021344409
EML1	22,29687499	2,6470568893	0,6153302282	4,3018476388	0,0000169380	0,0001489126
EML2	547,8075846	-0,5150747572	0,1840105559	-2,7991587478	0,0051235941	0,0164120840
EML2-AS1	22,58667206	-1,3643220996	0,5539673532	-2,4628204023	0,0137848946	0,0369022836
EML3	894,2108634	-0,5949030621	0,1671583324	-3,5588982893	0,0003724137	0,0019376453
EMP1	535,5760686	4,0986171971	0,9608779681	4,2654919074	0,0000199462	0,0001714407
EMP2	131,9672195	3,5398784716	0,6275612092	5,6406903733	0,0000000169	0,0000003897
EMSY	272,3964613	-0,4152431218	0,1480645343	-2,8037985181	0,0050504455	0,0162357288
EMX2OS	84,78274564	3,8183718878	0,9229046077	4,1386872079	0,0000349299	0,0002728707
ENAH	36,9608019	2,2440707709	0,5175643332	4,3358296296	0,0000145211	0,0001309139
ENDOD1	257,603518	1,1209772289	0,4404217240	2,5452360041	0,0109203903	0,0304890043
ENDOG	76,12289758	-0,9859128431	0,2354810426	-4,1868034571	0,0000282911	0,0002285700
ENDOV	89,27083553	-0,6384698019	0,2487531072	-2,5666807103	0,0102677108	0,0290038590
ENG	1008,670237	1,4711575196	0,4512906004	3,2598895663	0,0011145560	0,0047208267
ENO1	8208,584226	1,1576115407	0,2283159699	5,0702171266	0,0000003974	0,0000060299
ENOSF1	242,8320794	0,5836866038	0,1635284343	3,6304793499	0,0002828953	0,0015541924
ENPP2	327,0479184	7,5719511884	0,7769189393	9,7461151474	0,0000000000	0,0000000000
ENPP4	374,8302462	-0,6036258482	0,2009185167	-3,0043166445	0,0026617814	0,0096936088
ENSA	677,6581512	0,7273823076	0,3065158993	2,3730655056	0,0176411350	0,0448950041
ENTPD3-AS1	38,09609665	-1,0326636001	0,3145050758	-3,2834560699	0,0010254263	0,0044181298
ENTPD6	626,9042304	0,5354303572	0,1711408763	3,1285842243	0,0017564469	0,0069043088
ENTPD7	77,18687981	0,5412351519	0,2119972765	2,5530287969	0,0106790667	0,0299084971
ENY2	420,6800771	0,3695583021	0,1512857547	2,4427832140	0,0145744891	0,0385862752
EOMES	3029,323267	-1,9958054024	0,4495352571	-4,4397082786	0,0000090081	0,0000873551
EPAS1	212,5306165	3,9761568804	0,3386380756	11,7415772929	0,0000000000	0,0000000000
EPB41	1268,387816	-0,6666715892	0,2182511037	-3,0546081464	0,0022535475	0,0084563003
EPB41L2	152,8723565	1,8168776275	0,3223432442	5,6364687644	0,0000000174	0,0000003980
EPB41L3	258,4676065	3,1559841404	0,5873608805	5,3731602581	0,0000000774	0,0000014504
EPGS	381,454043	-0,5310549157	0,1750314614	-3,0340540585	0,0024129119	0,0088972025
EPHA2	291,1038844	9,4026541016	1,3892655614	6,7680752763	0,0000000000	0,0000000007
EPHB2	72,07345617	5,6451198502	0,8484909791	6,6531288593	0,0000000000	0,0000000015
EPHB3	32,21265867	7,3768453829	0,9712082359	7,5955334301	0,0000000000	0,0000000000
EPHB4	37,90058554	4,0376249788	0,7986028971	5,0558606707	0,0000004285	0,0000064292
EPHX1	42,98172666	2,6667123802	0,6269601524	4,2534001081	0,0000210549	0,0001793663
EPN1	276,2223165	0,7203519028	0,2470763896	2,9155027885	0,0035511593	0,0122717855
EPN2	79,32679635	1,2617753897	0,5084249623	2,4817337528	0,0130744925	0,0353024412
EPS8	123,2255371	4,0780991190	0,5865225906	6,9530128663	0,0000000000	0,0000000002
EPS8L1	229,7909853	3,6139434614	0,7762482650	4,6555247059	0,0000032316	0,0000368930
EPS8L2	312,2373842	3,2142511774	0,6695068670	4,8009382258	0,0000015792	0,0000199031
EPSTI1	277,4889432	0,8032682683	0,3335966916	2,4079023848	0,0160444703	0,0416821432
ERBB3	175,1786546	3,2930442704	0,7216660902	4,5631134883	0,0000050401	0,0000538280
ERC1	286,2660314	0,4615561694	0,1519705819	3,0371415543	0,0023883327	0,0088534723
ERCC3	774,500866	-0,3632783070	0,1412624385	-2,5716553597	0,0101213592	0,0286748989
ERCC6L	11,07922549	1,9835857771	0,7017791501	2,8265099882	0,0047058276	0,0153440989
ERCC6L2-AS1	90,61935433	-0,8001600193	0,2805952531	-2,8516510003	0,0043492823	0,0144154967
EREG	53,31703449	3,5030834512	0,5932575758	5,9038320134	0,0000000036	0,0000001000
ERGIC1	1098,459783	0,8253548869	0,2730922593	3,0222569805	0,0025089745	0,0092288724
ERGIC3	973,3258731	0,6180231874	0,1918671039	3,2211003082	0,0012769944	0,0052786758
ERI2	42,8577051	1,6640247925	0,4780418170	3,4809188929	0,0004996968	0,0024629764
ERICH6-AS1	75,65178576	-0,6601464081	0,2492221978	-2,6488266852	0,0080771742	0,0239521403
ERLIN1	183,7609176	0,6493158194	0,2136552688	3,0391523000	0,0023724410	0,0088066893
ERLIN2	[illegible]	[illegible]	[illegible]	[illegible]	[illegible]	[illegible]

EXTL3	306,6439973	-0,6486742224	0,1829630478	-3,5453837825	0,0003920418	0,0020210199
EXTL3-AS1	16,08950044	-1,1638312499	0,3757279913	-3,0975367204	0,0019513622	0,0075194203
EZH1	693,4406811	-0,7035446891	0,1387452253	-5,0714875958	0,0000003947	0,0000060014
EZR	4417,342908	1,3537555940	0,3638759272	3,7203769000	0,0001989257	0,0011626312
F13A1	330,354658	3,0843205239	1,2722351369	2,4243321414	0,0153365715	0,0402650048
F2R	1416,233582	-1,1537776169	0,2817583497	-4,0949044572	0,0000422343	0,0003219516
F2RL1	90,47589420	3,8752799451	0,9882323553	3,9214258918	0,0000880265	0,0005891780
F3	241,4972837	7,5654343578	1,2363689743	6,1190847984	0,0000000009	0,0000000310
F8	46,82124016	1,2406166245	0,3137559045	3,9540819072	0,0000768291	0,0005270742
FABP5	1296,514368	2,7463543243	0,6155307942	4,4617659265	0,0000081287	0,0000805687
FADD	181,9080554	-0,7269241934	0,3095321116	-2,3484613264	0,0188511567	0,0473654227
FAH	97,34062294	1,1752210545	0,3141328217	3,7411597045	0,0001831731	0,0010832643
FAM110A	95,96704277	-0,8736500953	0,2267680756	-3,8517670371	0,0001172686	0,0007479864
FAM110B	35,68693792	3,8927513534	0,6071993002	6,4110109352	0,0000000001	0,0000000058
FAM110C	41,9629925	1,7959132606	0,7447740842	2,4113530514	0,0158934551	0,0413943315
FAM111A-DT	61,34768898	-0,9229776527	0,2831015746	-3,2602349672	0,0011131995	0,0047198361
FAM111B	15,60681418	1,7727214938	0,5026405626	3,5268174230	0,0004205867	0,0021368456
FAM114A1	102,6536731	4,3513199680	0,6691258922	6,5029914680	0,0000000001	0,0000000034
FAM117A	506,6026735	-0,7953474797	0,1524915193	-5,2158833588	0,0000001831	0,0000030599
FAM118A	251,872613	-0,9840861409	0,3830977406	-2,5687599704	0,0102062125	0,0288607071
FAM118B	123,2892529	-0,5753900217	0,1486722380	-3,8702022824	0,0001087451	0,0007037062
FAM120B	516,9153573	-0,4560488810	0,1325072952	-3,4416888550	0,0005780948	0,0027779920
FAM135A	70,3902214	1,4119388808	0,3267300585	4,3214232797	0,0000155026	0,0001381887
FAM136A	412,0920882	0,3966011439	0,1657046509	2,3934219210	0,0166920337	0,0429178172
FAM13A	102,0480745	0,8903368802	0,3183902325	2,7963699549	0,0051680205	0,0165190110
FAM13B	508,9210853	0,4166973126	0,1542054539	2,7022216271	0,0068877834	0,0209797611
FAM169B1	104,2226263	0,6511090453	0,2611621485	2,4931217982	0,0126625402	0,0343763192
FAM162A	283,0403325	0,8542445854	0,2478703883	3,4463357703	0,0005682438	0,0027414201
FAM171A1	39,38349216	3,6646995273	0,8760558435	4,1831802897	0,0000287459	0,0002321057
FAM174A	59,58940585	0,8094821773	0,3302947719	2,4507871336	0,0142544223	0,0379183900
FAM174B	160,7345677	5,7968834668	1,0879505058	5,3282694716	0,0000000992	0,0000018060
FAM177A1	289,3458485	0,6118748783	0,2487297848	2,4599884230	0,0138937626	0,0371273672
FAM182A	22,46382084	-1,4627539307	0,4894522106	-2,9885484227	0,0028030608	0,0101038657

GPSM2	83,00578620	1,3429453150	0,3557835298	3,7746136066	0,0001602559	0,0009664175
GPX1	1440,975318	2,2736513911	0,3878299099	6,1203407657	0,0000000009	0,0000000308
GPX3	380,8534379	4,6711445242	0,6397503454	7,3015115319	0,0000000000	0,0000000000
GPX8	67,27752812	5,2236371415	0,8003469727	6,5267165024	0,0000000001	0,0000000030
GRAMD1A	551,5132270	0,7309265306	0,2074322744	3,5236875876	0,0004255857	0,0021575834
GRAMD2B	268,5825304	1,9053045829	0,5777897788	3,2975740536	0,0009752395	0,0042426776
GRAP	168,8538176	-1,6634196969	0,4134795171	-4,0229796837	0,0000574665	0,0004174304
GRAP2	332,6969724	-1,2208156670	0,4096941423	-2,9798221185	0,0028841581	0,0103384203
GRB10	31,56539379	2,7441498569	0,5949494356	4,6124065218	0,0000039803	0,0000438502
GRHL1	65,28835334	3,2366004095	0,7058291172	4,5855297419	0,0000045284	0,0000491151
GRIK4	23,71989094	-2,5278654560	0,9167307090	-2,7570424238	0,0058326784	0,0182582992
GRINA	211,7600432	1,8501578245	0,4140310376	4,4686452376	0,0000078717	0,0000783093
GRK2	4169,970371	-1,0140834647	0,2467489434	-4,1097800129	0,0000396036	0,0003051315
GRK3	70,5321155	2,1624946899	0,4664646852	4,6359237013	0,0000035535	0,0000396927
GRK5	151,5969720	-1,1698943067	0,2836767650	-4,1240399325	0,0000372284	0,0002884828
GRN	2756,831345	1,5105275007	0,3569885129	4,2313056190	0,0000232339	0,0001940000
GRTP1	18,94436263	3,1872315100	0,7327535878	4,3496634656	0,0000136347	0,0001240856
GSDMD	997,9046442	-0,6729036600	0,1609815273	-4,1800054419	0,0000291502	0,0002346662
GSDME	87,34632620	1,8374776056	0,6900980791	2,6626325465	0,0077532028	0,0231801190
GSN	1659,588703	1,0467726456	0,2907613469	3,6001093566	0,0003180834	0,0017077791
GSPT1	781,8518439	0,5422542864	0,1149422176	4,7176250626	0,0000023861	0,0000283222
GSS	526,3349728	-0,2476722331	0,0876721946	-2,8220981954	0,0047621229	0,0154901339
GSTCD	36,52826995	0,9747501528	0,2990924845	3,2590258982	0,0011179546	0,0047316714
GSTM2	664,1037270	1,5865598907	0,3615208604	4,3885851394	0,0000114085	0,0001060941
GSTO2	45,17690108	1,6115443659	0,5378609323	2,9962101154	0,0027335801	0,0099015037
GSTP1	1814,00773	0,5733371255	0,2141832001	2,6768528575	0,0074317043	0,0223480439
GTF2F1	474,5229517	0,3802883434	0,1245304129	3,0537788578	0,0022597858	0,0084749840
GTF2H3	140,1497819	0,5452198343	0,1938980538	2,8123880987	0,0049173510	0,0158723127
GTF2IRD1	49,22770187	4,5840458095	0,9024456421	5,0795810800	0,0000003783	0,0000057727
GTF3C1	997,8219127	-0,7580921419	0,2049278287	-3,6993128105	0,0002161840	0,0012451163
GTF3C5	490,6183754	-0,6721719380	0,1766030821	-3,8061166892	0,0001411658	0,0008733046
GTPBP1	553,8256266	-0,3156469833	0,1339992253	-2,3555881198	0,0184934102	0,0465406785
GTSE1	19,36734654	1,8039646992	0,4621864292	3,9031104878	0,0000949643	0,0006285948
GUSBP11	692,3144705	-0,6845602070	0,1811305728	-3,7793741633	0,0001572230	0,0009541127
GVINP1	203,3240403	-1,2326959100	0,5005445279	-2,4627097666	0,0137891476	0,0369063287
GXYLT1	148,3413589	0,5425983418	0,2330752733	2,3279961629	0,0199123074	0,0495781831
GZMK	554,6812745	1,3370755057	0,3586038822	3,7285487656	0,0001925704	0,0011303956
H1-0	414,8437287	5,8850398453	0,7299644782	8,0757800151	0,0000000000	0,0000000000
H1-3	56,27395846	-2,2501109749	0,5570159367	-4,0395809649	0,0000535468	0,0003938468
H1-4	82,32502651	-1,4445934276	0,5084302237	-2,8412796095	0,0044932897	0,0147835932
H19	711,2260527	12,2898827353	1,4245701111	8,6270816864	0,0000000000	0,0000000000
[illegible]	[illegible]	[illegible]	[illegible]	[illegible]	[illegible]	[illegible]

JUNB	5133,73672	1,5759437107	0,4213659281	3,7400833944	0,0001839592	0,0010864813
JUND	4454,769782	1,9701963139	0,3896269130	5,0566227546	0,0000004267	0,0000064107
JUP	826,7196327	6,2144709699	0,9259249422	6,7116357784	0,0000000000	0,0000000010
KANK1	106,8884761	1,6689904203	0,4367380300	3,8214821793	0,0001326520	0,0008281521
KANK2	96,18998759	3,4458791960	0,6094559424	5,6540770527	0,0000000157	0,0000003642
KANK3	80,52602602	-1,1627116847	0,3245488659	-3,5825363583	0,0003402742	0,0018077212
KANSL2	460,4072273	-0,4767679798	0,1767257878	-2,6976312252	0,0069834755	0,0212184153
KAT5	546,8503861	-0,4989662564	0,1514230573	-3,2951808349	0,0009835836	0,0042720247
KAT6A	944,1707855	-0,4730077335	0,1384978189	-3,4152721260	0,0006371832	0,0030008697
KATNAL1	114,3838018	0,8827282866	0,3089840051	2,8568737283	0,0042783600	0,0142189647
KATNB1	291,497953	-1,0000804563	0,2071777900	-4,8271605573	0,0000013849	0,0000177535
KAZN	253,6827296	5,9124790197	0,9664297620	6,1178569332	0,0000000009	0,0000000311
KBTBD11	35,95250202	2,6386413383	0,8272085233	3,1898140117	0,0014236438	0,0057828559
KBTBD3	59,13373771	-0,7889743438	0,2408846435	-3,2753202215	0,0010554227	0,0045170076
KBTBD4	339,8580782	-0,6678785429	0,2132877954	-3,1313490848	0,0017400519	0,0068482745
KBTBD6	83,67139657	-0,8532183402	0,2720584200	-3,1361470238	0,0017118336	0,0067569738
KBTBD7	112,0865468	-0,8167905355	0,2510224294	-3,2528547948	0,0011385046	0,0047978308
KCNC4	46,3763468	1,3890935907	0,4740225249	2,9304379387	0,0033849460	0,0118032848
KCND1	25,27653827	1,0610103160	0,4318413576	2,4569446567	0,0140124272	0,0373998155
KCNE3	59,4691328	1,7090615885	0,6299196033	2,7131424066	0,0066649458	0,0203827695
KCNJ15	29,80327636	1,8705419682	0,6345248473	2,9479412446	0,0031989786	0,0112570583
KCNJ5-AS1	11,98203813	2,6800797490	0,8361020007	3,2054459226	0,0013485338	0,0055322357
KCNK12	15,21038405	-2,1375651535	0,4712934528	-4,5355290649	0,0000057459	0,0000598536
KCNK5	18,99018184	4,8418708134	0,7908896195	6,1220563455	0,0000000009	0,0000000306
KCNMA1	27,95476779	3,1683010097	0,5785188893	5,4756455749	0,0000000436	0,0000008802
KCNMB1	19,29012483	1,1172024488	0,4105374304	2,7213169032	0,0065022394	0,0199908960
KCNN4	111,8092551	1,8419573772	0,6743261864	2,7315524954	0,0063036698	0,0194872290
KCNQ1	48,27231553	3,5026450933	0,8265581679	4,2376268584	0,0000225895	0,0001902718
KCNQ5	63,66062982	0,9594452652	0,3128207150	3,0670771439	0,0021616306	0,0081683178
KCNS1	21,97661324	2,6445776859	0,7105470844	3,7218894341	0,0001977376	0,0011571949
KCTD1	64,20223249	2,5908663539	0,8228105842	3,1488004886	0,0016394209	0,0064978225
KCTD12	162,8994203	4,0611826655	0,8172515862	4,9693175084	0,0000006719	0,0000094303
KCTD13	188,7558028	-0,5028981343	0,1204102515	-4,1765391918	0,0000295978	0,0002374172
KCTD21	101,730392	-1,2850776214	0,3737422132	-3,4384054898	0,0005851508	0,0028038908
KCTD3	157,1257763	3,8482978586	0,5241242113	7,3423394238	0,0000000000	0,0000000000
KCTD7	274,2810388	-0,7845523021	0,1869707751	-4,1961226383	0,0000271523	0,0002204284
KDELR1	328,5484351	1,2562002467	0,3520206528	3,8526155689	0,0001168628	0,0007466167
KDELR2	906,9513066	1,1039849854	0,2108305899	5,2363605592	0,0000001638	0,0000027782
KDELR3	140,7907323	4,7896721691	0,6443811388	7,4329816322	0,0000000000	0,0000000000
KDM2B	380,4746867	-0,3891100208	0,1041359618	-3,7365576113	0,0001865567	0,0010998920
KDM5C	1013,262952	0,4908157902	0,1619723084	3,0302450782	0,0024435537	0,0090300231
KDM6A	688,8292236	0,4563364810	0,1876974881	2,4312338206	0,0150475003	0,0396126254
KDM8	66,11075843	-0,8792082584	0,2294331081	-3,8320901703	0,0001270591	0,0007991664
KDSR	195,1122362	0,8093202138	0,2406977574	3,3623820001	0,0007727035	0,0035125257
KEAP1	626,6501639	-0,6304764339	0,2057344920	-3,0645149855	0,0021802325	0,0082316773
KHDRBS1	1110,505348	0,2757351180	0,1160059018	2,3769058491	0,0174585390	0,0445229129
KHK	73,75678963	-1,5057748236	0,3863602524	-3,8973336782	0,0000972576	0,0006407670
KHNYN	1065,773626	-0,2862194667	0,1188435771	-2,4083713543	0,0160238725	0,0416474518
KIAA0825	23,94798824	1,2625335794	0,3058167134	4,1283995408	0,0000365297	0,0002837217
KIAA1328	120,8778685	0,4380181342	0,1878585515	2,3316255723	0,0197203973	0,0492095936
KIAA1522	139,5719297	3,3995707188	0,8287053702	4,1022670312	0,0000409122	0,0003135979
KIAA2013	963,4155352	-0,8894252680	0,1212713225	-7,3341763723	0,0000000000	0,0000000000
KIAA2026	490,4180435	-0,4975330632	0,1563950568	-3,1812582397	0,0014663685	0,0059299754
KIDINS220	957,1379957	0,3583760047	0,1313953246	2,7274639025	0,0063823240	0,0196896818
KIF11	81,42475363	1,9723646212	0,4410114097	4,4723664240	0,0000077359	0,0000772439
KIF14	14,72058643	1,9451574496	0,5481833676	3,5483700610	0,0003876232	0,0020020753
KIF15	22,38153295	2,0884261795	0,6159207667	3,3907383750	0,0006970461	0,0032307989
KIF18A	26,16254218	1,4272777684	0,3849896558	3,7075743384	0,0002092540	0,0012145575
KIF18B	20,75260953	2,0705714003	0,5482489815	3,7767128142	0,0001589118	0,0009610064
KIF1B	267,8476747	0,9305055140	0,2025287581	4,5941869517	0,0000043444	0,0000473870
KIF20A	91,47585976	5,0650067132	0,6548355044	7,7347771751	0,0000000000	0,0000000000
KIF21B	1353,338043	-0,4641456541	0,1852788806	-2,5051053978	0,0122414909	0,0334491407
KIF23	51,3481914	2,7338312941	0,5370342034	5,0906092701	0,0000003569	0,0000054716
KIF27	52,89153585	-0,8739632953	0,3748892339	-2,3312573856	0,0197397918	0,0492437994
KIF2A	626,0755022	-0,3989110544	0,1324680515	-3,0113755719	0,0026006696	0,0094993252
KIF2C	47,98650043	2,3952402433	0,4572001660	5,2389312633	0,0000001615	0,0000027448
KIF4A	35,97864638	2,7131614364	0,6612384443	4,1031513817	0,0000407560	0,0003129357
KIF5B	1064,604665	0,4203407981	0,1655345930	2,5386886838	0,0111268785	0,0309501539
KIF9	35,09665664	0,9512802627	0,3737951423	2,5449240908	0,0109301496	0,0305101232
KIFC1	66,32984819	2,7990856099	0,6129682179	4,5674318864	0,0000049374	0,0000528537
KIFC3	404,9652091	-1,0199532937	0,3709528191	-2,7495499192	0,0059677172	0,0186024719
KIN	154,8849296	-0,4523500959	0,1411322973	-3,2051493841	0,0013499240	0,0055345050
KIR3DL2	308,4252154	-1,1241670554	0,4514794459	-2,4899628668	0,0127756442	0,0346357353
KIRREL1	65,32731123	8,7075823492	1,0500882820	8,2922385380	0,0000000000	0,0000000000
KIZ	303,4569061	-0,4828710783	0,1833890888	-2,6330414835	0,0084624011	0,0248210762
KLC4	226,681999	-0,5150946726	0,2102652618	-2,4497359683	0,0142961002	0,0379912160
KLF10	1215,211351	1,2677372917	0,3500170878	3,6219097164	0,0002924136	0,0015900611
KLF11	326,7540907	1,0269925761	0,3891736627	2,6389056471	0,0083174126	0,0244811664
KLF12	476,6252796	-0,5791139141	0,2189339018	-2,6451541278	0,0081653715	0,0241526282
KLF13	1689,094843	-0,6459849459	0,2729785874	-2,3660924726	0,0179769536	0,0455772800
KLF4	250,94471	6,1415181596	1,1384596708	5,3945856119	0,0000000687	0,0000013039
KLF5	206,387801	4,9852148344	0,8647730817	5,7764455675	0,0000000076	0,0000001938
KLF6	5829,824669	1,0698727364	0,3096952474	3,4546017262	0,0005511063	0,0026721507
KLHDC3	688,105964	-0,4469182391	0,0930540768	-4,8242834089	0,0000014051	0,0000179801
KLHDC4	659,2773413	-0,5413813581	0,1939538225	-2,7912899633	0,0052498418	0,0167566684
KLHDC7B	51,03517934	-0,8594667395	0,3796372494	-2,5273250742	0,0114935064	0,0317438882
KLHDC8B	81,38878997	1,5935226622	0,3863604938	4,1244451433	0,0000371630	0,0002881413
[illegible]	[illegible]	[illegible]	[illegible]	[illegible]	[illegible]	[illegible]
LDLRAD3	49,73822518	4,4318138967	0,5214309646	8,4993301087	0,0000000000	0,0000000000
LDLRAD4	690,0063709	-1,0708761998	0,4200526109	-2,5493858909	0,0107912819	0,0301913646
LEAP2	13,33146542	-1,5489742506	0,4987449419	-3,1057444808	0,0018980065	0,0073474740
LEPROT	520,289688	0,6154347639	0,2218895613	2,7736084576	0,0055438357	0,0175741943
LEPROTL1	900,0779781	-0,7321628671	0,2369920839	-3,0893979879	0,0020056257	0,0076888983
LEXM	23,43771581	-1,3979772374	0,4463499147	-3,1341273506	0,0017236603	0,0067897184
LFNG	212,838852	-0,7403008849	0,2318447933	-3,1930882485	0,0014075892	0,0057256831
LGALS3	1298,834121	1,6345114137	0,4817950420	3,3925451099	0,0006924653	0,0032147485
LGALS3BP	1790,239807	5,5104828621	0,6148427329	8,9624265972	0,0000000000	0,0000000000
LGALS9	352,1645802	2,5589725237	0,6246404389	4,0967128678	0,0000419058	0,0003204842
LGMN	1782,942715	3,4383823919	0,6339738369	5,4235398876	0,0000000584	0,0000011300
LGR4	82,75912552	4,3409971974	0,8705119853	4,9867173236	0,0000006141	0,0000087014
LHFPL2	439,8526571	1,9257149426	0,6026212794	3,1955641270	0,0013955777	0,0056938536
LIF	328,6658173	7,4083432044	1,0757883315	6,8864256108	0,0000000000	0,0000000004
LILRA2	114,1602372	1,7209467536	0,7112536329	2,4195864336	0,0155377403	0,0406724980
LILRA4	16,46178941	3,4555126234	0,7657782251	4,5124201221	0,0000064092	0,0000658032
LILRA5	67,54774574	3,0971732282	0,8502144776	3,6428140438	0,0002696737	0,0014949784
LILRA6	169,3844672	2,8383540579	0,6876078596	4,1278673856	0,0000366143	0,0002842149
LILRB2	264,8214601	1,7723465074	0,7239760653	2,4480733444	0,0143622416	0,0381519187
LILRB3	193,4115462	1,5940378811	0,5809282799	2,7439448075	0,0060705743	0,0188750129
LILRB4	181,9835434	1,5925597835	0,5845824563	2,7242688629	0,0064444021	0,0198493483
LILRB5	61,41223786	2,4063264647	0,7572564919	3,1776801096	0,0014845333	0,0059891236
LIM2	64,75733551	-2,8163791147	0,4903295900	-5,7438489781	0,0000000093	0,0000002277
LIMA1	201,0122988	1,5402807738	0,4457444509	3,4555242824	0,0005492237	0,0026639824
LIMD2	2036,930579	-1,2489014044	0,2617006390	-4,7722520253	0,0000018218	0,0000223732
LIMK1	582,2637627	-0,5996434051	0,2423182073	-2,4746114282	0,0133381206	0,0358976757
LIMK2	504,1690457	0,6803905189	0,2616994134	2,5998891751	0,0093252784	0,0268258705
LIMS1	462,3033273	0,7633617481	0,2301554273	3,3167227771	0,0009107994	0,0040266289
LIN37	134,4276632	-1,2742558838	0,1661726399	-7,6682652725	0,0000000000	0,0000000000
LIN52	120,7772019	-0,8375770276	0,2220855535	-3,7714160797	0,0001623237	0,0009775739
LIN7B	39,95123675	-0,8914347442	0,3159416098	-2,8215173831	0,0047797047	0,0155210576
LIN7C	221,2760724	0,7872566635	0,2730057439	2,8836633700	0,0039307870	0,0133403845
LINC00299	220,962312	-0,8838762689	0,3526500852	-2,5063832561	0,0121972329	0,0333427863
LINC00324	75,31912087	-1,8601620843	0,4508006103	-4,1263521971	0,0000368563	0,0002858282
LINC00426	168,0127208	0,9264494276	0,3635262738	2,5484443857	0,0108204538	0,0302541147
LINC00507	11,00747364	1,2288190962	0,4648789569	2,6433097860	0,0082099881	0,0242445020
LINC00528	69,28864645	-0,6875365869	0,2707939224	-2,5389660917	0,0111180598	0,0309448067
LINC00612	11,20884770	-1,5841563596	0,4988587690	-3,1749243788	0,0014987555	0,0060286321
LINC00641	163,4820736	0,8633467565	0,3392612759	2,5447835148	0,0109345505	0,0305260793
LINC00654	9,824340887	2,5348205172	0,7524540502	3,3687379537	0,0007551318	0,0034454490
LINC00847	165,2284462	-1,2908138138	0,3779889328	-3,4149513432	0,0006379341	0,0030033552
LINC00852	29,78681851	-0,9198275727	0,3202886122	-2,8718620202	0,0040806109	0,0137278238
LINC00861	553,8872591	-0,9813141946	0,3205912852	-3,0609509360	0,0022062525	0,0083116440
LINC00921	50,75278405	-0,8906029318	0,2827980787	-3,1493874659	0,0016361311	0,0064905171
LINC00954	107,2029701	-1,2415840087	0,3805171556	-3,2628857609	0,0011028397	0,0046956761
LINC00987	39,02761487	-1,3321487766	0,3648480633	-3,6513425596	0,0002609746	0,0014527371
LINC00996	466,3253838	-2,5408019359	0,5904228091	-4,3032900610	0,0000168227	0,0001478959
LINC01003	51,76755632	-0,5876884184	0,2293377448	-2,5625455548	0,0103907955	0,0292778120
LINC01011	28,15629773	-1,4424272953	0,3643068545	-3,9593745683	0,0000751463	0,0005187021
LINC01089	93,6651352	-0,6279255520	0,2444610955	-2,5686113807	0,0102106893	0,0288670191
LINC01138	251,3068898	-1,3162410175	0,1864129883	-7,0608808472	0,0000000000	0,0000000001
LINC01503	49,85207705	2,3638974496	0,5304148729	4,4566852581	0,0000083233	0,0000818943
LINC01801	32,83228874	-2,3250495107	0,4635890346	-5,0153246458	0,0000005294	0,0000076711
LINC01962	25,47366603	-0,8622189343	0,3542910039	-2,4336489631	0,0149474835	0,0394187163
LINC02256	86,74210812	-0,8317044212	0,2682146393	-3,1008812234	0,0019293915	0,0074518250
LINC02361	60,5282872	-0,9625256372	0,4015411452	-2,3970784782	0,0165263837	0,0426221737
LINC02422	32,42225243	-1,2885272001	0,3465132558	-3,7185498867	0,0002003697	0,0011695475
LINC02446	45,04062006	1,7171660250	0,7128938823	2,4087259935	0,0160082117	0,0416450374
LINC02481	158,9759709	-1,3613761002	0,3659381709	-3,7202352979	0,0001990373	0,0011627784
LINC02610	23,00633762	-1,4425737401	0,4773264367	-3,0221953556	0,0025094854	0,0092288724
LINC02908	109,4768392	-1,0747431142	0,3804174662	-2,8251576277	0,0047255885	0,0153936161
LINC02987	72,20821439	-0,9316841790	0,3929937241	-2,3707355155	0,0177527296	0,0451278074
LINGO2	94,96379258	-1,0798138952	0,4512440871	-2,3410899466	0,0192275342	0,0481851817
LIPA	2295,383328	1,2190415472	0,5119261468	2,3812840090	0,0172524049	0,0441058632
LIPC	29,10479704	-2,9367238810	0,5642972051	-5,2042150034	0,0000001948	0,0000032274
LIPG	30,12069916	2,1671658214	0,5851671755	3,7034986103	0,0002126463	0,0012284205
LITAF	3216,800836	-0,8768734588	0,1890467297	-4,6385424987	0,0000035087	0,0000392731
LIX1L	298,0158781	-0,6016521173	0,1836009642	-3,2769184113	0,0010494632	0,0045000874
LMAN1	685,2233343	1,2082208096	0,3281405267	3,6820213982	0,0002313920	0,0013174857
LMBR1L	668,8246527	-0,8927637341	0,2345725409	-3,8059174816	0,0001412795	0,0008723046
LMNA	1562,561096	3,5891964253	0,5220845251	6,8747420247	0,0000000000	0,0000000004
LMNB1	147,4066685	0,9259102291	0,2522501372	3,6706034705	0,0002419785	0,0013667774
LMNTD2-AS1	64,0420468	1,6890187778	0,6724267944	2,5118255132	0,0120109459	0,0329459026
LMO2	43,5963229	3,2862657401	0,8386350888	3,9185884110	0,0000890691	0,0005952679
LMO7	297,7391838	3,8544084817	0,6868273044	5,6119032960	0,0000000200	0,0000004496
LNPEP	1189,674018	-0,6000662646	0,1733162745	-3,4622620764	0,0005356553	0,0026085094
LOC100128288	90,2990871	-0,8986639770	0,3142375516	-2,8598138404	0,0042509423	0,0141460033
LOC100128906	3215,130817	-3,5053487707	1,1374259335	-3,0818269402	0,0020573448	0,0078491569
LOC100129215	11,42182798	-1,1121458993	0,4636872255	-2,3984829388	0,0164631434	0,0424997910
LOC100129434	73,31356852	1,1854015146	0,4872669008	2,4327559285	0,0149843977	0,0394928517
LOC100289230	24,99746682	-0,8613229158	0,3334160304	-2,5833578390	0,0097843781	0,0279489585
LOC100505715	40,0491756	-1,8523783171	0,4017612319	-4,6106472456	0,0000040142	0,0000442512
LOC100507384	13,50109499	-1,3567122581	0,5618350531	-2,4147874906	0,0157443826	0,0410596751
LOC100996756	92,92244881	-1,0080134757	0,4042809855	-2,4933492099	0,0126544300	0,0343681654
LOC101927556	192,7803422	-0,8815887764	0,2827488437	-3,1179253109	0,0018212895	0,0071015518
LOC101928336	22,51383472	-1,3693656652	0,3566114601	-3,8371331775	0,0001244790	0,0007866136
LOC102724428	956,4359875	3,7284900390	1,0034218854	3,7157750453	0,0002025818	0,0011809227
LOC102724951	20,58251351	-1,8605876420	0,7576775841	-2,4556456217	0,0140631762	0,0375129512
LOC105369147	18,06872778	1,2880027861	0,3558951463	3,6190512836	0,0002956850	0,0016118565
LOC105375130	74,76018633	-1,5928608372	0,6409548322	-2,4851374185	0,0129501434	0,0350440148
LOC107985216	64,05603785	2,2504468192	0,7762118721	2,8992687437	0,0037403415	0,0128562299
LOC107985433	37,26698096	-1,8131272411	0,4389340733	-4,1307507238	0,0000361580	0,0002814849
LOC107985563	25,85372165	-2,9374961194	0,5906677899	-4,9731781061	0,0000006586	0,0000092636
LOC107986412	17,32201662	1,6246235506	0,6455882822	2,5165005982	0,0118526699	0,0325984749
LOC124901671	18,83499411	-1,3026637590	0,4056304895	-3,2114542487	0,0013206500	0,0054360699
LOC400499	42,68820615	-0,7463906723	0,2721073965	-2,7430003076	0,0060880628	0,0189250175
LOC728743	157,6926696	-1,2070065766	0,2567371003	-4,7013329018	0,0000025847	0,0000303312
LOC93622	292,7179328	-1,2222736008	0,2094043116	-5,8369075180	0,0000000053	0,0000001420
LONP1	483,6925671	0,4378549254	0,1379706917	3,1735357714	0,0015059434	0,0060525526
LONRF1	120,962467	1,0367181033	0,1893450802	5,4752840799	0,0000000437	0,0000008802
LOX	186,4773448	7,2268612757	0,9463352332	7,6366820361	0,0000000000	0,0000000000
LOXL1	60,61965675	3,0156768094	0,6832686263	4,4136032207	0,0000101664	0,0000967217
LOXL2	57,57541584	1,5624710585	0,4087065850	3,8229852173	0,0001318564	0,0008247151
LOXL3	54,47874454	1,9935621638	0,4557712992	4,3740405927	0,0000121968	0,0001124642
LOXL4	28,77888105	2,9255361251	0,5922181643	4,9399635328	0,0000007814	0,0000107759
LPAR1	46,35659675	3,7067612653	0,4624654173	8,0152182765	0,0000000000	0,0000000000
LPAR5	221,7286274	-2,5360389722	0,4654280874	-5,4488266627	0,0000000507	0,0000010096
LPCAT2	117,6088227	2,9202858703	0,4967795485	5,8784341651	0,0000000041	0,0000001138
LPGAT1	909,1882754	-0,5827605396	0,1943453210	-2,9985826602	0,0027123857	0,0098376712
LPIN3	43,68218756	3,6836896322	0,8602420833	4,2937792793	0,0000175657	0,0001536286
LPP	540,8009729	0,9521920224	0,3296771030	2,8882564600	0,0038738388	0,0132004736
LPP-AS2	17,93029233	1,3951946770	0,4183483455	3,3350068003	0,0008529731	0,0038168279
LPXN	1019,401147	-1,1424260461	0,3652502962	-3,1277895130	0,0017612626	0,0069175942
LRATD2	367,6188675	2,0856290283	0,4664266593	4,4737612313	0,0000076856	0,0000768127
LRCH3	492,1585405	-0,4092875532	0,1350325397	-3,0310290697	0,0024372178	0,0090123494
LRFN1	90,91483694	-0,9058994162	0,3149988515	-2,8758816481	0,0040290075	0,0135881836
LRFN4	40,52784705	1,7424185210	0,4677119820	3,7254050006	0,0001949886	0,0011428564
LRIF1	212,2584084	-1,2171389287	0,2620975476	-4,6438364387	0,0000034199	0,0000385853
LRIG3	30,83066575	7,6815667216	1,0999342423	6,9837514078	0,0000000000	0,0000000002
LRMDA	34,44126828	3,4228919381	0,5257816671	6,5101038206	0,0000000001	0,0000000033
LRP1	331,066822	3,1849816615	0,7248247334	4,3941404241	0,0000111212	0,0001040968
LRP10	1926,26879	-0,6177111411	0,1566585704	-3,9430812049	0,0000787801	0,0005377220
LRP11	132,5437057	3,2667487135	0,7272676390	4,4918109376	0,0000070620	0,0000714697
LRP2BP	15,22316611	0,7924381560	0,3283051516	2,4137364036	0,0157904011	0,0411817086
LRP5	63,00121858	3,8334549868	0,6480521967	5,9153491125	0,0000000033	0,0000000940
LRP6	38,48527176	6,0727982747	0,9912749061	6,1262503847	0,0000000009	0,0000000299
LRPPRC	650,2618364	0,8565523037	0,2156246276	3,9724233406	0,0000711451	0,0004958592
LRRC1	81,08406419	1,1834263554	0,4765653091	2,4832406654	0,0130193095	0,0352173228
LRRC14	539,7378812	-0,5913436180	0,2100522986	-2,8152218376	0,0048743574	0,0157562080
LRRC25	316,4264272	2,0754570652	0,6986965149	2,9704843534	0,0029733055	0,0105990705
LRRC3	12,97302898	1,9702144641	0,5257291701	3,7475864528	0,0001785457	0,0010605585
LRRC34	11,40857703	1,3110704560	0,4814501247	2,7231698338	0,0064658809	0,0199063946
LRRC37B	175,932725	-0,3784784523	0,1254932186	-3,0158836079	0,0025614711	0,0093841581
LRRC42	116,1870451	0,7119880950	0,2505437444	2,8417719804	0,0044863621	0,0147716326
LRRC43	30,4831851	-1,0816004999	0,3935854813	-2,7480701178	0,0059947188	0,0186779895
LRRC45	163,3490518	-0,4080226493	0,1550155018	-2,6322054542	0,0084832545	0,0248658856
LRRC46	17,72456734	-1,4293767247	0,4836510401	-2,9556111273	0,0031204013	0,0110444701
[illegible]	[illegible]	[illegible]	[illegible]	[illegible]	[illegible]	[illegible]

[illegible]						
MGP	63,74676973	3,6632884692	0,7439135255	4,9243471498	0,0000008464	0,0000116017
MGST1	66,002411	1,7134735146	0,6867205629	2,4950994343	0,0125921938	0,0342516571
MGST2	121,8113422	1,6820067911	0,4290446623	3,9210625360	0,0001275911	0,0008013886
MGST3	329,1863019	0,4084845689	0,1700155448	2,4026305918	0,0162776214	0,0421097029
MHENCR	116,7547137	-0,5747019852	0,2098287463	-2,7387661554	0,0061670221	0,0191218883
MIA3	629,7178954	-0,2776206890	0,1194472168	-2,3242086671	0,0201143130	0,0499517810
MIAT	1902,103444	-1,4184760532	0,3506921689	-4,0447896447	0,0000523701	0,0003862476
MICAL2	267,7916457	1,0912455281	0,2980922062	3,6607650768	0,0002514632	0,0014067728
MICAL3	148,0191562	1,3821953132	0,4035046478	3,4321330267	0,0005966493	0,0029427962
MICALL2	57,97990223	2,3859528280	0,6273115738	3,8034573690	0,0001426906	0,0008798053
MICB-DT	23,37691241	-1,3950341846	0,3947221858	-3,5342178237	0,0004089839	0,0020875703
MICOS10	245,91322	0,4883573701	0,1538779515	3,1736669585	0,0015052630	0,0060516249
MID1	36,71929505	1,7587478981	0,5373496619	3,2730061960	0,0010641090	0,0045469546
MID1IP1	95,15487868	0,6607180813	0,2391852153	2,7623700754	0,0057383395	0,0180252158
MIDN	1106,104528	1,3782228699	0,4701624261	2,9313760384	0,0033745402	0,0117748922
MIF	1184,834322	1,1921070185	0,2557163895	4,6618330340	0,0000031341	0,0000358822
MIF4GD	556,068081	-1,1536089471	0,1897216370	-6,0805344379	0,0000000012	0,0000000381
MIGA2	297,8789708	-0,7282757436	0,2064945312	-3,5268524508	0,0004205310	0,0021368456
MILR1	77,56419422	2,4409471796	0,5407973965	4,5136074903	0,0000063734	0,0000654856
MINDY2	420,2251208	-0,5088870138	0,1694610621	-3,0029731160	0,0026735605	0,0097222515
MINDY4	26,15720283	2,5299814753	0,5674986608	4,4581292289	0,0000082678	0,0000814965
MIOS	265,027629	0,9466318000	0,2039414560	4,6416840329	0,0000034558	0,0000388923
MIR100HG	20,57025687	1,4761273679	0,5845750135	2,5251290831	0,0115655799	0,0318870761
MIR1244-1	16,02028567	-1,5975761267	0,6479463881	-2,4655982470	0,0136784276	0,0366762693
MIR1244-2	16,02028567	-1,5975761267	0,6479463881	-2,4655982470	0,0136784276	0,0366762693
MIR1244-3	16,02028567	-1,5975761267	0,6479463881	-2,4655982470	0,0136784276	0,0366762693
MIR1244-4	16,02028567	-1,5975761267	0,6479463881	-2,4655982470	0,0136784276	0,0366762693
MIR181A2HG	25,81935485	-3,7612128257	0,4576261745	-8,2189635027	0,0000000000	0,0000000000
MIR210HG	36,41493657	2,3073047559	0,5911549345	3,9030457519	0,0000949897	0,0006285948
MIR22HG	267,7566544	1,0448149697	0,4072684238	2,5654210066	0,0103050683	0,0290894258
MIR3142HG	14,4538529	-3,1937682005	0,6457832410	-4,9379497055	0,0000007895	0,0000108766
MIR3667HG	66,98750866	-0,8596947716	0,2880672428	-2,9843544975	0,0028417732	0,0102218780
MIR663AHG	30,40567469	-2,0116669493	0,4597480318	-4,3756100325	0,0000121093	0,0001118680
MITD1	277,3455863	-0,9246532120	0,1826167837	-5,0632576590	0,0000004143	0,0000062731
MITF	109,7066255	2,6246675271	0,5830122674	4,5019068869	0,0000067346	0,0000685173
MKI67	102,0987039	1,8331323433	0,5779984979	3,1715174867	0,0015164474	0,0060893144
MKRN2	394,4516638	-0,5952380953	0,1351081565	-4,4056414559	0,0000105471	0,0000996941
MLEC	798,2894528	0,4863958232	0,1879126831	2,5884145512	0,0096418866	0,0275424009
MLF1	65,94676309	2,6215887208	0,6588546926	3,9790089530	0,0000692031	0,0004843860
MLLT10	226,4011956	0,5586758531	0,2262326245	2,4694756306	0,0135311235	0,0363675962
MLLT3	341,7400207	-1,1472834914	0,3833394188	-2,9929659439	0,0027637112	0,0099928036
MLXIPL	19,49671881	1,9659490959	0,4096478518	4,7991197491	0,0000015936	0,0000199911
MLYCD	106,1665402	-1,1608079961	0,2805296302	-4,1379158248	0,0000350475	0,0002736306
MMAA	80,28954698	-0,5825071495	0,2013450715	-2,8930787580	0,0038148563	0,0130457229
MMD	146,7530063	1,5165325020	0,4283721946	3,5402216136	0,0003997912	0,0020538890
MMEL1	25,31750352	2,6572498837	1,0058095179	2,6419017084	0,0082441977	0,0242188476
MMGT1	147,310528	0,7878888765	0,2690094001	2,9288525835	0,0034021573	0,0118450693
MMP11	11,50083939	1,1432478759	0,4884465235	2,3405768834	0,0192538706	0,0482322070
MMP14	53,96727883	5,8929699888	1,0544755639	5,5885315322	0,0000000229	0,0000005020
MMP2	99,15729312	7,2146738691	0,8486628735	8,5012248028	0,0000000000	0,0000000000
MMP9	167,390769	-2,8862285755	0,7522728985	-3,8366775956	0,0001247100	0,0007873344
MMS19	719,0894566	-0,3723534200	0,1296745284	-2,8714461083	0,0040859845	0,0137390334
MN1	20,88618	-1,5826801314	0,5086382868	-3,1116024346	0,0018607491	0,0072386265
MOB1B	153,5378011	1,3476205492	0,3447558481	3,9089128051	0,0000927124	0,0006165559
MOB2	243,7043158	-0,8026349128	0,1055066307	-7,6074357332	0,0000000000	0,0000000000
MOB3B	37,38701144	2,3602567325	0,4836708021	4,8798826029	0,0000010615	0,0000141602
MOCOS	16,18725535	1,2495789623	0,4485769095	2,7856515482	0,0053420262	0,0170266808
MOCS3	113,087421	-0,6637629511	0,2149413683	-3,0881116946	0,0020143275	0,0077178618
MOGS	940,8934436	-0,5073534505	0,1748852533	-2,9043846026	0,0036797582	0,0126706437
MON1A	116,9514326	-1,2780550806	0,2246858777	-5,6881860740	0,0000000128	0,0000003043
MON1B	752,8158395	-0,6749193688	0,1873203494	-3,6030221538	0,0003145387	0,0016943642
MORC3	591,2499557	-0,4329331754	0,1763573094	-2,4548638033	0,0140937974	0,0375657052
MORF4L1	1853,489814	0,5103896770	0,1394420725	3,6666659667	0,0002272113	0,0013008253
MORF4L2	741,4849708	1,3091078637	0,1923822919	6,8047212181	0,0000000000	0,0000000006
MORN2	37,88534274	2,4022650278	0,4921830836	4,8808362334	0,0000010564	0,0000141059
MOV10	789,7007884	-0,5731595426	0,1509779741	-3,7963123171	0,0001468645	0,0009018228
MPDU1	273,8584833	0,7434442304	0,2264719417	3,2827211386	0,0010281031	0,0044268292
MPEG1	504,0274954	2,8976708264	0,6967473220	4,1588545851	0,0000319847	0,0002530950
MPG	355,3265822	-0,5039787847	0,2059290631	-2,4473417060	0,0143914326	0,0382143781
MPHOSPH6	97,62677186	0,6265246687	0,2433856626	2,5742053251	0,0100470630	0,0285002039
MPHOSPH8	588,8514544	-0,6150272531	0,1264581957	-4,8634442873	0,0000011536	0,0000152432
MPIG6B	47,97366406	-1,4003468574	0,5390315111	-2,5978942394	0,0093797379	0,0269192477
MPLKIP	176,7353634	-0,6477688542	0,1646065069	-3,9352566695	0,0000831078	0,0005615619
MPND	74,15866845	0,5627944506	0,2417452153	2,3280479406	0,0199095582	0,0495781831
MPP1	560,2582591	1,2841074141	0,5019831121	2,5580689530	0,0105255219	0,0295615341
MPST	250,035791	-0,5008551314	0,1934831983	-2,5886223837	0,0096360419	0,0275315483
MPZL1	297,8957549	1,9203683007	0,1875880289	10,2366249691	0,0000000000	0,0000000000
MRAS	58,8273348	1,7905944534	0,7264638615	2,4648087102	0,0137086416	0,0367464699
MRC1	201,711179	2,8907253761	0,7288786648	3,9659898357	0,0000730920	0,0005076460
MRC2	48,58290707	3,8744317469	0,5821738511	6,6551112518	0,0000000000	0,0000000014
MREG	69,78219832	0,7673117505	0,2946946359	2,6037520088	0,0092209450	0,0265996288
MRFAP1L1	803,631582	-1,2515061509	0,2071403465	-6,0418270614	0,0000000015	0,0000000469
MRGBP	183,9657129	-0,6413273630	0,1229389627	-5,2166322931	0,0000001822	0,0000030504
MRI1	331,355467	-0,6952209290	0,2123187800	-3,2744203272	0,0010587899	0,0045271005
MRM3	131,8205692	-0,7161301105	0,2225897716	-3,2172642320	0,0012941935	0,0053407176
MROH6	63,53194478	3,3075758040	0,9709886451	3,4064000859	0,0006582564	0,0030775030
MROH8	14,59958355	-1,4349034136	0,4361167344	-3,2901819638	0,0010012261	0,0043232715
MRPL1	79,16152216	1,0171978076	0,3059054045	3,3252031269	0,0008835414	0,0039236117
MRPL11	186,993649	0,4687726025	0,1541643734	3,0407323828	0,0023600349	0,0087678913
MRPL15	337,425876	0,7962330138	0,2040136050	3,9028427229	0,0000950695	0,0006288134
MRPL16	252,9134683	-0,5400982794	0,1537218092	-3,5134785514	0,0004422801	0,0022321299
MRPL20-AS1	221,8890008	-0,9151959458	0,1862815533	-4,9129708758	0,0000008971	0,0000122335
MRPL24	197,3721568	0,4412813119	0,1597986171	2,7614829227	0,0057539349	0,0180488213
MRPL3	460,2944591	1,0057143000	0,1703489497	5,9038133074	0,0000000036	0,0000001000
MRPL34	223,4063854	-0,6159348089	0,1544687333	-3,9874400190	0,0000667901	0,0004711711
MRPL36	131,2191604	-0,5130978719	0,1451331976	-3,5353584193	0,0004072224	0,0020822197
MRPL37	352,5139255	0,3395035821	0,1136367818	2,9875416572	0,0028123097	0,0101267223
MRPL38	344,5845112	-0,3956236856	0,1157212522	-3,4187613431	0,0006290688	0,0029688869
MRPL46	163,796751	-0,5302756153	0,2167255131	-2,4467613797	0,0144146237	0,0382533191
MRPL48	231,2109225	-0,4238009184	0,1372643467	-3,0874799512	0,0020186139	0,0077320839
MRPL49	499,364854	-0,6580085027	0,1500067306	-4,3865265236	0,0000115175	0,0001069143
MRPL53	97,56599191	0,6639502515	0,2067505779	3,2113586256	0,0013210896	0,0054362169
MRPL54	184,4757272	0,7961465361	0,2213995149	3,5959723582	0,0003231821	0,0017317004
MRPL55	338,6659707	-0,7314470458	0,1303444702	-5,6116461601	0,0000000200	0,0000004496
MRPS11	253,3128234	-0,5174174975	0,1512045849	-3,4219696308	0,0006216926	0,0029392280
MRPS15	261,4715274	0,4221984827	0,1293787122	3,2632762805	0,0011013210	0,0046836589
MRPS18B	480,254707	-0,4725831485	0,1182273429	-3,9972407138	0,0000640851	0,0004551904
MRPS35	299,6281986	0,7707976324	0,1997851153	3,8581334307	0,0001142563	0,0007327452
MRPS6	981,7941335	-1,1772818023	0,2694028330	-4,3699681600	0,0000124265	0,0001141708
MRTFB	92,35849673	0,8334258646	0,3212407085	2,5943967950	0,0094757057	0,0271483608
MRTO4	200,6662037	1,4117861583	0,2975285742	4,7450439415	0,0000020846	0,0000252379
MS4A4A	444,7880818	3,7449755167	0,7180346989	5,2155812831	0,0000001832	0,0000030599
MS4A6A	517,6409456	5,1576781022	0,9253270053	5,5738877385	0,0000000249	0,0000005299
MS4A7	275,2085101	2,7904516289	0,6867287451	4,0632871705	0,0000483636	0,0003602515
MSANTD2	80,71510736	-1,0096846818	0,2219631734	-4,5488837925	0,0000053921	0,0000570239
MSL3	521,5382019	-0,4627418120	0,1624447381	-2,8486106563	0,0043910580	0,0145253480
MSLN	1833,780054	13,6459722599	1,6165550879	8,4413801888	0,0000000000	0,0000000000
MSR1	142,4862593	3,2271727739	0,7494218403	4,3062166064	0,0000166070	0,0001466738
MSRA	43,47896286	1,4010170340	0,4067467099	3,4444458921	0,0005722312	0,0027576911
MSRB1	237,7457779	-0,6225115959	0,2200636134	-2,8287802163	0,0046725776	0,0152556737
MSRB2	54,88766787	1,5801191486	0,2879117836	5,5229387588	0,0000000333	0,0000007000
MSRB3	35,34216821	1,6992462544	0,6242187175	2,7221968946	0,0064849492	0,0199538681
MST1R	99,1610601	2,9105214602	0,9257360001	3,1440080756	0,0016665084	0,0065896602
MT1E	46,62506244	3,4757397806	0,9969938957	3,4862197208	0,0004898884	0,0024251685
MT1X	86,49345273	1,3695195691	0,4994402151	2,7421091208	0,0061040058	0,0189676810
MT2A	342,0988662	1,2562938360	0,5357338433	2,3449962173	0,0190272726	0,0477722607
MTAP	178,7001322	1,0566998272	0,2924968458	3,6126879388	0,0003030093	0,0016440585
MTARC1	15,78891587	1,7538881581	0,4056340039	4,3238193579	0,0000153351	0,0001368768
MTCO1P12	1076,910583	2,4040843804	0,9863532636	2,4373458545	0,0147955167	0,0390715430
MTDH	1050,084169	0,4348587900	0,1272306785	3,4178768459	0,0006311166	0,0029775076
MTERF2	158,2370486	-0,9342101940	0,1846938754	-5,0581547013	0,0000004233	0,0000063665
MTF2	496,4578683	-0,5017911918	0,1976526740	-2,5387523565	0,0111248539	0,0309501519
MTFR1	38,72751783	0,9682040526	0,3806037998	2,5438633379	0,0109633969	0,0305794879
MTFR2	15,60686839	1,4659095494	0,6201485490	2,3638038849	0,0180883851	0,0457994099
MTG1	427,903414	-0,9225689380	0,1703546196	-5,4155792215	0,0000000611	0,0000011713
MTG2	224,1497012	-0,6163542565	0,1715306953	-3,5932582427	0,0003265674	0,0017456700
MTHFD1	356,6050505	0,7223933096	0,2033158789	3,5530589808	0,0003807791	0,0019743014
MTHFD1L	108,4724527	3,5154756144	0,5329335870	6,5964809853	0,0000000000	0,0000000020
MTHFD2	286,8539686	1,3316273050	0,2566486024	5,1885225004	0,0000002120	0,0000034720
MTHFS	113,3157357	-0,7653894747	0,2512936978	-3,0457965370	0,0023206481	0,0086533437
MTHFSD	95,63476494	-0,9005405406	0,2673959297	-3,3678169351	0,0007576589	0,0034558076
MTIF2	250,3835382	0,4347128258	0,1867406906	2,3278956188	0,0199176469	0,0495798576
MTM1	108,6781835	1,3995237798	0,2633056120	5,3152068007	0,0000001065	0,0000018248
MTMR1	341,0112815	0,5266259611	0,2262465264	2,3277084933	0,0199275879	0,0495887100
MTMR10	258,8098667	0,5137865840	0,1439366131	3,5695326509	0,0003576173	0,0018788168
MTMR11	24,28679849	2,5179077824	0,5941648752	4,2377257346	0,0000225795	0,0001902718
MTMR2	323,2511164	0,3571804077	0,1408632843	2,5356510087	0,0112238526	0,0311619339
MTMR3	709,3450073	-0,6178106888	0,1499788363	-4,1193191252	0,0000379994	0,0002941181
MTND2P28	1016,670018	1,1296937258	0,3675326821	3,0737221836	0,0021140549	0,0080178020
MTO1	345,9325897	-0,4010896803	0,1666135272	-2,4073056195	0,0160707148	0,0417107252
MTPAP	161,5529639	0,6455344532	0,1968757948	3,2788919211	0,0010421554	0,0044730267
MTRFR	237,1613349	-0,8219352544	0,1695079782	-4,8489478972	0,0000012412	0,0000162829
MTSS1	589,0925382	-1,0055649626	0,3936797495	-2,5542715721	0,0106410225	0,0298271247
MTUS1	91,52973161	7,2288830696	1,2283465222	5,8850519289	0,0000000040	0,0000001100
MTX1	277,0378642	0,3925132964	0,1573771561	2,4940932100	0,0126279379	0,0342169885
MTX2	189,603146	0,5430874615	0,2211071384	2,4562185801	0,0140407726	0,0374606144
MUC1	717,6957113	9,8195823360	1,5128298929	6,4908273063	0,0000000001	0,0000000037
MUC16	1311,605328	12,2576526677	1,7399287067	7,0450485528	0,0000000000	0,0000000001
MUC20	370,5349119	3,6883594229	1,0074962311	3,6609159847	0,0002513152	0,0014065289
MUS81	542,2181906	-1,0198887377	0,1881198704	-5,4214833109	0,0000000591	0,0000011379
MUTYH	244,0822305	-0,9330873101	0,2310782340	-4,0379714464	0,0000539154	0,0003958092
MVD	471,5166006	-1,2981714925	0,2096874442	-6,1909834309	0,0000000006	0,0000000206
MX1	806,3610574	1,4475002701	0,3552545423	4,0745440172	0,0000461046	0,0003458129
MX2	311,2553552	0,8122782380	0,3119209770	2,6041154586	0,0092111721	0,0265942463
MXD1	396,4542178	0,9992486132	0,2546371325	3,9242061964	0,0000870162	0,0005841585
MXD4	1683,693327	-1,3021578364	0,2241319279	-5,8097828760	0,0000000063	0,0000001635
MXRA5	179,2753863	10,6642842982	1,3763025397	7,7485029565	0,0000000000	0,0000000000
MXRA7	949,8956652	-0,6783532582	0,2636255566	-2,5731695625	0,0100771823	0,0285732497
MXRA8	105,7558212	4,1775475467	0,9369037351	4,4588866393	0,0000082386	0,0000813528
MYADM	1197,384164	2,8632076103	0,3845526266	7,4455546833	0,0000000000	0,0000000000
MYBBP1A	302,1549538	0,6880461774	0,2426425173	2,8356502125	0,0045766621	0,0135635403
MYBL2	113,256855	4,6224765641	0,8828018359	5,2361428990	0,0000001640	0,0000027782
MYC	244,161149	1,3421238862	0,3465709213	3,8714626960	0,0001081838	0,0007007486
MYCL	30,52158998	3,3099853642	0,9309850946	3,5553580431	0,0003774647	0,0019605233
MYDGF	512,8902516	0,4974484793	0,2095281418	2,3740122009	0,0175959690	0,0448054755
MYEF2	70,53686414	1,4339428748	0,4467910088	3,2094264355	0,0013300008	0,0054662016
MYH10	223,501078	1,6685502035	0,3211834059	5,1950075026	0,0000002047	0,0000033726
MYH11	29,35658602	-1,0140980597	0,4283643320	-2,3673751147	0,0179147650	0,0454538618
MYL6	5873,321669	0,4068606783	0,1358363455	2,9952269155	0,0027424074	0,0099259949
MYL6B	45,82992443	1,6596295885	0,3358909081	4,9409780031	0,0000007773	0,0000107310
MYL9	50,36083966	2,9862533145	0,9694121012	3,0804786847	0,0020666815	0,0078736294
MYLK	96,80907529	1,6504895770	0,5097434117	3,2378830975	0,0012042014	0,0050274662
MYLK-AS1	17,93177001	-1,2596155346	0,3368227459	-3,7396985510	0,0001842411	0,0010876688
MYO10	108,1570553	5,4824776603	0,7659638629	7,1585537928	0,0000000000	0,0000000001
MYO1B	46,95118822	4,0863194450	0,6010295208	6,7988664512	0,0000000000	0,0000000006
MYO1C	341,1335756	3,4956008286	0,6645408120	5,2593823777	0,0000001445	0,0000024878
MYO1D	141,9138275	1,4371578005	0,4655532212	3,0869892740	0,0020219489	0,0077398232
MYO1E	172,6482296	1,6016958455	0,4672382224	3,4280061563	0,0006080317	0,0028861058
MYO5B	214,3307144	5,8194082787	0,7884569909	7,3807556022	0,0000000000	0,0000000000
MYO7A	328,8721384	4,5609231556	0,5844431386	7,8038763488	0,0000000000	0,0000000000
MYOF	551,0926005	6,2658576068	1,0027461439	6,2486977837	0,0000000004	0,0000000149
MYOM2	488,4605561	-3,5572299325	1,2832758530	-2,7719916370	0,0055714468	0,0176326531
MYORG	44,78239235	1,1785179389	0,3906517903	3,0167990167	0,0025545913	0,0093666027
MYRF	257,1048178	1,8185702731	0,6971634055	2,6085280135	0,0090932568	0,0263158970
NAA15	331,5265288	0,5078629403	0,1620140698	3,1346861731	0,0017203922	0,0067824549
NAA16	302,4776462	0,8302781416	0,1753682598	4,7344808532	0,0000021962	0,0000262983
NAA35	215,4212977	0,4438680789	0,1855903744	2,3916546337	0,0167726169	0,0431085299
NAA40	456,1576218	-0,3879682044	0,1652134174	-2,3482850885	0,0188600796	0,0473789980
NAA50	964,6183843	0,5227017704	0,2172537614	2,4059503822	0,0161304554	0,0418173978
NAA60	563,9113424	-0,5754423253	0,2166586833	-2,6559855180	0,0079077010	0,0235221339
NAALADL1	202,183598	-1,2493565220	0,2751516392	-4,5406108629	0,0000056091	0,0000588917
NAB1	157,5637725	0,9545924673	0,3374020361	2,8292432755	0,0046658202	0,0152431894
NACA	2250,795719	0,7428505098	0,2048083178	3,6270367357	0,0002866947	0,0015702325
NADK2	144,3663134	0,9643674531	0,2001910429	4,8172357729	0,0000014556	0,0000185259
NAE1	412,4526827	-0,3946058714	0,1348625776	-2,9258353893	0,0034367504	0,0144106884
NAF1	51,00617394	1,0740222996	0,3763744755	2,8536002561	0,0043226886	0,0143468935
NAGA	593,816505	0,9376766138	0,3085781586	3,0387005291	0,0023760092	0,0088150726
NAIP	262,876571	0,9619631776	0,3560369864	2,7018620491	0,0068952157	0,0209928983
NAMPT	1083,617334	2,2567388818	0,4137072980	5,4549170082	0,0000000490	0,0000009785
NAMPTP1	35,2280772	2,8423580862	0,8060551196	3,5262790884	0,0004214426	0,0021398028
NANS	267,727093	1,0370896313	0,2109002214	4,9174421186	0,0000008768	0,0000119940
NAP1L1	1060,497637	0,6910158207	0,2177652836	3,1732128831	0,0015076194	0,0060556719
NAPSB	64,09020803	3,8999263830	1,4286552065	2,7297883810	0,0063374993	0,0195693270
NARF	976,7731521	-0,5552311686	0,1703229320	-3,2598732426	0,0011146202	0,0047208267
NARS1	975,7247534	0,4896038529	0,1833221052	2,6707300382	0,0075686491	0,0227143850
NAT14	31,06234783	1,5141101072	0,4494158444	3,3690625865	0,0007542430	0,0034437282
NATD1	168,6071175	-0,7295108793	0,2611220265	-2,7937546640	0,0052099888	0,0166413347
NAV1	75,28256758	2,2669329198	0,3336671616	6,7939966771	0,0000000000	0,0000000006
NBDY	229,4660601	0,4442102422	0,1905476733	2,3312264504	0,0197414221	0,0492437994
NBEA	28,12723771	0,9959049105	0,3771761973	2,6404235409	0,0082802474	0,0244037946
NBEAL1	177,0660333	0,5209660306	0,1939511449	2,6854496317	0,0072432291	0,0218694177
NBEAL2	3759,285162	-0,7077152846	0,1647976795	-4,2944499997	0,0000175127	0,0001532645
NBL1	261,8147656	2,0187177708	0,6203102916	3,2543676901	0,0011364508	0,0047920606
NBPF12	599,7796735	-0,4588188736	0,1845907064	-2,4856011580	0,0129332824	0,0350054255
NBPF20	190,1496417	-0,5933634219	0,2378789564	-2,4943922355	0,0126173033	0,0343018394
NBPF3	191,5842689	-0,7553898431	0,2987110650	-2,5288311399	0,0114443073	0,0316369636
NBPF8	407,1125781	-0,8441587716	0,2722720524	-3,1004238744	0,0019324388	0,0074514533

NCAPH2	508,9411294	-0,4367782635	0,1210995188	-3,6067712555	0,0003100307	0,0016772563
NCBP2	571,3010006	0,3259290630	0,1353524189	2,4080032370	0,0160400388	0,0416621432
NCBP3	729,1464773	-0,9880504257	0,2465869068	-4,0064989235	0,0000616254	0,0004419169
NCDN	174,315653	-0,5416320613	0,1677848235	-3,2281350007	0,0012460016	0,0051715020
NCEH1	221,4558066	1,5776105612	0,4084681378	3,8622610067	0,0001123424	0,0007226069
NCF1	198,0673299	1,7557841967	0,4945046285	3,5505920382	0,0003843657	0,0019875284
NCF4	143,5943267	2,1895671435	0,5976455564	3,6636550209	0,0002486416	0,0013967931
NCK1	367,2568592	-0,3469039460	0,1222180368	-2,8384022113	0,0045340010	0,0148920579
NCKAP1	320,3423219	2,1814069064	0,6715945138	3,2481011408	0,0011617797	0,0048836834
NCL	3655,722906	0,3444658544	0,1210739609	2,8451103895	0,0044396034	0,0146427498
NCOA1	1287,023404	-0,4217195769	0,1794007757	-2,3507121148	0,0187375246	0,0471238940
NCOA3	737,5740644	0,4352293618	0,1265128164	3,4401997692	0,0005812850	0,0027903237
NCOA7	391,1579368	0,6727578884	0,2348862634	2,8642283241	0,0041802657	0,0139861736
NCOR1	1586,854733	-0,7612882546	0,2329701707	-3,2677542028	0,0010840447	0,0046218683
NCR1	449,8592821	-0,9854250313	0,3289298993	-2,9958511931	0,0027367995	0,0099104982
ND1	16648,74741	1,1072353407	0,4114523788	2,6910412916	0,0071229378	0,0215642697
ND2	23699,47554	1,1444773995	0,3805221833	3,0076496189	0,0026327549	0,0096009254
ND3	2941,713617	1,2156308189	0,4957876377	2,4519183748	0,0142096891	0,0378362940
NDC1	174,391233	0,4806280071	0,1951981095	2,4622574903	0,0138065507	0,0369413231
NDC80	63,4087355	1,5461683705	0,3784281626	4,0857645479	0,0000439319	0,0003320177
NDE1	372,7808525	-0,7412579023	0,2115606490	-3,5037640820	0,0004587316	0,0022978259
NDFIP1	454,7576139	0,5141415802	0,2205008272	2,3316779505	0,0197176396	0,0492095936
NDFIP2	199,4540764	1,5117893099	0,5785602903	2,6130191362	0,0089746277	0,0260510004
NDN	40,82073651	4,2193100963	0,9802251143	4,4064470837	0,0000105080	0,0000993940
NDOR1	246,5092373	-0,7799982523	0,2557632077	-3,0473435145	0,0023087370	0,0086189650
NDRG1	1060,98637	1,8462325252	0,5149634524	3,5851789129	0,0003368472	0,0017920804
NDRG3	620,8040164	-0,6501368840	0,1702909841	-3,8178144738	0,0001346391	0,0008378275
NDRG4	25,43112029	4,0023098399	0,7547634055	5,3027343545	0,0000001141	0,0000020338
NDST1	119,5949361	3,4145626109	0,7094421421	4,8130247813	0,0000014866	0,0000188418
NDUFA13	688,45882	0,3717343258	0,1541260877	2,4118845251	0,0158703070	0,0413420424
NDUFA4	896,2895357	0,5353227568	0,1389672965	3,8521491757	0,0001170857	0,0007472607
NDUFA6	423,9147375	-0,2344589861	0,0894484403	-2,6211411324	0,0087635974	0,0255514357
NDUFAB1	299,9790785	0,4053233243	0,1628287058	2,4890928360	0,0128069123	0,0347122512
NDUFAF2	62,09862142	0,6950152669	0,2361457890	2,9431618063	0,0032487857	0,0113906370
NDUFB5	248,9017335	0,7143796286	0,2167518271	3,2958413224	0,0009812742	0,0042633799
NDUFB9	696,4907762	0,8034504940	0,3166124972	2,5376461799	0,0111600750	0,0310360763
NDUFS2	1205,088893	-0,4765887240	0,1286267884	-3,7052058127	0,0002112192	0,0012234633
NDUFS4	209,4683856	0,6904947068	0,1938884012	3,5612997093	0,0003690236	0,0019237247
NDUFS5	532,2201082	0,6222004102	0,1615400279	3,8516794777	0,0001173105	0,0007479864
NECTIN2	328,8630717	4,5116577357	0,6450659236	6,9941033472	0,0000000000	0,0000000002
NECTIN3	40,46526525	2,8769964209	0,6933671897	4,1493114526	0,0000333477	0,0002621845
NEDD4	256,8769839	0,6875154787	0,2112482472	3,2545381460	0,0011357991	0,0047911985
NEDD4L	116,4173016	2,8318126471	0,5298267505	5,3446587285	0,0000000906	0,0000016749
NEFH	11,12935507	2,5414135040	0,8156254832	3,1159074310	0,0018337983	0,0071461855
NEK2	41,44307146	1,3265279099	0,5625989539	2,3578570502	0,0183807595	0,0464261884
NEK3	50,11006566	1,3605190766	0,4081731293	3,3331911854	0,0008585592	0,0038278262
NEK6	291,4116696	1,6681295576	0,4013827209	4,1559575707	0,0000323928	0,0002560229
NELFB	302,913683	-0,3868846119	0,1510469460	-2,5613534208	0,0104265229	0,0293539004
NEMP2-DT	9,49654126	-1,4414914734	0,5340178923	-2,6993317906	0,0069478869	0,0211389145
NEPRO-AS1	24,2196011	-1,4909580823	0,3714411303	-4,0139848836	0,0000597022	0,0004304184
NET1	381,9223842	4,6830737911	0,7502638326	6,2419026313	0,0000000004	0,0000000154
NETO2	148,1669652	1,6109616673	0,5254940544	3,0656125002	0,0021722391	0,0082061012
NEU1	871,5533401	0,8112544200	0,3085087632	2,6295992750	0,0085485568	0,0250192596
NF2	432,4099183	-0,3373425003	0,1186877083	-2,8422888959	0,0044793553	0,0147521709
NFAM1	69,79118177	2,1771991298	0,8215349600	2,6501600491	0,0080453648	0,0238946625
NFATC2	1312,643164	-1,1054499505	0,2594222377	-4,2611996570	0,0000203332	0,0001742328
NFATC2IP	719,4659208	-0,3763515420	0,1468607218	-2,5626425996	0,0103878920	0,0292757582
NFATC3	1911,673843	-0,7395848337	0,2564918762	-2,8834629956	0,0039332886	0,0133422540
NFE2L3	88,61011465	1,1305467865	0,3292298723	3,4339121454	0,0005949348	0,0028366256
NFIA	120,666516	1,1663783371	0,3801541541	3,0681720155	0,0021537260	0,0081430204
NFIC	160,065722	0,8857408821	0,3212594558	2,7570889078	0,0058319892	0,0182582992
NFIX	52,50004091	3,4947186250	0,6948091414	5,0297523766	0,0000004911	0,0000072008
NFKB1	1348,764229	-1,3442267307	0,3442153127	-3,9051915500	0,0000941508	0,0006245784
NFKB2	709,6372784	-1,3972137880	0,3394074572	-4,1166266632	0,0000384458	0,0002968908
NFKBIA	7644,238318	0,7408809669	0,3185365408	2,3251600865	0,0200634017	0,0498621468
NFKBIB	274,4665352	-0,5687913760	0,2363273799	-2,4067942366	0,0160932343	0,0417450248
NFKBIZ	1208,291477	2,3735122548	0,3942034867	6,0210331340	0,0000000017	0,0000000529
NFYB	226,070482	-0,5596531289	0,1909881628	-2,9303026995	0,0033863196	0,0118032848
NHEJ1	137,7006032	-0,4643109670	0,1772103252	-2,6201123810	0,0087900795	0,0256036506
NHLRC3	254,2975171	0,6338642658	0,2096498251	3,0234428558	0,0024991622	0,0091984478
NHP2	423,1069203	0,7603485529	0,2012803078	3,7770637430	0,0001586881	0,0009610064
NHSL1	20,67106485	3,3852944469	0,7927247886	4,2704536250	0,0000195076	0,0001679923
NHSL2	304,8199251	-1,2066555151	0,4139271518	-2,9151398017	0,0035552924	0,0122755369
NIBAN2	449,4091007	4,9803417482	0,9237403589	5,3913869845	0,0000000699	0,0000013217
NID2	39,19575336	3,1909432091	0,6769880258	4,7134411356	0,0000024357	0,0000288087
NIFK-AS1	88,6678114	-1,4763645352	0,3047913090	-4,8438537833	0,0000012734	0,0000165610
NIPBL	934,7432321	-0,4822607077	0,1722061086	-2,8004954851	0,0051025802	0,0163681434
NIPSNAP2	436,0784546	0,7942987125	0,2275919568	3,4900122301	0,0004829984	0,0024007234
NIPSNAP3A	212,0360139	0,7851538222	0,2628581940	2,9869862919	0,0028174236	0,0101397115
NIT1	216,3436044	-0,6405888871	0,1480071988	-4,3280927699	0,0000150406	0,0001348745
NKAPP1	22,79849611	-0,7786910301	0,2646647744	-2,9421785805	0,0032591192	0,0114179450
NKG7	7033,45709	-0,9163915683	0,2945969107	-3,1106625190	0,0018669814	0,0072533291
NLE1	65,7525886	0,8333366468	0,3082136407	2,7037647841	0,0068559801	0,0208977005
NLN	125,6806065	2,4158820717	0,4484314732	5,3874052465	0,0000000715	0,0000013475
NLRC3	2271,551793	-1,0483677200	0,2917669423	-3,5931387161	0,0003267185	0,0017457847
NLRP1	2762,98409	-0,7846441757	0,2914047117	-2,6926269353	0,0070891544	0,0214730974
NLRP3	109,6447228	1,8252445363	0,4213173017	4,3322325690	0,0000147605	0,0001325392
NLRX1	203,3246551	-0,6143638278	0,1872372329	-3,2812054431	0,0010336443	0,0044456155
NMD3	311,5889274	0,9802857983	0,2108717027	4,6487308896	0,0000033398	0,0000378402
NME1	258,5644358	1,6803234831	0,3784225293	4,4403368007	0,0000089818	0,0000871997
NME1-NME2	62,29704842	3,5378735140	0,8346138677	4,2389344950	0,0000224583	0,0001894041
NME2	674,5588346	1,7271173800	0,3133590201	5,5116245981	0,0000000356	0,0000007396
NMRK1	496,1320156	-0,9352152823	0,2887395235	-3,2389587027	0,0011996694	0,0050118544
NNMT	101,4783402	6,8417193428	0,8092957875	8,4539169101	0,0000000000	0,0000000000
NOCT	61,15742702	1,6026108553	0,5672777969	2,8256188841	0,0047189373	0,0152793938
NOL10	222,1094386	0,4355451250	0,1127247052	3,8637947587	0,0001116390	0,0007200102
NOL12	264,9215544	-0,7455052347	0,1919452153	-3,8839479974	0,0001027739	0,0006705461
NOL3	22,30801563	2,2749181430	0,5681697405	4,0034130310	0,0000624352	0,0004460601
NOP16	197,7765993	0,6947756928	0,2379006274	2,9204449794	0,0034953191	0,0121223899
NOP58	522,7562278	0,5260477997	0,1317427181	3,9929923687	0,0000652444	0,0004619619
NOSIP	431,1356217	-0,6622429450	0,1026899130	-6,4489580897	0,0000000001	0,0000000047
NOTCH2	1321,279	0,6513930175	0,1379459170	4,7220898730	0,0000023343	0,0000278301
NOTCH2NLC	401,5127275	-0,6655990499	0,2474240186	-2,6901149438	0,0071427412	0,0216144134
NOTCH3	97,97857804	4,9809244446	1,2553609206	3,9677220371	0,0000725626	0,0005047506
NPAS2	108,394417	6,4959680909	0,9873758912	6,5790223853	0,0000000000	0,0000000022
NPAT	364,2776323	-0,8458558456	0,1496736389	-5,6499985733	0,0000000160	0,0000003711
NPC1	1021,801712	-0,6108460901	0,1990309474	-3,0691010521	0,0021470395	0,0081223025
NPC2	981,4408442	2,0065237778	0,3925299865	5,1117719585	0,0000003192	0,0000049548
NPDC1	65,50106099	3,5131885944	0,7501196369	4,6835043525	0,0000028201	0,0000328363
NPHP1	15,89687107	1,8767529914	0,6249919534	3,0028434464	0,0026746998	0,0097248676
NPIPB11	106,4697231	-0,7046839474	0,2904460020	-2,4262132808	0,0152573009	0,0400630647
NPIPB2	124,6377184	-0,6147313552	0,2244531491	-2,7387958584	0,0061664650	0,0191218883
NPL	372,6538355	1,7225590392	0,5860440688	2,9392964923	0,0032895822	0,0115126924
NPM1	1902,131363	1,1519832455	0,2322957154	4,9591229501	0,0000007081	0,0000098959
NPM3	21,58129336	2,5865396751	0,6197782876	4,1894781491	0,0000279597	0,0002261642
NPRL2	758,7919095	-0,8745798695	0,1965555435	-4,4500391604	0,0000085855	0,0000841050
NPTN	518,1817488	0,7359946932	0,2300952681	3,1986506257	0,0013807238	0,0056402255
NPTX1	40,58908278	-2,3034332969	0,5075710759	-4,5381492491	0,0000056750	0,0000593906
NR1H2	813,8384957	-0,3554449508	0,1394975593	-2,5480370599	0,0108330962	0,0302831724
NR2C2	442,6888006	-0,4618934754	0,1459990079	-3,1636754380	0,0015579042	0,0062234694
NR2F1-AS1	30,81793116	1,6924262558	0,4027873444	4,2017860776	0,0000264817	0,0002158397
NR2F6	139,6553638	3,0916841064	0,6962985812	4,4402465739	0,0000089856	0,0000871997
NR3C1	1384,831632	-1,4638160330	0,2472887580	-5,9194924908	0,0000000032	0,0000000919
NR3C2	36,14579875	1,5844834753	0,4978782589	3,2025563010	0,0013621367	0,0055761436
NR4A1	663,7884608	2,7566223091	0,5056720667	5,4514031733	0,0000000500	0,0000009965
NR4A1AS	19,91775284	1,6182998853	0,5209906161	3,1061977609	0,0018950993	0,0073446693
NR4A3	154,1720185	3,3248060255	0,6860017212	4,8466438532	0,0000012557	0,0000164091
NRARP	16,14250548	2,1739455699	0,8832011550	2,4614387761	0,0138381014	0,0370152711
NRAS	557,0433093	1,1204796792	0,2848815156	3,9331427906	0,0000838424	0,0005653901
NRBP1	1579,358896	-0,4662018856	0,1503201280	-3,1013942837	0,0019261163	0,0074413106
NRBP2	243,3687397	1,0232740317	0,4278973645	2,3914006410	0,0167842262	0,0431301423
NRDE2	216,8342274	-0,3431840936	0,1340376134	-2,5603566417	0,0104564795	0,0294074830
NREP	139,5139825	0,9263352553	0,2957526873	3,1321279408	0,0017354423	0,0068341292
NRF1	296,9200868	-0,7180702000	0,1940770897	-3,6999225474	0,0002156653	0,0012426604
NRIP1	581,3649802	0,8452875620	0,2779337534	3,0413274809	0,0023553750	0,0087529949
NRP1	207,8415424	2,8520760935	0,7270700524	3,9226977981	0,0000875630	0,0005866509
NRP2	172,4627675	3,9435376342	0,7511236609	5,2501842769	0,0000001519	0,0000026020
NRSN2	82,91342423	0,9436400960	0,3934496661	2,3983756432	0,0164679672	0,0425040918
NSA2	356,5950112	0,6620865052	0,2469505245	2,6810101452	0,0073407649	0,0219342619
NSF	505,1470729	0,4788394630	0,1418983897	3,3745235857	0,0007394358	0,0033922310
NSMCE3	461,6731363	-0,5279278549	0,1974102889	-2,6742671714	0,0074892760	0,0225062860
NSMCE4A	437,1922556	-0,4493527509	0,1404530283	-3,1993098071	0,0013775705	0,0056324722
NSUN5	263,5706619	-0,6121880144	0,1927376651	-3,1762655999	0,0014916430	0,0060066627
NTSC2	1006,891568	0,5131714585	0,1307476140	3,9249011345	0,0000867654	0,0005827654
NTSDC2	107,1940108	1,6476798677	0,4037537716	4,0809022319	0,0000448612	0,0003379027
NTSDC3	46,4354327	1,1817549900	0,2856100929	4,1376482221	0,0000350884	0,0002737911
NTSE	62,5664294	4,7251348608	0,8569534622	5,5138756880	0,0000000351	0,0000007348
NTAQ1	55,62700879	0,9508255886	0,2774276031	3,4272926629	0,0006096317	0,0028923659
NTN4	150,9632957	5,1972140873	1,2440371279	4,1777805267	0,0000294367	0,0002364073
NUAK2	315,1780759	-0,9722694866	0,3287228701	-2,9577178070	0,0030992571	0,0109836757
NUBP2	503,5310294	-0,6465332549	0,1369288061	-4,7224041388	0,0000023307	0,0000278117
NUCB1	1073,073299	0,4629683599	0,1855551087	2,4950450741	0,0125941131	0,0342516571
NUDT14	201,1798893	0,6220545820	0,2580332488	2,4107525947	0,0159195997	0,0414463853
NUDT15	146,2716815	1,4614551384	0,5350436962	2,7314687543	0,0063052719	0,0194877062
NUDT16	109,6745547	0,7396328135	0,2052654081	3,6050561373	0,0003120855	0,0016843105
NUDT16L2P	35,77040745	1,8408800030	0,4387023412	4,1961937060	0,0000271438	0,0002204284
NUDT22	371,8342598	-0,5556095061	0,1337322504	-4,1546380110	0,0000325803	0,0002570520
NUDT7	25,33101958	-1,4388332019	0,3093878714	-4,6505805007	0,0000033100	0,0000376609
NUDT9	126,2767945	0,7992249899	0,2418739550	3,3043034335	0,0009521272	0,0041636231
NUF2	30,42676638	1,3071444323	0,3806482964	3,4339953153	0,0005947545	0,0028366256
NUFIP1	58,69198466	1,2704332729	0,3349499694	3,7929045799	0,0001488954	0,0009117979
NUMA1	2726,626882	-0,4498825736	0,1452880833	-3,0964932807	0,0019582430	0,0075394596
NUMB	605,6169298	0,3219897416	0,1205964822	2,6699762352	0,0075856616	0,0227502238
NUP42	179,3196852	-0,5131207811	0,1549604237	-3,3108748118	0,0009300481	0,0040856550
NUP50	506,6146705	-0,3373113133	0,1196759677	-2,8185384243	0,0048242837	0,0156403345
NUP58	293,4088263	0,7772306688	0,2639492572	2,9446215419	0,0032334992	0,0113458069
NUP88	694,4043712	-0,4863339259	0,1578740587	-3,0805182942	0,0020664066	0,0078736294
NUPR1	60,43087585	2,5372610231	0,6051553648	4,1927421711	0,0000275601	0,0002232350
NUS1	354,8397483	0,7821380281	0,1952050338	4,0067513253	0,0000615596	0,0004416804
NUSAP1	90,4121614	2,3823760373	0,4204589983	5,6661586009	0,0000000146	0,0000003407
NUTF2	438,5705751	0,3492366798	0,1345031411	2,5964946324	0,0094180385	0,0270037359
NXF1	1917,015552	-0,4683058139	0,1598111624	-2,9303688619	0,0033855877	0,0118032848
NXN	69,9428409	3,6570506004	0,7239069400	5,0518342033	0,0000004376	0,0000065462
NXPH4	61,67896725	-2,3030889431	0,7366882555	-3,1262701516	0,0017703884	0,0069433063
NYNRIN	47,79541157	5,6022236077	0,8225978695	6,8104158979	0,0000000000	0,0000000006
OAF	55,15496139	1,8095137215	0,4111238112	4,4013638949	0,0000107563	0,0001011029
OARD1	243,0598411	-0,4962918262	0,1271988797	-3,9016896625	0,0000955196	0,0006314804
OAS3	457,846003	1,1634271427	0,2843417870	4,0916502462	0,0000428314	0,0003258966
OASL	988,557541	-1,2868338238	0,4775238937	-2,6950145336	0,0070385561	0,0213472206
OAT	644,4454556	0,7341717165	0,1851755779	3,9647329563	0,0000734782	0,0005090149
OBSL1	74,27690148	6,4869399886	0,8452926128	7,6741945813	0,0000000000	0,0000000000
OCEL1	114,7661083	-0,3969420075	0,1671340666	-2,3749916191	0,0175493487	0,0447036777
OCLN	246,353099	2,4330950135	0,6825866659	3,5645217452	0,0003645202	0,0019076370
OCRL	57,25187498	3,9670870397	0,6062862728	6,5432572333	0,0000000001	0,0000000028
ODC1	861,3120757	2,0853047088	0,2881101170	7,2380581233	0,0000000000	0,0000000000
ODF3B	69,1984465	1,8898741873	0,3741005429	5,0517814612	0,0000004377	0,0000065462
ODR4	164,2261094	0,6333894713	0,2201349550	2,8772858793	0,0040111202	0,0135516261
OGFOD2	183,6019587	-1,1964750372	0,1885563761	-6,3454498973	0,0000000002	0,0000000086
OGFOD3	168,1866423	0,4598613584	0,1379400517	3,3337769102	0,0008567534	0,0038263550
OGFR	767,7598461	-0,4579745799	0,1672912504	-2,7375867243	0,0061891799	0,0191685139
OGFRL1	440,3570017	0,9124556238	0,2154531060	4,2350543969	0,0000228496	0,0001918628
OGG1	250,3687735	-0,6375045040	0,2445325663	-2,6070420825	0,0091321501	0,0262768294
OGT	5416,276702	-0,4503335911	0,1589728657	-2,8322349602	0,0046253221	0,0150316400
OTUB2	20,98685719	1,9841333040	0,5198334151	3,8161292316	0,0001355615	0,0008427982
OTUD1	286,5896315	0,9820429374	0,3198922039	3,0699183212	0,0021411732	0,0081046657
OTUD5	942,2809717	-0,5757314706	0,1387388790	-4,1497486118	0,0000332841	0,0002618372
OTUD6B	99,95644538	0,6208830885	0,2514411454	2,4692978851	0,0135378471	0,0363680062
OTUD7A	40,54743628	-1,9353829241	0,3499456486	-5,5306365121	0,0000000319	0,0000006733
OXLD1	275,6154247	-0,9829696053	0,1523174686	-6,4534266115	0,0000000001	0,0000000046
OXR1	288,9017531	1,0326468571	0,3034732256	3,4027949683	0,0006670031	0,0031162312
OXSM	89,29823689	-0,5600865958	0,1934860980	-2,8946629719	0,0037956582	0,0129465809
P2RX1	31,90572384	2,5574276754	0,7744413809	3,3022869526	0,0009589990	0,0041895901
P2RX5-TAX1BP3	78,41337384	-0,9473512983	0,3457561167	-2,7399408208	0,0061450248	0,0190758535
P2RY1	23,08940818	2,0906155621	0,7380203329	2,8404318042	0,0045052505	0,0148093058
P2RY10	288,0173589	-1,0112702254	0,4188536168	-2,4172618207	0,0156377644	0,0408332948
P2RY14	14,72787265	2,5306692173	0,8298718753	3,0494697949	0,0022924568	0,0085760059
P2RY2	28,62503363	4,6535152323	1,0261038843	4,5351307052	0,0000057568	0,0000598739
P3H1	486,694865	-0,7732057026	0,2825161971	-2,7368544191	0,0062029737	0,0192068152
P3H2	22,02111081	5,8481930381	0,8594474549	6,8045863772	0,0000000000	0,0000000006
P3H3	38,67641183	5,7447502543	0,7903911759	7,2682367028	0,0000000000	0,0000000000
P4HA1	727,6016632	2,2533810391	0,4225268831	5,3331069086	0,0000000965	0,0000017656
P4HA2	178,3931632	3,6296768617	0,5050169471	7,1872377401	0,0000000000	0,0000000001
P4HB	4073,958714	0,6451848031	0,2321798562	2,7831217299	0,0053838602	0,0171235251
PAAF1	106,6595734	-0,4800532799	0,1658305244	-2,8968506126	0,0037716955	0,0129009784
PABIR1	131,0198221	-0,4475216903	0,1194923363	-3,7451915658	0,0001802562	0,0010693029
PABPC1	4726,345867	0,8379887384	0,1768534119	4,7383803881	0,0000021543	0,0000258904
PABPC4	516,4612922	0,6541802898	0,2677477876	2,4432705710	0,0145548206	0,0385568545
PABPN1	1337,179082	-0,2067346026	0,0757785609	-2,7281410502	0,0063692367	0,0196538115
PACS1	1184,409216	-0,6351440995	0,1438680680	-4,4117012064	0,0000102562	0,0000974914
PADI2	59,6557331	4,2737826699	0,9395432562	4,5628713404	0,0000050459	0,0000538280
PAFAH1B3	113,1181399	0,9559007085	0,3374717562	2,8326242338	0,0046167608	0,0151109916
PAFAH2	446,5927456	-0,6940976500	0,1745255779	-3,9770557311	0,0000697738	0,0004878730
PAGR1	367,1287593	-0,6433280869	0,1944480284	-3,3085349930	0,0009378550	0,0041145789
PAICS	399,8071328	1,8619271821	0,3675021551	5,0664388112	0,0000004053	0,0000061438
PAIP1	453,6875263	0,6615372611	0,2408946312	2,7462825574	0,0060274827	0,0187627005
PAIP2	1138,557701	-0,5650754664	0,1598643971	-3,3542723326	0,0007957400	0,0036026441
PAK1	273,6548361	1,4086538909	0,2804705775	5,0224658277	0,0000005101	0,0000074291
PAK4	95,96243752	1,4076997556	0,4636173619	3,0429029938	0,0023430785	0,0087193356
PALD1	34,46278885	3,2732145322	0,5378311480	6,0861379204	0,0000000012	0,0000000370
PALM2AKAP2	185,7792365	1,3924327023	0,4309982376	3,2307088403	0,0012348400	0,0051363622
PALS1	109,0187382	1,2453087164	0,3144040240	3,9608548920	0,0000746819	0,0005166259
PAN3	425,4412079	0,4742664877	0,1977620355	2,3981675075	0,0164773282	0,0425200981
PANK2	369,8357611	-0,4695573872	0,1483202932	-3,1656181655	0,0015475379	0,0061956753
PANK4	345,722607	-0,9512976774	0,1653024251	-5,7548924447	0,0000000087	0,0000002149
PANX1	153,267381	0,9372954944	0,2908341267	3,2250015300	0,0012597201	0,0052171792
PAOX	63,58413836	-1,4255544506	0,4812264368	-2,9623361092	0,0030531430	0,0108459501
PAPLN	37,29645767	2,0645270403	0,3577457940	5,7709330904	0,0000000079	0,0000001991
PAPOLG	306,7878174	-0,7135636617	0,1964690687	-3,6319389431	0,0002812997	0,0015460571
PAPSS1	440,6760008	0,6481653555	0,2221063961	2,9182651516	0,0035196490	0,0121792206
PAPSS2	161,3188713	3,4787534862	0,6971587099	4,9898946208	0,0000006041	0,0000085866
PAQR3	96,89837057	0,8391085665	0,2600789360	3,2263611167	0,0012537506	0,0051976347
PAQR6	35,85895466	0,9691417243	0,2928860096	3,3055524225	0,0009478837	0,0041505040
PAQR7	23,87613393	1,9896371644	0,7782084627	2,5566891904	0,0105673590	0,0296545537
PARD3	119,8932815	5,2155678644	0,8935884552	5,8366553796	0,0000000053	0,0000001420
PARD6A	42,58585773	-1,1645591066	0,3063068062	-3,8019417261	0,0001435665	0,0008843959
PARD6B	68,99077562	1,7299838530	0,4836047845	3,5772885386	0,0003471769	0,0018368714
PARL	360,5873038	0,2833763627	0,1104609285	2,5653990656	0,0103057200	0,0290894258
PARP10	1538,324813	-0,7442489216	0,2168761689	-3,4316768195	0,0005998619	0,0028548236
PARP12	856,2552932	-0,6379344328	0,2211348410	-2,8848209986	0,0039163627	0,0132815097
PARP15	866,1199266	-0,6272874004	0,2452652623	-2,5556494663	0,0105985657	0,0296545537
PARP16	163,5378683	-0,7359900329	0,1519753489	-4,8428250909	0,0000012801	0,0000166219
PARP3	129,9091683	0,9536282842	0,3029714762	3,1475843737	0,0016462559	0,0065210724
PARPBP	30,26502694	1,0952964536	0,3140308398	3,4878627024	0,0004868980	0,0024128665
PARS2	42,98314	-1,5996195711	0,2869565606	-5,5744314319	0,0000000248	0,0000005391
PARVA	120,5714285	4,9880348276	0,6826408440	7,3070489764	0,0000000000	0,0000000000
PARVB	62,06935865	1,1466496348	0,4219012948	2,7178149227	0,0065714585	0,0201558072
PASK	129,6121984	-0,3974780436	0,1587943470	-2,5031057531	0,0123108753	0,0336046200
PATJ	285,8721825	1,6500083852	0,3207470981	5,1442659802	0,0000002686	0,0000043629
PATL1	294,0477982	0,8552726131	0,3037056396	2,8161235803	0,0048606966	0,0157271576
PAWR	126,1580207	2,2468502210	0,4640236902	4,8416713816	0,0000012875	0,0000166965
PAX8	457,2714809	5,5463334929	0,9699484256	5,7181736127	0,0000000108	0,0000002612
PAXIP1-DT	69,61173463	-0,9645885347	0,1796780061	-5,3684285330	0,0000000794	0,0000014869
PAXX	818,8872255	-1,0080584454	0,2655647175	-3,7959065298	0,0001471049	0,0009028875
PBDC1	217,3884343	-0,4869809222	0,1808088656	-2,6933464826	0,0070738714	0,0214398354
PBK1	44,35483066	3,3146850900	0,5628822004	5,8887722644	0,0000000039	0,0000001078
PBX3	119,7585659	0,3510021940	0,1493040159	2,3509226588	0,0187269259	0,0471060404
PBXIP1	1784,318521	-0,9648767717	0,2472097226	-3,9030694752	0,0000949803	0,0006285948
PC	62,70958913	1,9446930574	0,5452232117	3,5667826666	0,0003613910	0,0018934692
PCBD1	120,5551853	1,9921534435	0,4336557237	4,5906825397	0,0000044180	0,0000481115
PCBP1	3611,316158	-0,3996034605	0,1677398352	-2,3822812270	0,0172057520	0,0440284073
PCBP3	11,86734895	3,4894615501	0,8245538309	4,2319644403	0,0000231669	0,0001937301
PCCA	124,9098644	0,6765878043	0,2264555702	2,9877288909	0,0028105893	0,0101248083
PCDHGC3	53,38654338	6,1117958035	1,1261940067	5,4269476613	0,0000000573	0,0000011141
PCED1B	220,9062724	-1,2764956844	0,2610818789	-4,8892542433	0,0000010122	0,0000135833
PCGF1	152,9677221	-0,6857818428	0,1288456675	-5,3307807083	0,0000000978	0,0000017836
PCGF3-AS1	88,71147377	-1,2145564532	0,2870286803	-4,2314811601	0,0000232157	0,0001939689
PCGF5	563,3281322	0,3319157028	0,1171781488	2,8325733620	0,0046174969	0,0151109916
PCGF6	72,00653309	-0,4697792803	0,1916711160	-2,4509654354	0,0142473634	0,0379183900
PCID2	629,9002211	-0,9261392745	0,2236831082	-4,1404381954	0,0000346688	0,0002708887
PCIF1	769,8329005	-0,8377685706	0,2245367080	-3,7310984831	0,0001906457	0,0011200804
PCK2	187,1208232	1,1325608708	0,4570050276	2,4782240948	0,0132038187	0,0356087756
PCNT	666,8477275	-0,6126783344	0,1318572163	-4,6465362427	0,0000033755	0,0000381485
PCYT2	393,071024	-0,6668867264	0,1891798441	-3,5251468225	0,0004232481	0,0021473501
PDCD2	568,8569515	-0,3694252842	0,1076367322	-3,4320853197	0,0005986337	0,0028500651
PDCD4	2987,110322	-0,7262948353	0,3094303250	-2,3548100700	0,0185321752	0,0467034409
PDCD6IP	1257,311242	0,5637853838	0,1689865212	3,3362150502	0,0008492744	0,0038040711
PDCD6IP-DT	22,84999618	1,8837327639	0,5216022261	3,6114354380	0,0003045069	0,0016513549
PDCD7	211,3480234	-0,8618983284	0,1481479914	-5,8178198721	0,0000000060	0,0000001567
PDCL3	153,846774	0,5833786226	0,1953857781	2,9857961267	0,0028297786	0,0098563930
PDE1B	96,93201188	2,2032658281	0,6601140663	3,4997980582	0,0004656107	0,0023245996
PDE3B	592,7947122	-1,5038996081	0,5260102689	-2,8571678103	0,0042743979	0,0142127954
PDE6D	218,8060226	-0,7905526587	0,1447249238	-5,5039117515	0,0000000371	0,0000007680
PDE6G	83,29045281	-0,8249636235	0,3255366468	-2,5328548874	0,0112815439	0,0312657691
PDE7B	25,3047895	-1,5153251070	0,5471836714	-2,7693171166	0,0056173931	0,0177446869
PDE8A	155,6289451	0,4322669034	0,1655665324	2,6108350353	0,0090321451	0,0261728037
PDGFB	31,8741239	2,3303101194	0,8398319724	2,7770484216	0,0054855006	0,0174178650
PDHB	737,7670705	-0,3105954030	0,1188351246	-2,6136656590	0,0089576384	0,0260073936
PDHX	194,3973636	0,5055069833	0,1668944735	3,0290823698	0,0024529698	0,0090482252
PDIA3	4744,832764	0,5490015180	0,1923274345	2,8545348507	0,0043102616	0,0143108342
PDIA5	41,11761853	3,2861788579	0,4806122825	6,8374837416	0,0000000000	0,0000000005
PDIA6	2038,667809	0,9639446158	0,2096580112	4,7407900622	0,0000021289	0,0000256761
PDK1	199,2503572	0,7409009058	0,2538673859	2,9184564347	0,0035176903	0,0121786120
PDK2	382,5646484	-0,8033355276	0,1975339600	-4,0668223721	0,0000476585	0,0003557870
PDK4	260,0628415	3,4497423172	0,7907802835	4,3624536284	0,0000128612	0,0001176030
PDLIM1	568,388897	2,1949510908	0,6003283381	3,6562449165	0,0002559368	0,0014294232
PDLIM3	53,31360138	3,6839581404	0,7396834735	4,9938741387	0,0000005915	0,0000084339
PDLIM4	38,88227759	3,7982799678	0,8663110290	4,3844268880	0,0000116290	0,0001078747
PDLIM5	390,9469639	0,6329988147	0,2573808219	2,4622273389	0,0138077115	0,0369413231
PDLIM7	112,5117298	2,1806106430	0,3607262987	6,0450542322	0,0000000015	0,0000000462
PDP1	374,2235713	1,4271343434	0,4073871290	3,5031404822	0,0004598069	0,0023024556
PDP2	112,3228235	-1,2603810503	0,2052914190	-6,1397717155	0,0000000008	0,0000000277
PDRG1	195,2282719	-0,4598486541	0,1813078647	-2,5814400616	0,0095575289	0,0273536475
PDSS1	40,20669308	0,5800181991	0,2397604573	2,4608653392	0,0138602377	0,0370525053
PDXDC2P-NPIPB14P	248,8462126	-0,4925178032	0,2080698605	-2,3670790283	0,0179291039	0,0454816596
PDXK	648,571496	0,7861167512	0,1477962253	5,3191389994	0,0000001043	0,0000018862
PDXP	166,9411093	-1,3247781179	0,2589517955	-5,1159284563	0,0000003122	0,0000048524
PDZD2	35,17223156	1,3808837649	0,4969526360	2,7787152697	0,0054574339	0,0173377651
PDZD8	395,2109773	0,8220405844	0,2424305639	3,3920882713	0,0006936209	0,0032182480
PDZK1	19,7070005	2,6072563658	[illegible]	[illegible]	[illegible]	[illegible]

PEX16	267,3466603	-0,6786364910	0,2006829981	-3,3816342071	0,0007205603	0,0033203495
PFDN5	1567,957833	0,3096529827	0,1226599969	2,5244822309	0,0115868862	0,0319327373
PFKFB2	82,96318047	1,6690824106	0,5457790000	3,0581653205	0,0022269668	0,0083752370
PFKFB3	461,8765122	1,0109904507	0,3804670216	2,6572354328	0,0078784402	0,0234610188
PFKFB4	94,76283093	1,2723295640	0,3610733365	3,5237427840	0,0004254971	0,0021575834
PFKM	117,7319589	0,7285191439	0,3032432568	2,4024248768	0,0162867794	0,0421172048
PFKP	685,7174599	0,8878802659	0,3339126451	2,6590195934	0,0078369405	0,0233568131
PFN2	87,43451666	3,5076611888	0,6346899065	5,5265745402	0,0000000327	0,0000006868
PGAP6	652,1846844	-0,5356308512	0,1852141642	-2,8919540435	0,0038285394	0,0130825440
PGBD1	24,37637952	0,9255083634	0,3817290274	2,4245165984	0,0153287825	0,0402350649
PGBD2	126,0327205	-1,3779318668	0,2504108785	-5,5026837303	0,0000000374	0,0000007710
PGD	811,2673847	0,5599903996	0,2251721911	2,4869429789	0,0128846049	0,0348947247
PGGT1B	222,5473372	0,5500229978	0,2294860931	2,3967596050	0,0165407717	0,0426447364
PGK1	5047,222212	1,0358997758	0,2076846559	4,9878493489	0,0000006106	0,0000086688
PGLS-DT	22,52425978	-0,9714502298	0,3912155725	-2,4831583863	0,0130223172	0,0352180810
PGM3	287,2216717	0,8415558845	0,2537707093	3,3162058436	0,0009124856	0,0040321917
PGP	286,5538873	-0,6176914892	0,1852623434	-3,3341449092	0,0008556207	0,0038242571
PGRMC1	317,3920841	1,4140215722	0,3920207880	3,6070066063	0,0003097498	0,0016764101
PHACTR1	52,09765581	1,5065463343	0,3969287621	3,7955081063	0,0001473414	0,0009035148
PHAF1	200,2349163	-0,4197011726	0,1728099809	-2,4288859499	0,0151536521	0,0398377401
PHAX	253,7672341	-0,5189624602	0,1471505394	-3,5267452123	0,0004207014	0,0021368456
PHETA2	44,06251936	1,1497153217	0,2784257663	4,1293423982	0,0000363802	0,0002827239
PHF20	463,4839534	-0,3190332762	0,0929488866	-3,4323517791	0,0005983709	0,0028488819
PHF21A	610,4500762	-0,2983235271	0,1253934273	-2,3791001929	0,0173549581	0,0443175366
PHF23	202,8238633	-0,8916230529	0,2151765847	-4,1436804697	0,0000341776	0,0002677709
PHGDH	121,5118311	2,5738889987	0,6837967001	3,7640978762	0,0001671514	0,0010030575
PHKB	449,4739647	0,7911124384	0,1949696497	4,0576184011	0,0000495757	0,0003676522
PHLDA1	373,8114219	1,3399920853	0,5173463370	2,5901257812	0,0095940870	0,0274466335
PHLDA2	70,13573768	4,6617445037	0,9550320443	4,8812440711	0,0000010542	0,0000140907
PHLDA3	77,92541452	3,2325369494	0,8452310939	3,8244419663	0,0001310686	0,0008208323
PHLDB1	68,04251473	1,3677933865	0,2906105262	4,7066202461	0,0000025186	0,0000297108
PHLDB2	275,540311	-0,7740929475	0,3231802468	-2,3952359565	0,0166096726	0,0427547168
PHRF1	509,3484797	-0,6746202974	0,1364562321	-4,9438584584	0,0000007659	0,0000105953
PHTF1	209,857466	-0,5987831047	0,1981655775	-3,0216302562	0,0025141744	0,0092410671
PHTF2	193,1837879	1,1273469103	0,2767127017	4,0740699770	0,0000461986	0,0003464251
PHYH	91,530569	0,5245065114	0,2034007857	2,5786847859	0,0099177240	0,0281927275
PHYHD1	55,21867484	2,0974466216	0,7323423589	2,8640247365	0,0041829534	0,0139882120
PI4K2B	128,1284673	0,6015061677	0,1907676028	3,1530834318	0,0016155563	0,0064266181
PIAS1	826,7168741	-0,4618113340	0,1486845297	-3,1059810650	0,0018964886	0,0073470118
PIAS3	435,1137382	-0,4577183907	0,1657162187	-2,7620615187	0,0057437655	0,0180273723
PIBF1	166,8966608	0,5062251870	0,2006532033	2,5228861469	0,0116396079	0,0320583431
PIDD1	283,5046664	-0,6082236585	0,1825618024	-3,3316041490	0,0008634698	0,0038484450
PIGA	153,8580852	1,2656869173	0,3841263774	3,2949752785	0,0009843034	0,0042737818
PIGBOS1	90,55813432	-0,5623974692	0,1648219128	-3,4121523020	0,0006445209	0,0030301263
PIGC	392,555603	-0,6042775433	0,1705838212	-3,5424082949	0,0003964913	0,0020392710
PIGH	235,5224441	-0,3946826503	0,1496079479	-2,6367760368	0,0083698074	0,0246084770
PIGL	180,6425213	-0,8827751539	0,1694508398	-5,2096239525	0,0000001892	0,0000031522
PIGV	218,5353742	-1,1332230830	0,2059154526	-5,5033416326	0,0000000373	0,0000007693
PIGW	96,17033788	0,8978792924	0,3793994902	2,3665798601	0,0179533005	0,0455344586
PIGX	214,3495156	-0,8685548963	0,2031288387	-4,2758817607	0,0000190382	0,0001645822
PIK3CB	238,201184	1,3106468557	0,1827232139	7,1728524677	0,0000000000	0,0000000001
PIK3CD	2927,504486	-0,7475606579	0,2787148150	-2,6821705116	0,0073146167	0,0220552734
PIK3IP1	3045,838621	-1,3998232307	0,3436142568	-4,0738217436	0,0000462479	0,0003466016
PIK3R4	273,2658602	-0,4477031518	0,1407684211	-3,1804231963	0,0014706012	0,0059424672
PIK3R6	133,7356434	2,5031833703	0,4448186210	5,6274248699	0,0000000183	0,0000004173

RAD23B	524,370304	1,1847320793	0,3718131646	3,1863839819	0,0014407321	0,0058427501
RAD50	286,4493208	0,4482571512	0,1911957682	2,3444920582	0,0190529652	0,0478188281
RAD51	31,67738047	1,1563914859	0,4230587625	2,7334062982	0,0062682956	0,0193867782
RAD51AP1	29,41241563	0,9991955380	0,4302312054	2,3224617961	0,0202080832	0,0501661380
RAD51-AS1	198,322018	-0,5201814404	0,2006138283	-2,5929490738	0,0095156860	0,0272513050
RAD51D	211,8349135	-0,5508887144	0,1912786213	-2,8800328587	0,0039763373	0,0134577570
RAD54L	15,85096545	1,3297106098	0,4870216465	2,7302905722	0,0063278524	0,0195440325
RAD54L2	564,2675598	-0,4901098073	0,1895050806	-2,5862620981	0,0097022122	0,0276738975
RADIL	82,22054652	-2,3043957546	0,6764263138	-3,4067210392	0,0006574829	0,0030749547
RAI14	234,8466662	4,7698475372	0,6828998965	6,9847001796	0,0000000000	0,0000000002
RALB	416,2201576	0,9939758548	0,2811711421	3,5351268533	0,0004075794	0,0020822197
RALGAPA2	341,7127609	0,4399403755	0,1646925817	2,6712821206	0,0075562109	0,0226821140
RALGDS	2652,048634	-0,9478779753	0,2807545997	-3,3761798824	0,0007349990	0,0033753281
RAMAC	325,2226585	-0,5339796635	0,1990427670	-2,6827383452	0,0073022097	0,0220277226
RANBP1	320,1188291	0,4692733305	0,1548745145	3,0300229311	0,0024453517	0,0090300231
RANBP10	245,9647511	-0,6493057340	0,1534854361	-4,2304061565	0,0000233270	0,0001945260
RANBP17	21,85855244	1,1879858790	0,5089466206	2,3342061775	0,0195849286	0,0489258723
RANBP2	768,6756757	0,3386890378	0,1282134701	2,6416026149	0,0082514807	0,0243296671
RANGRF	226,8271032	-0,5897647014	0,1757247974	-3,3559927245	0,0007908065	0,0035827181
RAP1B	3509,05174	-0,7262229147	0,2177431189	-3,3352278522	0,0008522953	0,0038150632
RAP1GAP2	595,1927621	-1,6215756295	0,4550280720	-3,5636826150	0,0003656881	0,0019102578
RAP1GDS1	401,2397736	0,5977280996	0,1983072194	3,0141519881	0,0025769867	0,0094281710
RAPGEF1	1710,241752	-0,8572923819	0,2237416292	-3,8316176781	0,0001273035	0,0008022200
RAPGEF2	195,3070477	0,9911742559	0,2696691028	3,6823478081	0,0002310958	0,0013163563
RAPGEF3	148,6025843	4,4798997782	1,1058127843	4,0512280575	0,0000509495	0,0003768025
RAPGEF5	14,62794903	5,8098208876	1,3074361093	4,4436747970	0,0000088435	0,0000861939
RAPH1	58,32763373	4,7251906114	0,7412601886	6,3745371515	0,0000000002	0,0000000072
RARA-AS1	16,19125637	2,5999295617	0,4778231300	5,4411965388	0,0000000529	0,0000010430
RARB	21,32076662	2,0638118177	0,5679283783	3,6339297288	0,0002791369	0,0015366801
RARRES1	151,9038216	9,1526309421	1,2415419930	7,3719870977	0,0000000000	0,0000000000
RARRES2	58,36079282	6,7028951377	0,8291075879	8,0844896583	0,0000000000	0,0000000000
RARS1	452,6171274	0,5623995912	0,1528446753	3,6795497801	0,0002336461	0,0013271824
RARS2	368,6563972	0,2676828957	0,0996595342	2,6857730961	0,0072362212	0,0218829713
RASA1	386,4858795	0,5947008817	0,2013207984	2,9539962405	0,0031368783	0,0110907228
RASA3	1693,323008	-0,6533606206	0,2573082069	-2,5392140753	0,0111101818	0,0309292754
RASAL2	29,55532515	2,9241445753	0,7522327739	3,8872868589	0,0001013709	0,0006633217
RASAL3	2471,498543	-1,3422589314	0,3230121971	-4,1554434895	0,0000324657	0,0002564487
RASD1	43,99606452	4,4700027521	1,2073519150	3,7023196772	0,0002136372	0,0012325575
RASEF	49,16270882	3,7691362058	0,9451217853	3,9879582341	0,0000666444	0,0004703899
RASGRP1	1053,471154	-0,6860359514	0,2581943528	-2,6570525033	0,0078827165	0,0234675283
RASSF2	349,9901081	0,9830563968	0,3781703415	2,6010934460	0,0092927138	0,0267607889
RASSF5	3336,651821	-0,9222208353	0,2958600941	-3,1167471305	0,0018285835	0,0071278274
RASSF8	43,0947762	3,6716702969	0,3765101645	9,7518490676	0,0000000000	0,0000000000
RAVER2	72,25537992	1,4878114654	0,3854063481	3,8603709380	0,0001132150	0,0007284294
RB1	418,1118332	0,7047349578	0,2405015055	2,9302725418	0,0033866483	0,0118032848
RBBP4	1284,265344	-0,3797380634	0,0898655067	-4,2256042099	0,0000238301	0,0001978732
RBBP8	121,2189434	2,0801740018	0,3625733970	5,7372465580	0,0000000096	0,0000002255
RBFA	96,83266216	0,7435974349	0,2912062427	2,5535078781	0,0106643866	0,0298725991
RBFOX2	106,3563363	2,2392843724	0,5706557463	3,9240547161	0,0000870709	0,0005842348
RBFOX3	15,77823416	-1,0439272771	0,4022882721	-2,5949669789	0,0094600007	0,0271091355
RBKS	40,59371543	1,4928830788	0,5114812334	2,9187465822	0,0035144405	0,0121698919
RBL1	183,8229289	-0,3851922755	0,1520725111	-2,5328856129	0,0113127863	0,0313441959
RBM10	569,3973835	-0,3721087445	0,1107025808	-3,3613375764	0,0007756596	0,0035235837
RBM12	589,5529767	0,7443485416	0,1310515086	5,6798166602	0,0000000135	0,0000003179
RBM14	594,4048164	-0,6780019690	0,1298712298	-5,2205709473	0,0000001784	0,0000030011
RBM23	1026,261497	-0,8636149697	0,1759450678	-4,9084346733	0,0000009181	0,0000124819
RBM3	1855,060222	1,3065696317	0,3274445513	3,9902011706	0,0000660173	0,0004664528
RBM33-DT	43,02639104	-0,6958338637	0,2948849739	-2,3596789436	0,0182907571	0,0462248574
RBM4	1260,932689	-0,6719670807	0,1533018981	-4,3832926329	0,0000116899	0,0001083650
RBM47	315,732138	4,4017390868	0,6280836957	7,0082066655	0,0000000000	0,0000000002
RBM48	223,7968376	-0,9318356984	0,1700940956	-5,4783529964	0,0000000429	0,0000008689
RBM4B	227,3050271	-1,0461923942	0,2413087000	-4,3437820273	0,0000140050	0,0001270269
RBM5	1435,608448	-0,5773859140	0,1364288197	-4,2321403602	0,0000231478	0,0001937301
RBMS2	129,7045811	1,5162846154	0,3549665769	4,2716252378	0,0000194053	0,0001673261
RBMS3	16,79639092	1,3228194877	0,4536988805	2,9157102497	0,0035487991	0,0122667785
RBMX	1521,766839	0,3513371504	0,1220579195	2,8784461649	0,0039963948	0,0135120509
RBMXL1	177,6283358	0,9392694626	0,3268316635	2,8738820369	0,0040549855	0,0136616320
RBP1	70,36231005	6,8123775217	1,2743963883	5,3455719089	0,0000000901	0,0000016687
RBPMS	118,9868526	2,4498736819	0,7073894307	3,4590192837	0,0005421461	0,0026343994
RC3H2	571,3659422	0,3016972737	0,0847588668	3,5594774335	0,0003715935	0,0019348735
RCAN2	54,0302737	3,6899809430	0,8324410444	4,4327234558	0,0000093050	0,0000898462
RCAN3	135,5512653	2,1202740551	0,3621030043	5,8554445277	0,0000000048	0,0000001294
RCBTB1	196,0687916	0,5822678627	0,1659754584	3,5084050433	0,0004510610	0,0022847200
RCBTB2	340,4274003	-0,9321982974	0,3629832357	-2,5681600385	0,0102239942	0,0288864320
RCC1	227,0482376	0,8258882123	0,3286244873	2,5131874159	0,0119642363	0,0328717331
RCCD1	66,13434459	1,1866012375	0,4503950184	2,6345789560	0,0084241709	0,0247412917
RCE1	232,2696423	-0,4713041743	0,1445632022	-3,2601946228	0,0011133579	0,0047198861
RCN1	362,1922479	2,0024449864	0,4115969110	4,8650632274	0,0000011442	0,0000151556
RCN3	30,42648831	3,5388221814	0,6821179505	5,1879915768	0,0000002126	0,0000034766
RDH11	679,6609339	-0,5481782010	0,1508238302	-3,6343252728	0,0002787091	0,0015348525
RDH14	172,5810583	-0,5421874727	0,1668721613	-3,2491187771	0,0011576313	0,0048677655
RDH5	31,24605846	-1,2433306259	0,3592440249	-3,4609639680	0,0005382448	0,0026192248
RDX	201,0466149	0,9277582074	0,2712006030	3,4209297364	0,0006240745	0,0029494534
REC8	393,4253784	1,3285870004	0,4323208174	3,0731506487	0,0021181153	0,0080308386
REEP2	14,38586019	-1,1466658043	0,4906602559	-2,3369852978	0,0194399486	0,0486088998
REEP3	223,6215949	1,1979241822	0,2918023871	4,1052583364	0,0000403863	0,0003105711
REEP4	242,6137935	-0,4764304404	0,1790628828	-2,6606878712	0,0077981209	0,0232924925
RELA-DT	44,63537749	-0,8818361976	0,2407828433	-3,6623714409	0,0002498912	0,0014020572
RELB	303,8211009	-1,2416598647	0,2937022032	-4,2276154253	0,0000236181	0,0001967201
RELL1	94,74645502	0,5721749062	0,2435350570	2,3494560216	0,0188008649	0,0472478794
RELL2	55,87733308	-0,8942484129	0,2975966955	-3,0049003443	0,0026566788	0,0096776445
REPIN1	659,8519888	-0,6636404868	0,1836770787	-3,6130827617	0,0003025781	0,0016422181
REPS1	358,3761472	0,4640275759	0,1666806953	2,7839311272	0,0053704437	0,0170929703
REPS2	18,68736882	2,2370067600	0,4700025261	4,7608398585	0,0000019279	0,0000235475
RERE	304,6576844	1,4161152418	0,4758378800	2,9760456267	0,0029199142	0,0104360182
RESF1	3011,587167	-0,9836444892	0,2518422103	-3,9057967603	0,0000939154	0,0006236318
REST	820,3443126	-0,6360285888	0,1727042597	-3,6862355914	0,0002275957	0,0013016467
RETREG1	74,94175198	1,1431346714	0,3262048500	3,5043460309	0,0004577302	0,0022937632
RETREG3	1312,043109	-0,8266724712	0,2107879051	-3,9218211826	0,0000878822	0,0005887976
REXO1BD	575,404485	-0,5498806943	0,1900486482	-2,8860035396	0,0039016777	0,0132751723
REXO5	16,31277944	1,6993858407	0,4636539224	3,6652032018	0,0002471423	0,0013918557
RFC3	93,11408828	0,9976247346	0,3631264568	2,7473204332	0,0060084400	0,0187120784
RFFL	526,7423795	-0,8510884066	0,2693034500	-3,1603323560	0,0015758926	0,0062886199
RFK	160,8005893	1,1195105414	0,4051128012	2,7634528777	0,0057193175	0,0179780507
RFTN1	1111,169208	-0,8499542303	0,2657994583	-3,1977278502	0,0013851494	0,0056517524
RFX5	586,5648323	-0,5004961464	0,1547664361	-3,2338804126	0,0012212058	0,0050858385
RGL1	315,0185433	1,9547277841	0,6673132581	2,9292506335	0,0033978033	0,0118329707
RGPD1	90,06185503	-1,5277821278	0,5424413474	-2,8164927602	0,0048551137	0,0157126710
RGPD6	158,8633452	0,9157968256	0,3254032221	2,8143449832	0,0048876744	0,0157878805
RGS1	1320,174918	1,4956130985	0,5069930622	2,9499675836	0,0031780727	0,0112058201
RGS12	67,3646676	0,8892339157	0,3805468866	2,3366980731	0,0194597523	0,0486038898
RGS14	545,3885447	-0,6993097782	0,2348589554	-2,9775807210	0,0029055208	0,0103900931
RGS2	782,6343616	-1,4394239656	0,2024239367	-7,1059973881	0,0000000000	0,0000000001
RHBDF1	45,28920717	5,2756194873	0,9606978942	5,4915019948	0,0000000399	0,0000008159
RHBDF2	987,5918566	-0,5725277034	0,2286330354	-2,5041337630	0,0122751616	0,0335343364
RHD	22,6889705	-1,1240330885	0,3899401331	-2,8825786141	0,0039443470	0,0133696576
RHEB	424,0832234	0,5474945446	0,1980134899	2,7649495490	0,0056931600	0,0179125605
RHEBL1	39,74166831	-0,9775112634	0,2946524122	-3,3196470002	0,0009013134	0,0039906836
RHNO1	207,6597663	0,5087309611	0,2038887063	2,4342509708	0,0149226443	0,0393609235
RHOA	4845,323274	0,4634375958	0,1866964632	2,4824381364	0,0130486723	0,0352708254
RHOB	748,8764075	3,8533257681	0,6054043891	6,3648791429	0,0000000002	0,0000000076
RHOBTB1	26,05834287	3,4459627630	0,7580715711	4,5329188014	0,0000058099	0,0000062407
RHOBTB3	208,8025262	-0,9491755549	0,3446680390	-2,7538832945	0,0058892765	0,0183986329
RHOQ	274,4543161	0,7113125009	0,2830435612	2,5130849043	0,0119680528	0,0328755082
RHOT2	1708,483411	-0,7148838046	0,1722157961	-4,1510931089	0,0000330891	0,0002605461
RHOU	44,448653	2,7676015510	0,6298246261	4,2495881364	0,0000214154	0,0001818623
RHPN2	153,8768888	7,0472295698	1,0931251026	6,4469646388	0,0000000001	0,0000000047
RIC8A	1264,800504	-0,5842609686	0,1551089933	-3,7667768732	0,0001653687	0,0009932451
RIGI	387,7475484	-0,5457026519	0,1720587546	-3,1623981695	0,0015647545	0,0062562207
RIMBP3B	13,9885823	-2,3586706310	0,8827787030	-2,6718707603	0,0075429687	0,0226474143
RIMKLB	128,2485429	1,4121682206	0,2946067933	4,7934003986	0,0000016398	0,0000203987
RIN1	38,46700317	2,6413558257	0,5508540271	4,7950191803	0,0000016266	0,0000203098
RIN2	86,93758734	4,0246427041	0,7473864990	5,3849550524	0,0000000725	0,0000013641
RIN3	1230,247806	-0,6048311644	0,2496776609	-2,4224480565	0,0154163295	0,0404018294
RINL	403,8965355	-1,1698032947	0,2182307205	-5,3585640560	0,0000000839	0,0000015585
RIOK3	855,6092669	0,7721978605	0,2270542399	3,4009400608	0,0006715455	0,0031337756
RIOX1	228,6263256	-1,0300738077	0,2360545413	-4,3637110402	0,0000127874	0,0001171675
RIOX2	133,175871	1,4027179661	0,3213408225	4,3652031363	0,0000127005	0,0001165292
RIPK2-DT	125,4371158	0,7714439030	0,2440888984	3,1605297464	0,0015748252	0,0062874741
RIPOR2	1645,948555	-0,9035715477	0,2625210288	-3,4419315949	0,0005776403	0,0027767996
RITA1	172,9656838	-0,6945420907	0,1800212948	-3,8581107386	0,0001142669	0,0007327452
RIUM	346,878696	0,7881492068	0,1610806194	4,8928826800	0,0000009936	0,0000133745
RMDN1	266,8709387	0,6200000184	0,1760436074	3,5252620168	0,0004230625	0,0021472178
RMDN2	64,73410181	-0,8918296443	0,3092327965	-2,8840073058	0,0039264964	0,0133326544
RMI2	25,33757381	1,6673732856	0,6444246099	2,5873830082	0,0096708030	0,0276132813
RMND5B	171,1341864	0,9231932185	0,2434752472	3,7917323962	0,0001495995	0,0009148610
RN7SK	46,0357802	-1,7577262985	0,5754004025	-3,0547880934	0,0022521959	0,0084526853
RN7SL605P	27,35241001	-0,7014886452	0,2955050052	-2,3738438362	0,0176030407	0,0448150042
RNA5-8SN1	10,51408784	-1,2677169710	0,3780918598	-3,3529337860	0,0007995983	0,0036140341
RNA5-8SN2	10,51408784	-1,2677169710	0,3780918598	-3,3529337860	0,0007995983	0,0036140341
RNA5-8SN3	10,51408784	-1,2677169710	0,3780918598	-3,3529337860	0,0007995983	0,0036140341
RNA5-8SP10	10,51408784	-1,2677169710	0,3780918598	-3,3529337860	0,0007995983	0,0036140341
RNASE1	690,4583898	6,7239481168	1,0594727762	6,3465039098	0,0000000002	0,0000000085
RNASE4	19,87413218	3,6690644388	0,7479114199	4,9057473132	0,0000009307	0,0000126286
RNASE6	104,0834737	3,5088837798	0,6206738557	5,6533455493	0,0000000157	0,0000003650
RNASEH2C	422,8940808	-0,7347439354	0,1731668312	-4,2429715348	0,0000220579	0,0001864949
RNASET2	1222,191397	1,2658533971	0,2932944824	4,3193904862	0,0000156461	0,0001396992
RND3	262,1190536	9,6846750170	1,3688745509	7,0742549674	0,0000000000	0,0000000001
RNF113A	168,4327023	-0,5699080856	0,1988480328	-2,8645353859	0,0041762149	0,0139760947
RNF126	465,9701785	-0,4852906184	0,1848659755	-2,6250932166	0,0086625248	0,0253028256
RNF130	748,6647243	0,8816832939	0,3181319353	2,7714391299	0,0055809606	0,0176501542
RNF138	315,8209038	0,6791962639	0,1869555989	3,6329281812	0,0002802230	0,0015413985
RNF139	807,8262282	0,5679634513	0,2026762779	2,8023183435	0,0050736781	0,0162948678
RNF14	436,523495	-0,3263617570	0,1127684205	-2,8940625529	0,0038029239	0,0130181475
RNF141	308,7722394	1,7338372587	0,2508690166	6,9113248098	0,0000000000	0,0000000003
RNF144B	160,5543384	2,8554826109	0,5556742758	5,1295393641	0,0000002905	0,0000045725
RNF145	402,9100896	1,4116238614	0,2139990442	6,5964026457	0,0000000000	0,0000000020
RNF149	1079,401611	-0,4484317238	0,1637862643	-2,7379080023	0,0061831370	0,0191542057
RNF157	92,43026834	1,1755741616	0,3780655545	3,1094469452	0,0018743794	0,0072748406
RNF165	229,1973823	1,3240786179	0,4596085682	2,8808822457	0,0039656251	0,0134248777
RNF166	1017,780409	-0,8727431957	0,3516520573	-2,4818351916	0,0130707713	0,0353024412
RNF168	415,0088895	-0,6338028518	0,1685118460	-3,7588461653	0,0001706305	0,0010188084
RNF19A	1038,691094	-0,6823311100	0,2048967204	-3,3301352090	0,0008679894	0,0037995798
RNF207	86,3525144	1,6849229858	0,5552144738	3,0525555003	0,0022690177	0,0085048671
RNF216	851,4287495	-0,5361481596	0,1258405687	-4,2605350990	0,0000203938	0,0001743968
RNF216P1	172,9479714	-0,4980794921	0,1839154510	-2,7082981930	0,0067649610	0,0206617597
RNF217	74,76640818	2,6226040773	0,5394009902	4,8620675990	0,0000011617	0,0000153293
RNF25	198,8552977	-0,4806484476	0,1869742183	-2,5706709450	0,0101501719	0,0287381812
RNF31	614,3208782	-0,5148518602	0,1449004092	-3,5517626433	0,0003826599	0,0019810008
RNF34	446,3536233	-0,7331081372	0,1917111584	-3,8240243458	0,0001312909	0,0008219424
RNF41	393,057809	-0,6022390721	0,1715129948	-3,5113693558	0,0004458672	0,0022423281
RNF5	414,8850366	-0,6365336547	0,1375785683	-4,6412290991	0,0000034634	0,0000389455
RNLS	29,80057896	1,1739279006	0,4166216058	2,7999222285	0,0051114920	0,0163887338
RNPEPL1	1081,467639	-0,5342948883	0,1907084915	-2,8015155787	0,0050762367	0,0162991759
ROBO	713,6270879	-0,6889966912	0,1913308838	-3,6893687231	0,0002248112	0,0012880902
ROBO1	30,72425888	4,8642411211	0,8151345153	5,9674090964	0,0000000024	0,0000000705
ROGDI	105,2376686	0,7472462076	0,2313138363	3,2304402812	0,0012359846	0,0051363622
ROM1	29,23748827	-1,4481842915	0,4181749138	-3,4574390858	0,0005453355	0,0026478856
ROMO1	231,2579019	0,5131458936	0,1873988546	2,7384013557	0,0061738679	0,0191368146
ROR1	73,17429685	3,7408910856	0,6315974414	5,9229061168	0,0000000032	0,0000000902
RORA	1296,105204	-1,2268388672	0,3094060455	-3,9654850738	0,0000732530	0,0005082418
RORC	92,23482786	3,1110759074	0,5682326145	5,4750060482	0,0000000437	0,0000008802

SAMD12	138,1421711	2,2472614614	0,7505149187	2,9938929467	0,0027544167	0,0099626712
SAMD3	1386,422499	-0,8701417230	0,2546572627	-3,4169986936	0,0006271358	0,0029608024
SAMD9	718,9189907	-0,6171300972	0,2593709735	-2,3793336967	0,0173439671	0,0442978743
SAMSN1	717,6589836	-1,2159011438	0,4417586004	-2,7524108025	0,0059158260	0,0184615347
SAP30	46,5289698	0,9429455340	0,3579358088	2,6343884332	0,0084286517	0,0247436535
SAP30BP	665,7639085	-0,3256746697	0,1036584109	-3,1418065062	0,0016790896	0,0066335621
SAP30L	219,5017289	-0,6828110384	0,1328008286	-5,1416173032	0,0000002724	0,0000043133
SAPCD2	25,4837796	3,3138607162	0,6732078361	4,9224827849	0,0000008545	0,0000117003
SARAF	5225,075259	-0,5419172769	0,2243770902	-2,4152077045	0,0157262390	0,0410382274
SART1	672,2100521	-0,3464042363	0,1329415439	-2,6056891326	0,0091689637	0,0264894293
SART3	517,3528842	-0,7354479528	0,1352571698	-5,4374045688	0,0000000541	0,0000010608
SASH1	127,8170261	5,6319563487	0,9324498148	6,0399565309	0,0000000015	0,0000000474
SASH3	3325,285046	-1,0721949455	0,3807488982	-2,8160158948	0,0048623263	0,0157286497
SASS6	54,23781982	-0,5350294647	0,1977251628	-2,7058250181	0,0068114424	0,0207801483
SAT1	2836,237019	1,6195035476	0,3323077790	4,8735047745	0,0000010964	0,0000145676
SBDS	818,0471215	1,1217116084	0,3054906679	3,6718358227	0,0002408143	0,0013613640
SBF1	2125,481177	-0,6345951452	0,1865589006	-3,4015624110	0,0006700183	0,0031281464
SBF2	207,4031591	0,9386155955	0,1748781598	5,3672545321	0,0000000799	0,0000014945
SBK1	312,4371828	-0,8807686649	0,3090535619	-2,8498900300	0,0043734347	0,0144918822
SBNO1	714,4094033	-0,2914807753	0,0978517537	-2,9787987077	0,0028937987	0,0103595562
SBNO2	612,3269789	1,1859192242	0,3530245018	3,3593113737	0,0007813697	0,0035470021
SBSPON	26,17475435	1,7612829635	0,5721375039	3,0784259930	0,0020809721	0,0079168695
SCSD	217,6658839	1,0030888230	0,3489059786	2,8749545570	0,0040408565	0,0136213195
SCAMP1-AS1	102,3084619	-0,6406744478	0,2291403107	-2,7959918795	0,0051740700	0,0165344214
SCAMP2	1019,552108	-0,4561567558	0,1511877472	-3,0171542610	0,0025515993	0,0093607321
SCAMP3	554,8227885	-0,3710427064	0,0920888265	-4,0291389464	0,0000559815	0,0004088504
SCAPER	228,0337398	-0,5096736113	0,1846303333	-2,7605085375	0,0057711450	0,0180988978
SCARA3	705,1163013	10,1816762526	1,4942779522	6,8137766725	0,0000000000	0,0000000006
SCARB1	82,01644091	1,7162418037	0,3261749348	5,2617219185	0,0000001427	0,0000024627
SCARB2	805,3740492	1,7524023086	0,3567355382	4,9123288406	0,0000009000	0,0000122489
SCARF1	33,6756866	1,9203508002	0,5276579667	3,6394856448	0,0002729605	0,0015094692
SCARNA17	49,06937044	-1,3164561222	0,3722368009	-3,5356594324	0,0004067587	0,0020809473
SCAT1	30,35815699	-1,5864980700	0,4654287169	-3,4086768010	0,0006527877	0,0030554217
SCCPDH	322,910144	1,0020588017	0,2385815977	4,2000674469	0,0000266835	0,0002172780
SCD	275,9796852	2,7384950207	0,5316247680	5,1511802792	0,0000002589	0,0000041281
SCFD2	98,92189424	1,3613037947	0,4153370456	3,2799573179	0,0010382279	0,0044618607
SCIN	74,9949473	3,3742909416	0,6316346055	5,3421565451	0,0000000918	0,0000016935
SCLT1	139,705881	-0,5116589928	0,1696188372	-3,0164926532	0,0025571741	0,0093709676
SCLY	165,8457643	-0,6834875841	0,2515480921	-2,7171216581	0,0065852397	0,0201907349
SCMH1	117,5518923	0,8208207832	0,2226928662	3,6858871921	0,0002279073	0,0013026039
SCML1	69,31407606	1,0339544225	0,3194275875	3,2368970979	0,0012082672	0,0050417306
SCN9A	15,43741952	1,9517503659	0,4801864938	4,0645673940	0,0000481216	0,0003586474
SCNN1A	233,4252229	5,0202901851	1,0056482911	4,9920884243	0,0000005973	0,0000084986
SCRN2	471,5043091	-0,5470673026	0,2271342192	-2,4085639955	0,0160154182	0,0416474518
SCUBE1	28,08248535	2,8225446045	0,4939081088	5,7147160656	0,0000000110	0,0000002660
SCYL1	529,5409825	-0,3743491929	0,1315449468	-2,8457892304	0,0044301507	0,0146221701
SCYL3	217,3401195	-0,5359632894	0,1619747045	-3,3089320399	0,0009365257	0,0041100866
SDC3	75,25944492	3,7112018252	0,5528032314	6,7134229950	0,0000000000	0,0000000010
SDC4	369,7356956	4,3801182689	0,6726814880	6,5114297737	0,0000000001	0,0000000033
SDCBP2	50,68321554	2,5031571799	0,8242611237	3,0368497407	0,0023906459	0,0088571688
SDCBP2-AS1	40,87898616	-0,8294046464	0,3095514897	-2,6793753997	0,0073759649	0,0222103999
SDCCAG8	165,0573881	0,6844524368	0,2099212955	3,2605177536	0,0011120900	0,0047185929
SDF2	381,8701636	-0,7798954024	0,1040576803	-7,4813690236	0,0000000000	0,0000000000
SDHA	1750,550029	-0,5187788764	0,1510687616	-3,4340446752	0,0005946462	0,0028366256
SDHAF1	125,7912923	-1,1009899537	0,2150660923	-5,1193097985	0,0000003067	0,0000047884
SDHAF2	284,1682193	-0,7435539603	0,1259981284	-5,9013095650	0,0000000036	0,0000001011
SDHAF3	598,2950285	-0,5549411035	0,2238555659	-2,4789573800	0,0131811193	0,0355887686
SDR39U1	650,3349458	-0,6818527815	0,1427066437	-4,7780030672	0,0000017704	0,0000218626
SDS	51,74199484	2,7385685620	0,7433054856	3,6842109797	0,0002293221	0,0013084679
SDSL	32,76048521	2,6746383181	0,7731532567	3,4593895775	0,0005414012	0,0026317300
SEC14L1	572,7830125	0,6348234997	0,2315549934	2,7407031486	0,0061307869	0,0190358459
SEC14L2	61,94405137	1,7674113015	0,7517911681	2,3509328452	0,0187263629	0,0471060404
SEC23A	468,7325218	0,4413463408	0,1318867589	3,3464037224	0,0008186710	0,0036966217
SEC24A	233,6934503	0,5263694383	0,2209376551	2,3824321314	0,0171987333	0,0440284073
SEC24D	416,4648641	0,5400501860	0,2000379195	2,6997390657	0,0069393879	0,0211178297
SEC61G	248,7601951	0,8434996260	0,2502509639	3,3706149340	0,0007500061	0,0034313673
SEC63	568,8761825	0,5331150508	0,1478887216	3,6048147573	0,0003123757	0,0016852009
SECTM1	103,2237549	1,5682976228	0,4692066761	3,3424458825	0,0008304255	0,0037333538
SEH1L	220,0849694	0,5670950157	0,1454835488	3,8980009780	0,0000969900	0,0006393172
SEL1L	799,2002622	0,4376409569	0,1728952327	2,5312494168	0,0113658996	0,0314548945
SEL1L3	746,6521597	0,7547250559	0,1535408264	4,9154682407	0,0000008857	0,0000121031
SELENBP1	87,33820215	8,0293038584	1,0110215799	7,9416945205	0,0000000000	0,0000000000
SELENOF	1050,07472	0,3552594925	0,1324704513	2,6818017835	0,0073226834	0,0220744049
SELENOH	354,0882424	-0,4145277509	0,1349382366	-3,0719791081	0,0021264461	0,0080579854
SELENOI	151,0537814	1,2638466142	0,4788909618	2,6391114367	0,0083123652	0,0244728191
SELENON	164,3431387	1,0525009028	0,2843725810	3,7011335584	0,0002146385	0,0012376314
SELENOO	233,1615804	-0,5021081442	0,1528524881	-3,2849196662	0,0010201145	0,0043980594
SELENOP	408,5089374	7,6456153350	0,7692044326	9,9396402451	0,0000000000	0,0000000000
SELPLG	4817,025157	-1,3374549783	0,4029121610	-3,3194703656	0,0009018838	0,0039914467
SEM1	350,8875344	0,5343552298	0,1499491327	3,5635766619	0,0003658058	0,0019102578
SEMA3A	32,68929969	7,3265578324	1,0942351638	6,6955971395	0,0000000000	0,0000000011
SEMA3B	374,0901975	7,9376791258	1,0569843897	7,5097401659	0,0000000000	0,0000000000
SEMA3C	112,1177327	4,3808527234	0,7358648478	5,9669282157	0,0000000024	0,0000000706
SEMA4A	189,1966732	1,5365958699	0,5182057320	2,9652238004	0,0030246281	0,0107545409
SEMA4F	138,8782	-0,6265774811	0,2087559764	-3,0014828412	0,0026866818	0,0097578881
SEMA5A	29,7729678	4,4499723078	0,9591587179	4,6394525384	0,0000034933	0,0000392488
SEMA6A	20,09818337	1,2344260516	0,4743191908	2,6025218579	0,0092540916	0,0266773641
SEMA6B	20,04373588	1,6003627886	0,5028989449	3,1822750968	0,0014612295	0,0059116770
SEMA7A	44,07415556	1,7735586027	0,7207057514	2,4608636730	0,0138603021	0,0370525653
SENP1	194,8857603	-0,3882134268	0,1383030214	-2,8069699627	0,0050009905	0,0160998162
SENP2	232,511574	0,5595502882	0,2062415048	2,7130828434	0,0066660439	0,0203827685
SEPTIN10	87,76569635	4,8498888699	0,9987362990	4,8560049477	0,0000011977	0,0000157426
SEPTIN2	2307,834916	0,4434768563	0,1441063069	3,0774292964	0,0020879443	0,0079411503
SEPTIN4	20,37242963	-1,1928705145	0,3867694035	-3,0841904855	0,0020410687	0,0078003362
SEPTIN7	3164,684495	-0,7040324256	0,1652314078	-4,2608874126	0,0000203617	0,0001742328
SERF1A	135,3811378	-0,6120284419	0,2155465291	-2,8394725000	0,0045188190	0,0148658149
SERGEF	167,2024901	-0,5843149194	0,1959409972	-2,9820962829	0,0028628195	0,0102756134
SERHL2	13,45813544	1,8990093527	0,6840988827	2,7768052261	0,0054890065	0,0174268111
SERINC2	114,7474544	5,0865252546	1,0874535152	4,6774645383	0,0000029044	0,0000336725
SERINC3	2039,730027	-0,3359635547	0,1200355015	-2,7988682567	0,0051282056	0,0164229472
SERINC5	102,2399506	1,0337806984	0,4063856796	2,5438413561	0,0109640868	0,0305794879
SERP1	1376,108237	0,3700986351	0,1537561672	2,4109513250	0,0159109717	0,0414318365
SERP2	13,1117372	-1,1625647585	0,4852578965	-2,3957687969	0,0165856389	0,0427258755
SERPINA1	810,541887	4,4069983914	0,8768206607	5,0261114833	0,0000005005	0,0000073094
SERPINB8	225,4979413	0,8521197969	0,3321981149	2,5650952211	0,0103147494	0,0291000884
SERPINE1	465,7239099	3,9929863269	0,6773285721	5,8952158252	0,0000000037	0,0000001040
SERPINE2	144,0963961	1,6160738854	0,5036546685	3,2099692116	0,0013274919	0,0054608927
SERPINF1	47,49927646	2,2998136150	0,4851059727	4,7408479651	0,0000021283	0,0000256761
SERPING1	442,0104759	2,7607126262	0,4530396480	6,0937526888	0,0000000011	0,0000000355
SERPINH1	345,5741634	2,2779923433	0,3705942771	6,1468659652	0,0000000008	0,0000000265
SERPINI1	61,43084554	-1,0027982410	0,2214082894	-4,5291795122	0,0000059213	0,0000614481
SESN1	624,3190844	-0,9646158037	0,3285887894	-2,9356231835	0,0033287841	0,0116377752
SESN2	186,8407808	-0,7349972666	0,3096271337	-2,3738452280	0,0176516588	0,0449048057
SESTD1	164,7026458	1,4393438023	0,3767897847	3,8200181130	0,0001334419	0,0008315406
SETD2	1265,832872	-0,6143236825	0,1991543043	-3,0847020588	0,0020375615	0,0077898563
SETD4	174,6537682	-0,5480967364	0,1461407718	-3,7504915944	0,0001764882	0,0010511201
SETD7	109,1580173	2,1169362642	0,3964037719	5,3403534830	0,0000000928	0,0000017039
SFPQ	2278,949027	0,7500831220	0,1657614889	4,5250747154	0,0000060374	0,0000624145
SFXN1	296,5624148	0,6873390276	0,1707931041	4,0243956638	0,0000571218	0,0004153752
SFXN2	67,98038257	0,7352338954	0,2435035799	3,0193966583	0,0025327868	0,0092993210
SFXN4	88,32027534	1,1809927264	0,3241544972	3,6432019949	0,0002691627	0,0014933760
SGCB	81,40184003	1,7181602207	0,5481184347	3,1345456846	0,0017206179	0,0067824549
SGCE	61,58233435	2,8131062159	0,4648818344	6,0512285224	0,0000000014	0,0000000446
SGF29	195,1999757	-0,7708427808	0,1606672674	-4,7977587048	0,0000016045	0,0000200713
SGK1	732,8151807	2,6103810943	0,5535446843	4,7157549671	0,0000024082	0,0000285082
SGMS1	406,0347059	0,5914074998	0,1918152071	3,0832148755	0,0020477724	0,0078215142
SGMS2	82,76597474	5,8299197983	0,8603569261	6,7761641962	0,0000000000	0,0000000007
SGO1	23,0773618	1,2600623701	0,4401042598	2,8630990907	0,0041951855	0,0140108891
SGPL1	729,9798305	0,4301686227	0,1830287552	2,3502789066	0,0187593486	0,0471699668
SGPP2	67,75744633	3,3010993155	0,7141128977	4,6226574626	0,0000037886	0,0000420385
SGTB	115,185236	0,7974630535	0,2800282740	2,8477845975	0,0044024711	0,0145559472
SH2B2	19,74532306	1,6530575760	0,4554566901	3,6294506411	0,0002840250	0,0015584904
SH2D1A	630,223325	-1,2530847849	0,3527266126	-3,5525666062	0,0003814924	0,0019772384
SH2D1B	4610,167829	-1,9151049254	0,3903247002	-4,9064405216	0,0000009274	0,0000125967
SH2D2A	703,8018877	-1,5472898583	0,3512959746	-4,4045191807	0,0000106019	0,0001001412
SH2D3A	175,7448394	0,7457279500	0,2529245171	2,9484209703	0,0031940179	0,0112425422
SH2D3C	725,2531468	-1,4201187096	0,3382327142	-4,1986438626	0,0000268518	0,0002183840
SH2D4A	78,4808582	7,6839749583	1,3891437412	5,5314469853	0,0000000318	0,0000006722
SH3BP4	145,0536331	4,8350988422	0,8183546328	5,9083174315	0,0000000035	0,0000000977
SH3BP5	627,1756444	-0,7311640776	0,3047927382	-2,3988894287	0,0164449796	0,0424588332
SH3D19	37,82911594	5,4035138278	0,9255472244	5,8381827371	0,0000000053	0,0000001415
SH3PXD2B	39,83082884	5,5787154623	0,9286423768	6,0073884219	0,0000000019	0,0000000568
SH3RF1	92,48308639	4,1600231170	0,7077695660	5,8776518751	0,0000000042	0,0000001141
SH3RF3	11,48153782	3,1743979688	0,9535406438	3,3290641458	0,0008713833	0,0038785779
SH3TC1	260,8923819	0,9865505174	0,3291347900	2,9974057658	0,0027228803	0,0098680582
SH3YL1	168,0379013	1,0761894625	0,4062265959	2,6492343274	0,0080674374	0,0239483507
SHARPIN	525,463161	-0,4179849728	0,1073635842	-3,8931723187	0,0000989418	0,0006505896
SHB	61,74072008	3,8073430089	0,9007189040	4,2269999719	0,0000236828	0,0001971367
SHCBP1	24,736891	2,0733103830	0,5735071731	3,6151427224	0,0003001822	0,0016305296
SHF	14,73284657	-1,4257132835	0,4945264510	-2,8829286104	0,0039399672	0,0133602604
SHFL	980,3520887	-0,5074775079	0,1441841659	-3,5196479774	0,0004321199	0,0021882370
SHISA4	18,94714812	1,9216668843	0,4403422143	4,3640307970	0,0000127688	0,0001170760
SHISA5	2069,116512	-0,3808844024	0,1053225171	-3,6163625129	0,0002987719	0,0016241800
SHKBP1	1096,585111	-0,4837264093	0,1604926889	-3,0140125719	0,0025781712	0,0094299415
SHLD1	66,8300053	-0,8130319100	0,2108350874	-3,8562457508	0,0001151417	0,0007368899
SHLD2	247,9602507	0,8602626105	0,1884184696	4,5657021422	0,0000049783	0,0000533497
SHOX	11,40329251	-1,5996046893	0,5471274977	-2,9236415756	0,0034596286	0,0120203883
SHROOM1	36,45098117	1,7985003650	0,4495186781	4,0009469072	0,0000630895	0,0004497601
SHTN1	76,65783693	4,5363252425	0,6339279621	7,1559002182	0,0000000000	0,0000000001
SIAE	72,93573697	1,9172167075	0,2942163434	6,5163501305	0,0000000001	0,0000000032
SIDT1	439,4243019	-0,7995776559	0,2652647859	-3,0142623464	0,0025760494	0,0094273044
SIGLEC1	222,7935632	5,2063452847	0,8081723189	6,4421227541	0,0000000001	0,0000000049
SIGLEC14	77,6904399	1,8338320517	0,6529292626	2,8086228580	0,0049753894	0,0160404172
SIGLEC16	40,71670069	1,5238508804	0,5216039648	2,9214706620	0,0034838309	0,0120960125
SIGLEC5	29,24890764	4,5842924930	1,0281096771	4,4589527706	0,0000082361	0,0000813528
SIGLEC6	15,03041336	1,4625434960	0,6204440924	2,3572526743	0,0184107148	0,0464943785
SIGMAR1	576,8622035	-0,3848963877	0,1562756594	-2,4629324181	0,0137805888	0,0368866056
SIL1	242,3100286	0,9225652109	0,1809603549	5,0981620659	0,0000003430	0,0000052758
SIMC1	199,5661813	-0,8194330569	0,2241429839	-3,6558496946	0,0002563215	0,0014304405
SIN3A	564,9125785	-0,4573426999	0,1463157488	-3,1257243567	0,0017736772	0,0069521534
SINHCAF	693,7741347	1,4251488057	0,4516193001	3,1556419433	0,0016014532	0,0063811606
SIPA1L2	59,80094431	2,6942988278	0,5304885167	5,0789013202	0,0000003796	0,0000057868
SIRPA	142,1317505	2,5686375829	0,6493004160	3,9560079119	0,0000762126	0,0005238132
SIRPB1	119,096116	2,2752632415	0,7468417887	3,0465120310	0,0023151244	0,0086393946
SIRPG	61,48616704	2,1277958782	0,4783151717	4,4485228650	0,0000086463	0,0000845778
SIRT4	15,47482775	-2,5995778814	0,4528603303	-5,7403526158	0,0000000094	0,0000002316
SIRT7	653,1954582	-0,8551080847	0,0998500299	-8,5639041395	0,0000000000	0,0000000000
SIX4	25,17159846	2,3022278665	0,8689780571	2,6493509794	0,0080646530	0,0239466642
SKA1	30,85587013	1,3225898277	0,3202034271	4,1304803328	0,0000362006	0,0002818533
SKA2	129,7288699	0,6709446082	0,2627457097	2,5535892059	0,0106618963	0,0298725991
SKA3	25,67745187	3,2539860704	0,6577577417	5,0991206301	0,0000003412	0,0000052552
SKAP1	828,8158263	-0,5487332223	0,2334104983	-2,3509363387	0,0187262374	0,0471060404
SKI	714,7690754	1,2352498060	0,3363383793	3,6726619007	0,0002400370	0,0013575201
SKIC3	676,087072	0,4546625950	0,1112298819	4,0875809756	0,0000435870	0,0003297815
SLA	3715,106661	-1,7307917957	0,4495325404	-3,8491846242	0,0001185117	0,0007548299
SLAMF6	918,2388164	-1,1646170901	0,3523827041	-3,3049780155	0,0009498285	0,0041563154
SLAMF7	2299,619313	-1,0662550937	0,3164648835	-3,3692682802	0,0007536803	0,0034423268
SLC10A3	596,3668198	-0,6359608808	0,2157500278	-2,9478275729	0,0032001551	0,0112582559
SLC11A1	450,9694797	2,5428790467	0,6456194537	3,9386654685	0,0000819361	0,0005552976
SLC11A2	383,6138216	0,8746317078	0,1888486821	4,6313889932	0,0000036322	0,0000404714
SLC12A2	167,1357594	0,9567270803	0,2829648115	3,3810814681	0,0007220113	0,0033247586
SLC12A2-DT	74,05202806	0,6903288306	0,2652640324	2,6024592344	0,0092557818	0,0266772641
SLC12A7	377,1899464	0,8189204436	0,3514839185	2,3298964861	0,0198117285	0,0493825678
SLC12A8	58,47874993	2,9884779638	0,5124667705	5,8315546209	0,0000000055	0,0000001461
SLC15A2	32,56483148	2,0565009404	0,5645170931	3,6429380316	0,0002695426	0,0014948723
SLC15A3	50,54027876	1,8625090152	0,5393963083	3,4529509869	0,0005544897	0,0026863416
SLC15A4	823,2050597	-1,6252375346	0,1917682834	-8,4750089486	0,0000000000	0,0000000000
SLC16A1	229,8276071	1,9370601026	0,4360705213	4,4420799118	0,0000089093	0,0000867099
SLC16A10	42,6873714	2,2286678970	0,6705029002	3,3238751037	0,0008877594	0,0039410429
SLC16A14	8,77818006	2,5081093965	0,6933409233	3,6174258755	0,0002975475	0,0016181775
SLC16A3	444,6503675	2,2180227301	0,3955518807	5,6059956705	0,0000000207	0,0000004622
SLC16A4	31,63749856	1,1669826739	0,4233344137	2,7559362908	0,0058524395	0,0183108799
SLC16A5	98,42568049	1,5405720726	0,4244883455	3,6292361373	0,0002842611	0,0015591503
SLC16A6	101,5686135	1,7299522540	0,5879581777	2,9416346249	0,0032649544	0,0114354113
SLC17A9	158,9475223	1,0486634866	0,4112165325	2,5501491384	0,0107676846	0,0301441419
SLC18B1	86,11947919	1,1270850195	0,2995245645	3,7629134742	0,0001679453	0,0010064755
SLC19A1	90,68447982	1,9656278868	0,3415546482	5,7549440383	0,0000000087	0,0000002149
SLC19A2	55,37769534	0,8665652272	0,3507667393	2,4704885888	0,0134928623	0,0362720046
SLC1A3	52,43569236	1,7583126729	0,6238902031	2,8183046699	0,0048277976	0,0156470395
SLC1A5	407,5651477	0,7196911532	0,2351582861	3,0604541522	0,0022100159	0,0083231154
SLC1A7	86,09358792	-2,8527480144	0,5395649629	-5,2871224261	0,0000001243	0,0000021834
SLC20A1	1206,018582	0,4537190830	0,1617340545	2,8053404369	0,0050263461	0,0161658781
SLC22A20P	28,97328908	2,4471483290	0,5324473643	4,5960380373	0,0000043060	0,0000470162
SLC22A23	32,68238042	1,7116094331	0,5283201113	3,2397203822	0,0011964697	0,0050013918
SLC22A5	81,60201565	1,4398004821	0,3267044204	4,4070431622	0,0000104791	0,0000992605
SLC25A11	545,6659145	-0,4969727032	0,1629973076	-3,0489626519	0,0022963303	0,0085923395
SLC25A13	107,506668	1,7428974578	0,3635221301	4,7944741559	0,0000016310	0,0000203441
SLC25A14	77,20920742	0,4899473848	0,1799900325	2,7220806509	0,0064872308	0,0199538681
SLC25A20	546,6357717	-0,9491843773	0,2560787307	-3,7066115357	0,0002100508	0,0012186565
SLC25A24	287,0559197	1,5197247206	0,3335750733	4,5558701540	0,0000052169	0,0000554220
SLC25A3	2448,922229	0,5033483887	0,1310255851	3,8416038229	0,0001222330	0,0007742383
SLC25A32	247,0922154	0,3598507517	0,1256811840	2,8632030690	0,0041938169	0,0140106168
SLC25A33	89,94799217	0,6789528214	0,2182342965	3,1111192524	0,0018637965	0,0072463032
SLC25A36	552,7878835	0,6258117316	0,2172138024	2,8810863988	0,0039630699	0,0134196027
SLC25A37	222,5805322	1,2073723167	0,2994775184	4,0315991772	0,0000553986	0,0004054732
SLC25A39	1130,458967	0,4214791676	0,1628036366	2,5888744037	0,0096290207	0,0275270946
SLC25A42	327,1006364	-1,1414155863	0,2115549955	-5,3953610678	0,0000000684	0,0000013001
SLC25A5	1264,589556	0,7906070295	0,1725437498	4,5820671023	0,0000046040	0,0000498553
SLC25A53	107,0481095	-0,6983074195	0,2790652139	-2,5023090831	0,0123386153	0,0336666860
SLC26A6	355,6104754	-0,5768590587	0,2015877951	-2,8615773016	0,0042153869	0,0140582582
SLC27A2	30,89501891	1,6634538898	0,5904982239	2,8170340583	0,0048469386	0,0156901959
SLC27A3	639,4484257	-0,5094506619	0,1585727712	-3,2064184668	0,0013439839	0,0055168328
SLC29A1	89,48319335	1,4423518581	0,3863176563	3,7335903098	0,0001887595	0,0011109912
SLC2A10	40,77339433	2,7172676406	0,5489297429	4,9501191655	0,0000007417	0,0000103024
SLC2A3	1910,709437	1,4679126960	0,4152309085	3,5351720350	0,0004075098	0,0020822197
SLC2A5	273,6335877	5,1495704240	0,7558852190	6,8126345284	0,0000000000	0,0000000006
SLC2A9	25,21391294	2,6643635697	0,6702861764	3,9749642219	0,0000703898	0,0004911598
SLC30A1	391,8667235	0,9248185660	0,2350911529	3,9338722644	0,0000835882	0,0005640677
SLC30A9	443,6361196	0,8845485216	0,1699889752	5,2036253412	0,0000001954	0,0000032396
SLC31A1	338,1421422	1,2939647805	0,2809252040	4,6060829068	0,0000041032	0,0000450855
SLC34A2	474,6981304	9,6087788123	1,3155611682	7,3039392955	0,0000000000	0,0000000000
SLC35A2	250,7972476	0,7517070670	0,2655648738	2,8295312672	0,0046616245	0,0152405794
SLC35A3	271,8281366	0,8974017208	0,3437977607	2,6102605179	0,0090473294	0,0262111609
SLC35A4	906,4861227	-0,4272752768	0,1648370566	-2,5921089306	0,0095390117	0,0273064862
SLC35A5	195,042598	0,7812818794	0,2708662390	2,8843815823	0,0039218322	0,0133235391
SLC35B2	415,7229674	-0,4204449254	0,1555857189	-2,7023362325	0,0068854094	0,0209772773
SLC35E1	629,751494	0,5265233419	0,1686012644	3,1228808270	0,0017908416	0,0070058521
SLC35F5	377,015342	0,9436633812	0,2855132498	3,3051474213	0,0009492646	0,0041551548
SLC37A2	288,6846461	2,1472460082	0,5727073752	3,7492899540	0,0001773260	0,0010552352
SLC38A1	771,887789	0,6782247499	0,1843047309	3,6799096071	0,0002332166	0,0013262002
SLC39A11	166,6193512	1,1651304866	0,3552839177	3,2794349211	0,0010401520	0,0044687012
SLC39A13	560,4421279	-0,4279204371	0,1520331104	-2,8146529140	0,0048829941	0,0157803361
SLC39A14	186,6060632	1,3722137462	0,3201880773	4,2856490333	0,0000182206	0,0001585325
SLC39A4	156,2241838	1,2934226387	0,4463841194	2,8975528838	0,0037608513	0,0129076973
SLC39A7	798,5493101	0,8059469031	0,3285990204	2,4523715930	0,0141918021	0,0377961331
SLC39A8	815,0301489	2,2674477501	0,4760406128	4,7631412981	0,0000019060	0,0000233228
SLC40A1	452,9723009	4,5675491269	0,8786003893	5,1986650385	0,0000002007	0,0000033191
SLC41A2	42,26073247	2,4473598958	0,5290538307	4,6259184861	0,0000037294	0,0000414517
SLC43A1	294,683101	-1,3987682024	0,3637021053	-3,8458172550	0,0001201023	0,0007628961
SLC43A2	256,6967613	1,1745940363	0,3761836447	3,1223852791	0,0017938591	0,0070149168
SLC43A3	525,9160281	1,1983601155	0,4155629111	2,8837032454	0,0039302893	0,0133403846
SLC44A1	393,1787404	1,0118515914	0,2350533471	4,3047742300	0,0000167156	0,0001471928
SLC44A3-AS1	12,74385338	1,4652908227	0,4689536644	3,1246097069	0,0017804112	0,0069765166
SLC46A1	118,5879138	2,3407342676	0,4927235180	4,7506039025	0,0000020281	0,0000246374
SLC48A1	160,3504279	1,2688141947	0,3855968772	3,2905198917	0,0010000243	0,0043294716
SLC49A4	96,92345387	1,1144295827	0,2824887922	3,9450399925	0,0000797867	0,0005429365
SLC4A10	41,04510508	2,7990186134	0,7276579728	3,8460622856	0,0001200308	0,0007628961
SLC4A3	19,23628677	3,8848092131	0,9172057311	4,2354829251	0,0000228061	0,0001917365
SLC4A4	127,3272318	1,1253494892	0,3366452261	3,3428321630	0,0008292804	0,0037294067
SLC5A3	566,1511003	-0,9773286289	0,3075970961	-3,1773337304	0,0014863589	0,0059935263
SLC66A2	145,903282	0,5371506938	0,1822257152	2,9477216940	0,0032012512	0,0112590684
SLC6A16	32,50147819	-1,0268047288	0,3111004378	-3,3005570035	0,0009649311	0,0042073121
SLC6A8	34,47957034	2,4045947454	0,6723862792	3,5762097802	0,0003486119	0,0018401242
SLC7A1	174,956654	1,4273526365	0,4513575134	3,1623553263	0,0015649848	0,0062562207
SLC7A14	9,216765262	-1,2870421962	0,4517739642	-2,8488631443	0,0043875749	0,0145173940
SLC7A6OS	336,2180699	-0,6998892575	0,1724188701	-4,0592381637	0,0000492331	0,0003657159
SLC7A7	272,504882	1,9441275825	0,6353961952	3,0597091974	0,0022155200	0,0083368462
SLC7A8	115,4506317	4,7010090353	0,7409267033	6,3447693467	0,0000000002	0,0000000086
SLC8A1	99,87395818	3,2452926188	0,6364112206	5,0993642377	0,0000003408	0,0000052544
SLC9A3R1	2879,921106	-1,0919262567	0,2462852920	-4,4335810886	0,0000092681	0,0000895612
SLC9A3R2	85,10914182	2,9298979608	0,9191606424	3,1875798697	0,0014346883	0,0058235538
SLC9A7	65,93131541	1,3819928738	0,2791512968	4,9506948019	0,0000007395	0,0000102826
SLCO2B1	427,8583209	5,2151200341	0,7751277146	6,7280784002	0,0000000000	0,0000000009
SLCO3A1	467,3031706	0,9005381528	0,3534576626	2,5477963786	0,0108405725	0,0302977796
SLCO4A1	67,7680717	1,4576792362	0,5242978389	2,7806298789	0,0054253553	0,0172473505
SLCO4C1	209,6139891	-1,1801695630	0,2669869340	-4,4203286930	0,0000098551	0,0000942782
SLFN12L	1588,630643	-0,4444691999	0,1911339137	-2,3254334685	0,0200487936	0,0498438503
SLFN13	1059,167401	-0,4650555822	0,1541692345	-3,0165265044	0,0025568886	0,0093709676
SLFN5	2155,236089	-0,7740576290	0,2538044904	-3,0498184955	0,0022897970	0,0085723135
SLIT3	89,00684359	5,2882741742	0,6721594200	7,8675891711	0,0000000000	0,0000000000
SLITRK5	41,68431301	2,0893561951	0,6498505920	3,1991254561	0,0013784517	0,0056343637
SLPI	2327,345254	11,0284094650	1,1465185768	9,6190412337	0,0000000000	0,0000000000
SLX1A	254,6207919	-0,6601511989	0,1955582018	-3,3757274967	0,0007362080	0,0033797270
SLX1A-SULT1A3	303,0336111	-0,6917524611	0,2392639547	-2,8911687179	0,0038381201	0,0131086263
SLX1B	176,2704832	-0,5852721252	0,1818772289	-3,2179516309	0,0012910959	0,0053323567
SLX1B-SULT1A4	153,4452305	-1,5227149767	0,4151286457	-3,6680574227	0,0002444003	0,0013792990
SLX4	156,8942778	-0,5876247777	0,1615412607	-3,6376119104	0,0002751776	0,0015198568
SMAD1	72,54640477	1,7375920735	0,6198301427	2,8033358718	0,0050576968	0,0162551572
SMAD3	602,835306	-0,9354832688	0,3386962475	-2,7620127937	0,0057446227	0,0180273723
SMARCA1	64,17823179	6,6568806450	0,9767387587	6,8154156735	0,0000000000	0,0000000006
SMARCAL1	300,1686473	-0,5923904727	0,1971923559	-3,0041249217	0,0026634593	0,0096970956
SMARCC2	1301,582188	-0,4704211645	0,1434497345	-3,2793449642	0,0010404836	0,0044687012
SMARCD2	890,2795377	-0,5508176128	0,1376490739	-4,0016078372	0,0000629135	0,0004487630
SMARCD3	189,4698589	-1,3341240833	0,5044295017	-2,6448177176	0,0081734934	0,0241526282
SMCO4	109,0613365	1,2012839141	0,4685351158	2,5639143654	0,0103499077	0,0291870433
SMG6	297,7625551	-0,5984426144	0,2146776529	-2,7876334878	0,0053094575	0,0169389187
SMG8	194,182355	-0,9959432585	0,2486270793	-4,0057718619	0,0000618153	0,0004428067
SMIM10L2A	12,03699117	1,7657105495	0,6486400832	2,7221730435	0,0064854173	0,0199538681
SMIM11	110,8958715	-0,6319824238	0,2278146701	-2,7741101060	0,0055352940	0,0175593656
SMIM15	329,5734495	0,5119829615	0,2198259110	2,3290382797	0,0198570379	0,0494680233
SMIM24	15,97186789	2,3689456847	0,5620532357	4,2148065952	0,0000249992	0,0002057929
SMIM27	72,16617818	-0,6750787457	0,1651056371	-4,0887686056	0,0000433669	0,0003286704
SMIM29	276,0762234	-0,7938712918	0,1467581280	-5,4093855141	0,0000000632	0,0000012109
SMIM30	90,91335339	0,6901968261	0,2211536575	3,1209008822	0,0018028646	0,0070399838
SMIM35	56,96976886	1,9025554813	0,5322203743	3,5747513122	0,0003505609	0,0018473742
SMIM4	34,57073344	0,7195640591	0,2804995243	2,5652851136	0,0103088084	0,0290894258
SMIM5	42,97384605	4,2598850734	1,0810703026	4,0329339015	0,0000550848	0,0004036152
SMKR1	34,55554416	-1,0601277407	0,3336799196	-3,1770798254	0,0014876608	0,0059948835
SMOC2	65,23183554	6,9999470038	1,0388787457	6,7379826881	0,0000000000	0,0000000009
SMPD3	79,68986082	1,7682129861	0,4791841166	3,6900490625	0,0002242106	0,0012852960
SMPD4BP	357,7740354	-0,5333919970	0,1623282303	-3,2858856170	0,0010166228	0,0043858152
SMPDL3A	124,176622	2,5047468840	0,6860297300	3,6510762936	0,0002611436	0,0014520771
SMPDL3B	36,01268298	2,6131538534	0,7597989504	3,4393246054	0,0005831675	0,0027963787
SMS	559,3151958	1,3722714648	0,2969785692	4,6207760664	0,0000038231	0,0000423876
SMTN	38,73869118	0,9007321653	0,3671848024	2,4530758337	0,0141640475	0,0377296813
SMYD2	348,8037114	0,4934338011	0,0981752745	5,0260496226	0,0000005007	0,0000073094
SMYD4	230,4424305	-1,0676620425	0,2091008749	-5,1058664037	0,0000003291	0,0000050976
SMYD5	136,6886821	0,9110810135	0,2688462856	3,3888524949	0,0007018550	0,0032508493
SNAI3	110,4266843	-1,7815159782	0,4346162212	-4,0990554229	0,0000414840	0,0003177988
SNAI3-AS1	45,83175813	-1,0214702933	0,3524076715	-2,8985472671	0,0037489580	0,0128758822
SNAP25	18,82138793	1,4169894579	0,5331582575	2,6577276784	0,0078669432	0,0234371527
SNAP47	272,3476831	-0,4028046849	0,1260574432	-3,1954057996	0,0013963436	0,0056850528
SNAPC4	137,444941	-0,5472325534	0,1934314094	-2,8290780460	0,0046682314	0,0152473657
SNAPC5	204,2684059	-0,5659182869	0,2128174770	-2,6591772201	0,0078331748	0,0233568131
SNAPIN	373,100154	-0,4862585442	0,1870144168	-2,6001179609	0,0093191720	0,0268178249
SND1	988,3848658	0,4140584568	0,1500711774	2,7590804841	0,0057964257	0,0181610545
SNHG1	714,789763	0,7220651174	0,2670081431	2,7042711308	0,0068454408	0,0208744073
SNHG12	236,0709465	0,7848777903	0,3212732139	2,4430220052	0,0145649492	0,0385758288
SNHG16	257,2856696	1,1504272042	0,3155303287	3,6460114904	0,0002663421	0,0014801689
SNHG29	700,2584735	0,8266597706	0,2188060285	3,7780484217	0,0001580622	0,0009578093
SNHG3	314,033549	1,0231711805	0,3210972238	3,1864840445	0,0014401343	0,0058421362
SNHG5	499,9093164	0,7864040114	0,2517721673	3,1234747666	0,0017872919	0,0069932991
SNHG8	223,7662339	1,1676148558	0,2921011515	3,9972966545	0,0000640700	0,0004553904
SNN	200,7080477	2,4168285009	0,6017243849	4,0165061889	0,0000590678	0,0004265305
SNPH	56,00335305	-1,0824774993	0,3651685210	-2,9643231467	0,0030334955	0,0107803937
SNRNP35	197,8432899	-0,5762382304	0,1680530981	-3,4346753157	0,0005932642	0,0028357017
SNRNP40	498,8255362	-0,8591443854	0,2248932617	-3,8202318250	0,0001333263	0,0008312052
SNRPD1	290,1577857	0,5563433455	0,2112528804	2,6330749249	0,0084615679	0,0248210762
SNRPD2	823,4635267	0,4041100602	0,1220906612	3,3098626407	0,0009334143	0,0040993050
SNRPE	340,6778216	0,5123125126	0,1825639718	2,8062389313	0,0050123511	0,0161286745
SNRPF	175,5868537	0,6232067582	0,2227277186	2,7980655576	0,0051409677	0,0164520745
SNRPN	895,3752692	-0,5792048563	0,1753200778	-3,3036985165	0,0009541805	0,0041712468
SNTB1	320,7172994	1,1816757923	0,3732341256	3,1660463437	0,0015452723	0,0061902874
SNTB2	383,2622119	-0,3212565538	0,1375328975	-2,3358524372	0,0194989336	0,0487382815
SNW1	635,4675301	-0,2215589865	0,0880782870	-2,5154493626	0,0118880753	0,0326758207
SNX13	313,3442312	0,8351657688	0,2076719644	4,0215624304	0,0000578134	0,0004188504
SNX14	781,1528158	0,2982877389	0,1110666661	2,6856628352	0,0072385856	0,0218652070
SNX15	160,8749135	-0,4840580392	0,1395298165	-3,4692385988	0,0005219940	0,0025549233
SNX2	845,9292667	1,0192275254	0,2023878622	5,0360111734	0,0000004753	0,0000070385
SNX20	1290,234676	-1,6945676103	0,4016557817	-4,2189548555	0,0000245437	0,0002025498
SNX24	74,29401726	1,8276168548	0,4105276184	4,4518733276	0,0000085124	0,0000834504
SNX3	1203,528423	0,6826250137	0,1464112004	4,6623824673	0,0000031257	0,0000358887

[illegible]	[illegible]	[illegible]	[illegible]	[illegible]	[illegible]	[illegible]
SPOP	540,3175158	-0,7041788401	0,1694217456	-4,1810449608	0,0000290173	0,0002340987
SPOPL	176,4127285	0,9611904753	0,2956505173	3,2511036478	0,0011495794	0,0048414702
SPOUT1	451,923278	-0,8049716993	0,2070375305	-3,8880462750	0,0001010544	0,0006518938
SPP1	39879,28823	8,1069710685	0,9004067842	9,0035450671	0,0000000000	0,0000000000
SPR	90,7977293	3,4276237298	0,6104734454	5,6146675037	0,0000000197	0,0000004439
SPRED1	103,9467391	2,5969400535	0,5177772047	5,0155550107	0,0000005288	0,0000076711
SPRED2	71,0102456	3,2436888604	0,5766505215	5,6250514646	0,0000000185	0,0000004224
SPRY1	123,6953832	2,4403827860	0,4840762146	5,0423522415	0,0000004598	0,0000068282
SPRYD3	527,8721449	-0,8304885903	0,1780137093	-4,6653053495	0,0000030816	0,0000354515
SPSB1	109,1431072	1,4288084368	0,4102246194	3,4829621603	0,0004959429	0,0024491909
SPSB3	1160,936341	-1,3409009363	0,1840799122	-7,2843414606	0,0000000000	0,0000000000
SPTBN1	1093,292538	0,6590108282	0,2573029624	2,5612255765	0,0104303608	0,0293585646
SPTBN2	99,72370928	3,7645213097	0,9495011506	3,9647358115	0,0000734772	0,0005090149
SPTBN5	95,56750978	-1,0636849606	0,4369210359	-2,4345015993	0,0149122140	0,0393413849
SPTLC1	547,2366829	0,5384917833	0,1819731210	2,9591820936	0,0030845578	0,0109402233
SPTSSA	186,3768392	0,7083858873	0,1877263892	3,7735024375	0,0001609716	0,0009702993
SQLE	376,6221043	1,0860196920	0,4510039154	2,4080050185	0,0160399605	0,0416021432
SQSTM1	4108,174372	-0,6298926878	0,2400273846	-2,6234200943	0,0087051865	0,0254003490
SRC	104,9884373	1,5809235658	0,4977530053	3,1761205837	0,0014925889	0,0060079491
SRD5A3	201,2441968	1,9376476584	0,4534856085	4,2727875436	0,0000193044	0,0001665626
SREBF1	293,5122289	1,7388605747	0,2894450101	6,0075679794	0,0000000019	0,0000000568
SRF	321,6167744	-0,5016974068	0,1885976532	-2,6601465047	0,0078106645	0,0232183761
SRFBP1	68,17844277	0,6109653180	0,2153384296	2,8372330892	0,0045506377	0,0149321267
SRGAP1	73,34752958	2,4567164970	0,5070681217	4,8449341361	0,0000012665	0,0000164870
SRGAP2B	113,2385327	0,6916100776	0,2718856087	2,5437528007	0,0109669321	0,0305798612
SRGAP2C	151,4353473	0,8876368254	0,1734803932	5,1166406722	0,0000003110	0,0000048398
SRP14-DT	28,2258822	-0,9470252056	0,3948363005	-2,3985261851	0,0164611995	0,0424997910
SRP72	923,8207547	0,4362231330	0,1742255999	2,5038406143	0,0122853264	0,0335553222
SRPK2	461,6886137	-0,6190222870	0,1553777170	-3,9839836573	0,0000677595	0,0004765920
SRPRB	290,9583768	0,5825774685	0,1986650112	2,9324613620	0,0033628676	0,0117477806
SRRT	840,4998004	0,4343822225	0,1696528364	2,5604182739	0,0104540250	0,0294074830
SRSF11	1890,099827	-0,2963508269	0,1153103186	-2,5700286891	0,0101690093	0,0287673109
SRSF3	2687,830437	0,7428327845	0,1647025191	4,5101482887	0,0000064782	0,0000663095
SRSF4	863,3669833	-0,4186053770	0,1665564326	-2,5132945657	0,0119609417	0,0328693908
SRSF6	1205,273833	0,6571627388	0,2608873296	3,2855666384	0,0010177746	0,0043893773
SRSF7	1967,469381	0,4896792574	0,1716052464	2,8535214848	0,0043237604	0,0143468935
SRSF8	591,2605899	-0,4494544165	0,1148601600	-3,9130575532	0,0000911348	0,0006069641
SRXN1	457,6012569	-0,7303391041	0,2124909130	-3,4370368770	0,0005881155	0,0028140899
SS18L2	184,2650244	-0,4814899441	0,1332167853	-3,6143339075	0,0003011208	0,0016349680
SSB	507,7298085	0,5233978955	0,1757082131	2,9787886883	0,0028939028	0,0103595562
SSBP1	571,5697781	0,4305923886	0,1319496037	3,2632170284	0,0011011626	0,0046826589
SSBP2	242,5219445	0,5166203414	0,1939374495	2,6638503427	0,0077251923	0,0231171827
SSBP3-AS1	21,81514154	-1,3632471808	0,4860655383	-2,8046571362	0,0050370129	0,0161964150
SSH1	844,8856345	-0,8182188218	0,2191039061	-3,7343871964	0,0001881728	0,0011384490
SSNA1	358,6942282	-0,4849062790	0,1749594087	-2,7715358819	0,0055792523	0,0176490566
SSR1	1246,374379	0,4122920806	0,1740210719	2,3692077984	0,0178262348	0,0452633692
SSR3	620,9623248	0,8451670785	0,1865725537	4,5299646803	0,0000058994	0,0000613224
SSR4	876,1790931	0,5034387273	0,1977218090	2,5461672556	0,0108903630	0,0304179884
ST13	956,6893790	0,7270116798	0,2182960190	3,3289697366	0,0008719949	0,0038800170
ST14	827,7645878	5,5273823925	0,7280001736	7,5925564213	0,0000000000	0,0000000000
ST3GAL2	397,3393859	-0,7695627645	0,2973689882	-2,5879051587	0,0096561563	0,0275773104
ST3GAL6	44,39348393	3,6004527514	0,7421176031	4,8450658904	0,0000012657	0,0000164870
ST6GALNAC2	46,02367204	2,2836334878	0,6693722214	3,4116048067	0,0006458167	0,0030332623
ST6GALNAC6	2103,656494	-1,4039066354	0,2623479258	-5,3513159322	0,0000000873	0,0000016211
ST8SIA6	156,1776807	-1,8453098015	0,2926577070	-6,3096637807	0,0000000003	0,0000000105
STAB1	798,868144	5,2048009083	0,9392212042	5,5416135038	0,0000000300	0,0000006363
STAC3	14,58199022	2,4901892652	0,7464639037	3,3359807436	0,0008499905	0,0038060121
STAG3	202,5087213	-1,2019023003	0,2376150638	-5,0581779039	0,0000004233	0,0000063665
STAG3L5P-PVRIG2P-	846,110731	-0,5755610934	0,2268632089	-2,5370395727	0,0111794316	0,0310666282
STAP2	54,45478987	1,4633563766	0,5352428432	2,7345254570	0,0062472162	0,0193260240
STARD13	17,05656742	4,9813083330	0,8159821087	6,1046783749	0,0000000010	0,0000000335
STARD5	346,8513139	-1,0849173243	0,2777503061	-3,9060886715	0,0000938021	0,0006231867
STARD8	19,84881549	1,8020386100	0,5867496650	3,0712227335	0,0021318406	0,0080738813
STAT4	1543,775251	-1,0494666433	0,3224875660	-3,2542856033	0,0011367793	0,0047920606
STAT4-AS1	14,34401628	-1,5185307328	0,4500364193	-3,3742396562	0,0007401990	0,0033934189
STAT5B	1281,235899	-0,5301702511	0,1875306502	-2,8271125303	0,0046969819	0,0153226797
STAT6	2217,426532	-0,4334410762	0,1319585033	-3,2846771174	0,0010209930	0,0044004375
STBD1	41,36669005	2,6348872281	0,7315264507	3,6019028777	0,0003158964	0,0017001061
STEAP2	19,06908156	2,3602282133	0,7238955831	3,2604540433	0,0011123399	0,0047185929
STEAP3	101,72073	2,8989871084	0,6326257100	4,5824680590	0,0000045952	0,0000497998
STEAP4	52,06749185	4,0823836243	0,7173348215	5,6910434315	0,0000000126	0,0000003008
STIL	44,67668871	1,0931412573	0,3529472144	3,0971805775	0,0019537082	0,0075263059
STIM2	620,9418349	-0,5183561439	0,1809474164	-2,8646783370	0,0041743303	0,0139732618
STIMATE-MUSTN1	100,4428578	-0,7113268874	0,2121726589	-3,3368584769	0,0008472108	0,0037988057
STK11	717,8768675	-0,4199258655	0,1487629280	-2,8227856971	0,0047608383	0,0154896867
STK11IP	393,5846454	-0,6839210560	0,1908981790	-3,5826484029	0,0003401283	0,0018077212
STK24	790,8011242	0,5699158240	0,2070655961	2,7523448357	0,0059170179	0,0184615347
STK25	1012,571020	-0,3729155946	0,1045681611	-3,5662441684	0,0003621340	0,0018966239
STK26	517,2928094	-0,9541119802	0,1640707043	-5,8152488842	0,0000000061	0,0000001589
STK3	126,1544673	1,3293320950	0,3481838707	3,8179025703	0,0001345811	0,0008378375
STK32C	117,1168525	-0,8282814554	0,2560181740	-3,2352447581	0,0012153850	0,0050662997
STK38	1324,529861	-0,8340118206	0,2251935863	-3,7035327447	0,0002126177	0,0012284205
STK38L	216,8638087	1,1129043653	0,1915827147	5,8069140885	0,0000000064	0,0000001660
STK4	2206,448224	-0,7964386113	0,1743509478	-4,5680189701	0,0000049235	0,0000528475
STK40	868,1108349	-0,6715623037	0,2674547146	-2,5109383646	0,0120410718	0,0330221734
STMN1	698,8274949	0,9742307928	0,2760742925	3,5288718263	0,0004173252	0,0021261665
STON1	25,04830742	1,6403904081	0,5451261470	3,0091941419	0,0026194167	0,0095574273
STRAP	614,6508387	0,4028119718	0,1000028806	4,0280036857	0,0000562525	0,0004103838
STRIP1	530,3792528	-0,7969232281	0,1668619657	-4,7760028466	0,0000017881	0,0000220003
STRN3	99,01257665	0,7131126468	0,2768426325	2,5758772789	0,0099986127	0,0283926849
STS	122,8025427	1,2039947845	0,3304519186	3,6434794796	0,0002689771	0,0014929616
STT3B	1626,877419	0,4083860093	0,1375561528	2,9688676293	0,0029889932	0,0106489807
STUB1	821,1345711	-0,4206232590	0,1279069469	-3,2885098827	0,0010071925	0,0043507095
STX10	421,9523843	-0,3643612655	0,1099409968	-3,3141558434	0,0009192026	0,0040525604
STX16-NPEPL1	150,5585826	-0,7046772543	0,2630466781	-2,6789057340	0,0073863185	0,0222316294
STX3	193,1897763	0,7723127668	0,3202088282	2,4119094167	0,0158694848	0,0413420424
STX5	255,1671421	-0,3430811106	0,1295047252	-2,6491781163	0,0080687794	0,0239482507
STXBP1	84,64140457	1,9194120774	0,4281970071	4,4825443561	0,0000073758	0,0000743629
STYK1	88,53607899	2,3843035574	0,4990429380	4,7777523250	0,0000017727	0,0000218698
SUCLA2	343,1282395	0,7172225945	0,2994254447	2,3953294794	0,0166054361	0,0427547168
SUCLG2	346,8268682	0,8385865215	0,1687052239	4,9707205384	0,0000006670	0,0000093721
SUCO	319,1623439	0,4780488672	0,1910932746	2,5017828820	0,0123569676	0,0337030971
SUFU	133,3133294	-0,4871022876	0,1287280090	-3,7839658303	0,0001543490	0,0009404879
SUGP1	288,1978893	-0,3526726269	0,1182074784	-2,9835077408	0,0028496483	0,0102419935
SULF1	71,76202972	6,6846658030	0,8638261398	7,7384388986	0,0000000000	0,0000000000
SULF2	302,2083053	4,1191215800	0,7583889099	5,4314106209	0,0000000559	0,0000010922
SULT1A1	162,6793267	1,5349625682	0,3548378495	4,3256731777	0,0000152067	0,0001362729
SUMO3	457,4373533	0,7067565951	0,2395856472	2,9499145433	0,0031786184	0,0112058201
SUN1	829,1483094	0,8387146057	0,3499470702	2,3966898811	0,0165436587	0,0426447364
SUN2	9373,8159	-0,9884234216	0,2878425477	-3,4339378576	0,0005948806	0,0028366256
SUPT3H	61,98951346	0,6828412159	0,1786506794	3,8222144917	0,0001322586	0,0008260790
SUPT6H	884,5270353	-0,2053739694	0,0865496691	-2,3729029985	0,0176488882	0,0449048057
SUSD1	296,252242	1,6696667364	0,6399668202	2,6090300330	0,0090799201	0,0262886051
SUSD2	42,5343745	3,6479501016	0,9357736169	3,8983254449	0,0000968602	0,0006390873
SUV39H1	165,2270776	-0,9157487480	0,2291559537	-3,9961857115	0,0000643712	0,0004565000
SVIL	218,122222	1,9518726999	0,4967070688	3,9296253714	0,0000850783	0,0005722909
SWAP70	269,9421582	0,7653567930	0,2438280096	3,1389207258	0,0016957132	0,0066953063
SWSAP1	59,20730608	-0,6915185190	0,2247212259	-3,0772184129	0,0020894144	0,0079444967
SYAP1	377,1508405	0,5432784940	0,1652687434	3,2872229544	0,0010118069	0,0043692400
SYDE2	21,20443475	2,3993365574	0,7052687976	3,4024847828	0,0006677363	0,0031185741
SYNC	91,95381539	2,8088615016	0,9702872005	2,8948763823	0,0037930797	0,0129943598
SYNCRIP	904,4110561	0,6750516224	0,2063325011	3,2716688778	0,0010691471	0,0045655839
SYNE1	4019,453827	-0,6693490522	0,2570706076	-2,6037555698	0,0092209465	0,0265996288
SYNE2	3248,747266	-0,7068741252	0,2398623601	-2,9469889574	0,0032087429	0,0112737350
SYNGR2	1550,611128	0,7179601125	0,2310922829	3,1068112846	0,0018911708	0,0073315552
SYNGR3	109,0188839	-0,6575481813	0,2388119171	-2,7533331812	0,0058901782	0,0183986329
SYNJ2BP	173,7257716	1,1224695032	0,3046091099	3,6849472541	0,0002287500	0,0013068655
SYPL1	512,6787608	1,2826982623	0,2347340579	5,4644744529	0,0000000464	0,0000009213
SYT17	26,55638518	4,5335153360	0,9003349059	5,0353696472	0,0000004769	0,0000070544
SYT9-AS1	40,17381437	-0,8358088266	0,3127507675	-2,6724437271	0,0075301003	0,0226188686
SYTL1	1469,969285	-0,6586709756	0,2068491337	-3,1843061835	0,0014510144	0,0058738810
SZT2	722,7522976	-0,3826706418	0,1415685108	-2,7030759851	0,0068701039	0,0209353857
TAB3	305,192026	0,5136250898	0,1810648237	2,8366817406	0,0045583599	0,0149538201
TACC2	29,57577605	2,0713809628	0,6151654960	3,3671830179	0,0007593752	0,0034624627
TACC3	329,9525058	0,5481881324	0,1714224188	3,1978788782	0,0013844242	0,0056517524
TACSTD2	1216,010038	13,1809090538	1,7495887298	7,5380268549	0,0000000000	0,0000000000
TADA1	216,4891778	-0,3525884328	0,1464905264	-2,4070652986	0,0160812942	0,0417243699
TADA3	740,6687914	-0,4332218873	0,1320601279	-3,2804896836	0,0010362705	0,0044548711
TAF12	301,4783834	-0,5714718429	0,1390257506	-4,1105467183	0,0000394723	0,0003042942
TAF13	117,3241199	1,4316507719	0,4628914150	3,0928436462	0,0019824855	0,0076118662
TAF1C	524,9092885	-0,4739338422	0,1652556000	-2,8678837040	0,0041322740	0,0138531525
TAF1D	434,8456711	0,4465652042	0,1846643437	2,4182525478	0,0155952059	0,0407753441
TAF3	321,3053013	-0,4915588740	0,1950959680	-2,5195644945	0,0117500112	0,0323358541
TAF4B	34,19445347	1,5136795943	0,3225901240	4,6922688629	0,0000027019	0,0000316242
TAF5	94,45023718	-0,6434062553	0,2085818364	-3,0846710641	0,0020377739	0,0077899563
TAF5L	197,7391523	0,6929508569	0,2395037901	2,8932772075	0,0038124466	0,0130407956
TAF6	354,7587528	-0,4207781969	0,1428740035	-2,9450987844	0,0032285052	0,0113213278
TAF6L	160,4661078	-0,8335979383	0,1752807433	-4,7557873300	0,0000019767	0,0000241005
TAF8	275,955343	-0,6093137558	0,1529946223	-3,9825880361	0,0000681689	0,0004788898
TAF9	428,8043403	0,5749207717	0,1639349576	3,5070053402	0,0004531801	0,0022751971
TAFAZZIN	561,202826	-0,6707240944	0,1956618041	-3,4279765434	0,0006080978	0,0028861058
TAGAP	1819,131069	-1,3430706716	0,4301725533	-3,1221688172	0,0017952518	0,0070166118
TAGLN	119,5923758	1,8603656394	0,5737836361	3,2421029850	0,0011865114	0,0049728388
TANC1	71,29862861	4,3660121726	0,6635746319	6,5795421031	0,0000000000	0,0000000022
TAOK2	367,0079282	-0,4941636613	0,1369076236	-3,6094578162	0,0003068258	0,0016625909
TAP1	2759,784676	-1,1637906738	0,3216265268	-3,6184525060	0,0002963699	0,0016140822
TAPBP	4922,353941	-0,6322448314	0,2229077414	-2,8363520601	0,0045632114	0,0149624421
TAPT1	208,0434122	-0,4572572849	0,1892971316	-2,4155806932	0,0157172888	0,0364426582
TARBP1	293,4921964	0,9737072179	0,2835287804	3,4342447237	0,0005942075	0,0028366256
TATDN1	145,5177838	0,7980971648	0,2211865317	3,6082538961	0,0003082647	0,0016690640
TATDN3	121,4023408	0,5816673476	0,1785577211	3,3135815045	0,0009210596	0,0040580241
TBC1D1	515,0171692	0,7765753148	0,2084758191	3,7250138560	0,0001953045	0,0011428506
TBC1D10A	435,3325694	-0,9878473509	0,1610378790	-6,1342549045	0,0000000009	0,0000000285
TBC1D10B	333,6628194	-0,4938651166	0,1458792823	-3,3854369790	0,0007106503	0,0032828775
TBC1D10C	2771,89409	-1,2204040369	0,3325851793	-3,6694480476	0,0002430747	0,0013723934
TBC1D12	30,04128777	1,9828721816	0,4201988882	4,7190267881	0,0000023698	0,0000281526
TBC1D13	416,4614077	-0,6857356980	0,2525745519	-2,7149833301	0,0066279607	0,0202938202
TBC1D16	49,65570646	1,9928825048	0,3860853482	5,1617657286	0,0000002446	0,0000039258
TBC1D17	252,623814	-0,4887402616	0,1366565876	-3,5764120124	0,0003483425	0,0018395445
TBC1D2	259,005643	0,9400655613	0,3492802672	2,6991640059	0,0069513910	0,0211391728
TBC1D20	602,2729301	-0,5079446350	0,1649857222	-3,0787187410	0,0020789285	0,0079113309
TBC1D25	325,8824846	-0,8066601166	0,1706210638	-4,7277874058	0,0000022698	0,0000271327
TBC1D2B	1320,503611	-0,8559777825	0,3480555031	-2,4593140317	0,0139202796	0,0371758847
TBC1D5	541,6032985	0,3645021025	0,1234126494	2,9535278196	0,0031416429	0,0111046517
TBC1D7	101,4894443	0,5930691217	0,2495830950	2,3762391503	0,0174901229	0,0445950093
TBC1D8	97,38539445	2,1718871068	0,5026981361	4,3204797293	0,0000155690	0,0001386891
TBC1D8B	87,79629355	1,1437540030	0,2893045540	3,9534600730	0,0000770291	0,0005281773
TBC1D9	85,11815529	4,2635590377	0,7551097016	5,6462776585	0,0000000164	0,0000003785
TBCB	763,4487241	-0,3434414928	0,0993090751	-3,4583092463	0,0005435771	0,0026403995
TBCC	606,4288116	-0,5842526152	0,1899584908	-3,0756710955	0,0021002939	0,0079745996
TBCE	192,3674832	0,3293431547	0,1355941159	2,4288887237	0,0151451381	0,0398377401
TBL1X	315,8690469	-0,7694602347	0,2221867351	-3,4631240900	0,0005339421	0,0026020494
TBL2	492,577912	0,7890846435	0,2428005678	3,2498488426	0,0011546536	0,0048583222
TBL3	503,5589147	-0,5796117087	0,1636752874	-3,5412270473	0,0003982707	0,0020476408
TBP	170,2147302	-0,4583642743	0,1405679735	-3,2608016103	0,0011109774	0,0047157856
TBPL1	185,7718567	-0,4495852537	0,1702764346	-2,6403257429	0,0082826375	0,0244054949
TBRG1	528,641553	-0,4283714118	0,1598955505	-2,6790702456	0,0073826904	0,0222256795
TBRG4	462,3589307	-0,3728405839	0,1505647440	-2,4762807943	0,0132759121	0,0357816933
TBX19	57,08588096	-0,8085680932	0,1895577105	-4,2655563367	0,0000199405	0,0001714407
TBX21	1676,558274	-1,3937216513	0,2907346750	-4,7937923169	0,0000016366	0,0000203777
TC2N	968,5269733	0,9219117214	0,3031259915	3,0413483081	0,0023552121	0,0087529949
TCAF1P1	41,0451116	-1,1097611420	0,3409912845	-3,2545146820	0,0011358629	0,0047911985
TCEA2	115,1748552	0,6908010497	0,2315474090	2,9834108389	0,0028505508	0,0102425022
TCEA3	40,1358468	2,9408692793	0,6968432535	4,2202722987	0,0000244007	0,0002014932
TCEAL4	118,0445757	1,8400351943	0,3253765848	5,6550921241	0,0000000156	0,0000003627
TCEAL9	94,35382433	6,1431210738	0,7343252174	8,3656681380	0,0000000000	0,0000000000
TCEANC	91,12611605	-0,7828738698	0,2596048635	-3,0156363761	0,0025644059	0,0093897996
TCEANC2	151,8686536	-0,5719049336	0,2163306295	-2,6436613943	0,0082014655	0,0242246491
TCERG1	565,961181	0,5116993722	0,1196535717	4,2826674108	0,0000183838	0,0001597162
TCF25	1370,817834	-0,4411671609	0,1563232772	-2,8221463164	0,0047703408	0,0155093688
TCF4	156,6083864	1,6205591719	0,3129281442	5,1786842199	0,0000002234	0,0000036455
TCF7	1120,853814	-0,7581115695	0,2943882958	-2,5752087387	0,0100179608	0,0284296336
TCF7L2	57,63222593	1,5006008221	0,4662037673	3,2187659931	0,0012874350	0,0053188691
TCFL5	60,68687165	-0,7085971379	0,2447911402	-2,8947009160	0,0037951995	0,0129965809
TCHP	248,8813102	-0,7485862904	0,1509565372	-4,9589531164	0,0000007087	0,0000098959
TCN2	150,5268524	3,0449993725	0,5911761492	5,1507480077	0,0000002594	0,0000041328
TCP1	1199,156484	0,7163915806	0,1624238736	4,4106298214	0,0000103070	0,0000978915
TCTA	469,2797033	0,5237855380	0,1816437745	2,8835865115	0,0039317463	0,0133403846
TCTN1	108,9580611	0,9804262347	0,2667413231	3,6755693619	0,0002372195	0,0013444113
TDP1	258,7686	-0,5634880345	0,1894730070	-2,9739752557	0,0029396878	0,0104955432
TEAD3	44,46412719	3,7716631209	0,9372960670	4,0239826602	0,0000572221	0,0004158801
TEC	97,9734129	1,2291518761	0,3655996184	3,3620162989	0,0007737556	0,0035161208
TECPR2	106,4920327	-0,4362854365	0,1479601061	-2,9486693936	0,0031914518	0,0112364483
TEDC1	113,0645511	-0,5129050136	0,2115790462	-2,4241767925	0,0153431341	0,0402450048
TEFM	90,13657368	-0,8120990014	0,2452494363	-3,3113185243	0,0009285745	0,0040805133
TENT4A	199,0628126	0,4298595438	0,1824342109	2,3562441588	0,0184607793	0,0465758369
TEPSIN	328,682962	-0,8213712607	0,1631569884	-5,0342395313	0,0000004797	0,0000070728
TERF2	207,7208983	-0,5541915537	0,1790552373	-3,0950853635	0,0019675626	0,0075645223
TERF2IP	1155,088562	-0,5759037770	0,2109059843	-2,7306184736	0,0063215607	0,0195290813
TES	1597,811158	-0,3383530206	0,1372759701	-2,4647651028	0,0137103099	0,0367464099
TESK1	304,3904343	-1,0335052504	0,2011254115	-5,1386109919	0,0000002768	0,0000043674
TESK2	89,64442422	-0,7771782205	0,3269456474	-2,3770899744	0,0174498311	0,0445275753
TESPA1	393,7719252	-1,5923921818	0,3424282319	-4,6502933121	0,0000033146	0,0000376816
TET1	45,27898804	-1,0394178163	0,3281378715	-3,1676252776	0,0015369948	0,0061622292
TEX2	189,4915005	0,5423457028	0,2055480326	2,6385351185	0,0083265075	0,0244972222
TFAP2E-AS1	73,38276661	-1,0579198693	0,3674587448	-2,8790088827	0,0039892710	0,0134913536
TFB1M	68,68295512	-0,5610246295	0,1599539742	-3,5074128810	0,0004524865	0,0022725627
TFDP1	299,7745365	0,9758798417	0,2345719955	4,1602572367	0,0000317889	0,0002518417
TFEB	309,826464	-1,5548618888	0,2820373051	-5,5129653442	0,0000000353	0,0000007363
TFEC	33,55435737	1,7716104257	0,7357754663	2,4078138552	0,0160483569	0,0416021432
TFG	718,7728341	0,9392776895	0,1958679357	4,7954642818	0,0000016230	0,0000203835
TFIP11	681,5625085	-0,7914441308	0,1899189000	-4,1672252504	0,0000308293	0,0002452499
TFPI	72,66459013	1,0081294736	0,3748806007	2,6892014985	0,0071623171	0,0216591302
TFRC	930,5782919	2,1796856343	0,4519551914	4,8227914528	0,0000014156	0,0000180953
TG	12,63300991	1,1019322633	0,4533000677	2,4309113142	0,0150609005	0,0396401489
TGFA	93,69431602	1,3972237150	0,4687367994	2,9808364845	0,0028746224	0,0103097302
TGFB1I1	15,46926819	2,5368810775	0,5979915459	4,2423360247	0,0000221205	0,0001867893
TGFBI	1605,064438	2,2476487912	0,6740013488	3,3347828177	0,0008536574	0,0038186202
TGFBR2	1912,126285	-0,4798998797	0,1611141464	-2,9786327966	0,0028953754	0,0103626720
TGFBR3	1670,968259	-1,3252108631	0,3294749532	-4,0221884852	0,0000576599	0,0004181584
TGM1	123,076272	3,2472992759	0,7119321273	4,5612483989	0,0000050850	0,0000541064
TGM2	784,0567619	5,2699378768	0,7496774533	7,0296070044	0,0000000000	0,0000000001
TGOLN2	2297,552841	0,4139883493	0,1735006836	2,3860493378	0,0170304661	0,0436711895
THAP1	126,1452394	-0,7649922288	0,1587886431	-4,8176765914	0,0000014524	0,0000185125
THAP11	541,1145732	-1,1145789258	0,2538682907	-4,3907765027	0,0000112947	0,0001054273
THAP3	127,4162028	-1,4014497904	0,1931870364	-7,2543676699	0,0000000000	0,0000000000
THAP6	95,74588627	-0,5198859825	0,1963543605	-2,6476925759	0,0081042186	0,0240220500
THAP7	217,8619563	-0,4726656655	0,1822390809	-2,5985955552	0,0093605988	0,0268913596
THAP8	69,67080349	-0,6008248182	0,2395330109	-2,5083173962	0,0121307649	0,0332275327
THAP9-AS1	285,7203883	0,7898492448	0,2511930538	3,1443812672	0,0016643275	0,0065846053
THBD	225,3499052	3,5671342959	1,0110767539	3,5280549000	0,0004186253	0,0021302516
THBS3	222,5475583	1,3439890615	0,1812617876	7,4146309560	0,0000000000	0,0000000000
THNSL2	33,42616636	2,2330774343	0,5730896974	3,8965583301	0,0000975693	0,0006421922
THOC7	229,4117847	0,5347365618	0,1452070260	3,6825804957	0,0002308849	0,0013157117
THRAP3	1261,308937	-0,5022636893	0,1779669854	-2,8222258573	0,0047691577	0,0155092686
THUMPD1	681,18307	-0,2834831886	0,1163941154	-2,4355457112	0,0148693457	0,0392434105
THUMPD2	85,81717004	1,1113207289	0,2261484171	4,9141211907	0,0000008918	0,0000121743
THUMPD3	212,1451815	0,5106697889	0,1457843827	3,5029112472	0,0004602028	0,0023025810
THUMPD3-AS1	206,9055291	-0,5083429769	0,1716286802	-2,9618764568	0,0030577045	0,0108525607
THY1	202,4777293	5,2199017716	1,0680394986	4,8873677224	0,0000010219	0,0000136868
THYN1	270,8329069	-0,8903257737	0,1990438022	-4,4730142997	0,0000077125	0,0000770674
TIAM1	203,5415599	0,8380993160	0,2764540043	3,0316049066	0,0024325737	0,0089976484
TICRR	14,19724562	1,7566516990	0,7190829392	2,4429055444	0,0145695500	0,0385807874
TIGD7	60,58293205	-0,8603610835	0,2938995061	-2,9273988751	0,0034181020	0,0118944293
TIGIT	1001,89388	-2,0030419698	0,3810489045	-5,2566529938	0,0000001467	0,0000025154
TIMM13	198,6719475	0,4952828830	0,1435282901	3,4507686089	0,0005589826	0,0027055162
TIMM22	225,2968087	-0,7594232333	0,2266252514	-3,3510088955	0,0008051772	0,0036368069
TIMM23	409,9847652	0,3988967863	0,1598794646	2,4949845014	0,0125962632	0,0342518571
TIMM29	137,780703	-0,6631972599	0,1199102729	-5,5307793370	0,0000000319	0,0000006733
TIMM9	93,33223874	0,6987676519	0,1711106449	4,0837181835	0,0000443208	0,0003343933
TIMP1	5132,726815	2,8835004653	0,5258925771	5,4830598315	0,0000000418	0,0000008487
TIMP2	151,2253194	3,5840588068	0,5550347546	6,4753761402	0,0000000001	0,0000000040
TIMP3	218,53761	9,3293018480	1,3044234599	7,1520500317	0,0000000000	0,0000000001
TINAGL1	174,1558265	6,8850638260	1,1565965960	5,9615114378	0,0000000025	0,0000000727
TINF2	1281,439824	-0,6907999805	0,1824538589	-3,7861626210	0,0001529916	0,0009320614
TIPARP	564,5890605	1,1125098222	0,4385045275	2,5370545856	0,0111789522	0,0310666282
TJP1	101,7923648	7,8285195959	1,0733934340	7,2932434165	0,0000000000	0,0000000000
TJP2	310,6518877	3,1728825700	0,6004967186	5,2837474446	0,0000001266	0,0000022211
TK1	117,054116	2,0586042247	0,4809105705	4,2806383366	0,0000186258	0,0001614143
TKT	582,1223841	0,5666460182	0,2080782887	2,7232332700	0,0064646394	0,0199063946
TLCD3A	31,28022343	1,8118173977	0,5881222809	3,0806809206	0,0020652784	0,0078736294
TLE4	526,3082412	-0,9847473789	0,3113701208	-3,1626264470	0,0015635282	0,0062541127
TLE5	2974,735336	-0,4215780037	0,1749878557	-2,4133217626	0,0158078574	0,0412112649
TLN2	18,96669301	1,9926750780	0,4800454023	4,1510137755	0,0000331006	0,0002605461
TLR2	222,3480332	2,7231050455	0,6070759590	4,4856084402	0,0000072706	0,0000734703
TLR3	51,84750616	1,1490001197	0,4784072870	2,4017195201	0,0163182149	0,0421822878
TLR4	181,9892942	1,9890116077	0,7974254857	2,4942914960	0,0126208851	0,0343047483
TLR5	47,21085836	2,3674217663	0,6145009528	3,8525825071	0,0001168738	0,0007466167
TLR7	53,32151334	4,5282580070	0,7781985991	5,8188876800	0,0000000059	0,0000001561
TM4SF1	1219,305647	12,3510884384	1,3350074022	9,2517003413	0,0000000000	0,0000000000
TM4SF19	21,32375712	-2,4216096695	0,7991789838	-3,0301221737	0,0024445483	0,0090300231
TM9SF2	2036,477376	0,9844005787	0,2284952548	4,3081863941	0,0000164599	0,0001455649
TM9SF3	1558,135676	0,4934182553	0,1855681804	2,6589593874	0,0078382411	0,0233568131
TM9SF4	648,6868415	0,5435425759	0,2152252497	2,5254591484	0,0115547215	0,0318636654
TMC4	172,8172101	3,9242240912	0,8337094914	4,7069442431	0,0000025146	0,0000298896
TMCC1	114,4167048	0,7527501620	0,3001347888	2,5080736726	0,0121391355	0,0332368321
TMCO3	429,8743812	0,4399023086	0,1565498750	2,8081880597	0,0049821123	0,0160582452
TMCO6	146,8725807	-0,6352739144	0,1473085815	-4,3125381073	0,0000161391	0,0001430101
TMED2	1941,12547	0,5088633453	0,1773027292	2,8700254502	0,0041043876	0,0137940218
TMED3	414,6308087	1,0914662746	0,2763285064	3,9497427002	0,0000782349	0,0005350823
TMED7	509,7100666	0,9185453908	0,2758775968	3,3295396266	0,0008698968	0,0038745240
TMED7-TICAM2	56,57857239	1,3326365801	0,4013161344	3,3206653459	0,0008980315	0,0039787701
TMEM101	158,0690156	-0,7042517050	0,2411862550	-2,9199483741	0,0035008938	0,0121337358
TMEM102	91,43524165	-1,2006977836	0,2801273487	-4,2862589092	0,0000181709	0,0001584068
TMEM107	151,4570201	-0,9282781387	0,2289781409	-4,0540032996	0,0000503485	0,0003725623
TMEM109	1034,196738	-0,5198560837	0,2105212524	-2,4693573239	0,0135393818	0,0363680062
TMEM11	231,2882248	-0,6478559440	0,1716241259	-3,7748535678	0,0001601017	0,0009661643
TMEM115	338,8525284	-0,5624950879	0,1561489431	-3,6023445404	0,0003153600	0,0016978982
TMEM123	1456,716499	0,9532304203	0,2008991218	4,7448212415	0,0000020869	0,0000252279
TMEM126B	215,8940614	-0,5834988631	0,1779781862	-3,2784858393	0,0010436560	0,0044780395
TMEM129	440,3547338	-0,6819758201	0,1709632610	-3,9890150495	0,0000663482	0,0004685451
TMEM131	437,2925037	0,2369505796	0,1002920306	2,3626052618	0,0181469382	0,0459044884
TMEM131L	622,2105669	-0,8004995403	0,3311273663	-2,4174973796	0,0156276465	0,0408205166
TMEM132A	55,10878055	4,5461394245	0,7056515139	6,4424710139	0,0000000001	0,0000000049
TMEM140	396,6668546	-1,5595254855	0,2723291041	-5,7056693265	0,0000000116	0,0000002795

TPRA1	**333,9410791**	-0,8467321701	0,1506591880	-5,6201827565	0,0000000191	0,0000004323
TPST2	**1390,356664**	-0,7122087200	0,2916841492	-2,4417121123	0,0146177983	0,0386781138
TPT1	**10473,84164**	1,2525317805	0,2978244933	4,5413718855	0,0000055889	0,0000587711
TPX2	**135,3687544**	3,6399307618	0,5850989183	6,2211582685	0,0000000005	0,0000000173
TRA2B	**1820,336854**	0,6222226316	0,1591602202	3,9089211634	0,0000927092	0,0006165559
TRAF1	**686,2744412**	-1,7710480830	0,3939141653	-4,4960253760	0,0000069235	0,0000703228
TRAF2	**281,6702801**	-1,5034264886	0,2688494053	-5,5920941148	0,0000000224	0,0000004943
TRAF3IP2	**120,1254099**	0,9173124033	0,2120727776	4,3254604084	0,0000152214	0,0001362813
TRAF3IP3	**1978,207921**	-1,1493959823	0,2969954057	-3,8352138890	0,0001254551	0,0007912958
TRAF4	**164,5162625**	2,8598308807	0,6843445003	4,1789345562	0,0000292878	0,0002356328
TRAF6	**287,3713191**	-0,5060115674	0,1585593126	-3,1894772788	0,0014253034	0,0057888512
TRAM1	**1487,487098**	0,5773594719	0,2398622135	2,4070453727	0,0160821717	0,0417243699
TRAM2	**259,8343376**	0,7828505479	0,3249332885	2,4100071489	0,0159522079	0,0415152201
TRANK1	**874,8255776**	-1,0016638941	0,2431716698	-4,1191636130	0,0000380250	0,0002941475
TRAP1	**365,3632268**	0,6660314730	0,2145545098	3,1042529646	0,0019076016	0,0073782520
TRAPPC10	**1426,957542**	-0,4664863689	0,1417337627	-3,2912861403	0,0009973041	0,0043190842
TRAPPC2	**356,9353218**	-0,4015611070	0,1595667611	-2,5165711480	0,0118502971	0,0325984749
TRAPPC4	**422,5185856**	-0,7528704112	0,1805722589	-4,1693581062	0,0000305459	0,0002432825
TRBC1	**2913,24525**	-1,8051940064	0,3976271263	-4,5399173926	0,0000056276	0,0000590286
TRBC2	**2342,117523**	-1,3190549858	0,4278938593	-3,0826733246	0,0020515022	0,0078331239
TRD-AS1	**99,9601478**	-0,5900818616	0,2213972553	-2,6652627679	0,0076928187	0,0230356707
TREM1	**223,7625336**	2,8051773574	1,1532476519	2,4324154077	0,0149984945	0,0395367977
TREM2	**333,7569775**	7,5494728427	1,0569242018	7,1428715796	0,0000000000	0,0000000001
TREX1	**238,367767**	-1,1061923081	0,2559231356	-4,3223614993	0,0000154368	0,0001376934
TRG-AS1	**686,455207**	-1,1903389047	0,3328315194	-3,5763947679	0,0003483655	0,0018395645
TRGV7	**38,03759663**	-1,8687820518	0,5163184836	-3,6194366677	0,0002952451	0,0016105287
TRGV9	**196,7630752**	-1,3007973155	0,4689358724	-2,7739343308	0,0055382857	0,0175607361
TRIB1	**363,2969865**	3,7041065900	0,6609592558	5,6041375580	0,0000000209	0,0000004664
TRIM11	**418,1317145**	-0,5778886277	0,1270553215	-4,5483229949	0,0000054075	0,0000570864
TRIM13	**222,8712391**	-0,5073882784	0,1768291382	-2,8693703068	0,0041128997	0,0138123828
TRIM16	**101,524391**	1,8442852406	0,5122384804	3,6004425889	0,0003176760	0,0017069535
TRIM2	**82,53716218**	4,9582944909	0,8923382395	5,5565184982	0,0000000275	0,0000005908
TRIM21	**485,802802**	-1,0125972889	0,2341146470	-4,3252197239	0,0000152380	0,0001362813
TRIM23	**124,2171645**	-0,5782231404	0,1694987383	-3,4113713534	0,0006463699	0,0030335214
TRIM24	**242,6890544**	0,3725758436	0,1209782964	3,0796651479	0,0020723275	0,0078906726
TRIM27	**668,1639087**	-0,6150073588	0,2143718300	-2,8688814148	0,0041193621	0,0138198573
TRIM3	**149,1393702**	-0,4556684066	0,1646936753	-2,7667632388	0,0056615856	0,0179485316
TRIM35	**168,1361506**	-0,6545138667	0,2202089557	-2,9726941149	0,0029519848	0,0105328588
TRIM36	**15,83887707**	3,5591600985	0,8654788536	4,1123593983	0,0000391636	0,0003020871
TRIM4	**379,6796841**	-0,9020293971	0,2142520942	-4,2101310633	0,0000255223	0,0002092141
TRIM47	**124,8709074**	3,1568542343	0,5621137668	5,6160414859	0,0000000195	0,0000004412
TRIM5	**179,8175494**	-0,6725837897	0,2697012127	-2,4938167502	0,0126379898	0,0343373724
TRIM52	**426,1504874**	-0,6838085832	0,1563744828	-4,3729305836	0,0000122553	0,0001125531
TRIM56	**844,071277**	-0,8967422954	0,1645906134	-5,4483197843	0,0000000508	0,0000010109
TRIM58	**40,39085545**	1,7313784552	0,3052243547	5,6726302055	0,0000000142	0,0000003331
TRIM61	**28,68078893**	-1,0075661009	0,3222851825	-3,1263184147	0,0017700978	0,0069433063
TRIM62	**135,940904**	-0,4273525166	0,1808630139	-2,3628529023	0,0181349150	0,0458826986
TRIM65	**262,8925604**	-0,4209212113	0,1651295750	-2,5490358788	0,0108021186	0,0302154025
TRIM68	**184,5022848**	-0,9573251976	0,2270149184	-4,2170144781	0,0000247558	0,0002040697
TRIO	**336,1556393**	1,4837031389	0,3923521389	3,7815599609	0,0001559487	0,0009474811
TRIP10	**106,1421228**	1,9043018515	0,5795050392	3,2860833343	0,0010159095	0,0043841429
TRIP13	**47,75893561**	2,7978474375	0,5803130509	4,8212726443	0,0000014265	0,0000182164
TRIP4	**294,0858175**	-0,3537996479	0,1360474162	-2,6005613173	0,0093071385	0,0267965954
TRIP6	**129,398534**	3,7045915690	0,7035101713	5,2658678160	0,0000001395	0,0000024264
TRIQK	**49,65871231**	3,0927247925	0,4738205612	6,5272068072	0,0000000001	0,0000000030
TRMO	**210,87583**	-0,7715508543	0,2602315971	-2,9649557599	0,0030280289	0,0129216990
TRMT10A	**25,56993809**	1,1181232856	0,4211003194	2,6552420742	0,0079251514	0,0235688228
TRMT2A	**526,1919828**	-0,6542935656	0,1463584721	-3,1039769328	0,0019093714	0,0073808555
TRMT5	**35,34448591**	0,9718707732	0,3652777853	2,6606347612	0,0077993509	0,0232924925
TRMT6	**122,3217172**	0,7850834034	0,2194890453	3,5768882782	0,0003477353	0,0018376588
TRMU	**480,7739101**	-0,6833810289	0,1589339195	-4,3352162292	0,0000592081	0,0028310525
TRNAU1AP	**273,9044295**	-0,6129369803	0,1689936541	-3,6269885218	0,0002867483	0,0015702325
TROAP	**22,06217346**	2,5469268901	0,4988460645	5,1056369316	0,0000003297	0,0000051006
TRPC4AP	**1371,886824**	-0,3294357557	0,1246062957	-2,6438130887	0,0081977911	0,0242191106
TRPM4	**83,85033758**	4,0838866286	0,7184305106	5,6844560025	0,0000000131	0,0000003099
TRPS1	**141,0852459**	1,2624629752	0,3629851470	3,4780017486	0,0005051666	0,0024824910
TRPT1	**209,2985461**	-0,7071876670	0,2061627766	-3,4302407958	0,0006030458	0,0028661712
TRPV3	**34,71933343**	-1,4622282145	0,3621960598	-4,0371182256	0,0000541118	0,0003968182
TSC22D1	**773,2832471**	1,2436290591	0,3653191565	3,4070240880	0,0006567533	0,0030726102
TSEN15	**416,3001196**	-0,5545268753	0,1393351967	-3,9798047322	0,0000689719	0,0004832700
TSEN54	**991,5841856**	-0,5126598613	0,2043944110	-2,5081897246	0,0121351491	0,0332327785
TSKU	**27,42090193**	2,4362051282	0,6990984994	3,4847809590	0,0004925401	0,0024346637
TSPAN13	**119,2769835**	5,5414270169	0,6647120080	8,3365832874	0,0000000000	0,0000000000
TSPAN14	**2112,828273**	-0,6256713402	0,1483907785	-4,2163761556	0,0000248260	0,0002045026
TSPAN15	**150,6134325**	5,5066891231	0,8811490886	6,2497345864	0,0000000004	0,0000000149
TSPAN18	**21,75215837**	2,2622426549	0,5204530586	4,3466962476	0,0000138203	0,0001255208
TSPAN4	**143,9897494**	2,3285778875	0,4638737030	5,0198534354	0,0000005171	0,0000075246
TSPAN6	**119,8841261**	4,2339846729	0,7987189937	5,4261695377	0,0000000576	0,0000011167
TSPO	**826,6735368**	0,6087562537	0,2286270568	2,6626605884	0,0077525568	0,0231801190
TSPOAP1	**1713,574832**	-1,8365908481	0,2146170181	-8,5575266314	0,0000000000	0,0000000000
TSPOAP1-AS1	**545,623695**	-2,1267618370	0,3051780570	-6,9688983845	0,0000000000	0,0000000002
TSPYL4	**335,8290884**	1,2303242548	0,3543038937	3,4725112447	0,0005156134	0,0025292116
TSPYL5	**166,8471506**	1,4214544605	0,6036537726	2,3580585284	0,0183411241	0,0463347466
TSR1	**402,3405732**	0,5509025693	0,1746483549	3,1544975361	0,0016077474	0,0064024485
TSR2	**512,3230791**	-0,3793637967	0,1108209397	-3,4232140397	0,0006188533	0,0029288907
TSR3	**367,4926069**	-0,3920641142	0,0899528153	-4,3585530135	0,0000130925	0,0001194748
TSSC4	**415,0683075**	-0,7357127240	0,1508134542	-4,8782963567	0,0000010701	0,0000142604
TTC19	**437,0408775**	0,3499876822	0,1447685090	2,4175510759	0,0156253409	0,0408205166
TTC21A	**29,43614076**	2,2006451723	0,5193698490	4,2296856601	0,0000094371	0,0000907954
TTC23	**26,97468709**	1,7347031920	0,4730263314	3,6672444575	0,0002451784	0,0013825308
TTC24	**40,54236629**	-0,8263887023	0,3538839851	-2,3351966666	0,0195331492	0,0488147397
TTC26	**28,18812448**	1,5731611485	0,3607978221	4,3602290599	0,0000129926	0,0001186438
TTC27	**131,3860297**	0,7715739834	0,2181873706	3,5362908100	0,0004057877	0,0020767682
TTC30A	**42,95551471**	2,2350784725	0,5859285834	3,8145919105	0,0001364082	0,0008468883
TTC30B	**51,11361024**	1,1543924945	0,4822722011	2,3936529047	0,0166815266	0,0428889966
TTC31	**600,7722982**	-0,7576310986	0,1659718265	-4,5648175040	0,0000049993	0,0000535326
TTC38	**793,3807597**	-0,8553839525	0,2958966493	-2,8908200021	0,0038423812	0,0131131976
TTC7B	**51,85593292**	1,1685763275	0,3465451388	3,3720754304	0,0007460402	0,0034155445
TTC8	**49,95148892**	1,9959405135	0,3983437318	5,0105985217	0,0000005426	0,0000078113
TTC9C	**138,49294**	-1,0769065566	0,1838326142	-5,8580821529	0,0000000047	0,0000001276
TTI2	**161,1854849**	-1,0885335303	0,2749906693	-3,9627612142	0,0000740879	0,0005121843
TTK	**36,29911324**	3,5225800678	0,6696700412	5,2601726989	0,0000001439	0,0000024804
TTLL10	**17,24032155**	1,4625280052	0,3615018958	4,0456994058	0,0000521671	0,0003849614
TTLL11	**50,00790158**	-0,6110082672	0,2182625097	-2,7994186903	0,0051194550	0,0164027288
TTLL5	**158,9942704**	0,5108140584	0,1952175346	2,6166402553	0,0088799874	0,0258166282
TTLL7	**18,96311631**	1,1222160473	0,4835687838	2,3206382160	0,0203063778	0,0503637084
TTN	**253,9466505**	1,5352679018	0,3187241655	4,8169171597	0,0000014579	0,0000185480
TTYH3	**310,4578236**	1,4233492258	0,3717925727	3,8284452536	0,0001289553	0,0008088250
TUBA1A	**2485,584409**	1,6942127868	0,2608665226	6,4945580968	0,0000000001	0,0000000036
TUBA1B	**4789,006157**	1,5777988931	0,3467238754	4,5505819985	0,0000053495	0,0000566519
TUBA1C	**752,8397354**	2,5546142561	0,3833823411	6,6633592163	0,0000000000	0,0000000014
TUBB	**2619,261701**	1,1735841581	0,2304000879	5,0936792980	0,0000003512	0,0000053960
TUBB2A	**237,3940699**	3,1594150287	0,6667089032	4,7388222664	0,0000021496	0,0000258571
TUBB3	**96,594549**	3,5896729877	0,7495318186	4,7892202822	0,0000016743	0,0000207888
TUBB4B	**1539,832853**	1,6524091763	0,2557294612	6,4615518629	0,0000000001	0,0000000044
TUBB6	**323,9574978**	3,9439877283	0,5661274621	6,9666073318	0,0000000000	0,0000000002
TUBD1	**82,45067288**	-0,9466578742	0,2962158675	-3,1958378264	0,0013942545	0,0056799837
TUBG1	**225,3264842**	0,4548083250	0,1873234378	2,4280908492	0,0151785406	0,0398873792
TUBGCP5	**130,4686891**	0,7469618919	0,2419757962	3,0869085425	0,0020223621	0,0077398232
TUBGCP6	**1299,980053**	-0,6919236486	0,2344864637	-2,9508033752	0,0031694861	0,0111877829
TUFT1	**125,0140513**	3,2564078444	0,5857940920	5,5589432759	0,0000000271	0,0000005835
TUG1	**1144,582619**	0,6270234959	0,2046549031	3,0638088146	0,0021853852	0,0082465055
TUSC1	**21,9238568**	2,1826044751	0,5709553882	3,8227223163	0,0001319859	0,0008248272
TUSC2	**295,8300193**	-0,8768224508	0,0831152292	-10,5494800319	0,0000000000	0,0000000000
TUSC3	**136,8236368**	8,2918267286	1,2761529878	6,4975177803	0,0000000001	0,0000000036
TUT1	**320,8660175**	-0,9109630445	0,1699415495	-5,3604482071	0,0000000830	0,0000015476
TUT7	**821,8333326**	-0,4517588404	0,1611761033	-2,8028959083	0,0050646012	0,0162695782
TWF1	**449,2143563**	1,1201745118	0,3663702023	3,0574594985	0,0022319648	0,0083916890
TWSG1	**151,0808983**	1,1071356760	0,3737562796	2,9621853604	0,0030546383	0,0109483988
TXN	**362,3492242**	1,2202080513	0,3172674596	3,8459918105	0,0001200658	0,0007628961
TXNDC11	**542,5207599**	0,3219166571	0,1168216626	2,7556495944	0,0058575712	0,0183165196
TXNDC17	**218,601553**	0,7183680008	0,2497876451	2,8759148616	0,0040285836	0,0135881836
TXNDC5	**677,0045293**	1,6611186793	0,3320182210	5,0030949330	0,0000005642	0,0000080699
TXNDC9	**247,3619141**	-0,4195694320	0,1761485701	-2,3818930222	0,0172239002	0,0440580953
TXNRD3	**30,42106121**	0,8707451139	0,3622882024	2,4034597543	0,0162407546	0,0420466545
TYK2	**1788,053562**	-0,7030702398	0,1607012846	-4,3750101970	0,0000121427	0,0001120223
TYMP	**1287,149175**	1,0205237666	0,3989526407	2,5580323642	0,0105266295	0,0295615341
TYMS	**76,66204272**	2,0818601950	0,4278352218	4,8660315988	0,0000011386	0,0000151118
TYRO3	**28,73698142**	1,5089553043	0,5259620580	2,8689432766	0,0041184565	0,0138198573
TYSND1	**292,2544425**	-0,8562052483	0,2079538818	-4,1172842784	0,0000383263	0,0002962151
U2AF1L4	**283,7746521**	-0,4982482492	0,1534521426	-3,2469292433	0,0011665739	0,0049007754
UACA	**161,7811128**	5,4735581878	0,8274509432	6,6149647838	0,0000000000	0,0000000018
UAP1L1	**237,4889806**	0,7976871581	0,3073274982	2,5962114158	0,0094258048	0,0270168937
UBA52	**1752,689829**	0,7183124222	0,2429325055	2,9568394755	0,0031080988	0,0110092132
UBA7	**1789,817005**	-0,6857161992	0,2103382088	-3,2600958395	0,0011137457	0,0047200937
UBAP1L	**80,94041915**	-0,8489229455	0,2578604563	-3,2921796447	0,0009941409	0,0043081571
UBAP2	**211,5530061**	0,4405244431	0,1537730066	2,8647709565	0,0041731096	0,0139733618
UBAP2L	**965,0130312**	0,4547429559	0,1679238383	2,7079897208	0,0067716599	0,0206728378
UBASH3A	**87,53823845**	1,4223123244	0,5617325475	2,5320098164	0,0113410816	0,0314096623
UBASH3B	**268,0062628**	0,8683451810	0,3734229607	2,3253663962	0,0200523767	0,0498408503
UBC	**30816,94968**	0,5282193307	0,1767078840	2,9892221677	0,0027968777	0,0100927543
UBE2C	**138,1552287**	4,3012013285	0,6711413019	6,4087865364	0,0000000001	0,0000000059
UBE2D4	**90,01778874**	0,7579258182	0,2062013638	3,6756586145	0,0002372366	0,0013444113
UBE2E1	**160,1305837**	1,2788827748	0,3199188367	3,9975225848	0,0000640089	0,0004551904
UBE2E2	**110,4943141**	2,6237255449	0,4621681554	5,6769810666	0,0000000137	0,0000003220
UBE2F	**748,2681511**	-0,9281994757	0,1502371004	-6,1782307631	0,0000000006	0,0000000221
UBE2J1	**788,8427348**	0,4497821259	0,1835169555	2,4509022868	0,0142498630	0,0379163900
UBE2L6	**1401,811809**	-0,5439969125	0,2148061373	-2,5325128944	0,0113248204	0,0313710811
UBE2N	**578,8539235**	0,3638745765	0,1420303060	2,5619502384	0,0104086230	0,0293157703
UBE2O	**318,7754362**	-0,5725581403	0,1216651391	-4,7060246216	0,0000025259	0,0000297716
UBE2S	**367,3623051**	0,8428648173	0,3260757008	2,5848746629	0,0097414402	0,0277619703
UBE2T	**39,61256451**	1,0385000959	0,4093889676	2,5367205888	0,0111896223	0,0310797185
UBE3C	**507,5756184**	0,3736053656	0,1324099956	2,8215797759	0,0047787750	0,0155210676
UBL4A	**457,2427457**	-1,0688809168	0,2285885741	-4,6764539481	0,0000029188	0,0000337226
UBL7	**501,7985829**	-0,6604440531	0,2026652894	-3,2587905369	0,0011188824	0,0047344098
UBL7-DT	**92,95200876**	-0,6987281509	0,2335330625	-2,9919881299	0,0027716704	0,0100152171
UBOX5	**147,5272408**	-0,7156727588	0,1894976555	-3,7766839747	0,0001589002	0,0009610064
UBR1	**447,6107134**	-0,3694874039	0,1562350845	-2,3649451417	0,0180327418	0,0456757066
UBR7	**386,1717119**	-0,5005486968	0,1362207150	-3,6745490356	0,0002382699	0,0013488586
UBTD1	**36,22490192**	1,3875437634	0,4808271342	2,8857434714	0,0039049030	0,0132794325
UBXN1	**698,1438832**	-0,3863726186	0,1056813386	-3,6560155622	0,0002561658	0,0014301086
UBXN10	**46,53212241**	2,4896583879	0,7541280044	3,3013299682	0,0009622764	0,0041970968
UCHL3	**133,7023506**	1,0568294585	0,1687433711	6,2629391150	0,0000000004	0,0000000137
UCHL5	**326,3236884**	-0,3458816744	0,1261837592	-2,7410950243	0,0061234795	0,0190175437
UCK1	**211,6932923**	-0,5640146870	0,1421935563	-3,9665277518	0,0000729273	0,0005067637
UFM1	**613,2519354**	0,9018358208	0,2111732852	4,2705961597	0,0000194951	0,0001679823
UGCG	**247,852192**	1,7676399761	0,2866576265	6,1663804225	0,0000000007	0,0000000237
UGGT1	**603,744857**	0,4788367127	0,1826851472	2,6211038859	0,0087645601	0,0255514357
UGGT2	**51,15004904**	2,3003553470	0,5764506689	3,9905500664	0,0000659202	0,0004662566
UHRF1	**57,82437024**	1,9518976166	0,4846970446	4,0270466644	0,0000564818	0,0004116109
UHRF2	**651,7999546**	0,2911484797	0,1168865196	2,4908644786	0,0127432716	0,0345631799
ULBP3	**26,29903121**	1,4296296185	0,3678232631	3,8867297476	0,0001016038	0,0006641994
ULK3	**809,0937128**	-0,3536534684	0,1470894571	-2,4043359421	0,0162016766	0,0419702193
UNC119	**364,9394504**	-0,4952788972	0,1684200011	-2,9407368128	0,0032743261	0,0114652521
UNC119B	**159,2378047**	-0,4121165799	0,1734671856	-2,3815258118	0,0172410824	0,0440852906
UNC13B	**50,27323716**	3,8678065062	0,6193306514	6,2451398091	0,0000000004	0,0000000152
UNC13D	**1561,24853**	-0,5129848220	0,1900575803	-2,6990321420	0,0069526833	0,0211391728
UNC45A	**728,228126**	-0,7439643310	0,1264746848	-5,8823176608	0,0000000040	0,0000001114
UNC50	**399,2862419**	-0,6168297052	0,1341436035	-4,5982789263	0,0000042600	0,0000465792
UNC5B	**85,51799489**	6,2204776647	0,9768507384	6,3678896070	0,0000000002	0,0000000075
UNC5CL	**38,14841437**	-0,7788826723	0,2300412896	-3,3862731322	0,0007084883	0,0032774092
UNC93B1	**688,7948869**	1,4424782728	0,2498246666	5,7739625640	0,0000000077	0,0000001959
UNKL	**153,6807539**	-0,6709762079	0,1481665637	-4,5285264857	0,0000059396	0,0000615455
UPF3B	**123,4092853**	-0,6435137648	0,1600313817	-4,0211723336	0,0000579092	0,0004190889
UPK1B	**541,2297614**	6,6943999026	1,0728607757	6,2397657313	0,0000000004	0,0000000156
UPK3B	**259,4960417**	6,7825105892	1,2087109645	5,6113585450	0,0000000201	0,0000004496
UQCRB	**696,5084737**	0,7883388882	0,2770212205	2,8457707565	0,0044304077	0,0146231701
UQCRH	**425,8902624**	0,7180426820	0,2702139860	2,6573122390	0,0078766453	0,0234608643
UQCRQ	**495,8176171**	0,4048975857	0,1463643615	2,7663673142	0,0056684647	0,0179640916
URB1	**135,1808932**	0,8216948349	0,2816313198	2,9176259075	0,0035270722	0,0121979424
URB2	**94,38933154**	1,1031083708	0,3169778769	3,4800800031	0,0005012641	0,0024668157
URI1	**405,7503788**	0,5145943512	0,1972101420	2,6093706233	0,0090708939	0,0262681222
UROS	**361,855893**	0,3558802220	0,1294173832	2,7498636342	0,0059620016	0,0185888602
USP12	**297,8990672**	0,6392648177	0,1657744354	3,8562328150	0,0001151478	0,0007368899
USP14	**477,3622365**	0,3783508699	0,1422447343	2,6598585303	0,0078173479	0,0233255382
USP19	**890,4447451**	-0,5960837594	0,1878501362	-3,1907549901	0,0014190156	0,0057588349
USP21	**339,7959777**	-0,8954814397	0,2293979895	-3,9036150306	0,0000947665	0,0006280437
USP24	**825,0842688**	-0,3905780797	0,1253967076	-3,1154803610	0,0018364558	0,0071544702
USP28	**804,8874669**	-0,5624720186	0,2358962377	-2,3844144712	0,0171063239	0,0438558251
USP32	**236,9281988**	0,5839167455	0,1615635318	3,6751024570	0,0002378244	0,0013438688
USP40	**136,3897805**	0,9517949581	0,3194803050	2,9791976305	0,0028900434	0,0103512464
USP45	**67,03408641**	0,7245411017	0,2262550424	3,2009055071	0,0013699645	0,0056047788
USP48	**542,3029832**	0,5272704374	0,2175908254	2,4221068403	0,0154308131	0,0404315415
USP54	**79,71726822**	2,1606851936	0,5899657801	3,6623907120	0,0002498724	0,0014020572
USP6NL	**77,26130333**	2,6509403804	0,3645092836	7,2726341186	0,0000000000	0,0000000000
USPL1	**1095,104432**	0,4372834832	0,1844166433	2,3711714689	0,0177318027	0,0450831251
UTP20	**109,5792242**	1,1319843601	0,2594182270	4,3635480700	0,0000127970	0,0001171751
UTRN	**1782,151255**	-0,6251958279	0,1845545384	-3,3875938973	0,0007050858	0,0032635676
VAMP1	**564,7492575**	-0,8769990604	0,2042876594	-4,3047086580	0,0000167205	0,0001471928
VAMP3	**550,8696454**	0,7966486003	0,2480453719	3,2117051580	0,0013194972	0,0054329863
VARS1	**425,3876101**	0,6641315509	0,1901485507	3,4926984641	0,0004781661	0,0023793405
VASN	**84,47049173**	4,7472727349	0,7715043413	6,1532676885	0,0000000008	0,0000000255
VAT1	**609,7986787**	0,5901617665	0,2236496445	2,6387780219	0,0083205443	0,0244850298
VAV2	**104,0286872**	1,8575517384	0,4790080304	3,8779297555	0,0001053491	0,0006849611
VCAM1	**274,7106183**	6,7384136371	1,0152091852	6,6374631643	0,0000000000	0,0000000016
VCAN	**1257,612515**	4,0270158172	1,2771799853	3,1530527126	0,0016157263	0,0064266181
VDAC1	**1136,90777**	0,7490731756	0,2181309558	3,4340525980	0,0005946288	0,0028366356
VDAC2	**1026,800517**	0,6234587942	0,1900113292	3,2811664272	0,0010337873	0,0044456155
VDR	**52,71047692**	2,2699879574	0,5463706647	4,1546666285	0,0000325762	0,0002570520
VEGFA	**297,8157184**	5,3199373395	0,9771627206	5,4440240374	0,0000000521	0,0000010311
VEZT	**275,6327417**	0,9136084714	0,2163861969	4,2221199149	0,0000242015	0,0002000937
VGLL3	**24,22240308**	2,5637595682	0,8570832759	2,9912804373	0,0027782846	0,0100327298
VIM	**4940,796572**	1,3608133750	0,4104575850	3,3153568717	0,0009152620	0,0040404809
VIPR2	**50,20652232**	-1,7922844450	0,4459143519	-4,0193468486	0,0000583597	0,0004220965
VMA21	**268,9831582**	0,5613973425	0,1721776873	3,2417417704	0,0011880162	0,0049748021
VMO1	**52,49811093**	4,8255520089	0,8635626683	5,5879581022	0,0000000230	0,0000005028
VMP1	**632,5789836**	1,0551912640	0,2765186262	3,8159861370	0,0001356401	0,0008428975
VNN2	**253,1951184**	-1,0882702010	0,3515635407	-3,0955149635	0,0019647146	0,0075600510
VPS11	**636,5167125**	-0,5925930491	0,1567267706	-3,7812030072	0,0001560723	0,0009484125
VPS16	**737,4914597**	-0,5346911499	0,1845641232	-2,8970481400	0,0037669195	0,0129211866

VPS18	475,6697161	-0,6238911207	0,2241574228	-2,7832721881	0,0053813639	0,0171196309
VPS26B	877,524001	-0,4924069230	0,1950669899	-2,5243088188	0,0115926041	0,0319393712
VPS28	1086,272429	-0,3357486708	0,1347934418	-2,4908383249	0,0127442097	0,0345631799
VPS37C	42,12858513	1,9210951998	0,5067238424	3,7912074371	0,0001499167	0,0009163844
VPS51	742,2536285	-0,6292137265	0,1404315403	-4,4805727062	0,0000074443	0,0000748881
VPS54	241,6069506	0,5423313165	0,1463584464	3,7054753186	0,0002109947	0,0012230785
VPS8	367,7257064	0,4490111796	0,1160881542	3,8678133439	0,0001098157	0,0007088299
VRK3	571,1269513	-0,8588386936	0,1211762334	-7,0875176521	0,0000000000	0,0000000001
VSIG10	31,12502329	2,8401591421	0,6406042608	4,4335626782	0,0000092689	0,0000895612
VSIG4	3232,798545	10,9519421430	0,9664428608	11,3322189886	0,0000000000	0,0000000000
VWA7	18,73652674	3,2171497912	0,6112482196	5,4258448435	0,0000000574	0,0000011141
VWA8	171,729843	0,7098271451	0,2774765987	2,5581513845	0,0105230271	0,0295615341
VWF	13,77633002	-2,1580400078	0,5648080843	-3,8208376751	0,0001329992	0,0008298346
WASF1	28,99049892	1,6667193606	0,5473241987	3,0451584506	0,0023255775	0,0098561887
WASH5P	1220,172677	-0,4398567231	0,1591949410	-2,7630089163	0,0057271553	0,0179884847
WASHC3	258,3608061	-0,4241342980	0,1639869165	-2,5863926575	0,0096986374	0,0276692793
WASHC5	490,273221	0,4156998194	0,1392423838	2,9854402675	0,0028317044	0,0101883830
WASL	144,85213	1,5414089990	0,4689222910	3,2871309887	0,0010121374	0,0043690653
WASL-DT	19,39620696	1,7965278214	0,3114384999	5,7684837364	0,0000000080	0,0000002016
WBP1L	479,6083256	-0,4032925201	0,1596219667	-2,5265477459	0,0115189730	0,0317870341
WDCP	71,13834475	-1,0559371389	0,1917081782	-5,5080442763	0,0000000363	0,0000007537
WDFY3	65,25468312	4,9025764801	0,8705259884	5,6316764712	0,0000000178	0,0000004085
WDFY4	123,1722001	3,0488811289	0,8147340649	3,7421793786	0,0001824312	0,0010803010
WDPCP	161,6140853	0,4485239453	0,1695721498	2,6450320778	0,0081682932	0,0241526282
WDR12	149,822593	0,7570557321	0,2185481215	3,4640230572	0,0005321609	0,0025961881
WDR37	476,9734176	-0,5818105313	0,1845452891	-3,1527561501	0,0016173685	0,0064290504
WDR41	228,2142684	0,5311090739	0,2043882300	2,5985293478	0,0093624042	0,0268913586
WDR43	293,2193306	1,2579773619	0,2452925445	5,1187820285	0,0000003075	0,0000047960
WDR45	1011,212511	-0,8909178029	0,1632901284	-5,4560420250	0,0000000487	0,0000009737
WDR45B	797,5271158	0,6298837284	0,2229382690	2,8253729325	0,0047225530	0,0153874843
WDR53	92,45115088	-0,4529653014	0,1795772668	-2,5223977921	0,0116557817	0,0320963219
WDR5B-DT	128,635369	-0,7957684995	0,2881053099	-2,7620750895	0,0057435267	0,0180273723
WDR6	1687,124881	-0,6225927116	0,2581165923	-2,4120212733	0,0158643559	0,0413420424
WDR7	364,2914086	-0,4788386807	0,2021722757	-2,3684585086	0,0178618980	0,0453453691
WDR72	29,24837065	1,6749888181	0,6299360316	2,6589823952	0,0078377058	0,0232568131
WDR75	242,6611185	0,5507858092	0,2162132990	2,5474178126	0,0108523413	0,0303243749
WDR81	523,8072847	-0,6765006934	0,2219999518	-3,0473093821	0,0023090630	0,0086189650
WDR82	1412,973419	-0,4156561797	0,1312025408	-3,1680497729	0,0015346524	0,0061550721
WDR83	148,0066155	-1,0196585513	0,2103018085	-4,8476023984	0,0000012496	0,0000163778
WDR86	23,78256031	1,9720098162	0,5988817232	3,2927704316	0,0009938200	0,0043092571
WDR86-AS1	11,07547093	1,8248932406	0,6244826126	2,9219080147	0,0034787193	0,0120834999
[illegible]						
ZNF71	129,7051497	-0,6910437705	0,2434881778	-2,8380988899	0,0045382978	0,0149025342
ZNF718	80,32200381	-0,8013282004	0,2371499362	-3,3789981697	0,0007275049	0,0033477663
ZNF737	197,7092296	-1,0453409060	0,2855666860	-3,6605842948	0,0002516407	0,0014071809
ZNF740	309,3244885	-0,4895248017	0,1767651297	-2,7694173613	0,0056070517	0,0177244987
ZNF746	261,589648	-0,6195427411	0,2145577161	-2,8875341901	0,0038827441	0,0132274702
ZNF747-DT	26,53831937	-1,3975980884	0,3976823546	-3,5143578087	0,0004408186	0,0022264234
ZNF750	238,0409832	-0,3887650986	0,1592403595	-2,4413729021	0,0146315376	0,0387068586
ZNF76	619,4794588	-0,6168386474	0,1743920021	-3,5370799126	0,0004045772	0,0020713616
ZNF763	60,08016816	-1,2861218100	0,3354647455	-3,8338508805	0,0001261526	0,0007945786
ZNF764	97,19238803	-0,9274762856	0,2389070947	-3,8821630089	0,0001035314	0,0006741815
ZNF766	353,5138858	-0,5421086084	0,1715466536	-3,1601234816	0,0015770229	0,0062905212
ZNF768	76,29836531	1,3010813971	0,4305604416	3,0218321778	0,0025124980	0,0092374278
ZNF771	35,58005213	-1,4951916442	0,3644370088	-4,1027437065	0,0000408279	0,0003131306
ZNF772	75,0252321	-0,8955551950	0,3854966179	-2,3231207574	0,0201726662	0,0500874537
ZNF773	115,2260731	-0,9227267017	0,2522290264	-3,6582891144	0,0002539045	0,0014192504
ZNF775	57,08461419	-1,5362502707	0,3157187026	-4,8658675978	0,0000011396	0,0000151118
ZNF776	196,6253881	-0,7891703857	0,2677727526	-2,9471646310	0,0032070241	0,0112737350
ZNF778	153,3060214	-0,8136425119	0,2701658370	-3,0116410011	0,0025983970	0,0094961778
ZNF780A	105,5895576	-0,6571582006	0,2496359068	-2,6324666556	0,0084767343	0,0248522858
ZNF786	142,8665216	-1,5897358896	0,3353711858	-4,7402278119	0,0000021348	0,0000257156
ZNF79	113,8006867	-1,0329166550	0,2895642119	-3,5671407436	0,0003608977	0,0018916209
ZNF790-AS1	53,15567698	-0,7706614187	0,2937214977	-2,6238934915	0,0086982023	0,0253854733
ZNF792	251,1644458	-1,2557088767	0,3600003835	-3,4880764972	0,0004865089	0,0024128258
ZNF799	98,37033329	-0,9699489514	0,2419424423	-4,0090070281	0,0000609746	0,0004379499
ZNF80	64,30951462	-1,9168278429	0,6086606906	-3,1492551967	0,0016368719	0,0064915425
ZNF816	162,1065226	-0,8418705726	0,2882374032	-2,9207540839	0,0034918533	0,0121164580
ZNF821	70,93626836	-0,7508415279	0,3012153313	-2,4927088777	0,0126773455	0,0344095725
ZNF830	188,009187	-1,1302226225	0,1525905185	-7,4068984179	0,0000000000	0,0000000000
ZNF831	297,7111916	-0,7604882113	0,3192242891	-2,3823005868	0,0172049474	0,0440284073
ZNF835	22,73237742	-2,0465236877	0,5837487904	-3,5058234218	0,0004551971	0,0022826202
ZNF836	142,4445824	-0,8571066257	0,2915039989	-2,9402911417	0,0032790398	0,0114787714
ZNF879	101,5204264	-0,8536767403	0,3063871261	-2,7862683105	0,0053318718	0,0170023679
ZNF91	463,4716121	-0,7686263895	0,2470563959	-3,1111373850	0,0018636821	0,0072463032
ZNG1A	191,9246876	1,1116229446	0,2013729493	5,5202198122	0,0000000339	0,0000007088
ZNG1B	254,364974	0,8195004106	0,1235521193	6,6328316785	0,0000000000	0,0000000016
ZNG1C	98,00625033	0,8562854458	0,2583958071	3,3138519365	0,0009202022	0,0040556281
ZNG1E	243,1267238	1,4384937966	0,2353005551	6,1134313764	0,0000000010	0,0000000319
ZNG1F	45,63807588	0,9616297843	0,3357732897	2,8639257910	0,0041842603	0,0139891066
ZNHIT2	66,58991458	-0,7955679559	0,2098007954	-3,7920159182	0,0001494293	0,0009142358
ZNRD2	251,067208	-0,5094358177	0,1819336886	-2,8001181181	0,0051083911	0,0163826791
ZNRF1	207,653088	1,2597068170	0,4402142031	2,8615771782	0,0042153886	0,0140582582
ZP3	25,34776316	1,7184253616	0,4752009736	3,6162073673	0,0002988509	0,0016244971
ZPR1	467,344489	0,7045893190	0,2269910603	3,1040399477	0,0019089755	0,0073808555
ZSCAN16	109,5158707	-1,0936525854	0,2350659235	-4,6516848082	0,0000032923	0,0000375550
ZSCAN18	332,2921846	-0,7200178903	0,3017343692	-2,3862640909	0,0170205235	0,0436540105
ZSCAN22	128,3483862	-1,2260291948	0,2759959602	-4,4421997838	0,0000089044	0,0000867099
ZSCAN25	247,3813216	-0,3895202032	0,1396210688	-2,7898382852	0,0052734374	0,0168279913
ZSCAN26	258,3559005	-0,5371213316	0,2309749370	-2,3254528764	0,0200477569	0,0498428503
ZSCAN29	228,0115804	-0,5800298004	0,1290155177	-4,4958142308	0,0000069304	0,0000703495
ZSCAN31	35,31749736	2,3507958732	0,6711267892	3,5026820039	0,0004605817	0,0023046207
ZSCAN32	216,7112722	-0,7795862376	0,2637437646	-2,9558474639	0,0031181126	0,0110330692
ZSWIM3	57,29634072	-1,2789727888	0,4532182371	-2,8219793512	0,0047728250	0,0155136983
ZSWIM8	817,7670594	-0,8003560070	0,1504252985	-5,3206293316	0,0000001034	0,0000018734
ZWINT	118,0859479	3,1923332258	0,5057473190	6,3121106248	0,0000000003	0,0000000103
ZXDC	411,5374298	-0,4326841886	0,1479816747	-2,9239038509	0,0034567150	0,0120132673
ZYX	1743,521368	0,5199012316	0,2220249000	2,3416347968	0,0191994915	0,0481238650
