# Supplementary material for: Ovarian Cancer‐Associated Ascites Accumulates NK Lymphocytes Displaying a Transcriptomic Profile Consistent With the Immature/Secretory CD56bright Subtype
Source: Immunol Cell Biol. 2026 Aug 20;104(9):896–905. doi: 10.1111/imcb.70159 (PMC13628415; doi:10.1111/imcb.70159)
Supplement: Supplementary file 6 [file IMCB-104-896-s006.pdf]

Enriched biological processes associated with NK1 and NK2 scores						
**Score**	**GO pathway**	**NES**	**FDR**	**Pathway size**	**Leading-edge size**	**Leading-edge genes**
NK1	ANTIGEN RECEPTOR MEDIATED SIGNALING PATHWAY	2,30	2,81E-08	190	67	PTPN6, FYN, TXK, SKAP1, IKBKG, FCMR, INPP5D, LPXN, DUSP22, NFATC2, ZAP70, LAT, LCK, TRGC1, ADA, ITPRIPL1, TRBC2, TRBC1, CSK, PVRIG, GPS2, FCRL3, PRKCH, TRAF6, LAX1, PTPRC, CARD11, FOXP1, PTPRJ, CD79B, PRKCB, RFTN1, TESPA1, CLEC2D, RAB29, CBL, CD79A, ITK, CD226, LAT2, PRKD2, DGKZ, CRKL, CMTM3, CD72, RNF31, HLA-A, MAPK1, BTN3A2, LCP2, EIF2B5, BTN3A3, IKBKB, VAV3, SH2D1A, CLEC2B, CD8A, NCK1, TRGC2, THEMIS2, PTPN22, PLEKHA1, MALT1, RC3H1, PIK3CD, BCL2, NCKAP1L
NK1	NATURAL KILLER CELL ACTIVATION	2,12	1,05E-03	79	33	FYN, ELF4, SLAMF7, IL12RB1, CD244, RAB27A, IL2RG, FCGR3A, PTPRC, HLA-E, HLA-F, KLRD1, NCR1, KLRK1, STAT5B, UNC13D, ZBTB1, CASP8, FLT3LG, FGR, IL12RB2, CORO1A, BLOC1S3, NCR3, IL21R, SH2D1A, PTPN22, HAVCR2, RASGRP1, DCAF15, PIK3CD, VAMP2, TOX
NK1	LEUKOCYTE MEDIATED CYTOTOXICITY	1,99	1,05E-03	112	49	PTPN6, GZMM, INPP5D, SLAMF7, IL12RB1, SLAMF6, RAB27A, FCGR3A, PTPRC, HLA-E, HLA-F, TIGIT, IL18RAP, KLRD1, NCR1, KLRK1, MR1, TGFB1, ITGAM, STAT5B, P2RX7, CD226, UNC13D, LYST, SERPINB9, DNASE1, CORO1A, HLA-A, NCR3, CRTAM, HCST, KLRC4, SH2D1A, NLRP6, HAVCR2, RASGRP1, CYRIB, VAMP2, NCKAP1L, CTSC, LILRB1, AGER, HLA-C, KIR3DL1, POMC, VAV1, ARRB2, RIPK3, B2M
NK1	NATURAL KILLER CELL DEGRANULATION	1,98	3,10E-02	9	6	RAB27A, FCGR3A, HLA-F, UNC13D, CORO1A, VAMP2

NK1	REGULATION OF ANTIGEN RECEPTOR MEDIATED SIGNALING PATHWAY	2,29	1,14E-04	74	38	PTPN6, FYN, IKBKG, FCMR, LPXN, DUSP22, LCK, ADA, ITPRIPL1, CSK, PVRIG, GPS2, FCRL3, PRKCH, PTPRC, CARD11, FOXP1, PTPRJ, TESPA1, RAB29, CBL, CD226, PRKD2, DGKZ, CMTM3, CD72, SH2D1A, NCK1, PTPN22, MALT1, RC3H1, PLCL2, LAPTM5, CBLB, LYN, CD300A, UBR2, CYLD
NK1	REGULATION OF NATURAL KILLER CELL MEDIATED IMMUNITY	2,28	7,97E-04	49	27	INPP5D, SLAMF6, SH2D1B, HLA-E, HLA-F, TIGIT, IL18RAP, KLRD1, NCR1, KLRK1, TGFB1, STAT5B, CD226, SERPINB9, HLA-A, NCR3, CRTAM, KLRC4, SH2D1A, CD96, HAVCR2, RASGRP1, LILRB1, HLA-C, VAV1,
NK1	NATURAL KILLER CELL MEDIATED IMMUNITY	2,08	1,05E-03	73	37	PTPN6, INPP5D, SLAMF7, SLAMF6, RAB27A, FCGR3A, SH2D1B, HLA-E, HLA-F, TIGIT, IL18RAP, KLRD1, NCR1, KLRK1, TGFB1, STAT5B, CD226, UNC13D, LYST, SERPINB9, CORO1A, HLA-A, NCR3, CRTAM, HCST, KLRC4, SH2D1A, CD96, HAVCR2, RASGRP1, VAMP2, LILRB1, HLA-C, KIR3DL1, VAV1, ARRB2, B2M
NK2	CYTOPLASMIC TRANSLATION	2,87	2,28E-17	163	95	RPL13, RPS3, RPS4X, PABPC1, RPL7A, RPL26, RPS25, RPS15, RPL41, RPS27, RPS19, RPL23A, RPLP2, RPS3A, RPL27A, EEF1D, RPS6, RPL36, RPS12, RPL39, RPS8, RPL29, RPL10, RPL13A, RPL3, RPL27, RPS14, RPL28, RPS27A, RPL6, RPS29, RPL34, RPS10, RPLP1, RPS15A, RPS23, NEMF, RPL18, HNRNPD, RPL22L1, RPL24, RPL11, DHX29, RPL32, EIF3F, RPL17, RPL21, RPS11, FAU, UBA52, RPL30, RPS20, RPL18A, RACK1, RPL31, RPS24, RPL37, RPS7, RPL4, RPS28, RPL12, RPL23, RPS17, RPL36AL, RPS21, RPS18, EIF5, ALKBH3, RPL35A, RPS5, RPS2, RPL22, RPSA, RPL35, ZNF385A, EIF3CL, RPL38, RPL36A, RPL14, FMR1, EIF4A1, DHX36, RPS13, RPL7, RPS26, EEF2, RPL10A, EIF3K, RPS9, RPL8, RPLP0, RPL5, RPL37A,

NK2	NEGATIVE REGULATION OF TYPE II INTERFERON PRODUCTION	1,97	2,95E-02	30	16	IRGM, DDIT3, CD96, SLAMF1, TNFSF4, LILRB4, ZNF683, LAPTM5, HLA-DRB1, NLRP6, GATA3, IL20RB, LGALS9C, TLR4, RARA, ZC3H12A
NK2	POSITIVE REGULATION OF LEUKOCYTE CELL CELL ADHESION	1,68	5,17E-03	229	90	CCR7, TMIGD2, CD27, PNP, PIK3R6, TNFSF11, EGR3, ABL1, SOCS1, SIRPG, PHF10, CCL2, NOD2, LEF1, HLA-DMB, TNFSF13B, SLAMF1, IL2RA, TNFSF9, TNF, SMARCD3, TNFSF4, HLA-DPB1, EPHA4, NKAP, TNFSF14, IL23A, FUT7, CD40LG, BRD2, IL23R, HLA-DMA, NFKBID, SART1, LILRB4, AGER, IFNG, CD209, HLA-G, AP3D1, HLA-DQA1, NCK2, NR4A3, CD74, TESPA1, CCDC88B, ARID1B, BRD7, SYK, CD5, HLA-DPA1, ABL2, FCHO1, KLHL22, VCAM1, BTN2A2, NFKBIZ, ZMIZ1, IL1B, CD83, HLA-DRB1, ICOS, DPP4, DOCK8, GATA3, HLA-DRA, HLX, IL4I1, KAT5, CYLD, HLA-DRB5, DUSP10, CD28, CORO1A, MALT1, STAT5A, CCR2, SMARCC2, BCL6, EP300, BRD4, ARID1A, NLRP3, IL12RB2, EBI3, HLA-DOA, FBXO38, NFAT5, RARA, CD1D
NK2	LEUKOCYTE CHEMOTAXIS	1,86	1,26E-03	157	46	CCR7, JAML, KIT, GPR35, SLC8B1, TNFSF11, RPS19, ITGA1, STAP1, CCL4L2, VEGFB, GPR18, ZNF580, RPL13A, CKLF, CCL2, CCR5, CCR1, CCL3, CCL22, SLAMF1, CCR6, C5, IL17RA, CXCR3, CXCR5, TNFRSF11A, CXCL3, TNFSF14, IL23A, CSF1, PDE4B, JAM3, MPP1, CD74, CNR2, SYK, PTK2B, MAPK3, CCL8, DUSP1, DAPK2, IL1B, SWAP70, C5AR2, IL16

NK2	OXIDATIVE PHOSPHORYLATION	-1,89	3,09E-03	121	52	SDHA, GHITM, NDUFS1, NDUFA5, DNAJC30, DLD, UQCRFS1, ATPSCKMT, VCP, C2orf69, ATP5F1A, SDHD, NDUFB7, NDUFC2, MSH2, SLC25A23, SDHC, ATP5PB, SDHB, ATP5F1B, NDUFC2-KCTD14, PPIF, NDUFAB1, MTCH2, NDUFA7, CHCHD2, NDUFA12, COX8A, ETFRF1, NDUFS3, NDUFB4, CCNB1, UQCRC2, RHOA, NDUFS2, NDUFS6, UQCRC1, COX6B1, NDUFB5, NDUFB9, COQ9, PARK7, COX6A1, ATP5F1C, NDUFA6, COX5B, UQCRQ, NDUFB10, NDUFA2, CYC1, BID, NDUFA10
NK2	MITOCHONDRIAL GENE EXPRESSION	-1,76	4,33E-03	169	75	FASTKD5, MRPS30, TFAM, PRKAA1, TRMT10C, GFM1, LARS2, MRPS10, IARS2, MRPL50, NSUN3, TRMT61B, MRPL58, FOXO3, MRPL19, MRPS16, MRPS17, PRORP, MRPL35, TACO1, SUPV3L1, MRPS22, MRPS18B, MRPL17, TFB2M, MRPL44, MRPS14, TRMT5, TBRG4, OSGEPL1, MRPS18A, MRPL47, MRPS27, YRDC, MRPL30, GATC, MRPL9, DARS2, FASTKD1, METTL8, GFM2, MRPL55, LRPPRC, FASTKD3, MRPL34, SARS2, RARS2, MRPS9, PTCD3, MTRES1, MRPL1, MALSU1, MPV17L2, MRPL40, MRPL27, CHCHD1, MRPL12, MRPL42, MTERF2, MRPS23, MRPL15, MRPS35, ANGEL2, MRPL46, MRPL18, MRPL32, MRPL3, MTPAP, YARS2, MRPL48, MRPS12, MTRF1, TSFM, NGRN, DAP3
NK2	TRICARBOXYLIC ACID CYCLE	-2,18	3,60E-03	29	16	SDHA, OGDH, DLD, PDHB, DLAT, NNT, DLST, CS, SDHD, ACO1, IDH3A, SDHC, SDHB, MDH1, IDH2, SUCLA2

NK2	CELL CHEMOTAXIS	1,79	9,92E-04	205	59	CCR7, JAML, KIT, GPR35, SLC8B1, TNFSF11, RPS19, ITGA1, STAP1, CCL4L2, CXCR6, VEGFB, EGR3, GPR18, ZNF580, RPL13A, CCRL2, CKLF, CCL2, ENG, CCR5, CCR1, CCL3, LEF1, CCL22, SLAMF1, CCR6, C5, IL17RA, CXCR3, CXCR5, ARRB2, TNFRSF11A, CXCL3, TNFSF14, CCR9, IL23A, GAB1, CSF1, PDE4B, JAM3, CXCL2, P2RX4, MPP1, CD74, CNR2, SYK, NR4A1, VCAM1, PTK2B, MAPK3, CCL8, DUSP1, DAPK2, IL1B, TMSB4X, SWAP70, C5AR2, IL16
NK2	MITOCHONDRIAL TRANSLATION	-1,64	2,71E-02	130	60	MRPS30, TRMT10C, GFM1, LARS2, MRPS10, IARS2, MRPL50, NSUN3, MRPL58, MRPL19, MRPS16, MRPS17, MRPL35, TACO1, MRPS22, MRPS18B, MRPL17, MRPL44, MRPS14, MRPS18A, MRPL47, MRPS27, MRPL30, GATC, MRPL9, DARS2, METTL8, GFM2, MRPL55, LRPPRC, FASTKD3, MRPL34, SARS2, RARS2, MRPS9, PTCD3, MRPL1, MALSU1, MPV17L2, MRPL40, MRPL27, CHCHD1, MRPL12, MRPL42, MRPS23, MRPL15, MRPS35, MRPL46, MRPL18, MRPL32, MRPL3, YARS2, MRPL48, MRPS12, MTRF1, TSFM, NGRN, DAP3, EARS2, MRPL20

NK2	POSITIVE REGULATION OF CELL CELL ADHESION	1,62	9,70E-03	259	98	CCR7, TMIGD2, CD27, PNP, PIK3R6, TNFSF11, EGR3, ABL1, SOCS1, SIRPG, MMRN1, GP6, PHF10, CCL2, NOD2, LEF1, HLA-DMB, TNFSF13B, SLAMF1, IL2RA, TNFSF9, TNF, SMARCD3, TNFSF4, HLA-DPB1, EPHA4, NKAP, TNFSF14, IL23A, FUT7, CD40LG, BRD2, IL23R, HLA-DMA, NFKBID, SART1, LILRB4, AGER, IFNG, CD209, HLA-G, AP3D1, HLA-DQA1, RNASE10, NCK2, NR4A3, CD74, TESPA1, CCDC88B, ARID1B, BRD7, SYK, CD5, HLA-DPA1, ABL2, FCHO1, KLHL22, VCAM1, BTN2A2, NFKBIZ, EMILIN1, PIEZO1, ZMIZ1, IL1B, CD83, HLA-DRB1, ICOS, DPP4, DOCK8, GATA3, HLA-DRA, HLX, IL4I1, KAT5, CYLD, KIF26B, HLA-DRB5, DUSP10, CD28, CORO1A, MALT1, STAT5A, CCR2, FLOT1, SMARCC2, BCL6, ADAM19, EP300, BRD4, ARID1A, NLRP3, IL12RB2, EBI3, HLA-DOA, FBXO38, NFAT5, RARA, CD1D
NK2	ELECTRON TRANSPORT CHAIN	-1,77	1,73E-02	101	49	SDHA, GHITM, NDUFS1, NDUFA5, DLD, UQCRFS1, SDHD, NDUFB7, NDUFC2, SDHC, SDHB, NDUFC2-KCTD14, NDUFAB1, MTCH2, NDUFA7, CHCHD2, DHRS2, NDUFA12, COX8A, NDUFS3, NDUFB4, CCNB1, UQCRC2, NDUFS2, NDUFS6, UQCRC1, COX6B1, NDUFB5, NDUFB9, COQ9, PARK7, COX6A1, NDUFA6, COX5B, UQCRQ, NDUFB10, NDUFA2, CYC1, FDX1, BID, HCCS, NDUFA10, PUM2, COX7A2, COX7B, NDUFA4, CYCS, GBA1, CDK1

NK2	ATP SYNTHESIS COUPLED ELECTRON TRANSPORT	-2,02	8,85E-04	79	44	SDHA, GHITM, NDUFS1, NDUFA5, DLD, UQCRFS1, SDHD, NDUFB7, NDUFC2, SDHC, SDHB, NDUFC2-KCTD14, NDUFAB1, MTCH2, NDUFA7, CHCHD2, NDUFA12, COX8A, NDUFS3, NDUFB4, CCNB1, UQCRC2, NDUFS2, NDUFS6, UQCRC1, COX6B1, NDUFB5, NDUFB9, COQ9, PARK7, COX6A1, NDUFA6, COX5B, UQCRQ, NDUFB10, NDUFA2, CYC1, BID, NDUFA10, COX7A2, COX7B, NDUFA4, CYCS, CDK1
